# Generation of 1,2-oxathiolium ions from (arysulfonyl)- and (arylsulfinyl)allenes in Brønsted acids. NMR and DFT study of these cations and their reactions

**DOI:** 10.3762/bjoc.14.268

**Published:** 2018-11-22

**Authors:** Stanislav V Lozovskiy, Alexander Yu Ivanov, Olesya V Khoroshilova, Aleksander V Vasilyev

**Affiliations:** 1 Department of Organic Chemistry, Institute of Chemistry, Saint Petersburg State University, Universitetskaya nab., 7/9, Saint Petersburg, 199034, Russia; 2 Center for Magnetic Resonance, Research Park, St. Petersburg State University, Universitetskiy pr., 26, Saint Petersburg, Petrodvoretz, 198504 , Russia; 3 Department of Chemistry, Saint Petersburg State Forest Technical University, Institutsky per., 5, Saint Petersburg, 194021, Russia

**Keywords:** (arylsulfinyl)allenes, (arylsulfonyl)allenes, butadienes, 1,2-oxathiolium ions, thiochromene 1,1-dioxides

## Abstract

In strong Brønsted acids (CF_3_SO_3_H, FSO_3_H, D_2_SO_4_), (arysulfonyl)allenes (ArSO_2_–CR^1^=C=CR^2^R^3^) and (arylsulfinyl)allenes (ArSO–CR^1^=C=CR^2^R^3^) undergo cyclization into the corresponding stable 1,2-oxathiolium ions, which were studied by means of NMR and DFT calculations. Quenching of solutions of these cations with low nucleophilic media, aqueous HCl, leads to their deprotonation with a stereoselective formation of (arysulfonyl)butadienes (for instance, ArSO_2_–CR^1^=C–C(Me)=CH_2_, for R^2^ = R^3^ = Me, yields of 87–98%). Reactions of (arysulfonyl)allenes in the system TfOH (0.1 equiv)–HFIP (hexafluoropropan-2-ol) followed by hydrolysis give rise to allyl alcohols (ArSO_2_–CR^1^=CH–C(OH)R^2^R^3^, yields of 78–99%). Reflux of solutions of (arysulfonyl)allenes in the presence of TfOH (1 equiv) in 1,2-dichlorobenzene leads to the cyclization into thiochromene 1,1-dioxides in high yields. Under the action of TfOH or AlX_3_ (X = Cl, Br) followed by hydrolysis of reaction mixtures, (arylsulfinyl)allenes give allyl alcohols (ArSO_2_–CR^1^=CH–C(OH)R^2^R^3^). Plausible reaction mechanisms have been proposed for all studied reactions.

## Introduction

Allenes are widely explored in organic synthesis for the construction of various molecules [1–7]. In particular, arylsulfonyl (ArSO_2_) allenes are usefull building blocks in miscellaneous transformations. For instance, addition of such allenes to Michael acceptors leading to terminal acetylenes has been recently shown [8]. These allenes give rise to pyrrolidines [9], pyrroles [10], chromenes [11], benzoazepinones [12], macrolides [13], and some other carbo- and heterocycles [14–16]. It should be specially emphasized that many compounds containing SO_2_ groups are drugs, such as, dapson [17], oxicams [18], or amisulpride [19]. Substantial contribution in this area was made by Harmata et al. [20–24]. However, to the best of our knowledge, electrophilic reactions of (arylsulfonyl)allenes have not been widely studied yet. It has been shown by Ma et al. that ArSO_2_-allenes take part into halogenohydroxylation (Hal = I, Br) or addition–elemination of bromine (forming bromobutadienes) with high stereoselectivity [25–26]. Apart from that, reactions of sulfur containing allenes were studied in acidic media [27–28]. Despite promising results, there was no further research in this area.

**Scheme 1:**
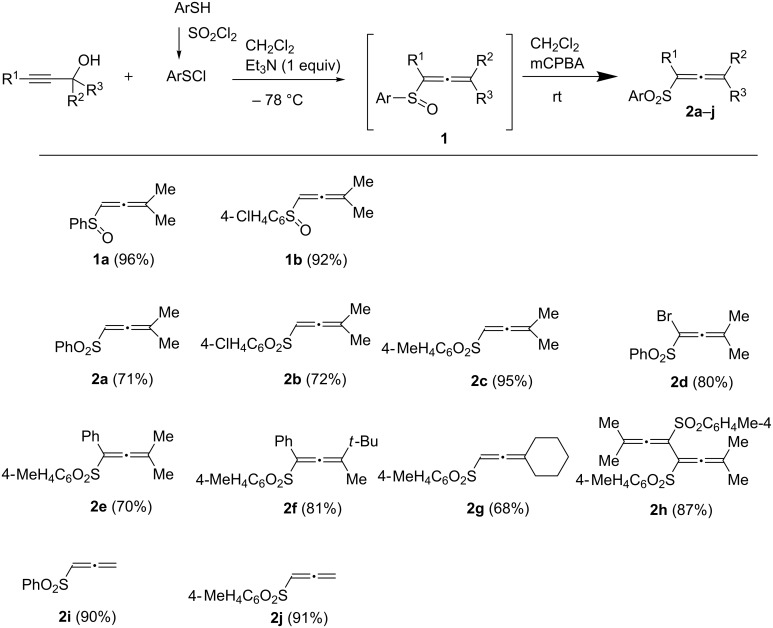
(Arylsulfinyl)allenes **1** and (arylsulfonyl)allenes **2** used in this study.

Based on our recent work on transformations of phosphonoallenes under the action of strong Brønsted or Lewis acids [29–32], we undertook a special study on reactions of (arylsulfonyl)allenes **2a**–**j** and (arylsulfinyl)allenes **1a**,**b** (Scheme 1).

**Figure 1:**
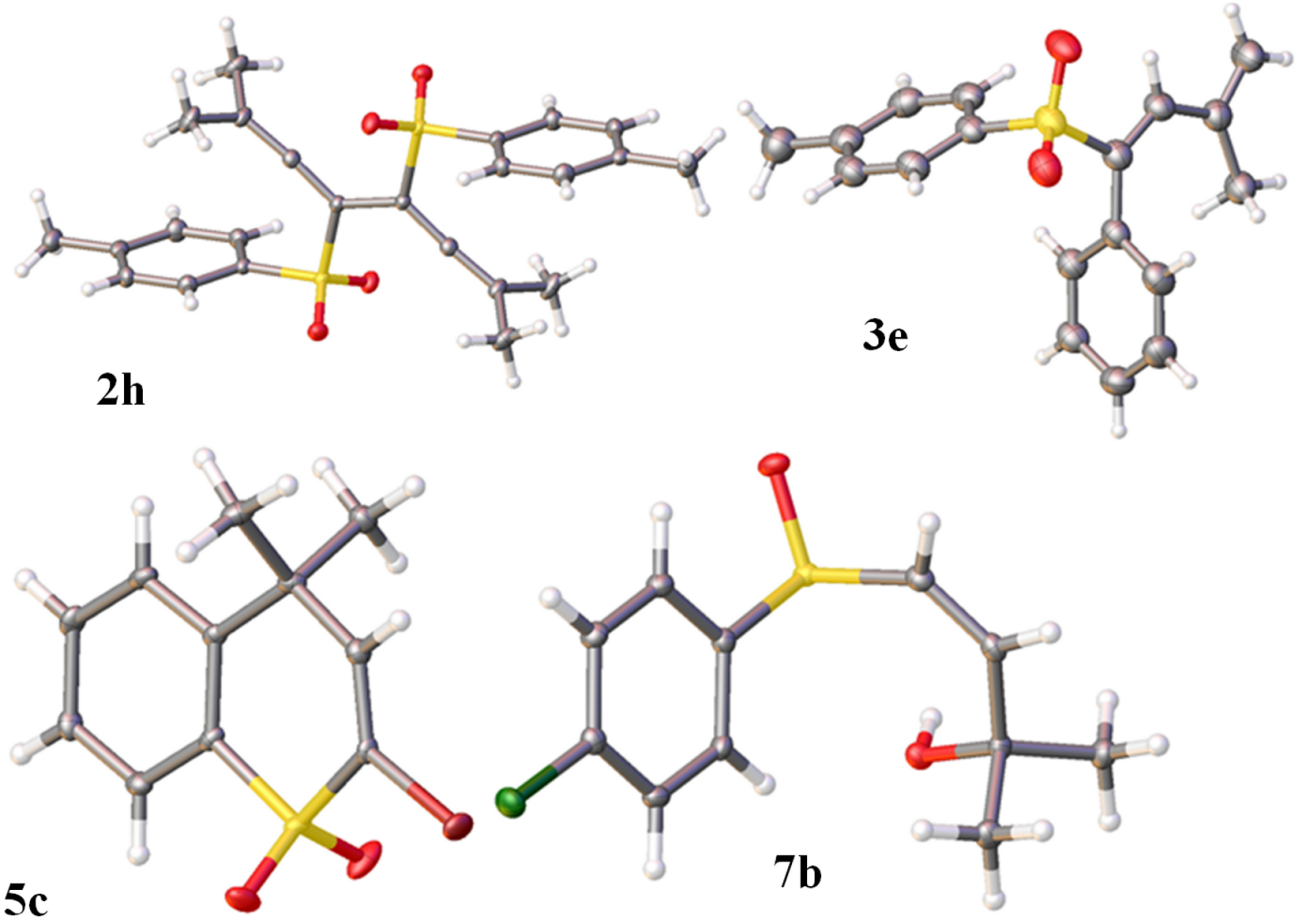
X-ray crystal structures of compounds **2h** (CCDC 1843276), **3e** (CCDC 1843277), **5c** (CCDC 1580895), **7b** (CCDC 1843239); ellipsoid contours of probability levels are 50%.

The reaction between proparylic alcohols and arylsulfanyl chlorides followed by acetylene–allene rearrangement was used to prepare (arylsulfinyl)allenes **1** according to the literature procedure [24–25]. The latter were in situ oxidized to (arylsulfonyl)allenes **2** (see X-ray structure of **2h** in Figure 1). Allenes **1a**,**b** were specially isolated to compare their reactivity with allenes **2**.

The main goals of this work were the investigation of reactions of sulfur-containing allenes **1** and **2** under electrophilic activation with Brønsted or Lewis (super)acids, and the study on cationic intermediates of these reactions by means of NMR and DFT calculations.

## Results and Discussion

First, the behavior of allenes **1a**,**b** and **2a**–**h** in Brønsted acids (TfOH, D_2_SO_4_) was studied by means of NMR (Table 1). Dissolving these allenes in TfOH or D_2_SO_4_ directly in NMR tubes at room temperature gave intensively colored red solutions of cationic species, which were stable for a long time. The NMR data, including ^1^H, ^13^C, DEPT, COSY, and HSQC spectra (see Supporting Information File 1), demonstrated unambiguously that compounds **1a,b** and **2a**–**h** underwent cyclization into the corresponding ions **Aa**,**b** and **Ba**–**h** via protonation (deuteration for D_2_SO_4_, **Bd-*d***) of the central carbon allenic triad followed by nucleophilic attack of oxygen of the SO_2_ group (for **Ba**–**h**) or SO group (for **Aa**,**b**) onto the carbocationic center. The similar cyclization was observed for phosphonoallenes (see **P1**, **P2**, Table 1) by us previously [30,32]. A new signal of the attached proton H4 at δ 8.05–6.83 ppm range appeared in ^1^H NMR spectra of species **A**, **B**. The comparison of ^13^C NMR spectra of oxathiolylium **A**, **B** and oxaphospholium **P1**, **P2** ions shows that for the former species the signal of carbon C5 is about 10–15 ppm downfield shifted relatively the same signal in the cations **P1**, **P2** (Table 1). This reveals that carbon C5 bears a rather large positive charge in cations **A**, **B**. For dication **Bh**, different signals were detected for quaternary carbons C5 and C5', and vinyl carbons C4 and C4', etc., that, probably, indicates formation of two diastereomers (one *meso*-form and one pair of enantiomers) due to the stereogenic sulfur centers.

**Table 1:** Selected ^1^H and ^13^C NMR data for cations **Aa**,**b** and **Ba**–**h**, **P1**, **P2** derived at the protonation of the corresponding allenes at room temperature in TfOH and D_2_SO_4_.

initial allene	cation	acid	^1^H NMR, δ, ppm		^13^C NMR, δ, ppm		
			
			H3	H4	C3	C4	C5

**1a**	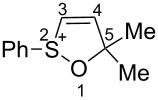 **Aa**	TfOH	6.85 d (*J* = 5.7 Hz)	7.47 d (*J* = 5.7 Hz)	117.2	150.2	112.4
**1b**	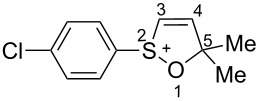 **Ab**	TfOH	6.83 d (*J* = 6.1 Hz)	7.51 d (*J* = 6.1 Hz)	117.2	150.6	112.8
**2a**	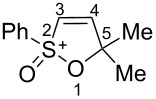 **Ba**	TfOH	7.16 d (*J* = 6.2 Hz)	8.05 d (*J* = 6.2 Hz)	121.0	158.7	112.0
**2b**	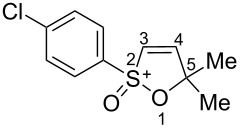 **Bb**	TfOH	7.17 d (*J* = 6.0 Hz)	8.07 d (*J* = 6.0 Hz)	122.4	159.6	113.0
**2c**	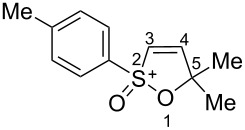 **Bc**	TfOH	7.12 d (*J* = 6.2 Hz)	8.01 d (*J* = 6.2 Hz)	121.3	157.1	110.2
**2d**	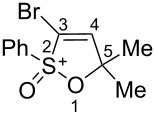 **Bd**	TfOH	–	7.95 s	109.9	153.0	112.7
**2d**	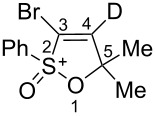 **Bd-*d***	D_2_SO_4_	–	–	109.0	153.0	113.0
**2e**	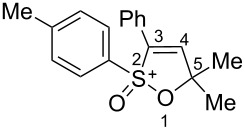 **Be**	TfOH	–	7.91	123.4	154.1	110.0
**2f**	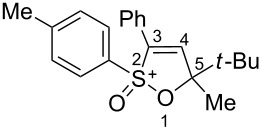 **Bf** 2 isomers in a ratio of 5:1^a^	TfOH	–	8.02	115.6	154.1	119.3
**2g**	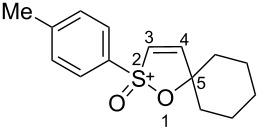 **Bg**	TfOH	7.11 d (*J* = 6.2 Hz)	8.02 d (*J* = 6.2 Hz)	122.6	157.7	114.7
**2h**	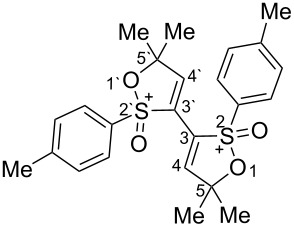 **Bh**	TfOH	–	8.35 and 8.09	120.9 and 120.7	157.9 and 157.1	112.8 and 112.2
Data from ref. [30]	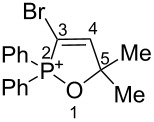 **P1**	TfOH	–	7.78 d (*J*_HP_ = 28 Hz)	102.0	164.3	103.6
Data from ref. [32]	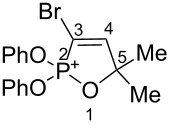 **P2**	TfOH	–	7.84 d (*J*_HP_ = 46 Hz)	96.0	169.1	102.8

^a^NMR data for major isomer.

In the case of the cation **Bf**, the signals of two isomers were found in the spectra in a ratio of 5 to 1. These isomers appear due to *cis*-, *trans*-orientation of *t-*Bu and ArS groups in the five-membered ring.

To the best of our knowledge, this is one of the first examples of full NMR characterization of such broad series of cyclic sulfur containing cations **Aa**,**b** and **Ba**–**h.** Allenes **2i**,**j** did not react with acids at room temperature, however, they react with TfOH at higher temperature (see below).

To estimate the charge distribution in species **Aa**, **Ba** we carried out DFT calculations (Table 2). The calculations confirm the experimental NMR data (Table 1) and prove that C5 does have a large positive charge 0.25 e, which should make this carbon a highly reactive electrophilic center. Another electrophilic center is the sulfur atom, which also bears a large positive charge (1.21–2.06 e). Apart from that, the atomic coefficient of contribution in the LUMO for sulfur is much higher than for C5. Thus, the electrophilic reactivity of sulfur may be explained by both charge and orbital control. Also, *ortho-*carbons in the phenyl group bear a negative charge −0.17 to −0.16 e; this means that intramolecular cyclization on these atoms is possible.

**Table 2:** Selected electronic characteristics of cations **Aa**, **Ba** generated from allenes **1a**, **2a**, correspondingly (DFT calculations).

cation	q(S)^a^ e	q(C3)^a^ e	q(C4)^a^ e	q(C5)^a^ e	q(C*_o_*_-Ph_)^a^ e	k_LUMO_^b^ %				
						
						S	C3	C4	C5	∑C_Ph (_*_ortho_*_ + _*_para_*_ + _*_ipso_*_)_

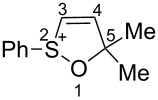 **Aa**	1.21	−0.38	−0.13	0.25	−0.17	35	4	0	2	38
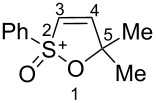 **Ba**	2.06	−0.43	−0.10	0.25	−0.16	11	14	25	1	47

^a^Natural charges. ^b^Contribution of atomic orbital into the molecular orbital.

To conclude the study on electronic characteristics of 1,2-oxathiolylium ions **A**, **B** by means of NMR and DFT calculations, one should expect that these species may react in several pathways. First, they may undergo nucleophilic attack on sulfur or on carbon C5, due to a high positive charge on it. Another pathway may be an electrophilic cyclization at the *ortho-*carbon in the S-phenyl ring.

Then, the preparative reactions of allene **2a** under the action of different electrophilic reagents were conducted. Transformations of **2a** using an excess of various Brønsted acids followed by aqueous quenching of the reaction mixture are shown in Table 3. These reactions resulted in the formation of three different products, ***Z*-3a**, ***Z*-4a** and **5a**, depending on the reaction conditions. At room temperature in CF_3_SO_3_H or H_2_SO_4_ for a short time, 10 min or 1 h, respectively, butadiene ***Z*-3a** and alcohol ***Z*-4a** were formed (Table 3, entries 1 and 3). Increasing the reaction temperature to 60 °C and the time to 8 h in CF_3_SO_3_H led to the formation of thiochromene 1,1-dioxide **5a** (Table 3, entry 2). Decreasing the reaction temperature down to −60 °C in FSO_3_H with work-up of the superacidic reaction solution with a low nucleophilic medium (frozen aqueous HCl at −60 °C) gave almost quantitatively butadiene ***Z*-3a** with a small admixture of its *E*-isomer (Table 3, entry 4). Weaker acids, CF_3_CO_2_H or aqueous HCl, did not activate allene **2a**, no reactions took place (Table 3, entries 5 and 6). Apart from that, Lewis acids of various strength (AlCl_3_, AlBr_3_, FeCl_3_, CeCl_3_, BF_3_-Et_2_O, In(OTf)_3_) were found to be ineffective for this transformation, no reactions of allenes **2** occurred with them.

**Table 3:** Reactions of allene **2a** under the action of various Brønsted acids.

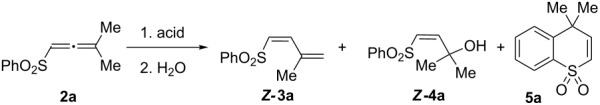						

entry	reaction conditions			reaction products, yield, %		
	
	acid (equiv)	temperature, °C	time	***Z*-3a**	***Z*-4a**	**5a**

1	TfOH (40)	rt	10 min	18	81	–
2	TfOH (40)	60	8 h	–	–	40
3	H_2_SO_4_ (40)	rt	1 h	20	20	–
4	FSO_3_H (40)^a^	−60	1 h	90 (+ ***E***-**3a**, 9%)	–	–
5	CF_3_CO_2_H (40)^b^	50	24 h	–	–	–
6	HCl_aq_ (40)^b^	rt	24 h	–	–	–

^a^Work-up with frozen aqueous HCl at −60 °C. ^b^Quantitative recovery of starting **2a**.

**Scheme 2:**
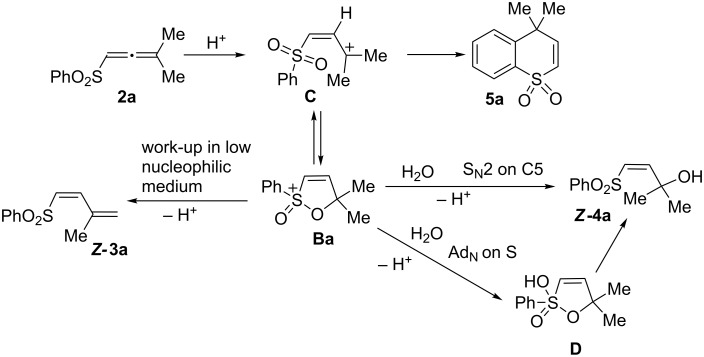
Plausible reaction mechanisms of transformations of allene **2a** in Brønsted acids.

Taking into account the data on the formation of cations **B** (Table 1), their electronic characteristics (Table 2), and reactions of allene **2a** in Brønsted acids (Table 3), one may propose a plausible mechanism for the transformation of **2a** (Scheme 2). Protonation of the allene system gives cation **C**, which is cyclized into stable species **Ba**. Upon work-up of the acidic reaction solution, the fate of the cation **Ba** strongly depends on the nucleophilicity of the quenching medium. Under the conditions of low nucleophilic work-up with aqueous HCl (Table 3, entry 4), the deprotonation takes place leading to butadiene ***Z*-3a**. The predominant formation of the *Z*-isomer of **3a** may reveal that cation **Ba** undergoes deprotonation and recyclization, rather than species **C**. Quenching of species **Ba** with water (high nucleophilicity) affords alcohol ***Z*-4a**. The formation of compound **4a** in exclusively *Z*-configuration may indicate that cation **Ba** reacts with H_2_O in S_N_2 manner, keeping in mind that carbon C5 in **Ba** possesses a large positive charge (see data in Table 1 and Table 2). An alternative mechanism of the formation of alcohol **4a** includes the attack of H_2_O on the sulfur electrophilic center giving intermediate **D**, which is rearranged into alcohol ***Z*-4a**. Preparation of sulfur heterocycle **5a** at high reaction temperature (Table 3, entry 2) shows that the intramolecular cyclization to the *ortho*-carbon of phenyl ring occurs, most probably, through cation **C** (Scheme 2). And this reaction has a high activation barrier, analogously to the similar cyclization of phosphonoallenes to the corresponding phosphonoheterocycles [30].

**Scheme 3:**
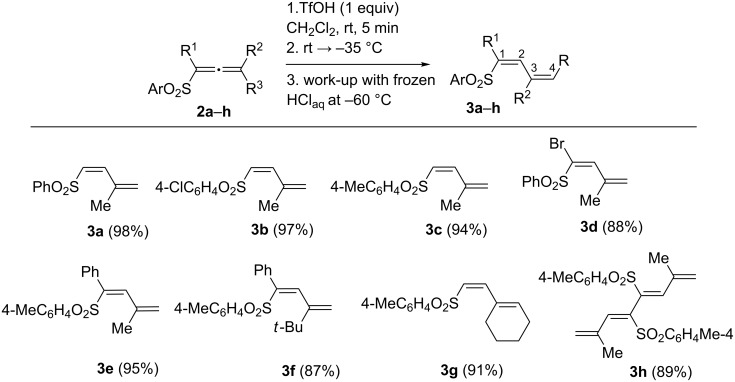
Selective formation of butadienes **3a**–**h** from allenes **2a**–**h**.

We decided to achieve the selective formation of each of these different products, butadienes **3**, adducts with nucleophiles **4**, and thiochromene 1,1-dioxides **5**, from allenes **2**. The preparation of compounds **3a**–**h** was done by the following method (Scheme 3). Reactions of **2a**–**h** were carried out in CH_2_Cl_2_ with 1 equivalent of TfOH to generate the corresponding cations **Ba**–**h**. Then, the reaction mixture was cooled down to −35 °C and quenched under very mild and low nucleophilic conditions with frozen aqueous HCl at −60 °C, that finally led quantitatively to butadienes **3a**–**h** (see X-ray structure of **3e** in Figure 1). Worth noting, that compounds **3a**–**h** have strictly *cis*-configuration of SO_2_Ar group and a vinyl substituent at C2 carbon. It should be mentioned that palladium-catalyzed isomerization of such (arylsulfonyl)allenes **2** into *trans*-butadienes **3** was described recently [23]. Herein, we have developed a novel metal-free approach for the synthesis of *cis*-isomers of **3**.

**Scheme 4:**
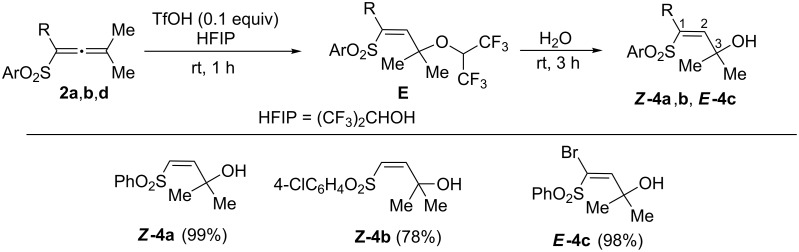
Reactions of allenes **2** in the system HFIP/TfOH followed by interaction with nucleophiles leading to allyl alcohols **4**.

Then, we tried to get selectively products of the nucleophilic attack onto cations **B** (like structure ***Z*-4a** in Scheme 2) by quenching of the acidic reaction solutions (in TfOH) with various nucleophiles (water, methanol, benzene, acetonitrile). But, in all cases, these reactions were unselective. For instance, for allene **2a**, mixtures of butadiene ***Z*-3a** and alcohol ***Z*-4a** were obtained. To overcome this obstacle we decided to use 1,1,1,3,3,3-hexafluoropropan-2-ol (HFIP), which was known to form the corresponding ether for further substitution reactions [33]. Indeed, the use of HFIP and a catalytic amount of TfOH (0.1 equiv) followed by hydrolysis allowed to achieve an exclusive formation of allyl alcohols ***Z*-4a**,**b** and ***E*-4c** from allenes **2a**,**b**,**d**, respectively, in high yields (Scheme 4). The most probably, this reaction proceeds through intermediate formation of ethers **E** from the corresponding species **B**. The ethers **E** are hydrolyzed to compounds **4**. Reactions of allenes **2** with other nucleophiles (methanol, benzene, acetonitrile) led to the formation of complex mixtures of reaction products. It must be noted that no reaction proceeded in HFIP without TfOH.

The assignment of the *cis*-configuration of the ArSO_2_ group and the C3-substituent in compounds **3** and **4** was based on the low spin–spin interaction constant of 8.0–11.8 Hz between the vinyl protons in the ^1^H NMR spectrum (see Supporting Information File 1) and on comparison with the known *trans*-isomers of **3** [23].

**Scheme 5:**
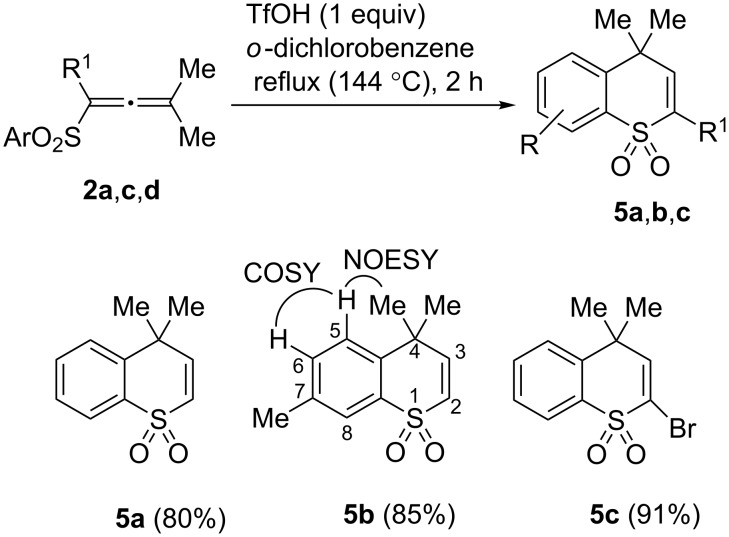
Formation of thiochromene 1,1-dioxides **5a**–**c** from allenes **2a**,**c**,**d**.

Compounds ***Z*-3a** and ***Z*-4a** could be interconverted in acids through species **Ba** (Scheme 2). Thus, both ***Z*-3a** and ***Z*-4a** give cation **Ba** upon dissolving in TfOH. Then, a different quenching of solution of the cation affords ***Z*-3a** (Scheme 3) or ***Z*-4a** (Scheme 5). Heating of the solution of **Ba** leads to **5a** (see Scheme 5).

The exclusive formation of thiochromene 1,1-dioxides **5a**–**c** was obtained by running the reaction of **2a**,**c**,**d** with 1 equivalent of CF_3_SO_3_H at high temperature (reflux in *ortho*-dichlorobenzene at 144 °C) for 2 h (Scheme 5, see X-ray structure of **5c** in Figure 1). It was found by H,H-NOESY and COSY correlations, that there was a [6,7]-shift of the methyl group in the thiochromene system of **5b** obtained from allene **2c**, which, at first, should give 6-methyl substituted thiochromene. This shift is caused by the action of superacid at high reaction temperature. Phenyl-substituted allenes **2e**,**f** did not afford the corresponding thiochromenes, due to oligomerization under the harsh reaction conditions. Other allenes **2g**,**h** gave the corresponding heterocycles **5** in very poor yields (<4%, by GC–MS and ^1^H NMR data).

**Scheme 6:**
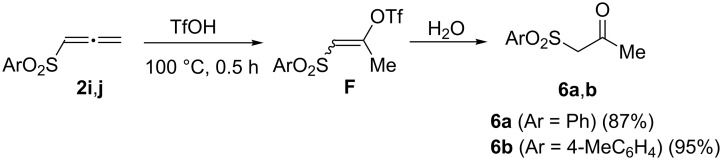
Formation of (arylsulfonyl)acetones **6a**,**b** from allenes **2h**,**j** in TfOH (100 °C, 0.5 h) followed by hydrolysis of superacidic reaction solutions.

As it was mentioned above, unsubsituted allenes **2i**,**j** did not react with TfOH at room temperature (see discussion on NMR of cations **B**, Table 1). Under the heating in TfOH at 100 °C for 0.5 h, these allenes afforded (arylsulfonyl)acetones **6a**,**b**, which may be formed under the hydrolysis of the formed vinyl triflates **F** (Scheme 6).

**Scheme 7:**
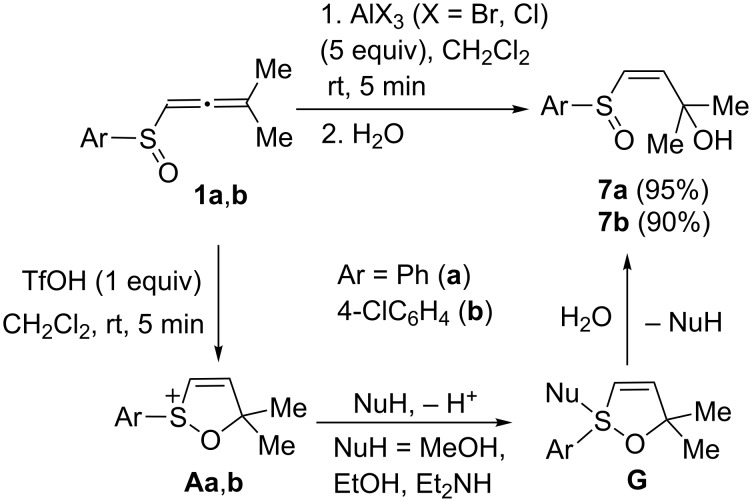
Reactions of (arylsulfinyl)allenes **1a**,**b** under superelectrophilic activation.

Finally, in this study, we carried out reactions of (arylsulfinyl)allenes **1a**,**b** under superelectrophilic activation with TfOH or AlX_3_ (X = Cl, Br). The corresponding cations **Aa**,**b** generated from **1a**,**b** in TfOH were subjected to quenching with various nucleophiles followed by hydrolysis (Scheme 7). In all the cases, allyl alcohols **7a**,**b** were isolated (see X-ray structure of **7b** in Figure 1). The same alcohols were obtained in reactions of **1a**,**b** with AlX_3_ (X = Cl, Br) after the hydrolysis of reaction solutions. In these reactions, presumably, intermediate adducts **G**, which are formed upon interaction of species **Aa**,**b** with nucleophiles, are easily hydrolazible and give rise to alcohols **7a**,**b**.

## Conclusion

Transformations of (arylsulfonyl)- and (arylsulfinyl)allenes under the action of the Brønsted superacid TfOH, or strong Lewis acids AlX_3_ (X = Cl, Br) have been studied. Under electrophilic conditions, these allenes form the corresponding 1,2-oxathiolium ions, which have been studied by NMR and DFT calculations. Depending on electrophilic activator, reaction conditions (temperature, time), and nucleophilicity of media for quenching of solutions of 1,2-oxathiolium ions, these species may undergo various transformations leading to the selective formation of one of the reaction products: conjugated dienes, allyl alcohols, or thiochromene 1,1-dioxides. These reactions open new opportunities for organic synthesis based on electrophilic activation of sulfur containing allenes.

## Supporting Information

File 1 Copies of ^1^H and ^13^C NMR spectra of compounds and cations, X-ray data, and data of DFT calculations.
