# Supplementary material for: Generation of 1,2-oxathiolium ions from (arysulfonyl)- and (arylsulfinyl)allenes in Brønsted acids. NMR and DFT study of these cations and their reactions
Source: Beilstein J Org Chem. 2018 Nov 22;14:2897–906. doi: 10.3762/bjoc.14.268 (PMC6278758; doi:10.3762/bjoc.14.268)
Supplement: File 1 — Copies of 1H and 13C NMR spectra of compounds and cations, X-ray data, and data of DFT calculations. [file Beilstein_J_Org_Chem-14-2897-s001.pdf]

BEILSTEIN JOURNAL OF ORGANIC CHEMISTRY

Supporting Information

for

Generation of 1,2-oxathiolium ions from (arysulfonyl)- and (arylsulfinyl)allenes in Brønsted acids. NMR and DFT study of these cations and their reactions

Stanislav V. Lozovskiy, Alexander Yu. Ivanov, Olesya V. Khoroshilova and Aleksander V. Vasilyev

Beilstein J. Org. Chem. **2018,** *14,* 2897–2906. doi:10.3762/bjoc.14.268

Copies of ^{1}H and ^{13}C NMR spectra of compounds and cations, X-ray data, and data of DFT calculations

License and Terms: This is a supporting information file under the terms of the Creative Commons Attribution License (http://creativecommons.org/licenses/by/4.0). Please note that the reuse, redistribution and reproduction in particular requires that the authors and source are credited.
The license is subject to the *Beilstein Journal of Organic Chemistry* terms and conditions: (https://www.beilstein-journals.org/bjoc)

Contents

I. Experimental section. Characterization of compounds...S2
II. Copies of NMR spectra..S9
III. X-ray data...S88
IV. Data of DFT calculations..S92
V. References...S145

I. Experimental section. Characterization of compounds

The NMR spectra of solutions of compounds in $CDCl_3$ or acetone-d_6 were recorded at 400, and 100 MHz for ^{1}H and ^{13}C NMR spectra, respectively, at 25 °C with Bruker-400 spectrometer. The solvent residual signals of $CDCl_3$ (δ 7.26 ppm) or acetone-d_6 (δ 2.05 ppm) for ^{1}H NMR spectra and the carbon signal of $CDCl_3$ (δ 77.0 ppm) or acetone-d_6 (δ 206.3 ppm) for ^{13}C NMR spectra were used as references. NMR spectra in TfOH or D_2SO_4 were referenced to the signal of CH_2Cl_2 added as internal standard: δ 5.32 for ^{1}H NMR spectra, and δ 54.0 ppm for ^{13}C NMR spectra respectively. IR spectra of compounds in KBr were taken with a Shimadzu IR Affinity-1 spectrometer in the region of 400–4000 cm^{-1} with resolution 4 cm^{-1}, Happ-Genzel apodization, 20 scans. HRMS was carried out with a Bruker MicroTOF instrument (ESI). The preparative reactions were monitored by thin-layer chromatography carried out on silica gel plates using UV light for detection. Preparative TLC was performed on silica gel 5–40 μm.

X-ray diffraction study. A suitable crystal was selected and studied on the Bruker diffractometer for X-ray analysis. The crystal was kept at 100(2) K during data collection. Using Olex2[1] the structure was solved with the ShelXS[2] structure solution program using Direct Methods and refined with the ShelXL refinement package using Least Squares minimization. CCDC 1843276 – (**2h**), CCDC 1843277 – (**3e**), CCDC 1580895 – (**5c**), CCDC 1843239 – (**7b**), contain the supplementary crystallographic data, which can be obtained free of charge at www.ccdc.cam.ac.uk/conts/retrieving.html or from the Cambridge Crystallographic Data Centre, 12 Union Road, Cambridge CB2 1EZ, UK; Fax: (internat.) + 44-1223-336-033; E-mail: deposit@ccdc.cam.ac.uk.

DFT calculations. All computations were carried out at the DFT/HF hybrid level of theory using hybrid exchange functional M06 by using GAUSSIAN 2009 program packages.[3] The geometries optimization were performed using the M06/6-311+G(2d,2p) basis set (standard 6-311 basis set added with polarization (d, p) and diffuse functions). Optimizations were performed on all degrees of freedom and gas-phase optimized structures were verified as true minima with no imaginary frequencies. The Hessian matrix was calculated analytically for the optimized structures in order to prove the location of correct minima and to estimate the thermodynamic parameters.

Allenes **1a,b** and **2a–2j** were synthesized according to the literature procedure.[4] Spectral characteristics of the following compounds correspond to the literature data: 3-methyl-1-(phenylsulfinyl)buta-1,2-diene (**1a**),[4] 3-methyl-1-(4-chlorophenylsulfonyl)buta-1,2-diene (**1b**),[5] 3-methyl-1-(4-chlorophenylsulfonyl)buta-1,2-diene (**2b**),[6] 3-methyl-1-(4-methylphenylsulfonyl)buta-1,2-diene (**2c**),[7] 2-(4-methylphenylsulfonyl)-ethenylidenecyclohexane (**2g**),[7] 1-(phenylsulfonyl)propa-1,2-diene (**2i**),[8] 1-(4-methylphenylsulfonyl)propa-

1,2-diene (**2j**),[8] 1-(4-methylphenylsulfonyl)propan-2-one (**6a**),[9] 1-(4-methylphenylsulfonyl)propan-2-one (**6b**),[9] (Z)-2-methyl-4-(phenylsulfinyl)but-3-en-2-ol (**7a**).[10]

3-Methyl-1-(phenylsulfonyl)buta-1,2-diene (2a). Yield 2.37 g (71%). Colorless oil. **^{1}H NMR** (400 MHz, $CDCl_3$) δ, ppm: 7.86 – 7.79 (m, 2H), 7.50 – 7.44 (m, 3H), 6.00-6.05 (m, 1H), 1.69 (d, J = 2.8 Hz, 6H); **^{13}C NMR** (100 MHz, $CDCl_3$) δ, ppm: 203.7, 141.3, 133.2, 129.1, 127.4, 107.0, 98.8, 19.3; **IR** (KBr), cm^{-1}: 1306 (S=O), 1969 (C=C=C); **HRMS(ESI)**: m/z calcd for $C_{11}H_{12}NaO_2S$ [M+Na]$^+$ 231.0450 , found 231.0447.

1-Bromo-3-methyl-1-(phenylsulfonyl)buta-1,2-diene (2d). Yield 3.15 g (80%). Colorless solid, mp 77-78 °C. **^{1}H NMR** (400 MHz, $CDCl_3$) δ, ppm: 7.98 – 7.95 (m, 2H), 7.70 – 7.66 (m, 1H), 7.61 – 7.56 (m, 2H), 1.92 (s, 6H).; **^{13}C NMR** (100 MHz, $CDCl_3$) δ, ppm: 201.0, 138.3, 133.8, 129.0, 128.8, 114.7, 89.6, 19.9; **IR** (KBr) cm^{-1}: 1321 (S=O), 1956(C=C=C); **HRMS (ESI)**: m/z calcd for $C_{11}H_{11}BrNaO_2S$ [M+Na]$^+$ 308.9555 , found 308.9560.

3-Methyl-1-(4-methylphenylsulfonyl)-1-phenylbuta-1,2-diene (2e). Yield 2.54 g (70%). Greenish solid, mp 66-68°C. **^{1}H NMR** (400 MHz, $CDCl_3$) δ: 7.75-7.70 (m, 2H), 7.50-7.47 (m, 4H), 7.33-7.28 (m, 3H), 2.42 (s, 3H), 1.83 (s, 6H). ^{13}C NMR (101 MHz, Acetone) δ 202.8, 144.0, 137.8, 129.4, 129.4, 128.6, 128.3, 127.9, 127.9, 106.7, 21.1, 19.3. **HRMS (ESI)**: m/z calcd for $C_{18}H_{18}NaO_2S$ [M+Na]$^+$ 321.0925 , found 321.0935.

3,4,4-Trimethyl-1-(4-methylphenylsulfonyl)-1-phenylpenta-1,2-diene (2f). Yield 1.56 g (81%). Yellow oil. **^{1}H NMR** (400 MHz, $CDCl_3$) δ: 8.03 (d, J = 7.8 Hz, 2H), 7.74 (d, J = 8.2 Hz, 2H), 7.48 – 7.40 (m, 2H), 7.36 – 7.29 (m, 3H), 2.42 (s, 3H), 1.81 (s, 3H), 1.08 (s, 9H). 13C NMR (101 MHz, $CDCl_3$) δ: 201.4, 144.0, 137.8, 137.8, 129.3, 129.3, 128.6, 128.6, 128.4, 128.3, 119.8, 35.1, 28.5, 21.5, 14.5. **HRMS (ESI)**: m/z calcd for $C_{21}H_{24}NaO_2S$ [M+Na]$^+$ 363.1395, found 363.1396.

2,7-Dimethyl-4,5-bis(4-methylphenylsulfonyl)octa-2,3,5,6-tetraene (**2h**). Yield 2.12 g (87%). Colorless solid, mp 55°C. Structure confirmed by single crystal X-ray analysis (see SI). **^{1}H NMR** (400 MHz, $CDCl_3$) δ: 7.62 – 7.59 (m, 2H), 7.28 – 7.25 (m, 2H), 2.45 (s, 3H), 1.77 (s, 6H). **^{13}C NMR** (101 MHz, $CDCl_3$) δ: 204.7, 144.0, 137.5, 129.3, 127.8, 124.9, 109.5, 19.4, 9.3. **HRMS (ESI)**: m/z calcd for $C_{24}H_{26}Na_2O_4S_2$ [M+2Na]$^+$ 488.1068 , found 488.1063.

General procedure for synthesis of butadienes 3a-h from allenes 2a-h

Small scale procedure. 0.144 mmol of allene **2** was added to a solution of 21.6 mg (0.144 mmol) TfOH in CH_2Cl_2 (1 ml) with vigorous stirring. After 5 min the mixture was cooled to −35 °C and quenched with chilled aqueous HCl (−60°C). Reaction mixture was diluted with CH_2Cl_2 (25 ml). Organic phase was washed with water, a saturated aqueous solution of $NaHCO_3$, water, and dried with Na_2SO_4, the solvent was distilled off under reduced pressure to give pure reaction products.

Large scale procedure (it was checked for the synthesis of compounds **3a** and **3c**). 4.8 mmol of allene **2** was added to a solution of 0.72 g (4.8 mmol) TfOH in CH_2Cl_2 (15 ml) with vigorous stirring. After 5 min mixture was cooled to −35 °C and quenched with chilled aqueous HCl (−60 °C). Reaction mixture was diluted with CH_2Cl_2 (100 ml). The organic phase was washed with water, with a saturated aqueous solution of

$NaHCO_3$, water, and dried with Na_2SO_4, the solvent was distilled off under reduced pressure to give pure reaction products.

(1*Z*)-3-Methyl-1-(phenylsulfonyl)buta-1,3-diene (3a). Obtained from **2a** (1.00 g, 4.8 mmol or 30.0 mg, 0.144 mmol). Yield 0.98 g or 28.5 mg (98% or 95%). Colorless oil. **^{1}H NMR** (400 MHz, $CDCl_3$) δ: 7.95 – 7.91 (m, 1H), 7.66 – 7.61 (m, 2H), 7.58 – 7.53 (m, 2H), 6.55 (d, *J* = 11.8 Hz, 1H), 6.36 (d, *J* = 11.8 Hz, 1H), 5.27 (s, 1H), 5.24 (s, 1H), 1.87 (s, 3H); **^{13}C NMR** (101 MHz, $CDCl_3$) δ: 143.7, 141.5, 138.1, 133.2, 130.8, 128.9, 127.7, 121.4, 21.7; **IR** (KBr), cm^{-1}: 1311 (S=O), 1631(C=C), 1615 (C=C); **IR** (KBr), cm^{-1}: 1311 (S=O), 1631(C=C), 1615 (C=C); **HRMS(ESI)**: m/z calcd for $C_{11}H_{12}NaO_2S$ [M+Na]$^+$ 231.0456, found 231.0456.

(1*Z*)-1-(4-Chlorophenylsulfonyl)-3-methylbuta-1,3-diene (3b). Obtained from **2b** (38.0 mg, 0.144 mmol). Yield 37.1 mg (97%) Solidifying colorless oil. **^{1}H NMR** (400 MHz, $CDCl_3$) δ: 7.89 – 7.85 (m, 2H), 7.54 – 7.50 (m, 2H), 6.58 (d, *J* = 11.8 Hz, 1H), 6.34 (d, *J* = 11.8 Hz, 1H), 5.27 (1H), 5.25 (1H) 1.87 (1H).**^{13}C NMR** (101 MHz, $CDCl_3$) δ: 144.2, 140.0, 138.1, 130.5, 129.6, 129.3, 129.1, 121.6, 21.7. **HRMS(ESI)**: m/z calcd for $C_{11}H_{11}ClNaO_2S$ [M+Na]$^+$ 265,0066, found 265.0078.

For minor *E*-isomer selected signals: **^{1}H NMR** (400 MHz, $CDCl_3$) δ: 7.37 (d, *J* = 15.2 Hz, 1H), 6.31 (d, *J* = 15.2 Hz, 1H), 5.51 (1H), 5.39 (1H) 1.86 (3H).

(1*Z*)-3-Methyl-1-(4-methylphenylsulfonyl)buta-1,3-diene (3c). Obtained from **2c** (1.06 g, 4.8 mmol or 32.0 mg, 0.144 mmol). Yield 1.01 g or 31.1 mg (94% or 97%). Colorless oil. **^{1}H NMR** (400 MHz, $CDCl_3$) δ: 7.81 (d, *J* = 8.2 Hz, 2H), 7.34 (d, *J* = 8.2 Hz, 2H), 6.52 (d, *J* = 11.9 Hz, 1H), 6.34 (d, *J* = 11.9 Hz, 1H), 5.26 (m, 1H), 5.24 (m, 1H), 2.46 (s, 3H), 1.89 (s, 3H).**^{13}C NMR** (101 MHz, $CDCl_3$) δ: 144.2, 143.2, 138.7, 138.2, 131.0, 129.6, 127.8, 121.3, 21.8, 21.6. **HRMS(ESI)**: m/z calcd for $C_{12}H_{14}NaO_2S$ [M+Na]$^+$ 245.0612, found 245.0610.

(1*E*)-1-Bromo-3-methyl-1-(phenylsulfonyl)buta-1,3-diene (3d). Obtained from **2d** (41.0 mg, 0.144 mmol). Yield 36.4 mg (88%). Colorless oil. **^{1}H NMR** (400 MHz, acetone-*d*) δ: 7.93 – 7.87 (m, 3H), 7.66 – 7.61 (m, 1H), 7.66 – 7.62 (m, 2H), 5.63 (s, 1H), 5.53 (m, 1H), 2.01 (s, 3H). **^{13}C NMR** (101 MHz, $CDCl_3$) δ: 140.9, 138.6, 137.7, 134.1, 129.3, 128.8, 126.4, 120.3, 20.5. **IR (KBr),** cm^{-1}: 1312 (S=O), 1621(C=C), 1630 (C=C) **HRMS(ESI)**: m/z calcd for $C_{11}H_{11}BrNaO_2S$ [M+Na]$^+$ 308.9561, found 308.9554.

(1*Z*)-3-Methyl-1-(4-methylphenylsulfonyl)-1-phenylbuta-1,3-diene (3e). Obtained from **2e** (40.7 mg, 0.144 mmol). Yield 38.7 mg (95%). Solidifying colorless oil. Structure confirmed by single crystal X-ray analysis (see below). **^{1}H NMR** (400 MHz, $CDCl_3$) δ: 7.59 (d, *J* = 8.3 Hz, 2H), 7.42 – 7.29 (m, 2H), 6.70 (d, *J* = 0.7 Hz, 1H), 5.12 (s, 1H), 2.40 (s, 3H), 1.87 (d, *J* = 0.7 Hz, 3H).**^{13}C NMR** (101 MHz, $CDCl_3$) δ: 144.0, 143.7, 143.1, 139.5, 138.4, 135.7, 129.7, 129.1, 128.4, 128.2, 127.9, 116.1, 21.6, 20.5. **HRMS(ESI)**: m/z calcd for $C_{18}H_{18}NaO_2S$ [M+Na]$^+$ 321.0925, found 321.0928.

(1Z)-3-(*tert*-Butyl)-1-(4-methylphenylsulfonyl)-1-phenylbuta-1,3-diene (3f). Obtained from **2f** (47.0 mg, 0.144 mmol). Yield 41.2 mg (87%). Solidifying yellow oil.**^{1}H NMR** (400 MHz, $CDCl_3$) δ: 7.54 (d, *J* = 8.3 Hz, 2H), 7.39 – 7.24 (m, 7H), 6.83 – 6.81 (m, 1H), 5.32 (q, *J* = 0.6 Hz, 1H), 5.25 (s, 1H), 2.81 (s, 3H), 2.37 (s, 3H), 1.14 (s, 9H). **^{13}C NMR** (101 MHz, $CDCl_3$) δ: 156.3, 149.5, 149.1, 147.0, 143.4, 141.6, 134.9,

134.2, 133.7, 133.5, 133.1, 118.9, 40.7, 34.0, 25.7. **HRMS(ESI)**: m/z calcd for $C_{21}H_{24}NaO_2S$ [M+Na]$^+$ 363.1395, found 363.1399.

(1*Z*)-[2-(4-Methylphenylsulfonyl)ethenyl]cyclohexene (3g). Obtained from **2g** (35.0 mg, 0.144 mmol). Yield 31.8 mg (91%). Yellow oil.^{1}H NMR (400 MHz, $CDCl_3$) δ 7.78 (d, *J* = 8.3 Hz, 2H), 7.33 (d, *J* = 8.3 Hz, 2H), 6.40 (d, *J* = 11.9 Hz, 1H), 6.27 (d, *J* = 11.9 Hz, 1H), 6.13-6.6.10 (m, 1H), 2.46 (s, 3H), 2.14-2.10 (m, 2H), 2.07-2.04 (m, 2H), 1.56 – 1.46 (m, 4H). ^{13}C NMR (101 MHz, $CDCl_3$) δ 143.9, 143.9, 139.0, 136.1, 132.5, 129.4, 129.0, 127.7, 27.2, 25.8, 22.0, 21.5, 21.2.**HRMS(ESI)**: m/z calcd for $C_{12}H_{14}NaO_2S$ [M+Na]$^+$ 245.0612, found 245.0610.

(3*Z*,5*Z*)-2,7-Dimethyl-4,5-bis(4-methylphenylsulfonyl)octa-1,3,5,7-tetraene (3h). Obtained from **2h** (61.6 mg, 0.144 mmol). Yield 55.3 mg (89%). Yellow oil. **^{1}H NMR** (400 MHz, $CDCl_{3)}$ δ: 7.86 (d, J = 8.3 Hz, 4H), 7.27 (d, J = 8.0 Hz, 4H), 6.71 (s, 1H), 5.17 (m, 2H), 5.08 (m, 2H), 2.44 (s, 6H), 1.44 (s, 6H). **^{13}C NMR** (101 MHz, $CDCl_3$) δ: 147.4, 139.1, 136.1, 132.4, 128.3, 128.0, 122.4, 118.2, 20.6, 20.5. **HRMS(ESI)**: m/z calcd for $C_{24}H_{26}NaO_4S_2$ [M+Na]$^+$ 465.1170, found 465.1172.

General procedure for synthesis of compounds 4 from allenes 2a,b,d. 0.144 mmol of allene **2** was added to a solution of 2.16 mg (0.0144 mmol) TfOH in HFIP (5 ml) with stirring at room temperature. After 1 h, water (2 equiv) was added to reaction mixture. After 3 h of stirring, 30 ml of $CHCl_3$ were added, organic phase was washed with water, a saturated aqueous solution of $NaHCO_3$, water again, and dried with Na_2SO_4, the solvent was distilled off under reduced pressure to give pure products.

(***Z***)**-2-Methyl-4-(phenylsulfonyl)but-3-en-2-ol** (**4a**). Obtained from **2a** (30.0 mg, 0.144 mmol) and H_2O (5.2 mg, 0.288 mmol). Yield 32.4 mg (99%) Colorless oil. **^{1}H NMR** (400 MHz, $CDCl_3$) δ 8.05 – 8.00 (m, 1H), 7.70 – 7.64 (m, 2H), 7.62 – 7.55 (m, 2H), 6.38 (d, *J* = 12.3 Hz, 1H), 6.18 (d, *J* = 12.3 Hz, 1H), 1.51 (s, 6H);**^{13}C NMR** (101 MHz, $CDCl_3$) δ 153.3, 140.5, 133.7, 129.3, 128.5, 127.6, 70.8, 30.3; **IR(KBr)** , cm^{-1}: 1291(S=O), 1610(C=C), 3478 (O-H); **IR(KBr)** , cm^{-1}: 1291(S=O), 1610(C=C), 3478 (O-H); **HRMS(ESI)**: m/z calcd for $C_{11}H_{14}NaO_3S$ [M+Na]$^+$ 249.0555, found 249.0557.

(*Z*)-2-Methyl-4-(4-chlorophenylsulfonyl)but-3-en-2-ol (4b). Obtained from **2b** (38.1 mg, 0.144 mmol) and H_2O (5.2 mg, 0.288 mmol). Yield 29.4 mg (78%) Colorless oil. ^{1}H NMR (400 MHz, $CDCl_3$) δ 7.95 (d, *J* = 8.6 Hz, 2H), 7.53 (d, *J* = 8.6 Hz, 2H), 6.38 (d, *J* = 12.3 Hz, 1H), 6.15 (d, *J* = 12.3 Hz, 1H), 4.72 (1H), 1.47 (6H); ^{13}C NMR (101 MHz, $CDCl_3$) δ 154.0, 152.1, 129.6, 129.2, 128.2, 126.1, 71.0, 30.2; **HRMS(ESI)**: m/z calcd for $C_{11}H_{13}ClNaO_3S$ 283.0172, found 283.0180

(*E*)-4-Bromo-2-methyl-4-(phenylsulfonyl)but-3-en-2-ol (**4c**). Obtained from **2d** (41.2 mg, 0.144 mmol) and H_2O (5.2 mg, 0.288 mmol). Yield 43.0 mg (98%) Colorless oil. **^{1}H NMR** (400 MHz, acetone-*d*) δ 7.96 – 7.88 (m, 2H), 7.75 – 7.67 (m, 1H), 7.70 – 7.66 (m, 2H), 7.24 (s, 1H), 1.53 (s, 6H); **^{13}C NMR** (101 MHz, acetone-*d*) δ 146.6, 140.9, 134.2, 129.2, 128.7, 117.2, 46.8, 21.2; **IR(KBr)** , cm^{-1}: 1297(S=O), 1614(C=C), 3465 (O-H); **HRMS(ESI)**: m/z calcd for $C_{11}H_{13}BrNaO_3S$ [M+Na]$^+$ 326.9666, found 326.9670.

General procedure for synthesis of thiochromene-1,1-dioxides 5a-c from allenes 2a,c,d. 1 mmol of allene **2** was added to a solution of 1 equiv. of TfOH (151 mg) in 10 ml of anhydrous *o*-dichlorobenzene and

stirred under reflux during 2 h. After cooling, reaction mixture was filtered from insolubilities through celite pad and solvent was evaporated under reduced pressure to give pure compound.

4,4-Dimethyl-4*H*-thiochromene-1,1-dioxide (5a). Obtained from **2a** (208 mg, 1 mmol). Yield 165 mg (80%). Colorless oil. **^{1}H NMR** (400 MHz, $CDCl_3$) δ 8.06 – 8.02 (m, 1H), 7.63 – 7.57 (m, 1H), 7.53 – 7.49 (m, 2H), 6.67 (d, *J* = 11.4 Hz, 1H), 6.52 (d, *J* = 11.4 Hz, 1H), 1.58 (s, 6H);**^{13}C NMR** (101 MHz, $CDCl_3$) δ 147.8, 132.3, 127.9, 126.7, 125.3, 123.3, 36.3, 30.5; **IR (KBr)**, cm^{-1}: 1283 (S=O), 1699 (C=C); **HRMS(ESI)**: m/z calcd for $C_{11}H_{12}NaO_2S$ $[M+Na]^+$ 231.0450, found 231.0455.

4,4,7-Trimethyl-4*H*-thiochromene-1,1-dioxide (5b). Obtained from **2c** (222 mg, 1 mmol). Yield 189 mg (85%). **^{1}H NMR** (400 MHz, $CDCl_3$) δ: 7.84 (s, 1H), 7.399 (s, 1H), 7.395 (s, 1H), 6.65 (d, *J* = 11.4 Hz, 1H), 6.50 (d, *J* = 11.4 Hz, 3H), 2.44 (s, 3H), 1.55 (s, 6H). **^{13}C NMR** (101 MHz, $CDCl_3$) δ 147.8, 138.1, 137.3, 135.8, 135.8, 133.3, 126.6, 125.2, 123.1, 36.7, 30.5, 20.9. **HRMS(ESI)**: m/z calcd for $C_{12}H_{14}NaO_2S$ $[M+Na]^+$ 245.0612, found 245.0618.

2-Bromo-4,4-dimethyl-4*H*-thiochromene-1,1-dioxide (5c). Obtained from **2d** (287 mg, 1 mmol). Yield 261 mg (91%). Structure confirmed by single crystal X-ray analysis (see SI). **^{1}H NMR (400 MHz, $CDCl_3$)** δ: 8.08 – 8.04 (m, 1H), 7.65 – 7.60 (m, 1H), 7.56 – 7.49 (m, 2H), 6.79 (s, 1H), 1.60 (s, 6H);**^{13}C NMR (101 MHz, $CDCl_3$)** δ: 148.0, 142.65, 132.7, 128.2, 126.7, 124.2, 117.0, 40.1, 30.5; **IR (KBr) cm^{-1}**: 1695 (C=C), 1289 (S=O); **HRMS(ESI)**: m/z calcd for $C_{11}H_{11}BrNaO_2S$ $[M+Na]^+$ 308.9555, found 308.9556.

General procedure for synthesis of propan-2-ones 6 form allenes 2i,j. A mixture of allene **2** (0.144 mmol) and 21.6 mg (0.144 mmol) of TfOH in $CHCl_3$ (10 mL) was stirred at 130 °C temperature for 30 min in high pressure glass tube. After cooling, reaction mixture was diluted with 30 ml of $CHCl_3$, filtered through celite pad, washed with saturated water solution of NaCl, a saturated aqueous solution of $NaHCO_3$, water , and dried with Na_2SO_4. The solvent was distilled off under reduced pressure (without warming) to give pure product.

1-(Phenylsulfonyl)propan-2-one (6a). Obtained from **2i** (25.9 mg, 0.144 mmol).Yield 24.8 mg (87%). Colorless oil. Spectral characteristics correspond to the literature date.[26]

1-(4-Methylphenylsulfonyl)propan-2-one (6b). Obtained from **2j** (27.9 mg, 0.144 mmol).Yield 29.0 mg (95%). Colorless oil. Spectral characteristics correspond to the literature date.[26]

Procedure for synthesis of compounds 7a,b from allenes 1a,b. 1 mmol of allene **1a,b** was added to a solution 0.15 g (1 mmol) TfOH in CH_2Cl_2 (5 ml) at room temperature with stirring. After 5 min, 2 mmol (0.035 g, 2 eq.) of H_2O were added to reaction mixture. After 5 min of stirring, 30 ml of $CHCl_3$ were added, organic phase was washed with water, a saturated aqueous solution of $NaHCO_3$, water again, and dried with Na_2SO_4, the solvent was distilled off under reduced pressure to give pure adducts.

(Z)-2-Methyl-4-(phenylsulfinyl)but-3-en-2-ol (7a). Obtained from **1a** (192 mg, 1 mmol). Yield 201 mg (95%). Colorless oil. Spectral characteristics correspond to the literature date.[27]

(*Z*)-4-(4-Chlorophenylsulfinyl)-2-methylbut-3-en-2-ol (7b).Obtained from **1b** (226 mg, 1 mmol). Yield 205 mg (90%). Solidifying colorless oil. Structure confirmed by single crystal X-ray analysis (see SI). ^{1}H NMR (400 MHz, $CDCl_3$) δ: 7.72 (d, *J* = 8.5 Hz, 2H), 7.48 (d, *J* = 8.5 Hz, 2H), 6.18 (d, *J* = 10.8 Hz, 1H),

5.96 (d, J = 10.8 Hz, 1H), 3.24 (s, 1H), 1.52 (s, 3H), 1.40 (s, 3H). ^{13}C NMR (101 MHz, $CDCl_3$) δ: 146.6, 137.1, 134.4, 129.5, 126.4, 122.4, 72.2, 31.0, 30.2. **HRMS(ESI)**: m/z calcd for $C_{11}H_{13}ClNaO_2S$ $[M+Na]^+$ 267.0222, found 267.0222.

Characterization of ions A,B. These cations were generated at the protonation of allenes **1** and **2** in TfOH or D_2SO_4 directly in NMR tubes.

2-Phenyl-5,5-dimethyl-2,5-dihydro-1,2-oxathiol-2-ium (Aa). Obtained from **1a**. **^{1}H NMR** (400 MHz, TfOH) δ: 8.03 (d, J = 7.2 Hz, 3H), 7.86 (t, J = 7.2 Hz, 2H), 7.49 (d, J = 5.7 Hz, 1H), 6.85 (d, J = 5.7 Hz, 1H), 1.84 (s, 3H), 1.76 (s, 3H); **^{13}C NMR** (101 MHz, TfOH) δ: 150.2, 139.9, 132.8, 132.3, 131.1, 117.2, 112.4, 27.9, 27.7.

2-(4-Chlorophenyl)-5,5-dimethyl-2,5-dihydro-1,2-oxathiol-2-ium (Ab). Obtained from **1b**. **^{1}H NMR** (400 MHz, TfOH) δ: 7.96 (d, J = 8.7 Hz, 2H), 7.84 (d, J = 8.7 Hz, 2H), 7.51 (d, J = 6.1 Hz, 1H), 6.83 (d, J = 6.1 Hz, 1H), 1.84 (s, 3H), 1.76 (s, 3H); **^{13}C NMR** (101 MHz, TfOH) δ 150.6, 148.0, 133.7, 133.3, 129.4, 117.2, 112.8, 27.9, 27.8.

5,5-Dimethyl-2-oxo-2-phenyl-5*H*-1,2-oxathiol-2-ium (Ba). Obtained from **2a**. **^{1}H NMR** (400 MHz, TfOH) δ: 8.20 – 8.06 (m, 1H), 8.05 – 8.03 (m, 2H), 8.05 (d, J = 6.2 Hz, 1H, H_4), 7.98 – 7.89 (m, 2H), 7.16 (d, J = 6.2 Hz, 1H, H_3), 2.11 (s, Me), 2.09 (s, Me); **^{13}C NMR** (100 MHz, TfOH) δ, ppm: 158.7, 140.2, 132.1, 130.3, 128.6, 122.1, 112.0(C_5), 27.6(Me), 25.2(Me).

2-(4-Chlorophenyl)-5,5-dimethyl-2-oxo-5*H*-1,2-oxathiol-2-ium (Bb). Obtained from **2b**. **^{1}H NMR** (400 MHz, TfOH) δ: 8.07 (d, J = 6.0 Hz, 1H), 7.98 (d, J = 8.5 Hz, 2H), 7.90 (d, J = 8.5 Hz, 2H), 7.17 (d, J = 6.0 Hz, 1H), 2.10 (3H), 2.09 (3H); **^{13}C NMR** (101 MHz, TfOH) δ: 159.6, 149.0, 133.1, 132.2, 129.9, 127.2, 122.4, 113.0, 28.0, 25.7.

5,5-Dimethyl-2-(4-methylphenyl)-2-oxo-5*H*-1,2-oxathiol-2-ium (Bc). Obtained from **2c**. **^{1}H NMR** (400 MHz, TfOH) δ: 8.01 (d, J = 6.2 Hz, 1H), 7.92 (d, J = 8.5 Hz, 1H), 7.75 (d, J = 8.4 Hz, 1H), 7.12 (d, J = 6.2 Hz, 1H), 2.64 (s, 3H), 2.09 (s,3H), 2.08 (s, 3H); **^{13}C NMR** (101 MHz, TfOH) δ 157.1(C_4), 152.8, 131.8, 129.3, 123.8, 121.3(C_4), 110.2 (C_3), 26.5, 24.2, 20.7.

3-Bromo-5,5-dimethyl-2-oxo-2-phenyl-5*H*-1,2-oxathiol-2-ium (Bd). Obtained from **2d**. **^{1}H NMR** (400 MHz, TfOH) δ, ppm: 8.23 – 8.16 (m, 1H), 8.12 – 8.07 (m, 2H), 8.04 – 7.99 (m, 1H), 7.95 (s, 1H, H_4), 2.15 (s, Me), 2.15 (s, Me); **^{13}C NMR** (100 MHz, TfOH) δ, ppm: 153.0, 139.6, 131.0, 129.7, 123.7, 112.7, 109.9, 26.3(Me), 24.1(Me).

3-Bromo-4-deutero-5,5-dimethyl-2-oxo-2-phenyl-5*H*-1,2-oxathiol-2-ium (Bd-*d*). Obtained from **2d**. **^{1}H NMR** (400 MHz, D_2SO_4) δ 8.20 – 8.15 (m, 1H), 8.03 – 8.00 (m, 2H), 7.98 – 7.93 (m, 2H), 2.11 (s, Me), 2.11 (s, Me); **^{13}C NMR** (101 MHz, D_2SO_4) δ 153.0(t, C_4, J = 20 Hz), 139.9, 131.3, 129.8, 123.4, 113.0 (C_5), 109.0(C_3), 26.7(Me), 24.5(Me).

5,5-Dimethyl-2-(4-methylphenyl)-2-oxo-5*H*-1,2-oxathiol-2-ium (Be). Obtained from **2e**. **^{1}H NMR** (400 MHz, TfOH) δ 7.97 (d, J = 8.0 Hz, 2H), 7.91 (s, 1H), 7.71 (d, J = 8.0 Hz, 2H), 7.58 (d, J = 7.3 Hz, 3H), 7.54 – 7.47 (m, 2H), 2.60 (s, 3H), 2.19 (s, 3H), 2.18 (s, 3H); **^{13}C NMR** (101 MHz, TfOH) δ 154.41(C_4), 148.65, 137.19, 133.79, 133.53, 131.18, 130.92, 128.64, 125.13, 123.47(C_3), 110.04(C_5), 28.62, 26.46, 22.27.

5-(*tert*-Butyl)-5-methyl-2-(4-methylphenyl)-2-oxo-3-phenyl-5*H*-1,2-oxathiol-2-ium (**Bf**). Obtained from **2f**. Mixture of isomers (5:1). Data for major isomer**:** ^{1}**H NMR** (400 MHz, TfOH) δ 8.02 (s, 1H), 7.99 – 7.90 (m, 2H), 7.70 (d, *J* = 8.0 Hz, 2H), 7.62 – 7.48 (m, 5H), 2.59 (s, Me), 2.17 (s, Me), 1.39 (s, 3Me); ^{13}**C NMR** (101 MHz, TfOH) δ 154.1(C_4), 148.2, 133.8, 133.5, 131.2, 130.7, 128.7, 128.5, 125.6(C_3) 119.8(C_5), 41.0, 26.4, 22.0, 20.7.

2-(4-Methylphenyl)-2-oxo-1-oxa-3-phenyl-2-thiaspiro[4.5]dec-3-en-2-ium (Bg). Obtained from **2g**. ^{1}**H NMR** (400 MHz, TfOH) δ 8.02 (d, *J* = 6.2 Hz, 1H), 7.92 (d, *J* = 8.2 Hz, 2H), 7.74 (d, *J* = 8.2 Hz, 2H), 7.11 (d, *J* = 6.2 Hz, 1H), 2.64 (s, 3H), 2.30 (s, 4H), 2.04 – 1.77 (m, 6H). ^{13}**C NMR** (101 MHz, TfOH) δ 157.7(C_4), 154.1, 133.2, 130.7, 125.5, 122.6(C_3), 114.7(C_5), 37.6, 35.3, 24.3, 23.1, 22.7, 22.1.

Dication (Bh). Obtained from **2h**. ^{1}**H NMR** (400 MHz, TfOH) δ 8.35 (s, 1H), 8.09 (s, 1H), 7.95 (d, *J* = 8.4 Hz, 2H), 7.87 (d, *J* = 8.3 Hz, 2H), 7.89 – 7.66 (m, 4H), 2.73 (s, 3H), 2.72 (s, 3H), 2.19 (s, Me), 2.16 (s, Me), 2.14 (s, Me), 2.06 (s, Me); ^{13}**C NMR** (101 MHz, TfOH) δ 157.9, 157.1, 155.9, 155.4, 132.9, 132.7, 130.1, 129.7, 120.9, 120.7, 112.8, 112.2, 26.5, 26.5, 24.2, 23.9, 21.0, 21.0.

II. Copies of NMR spectra

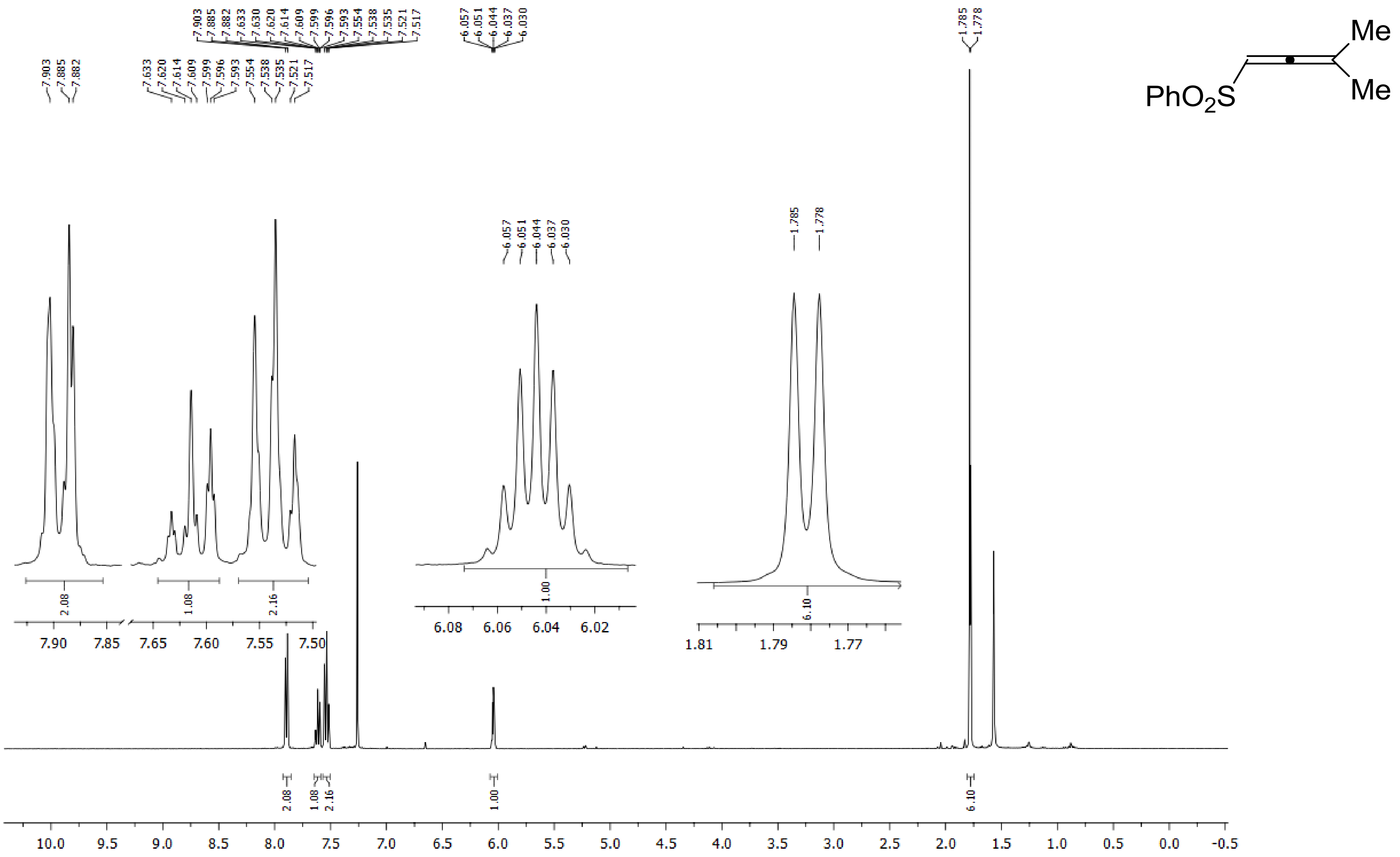

Fig. S1.^{1}H NMR spectrum of the compound **2a** (400 MHz, $CDCl_3$).

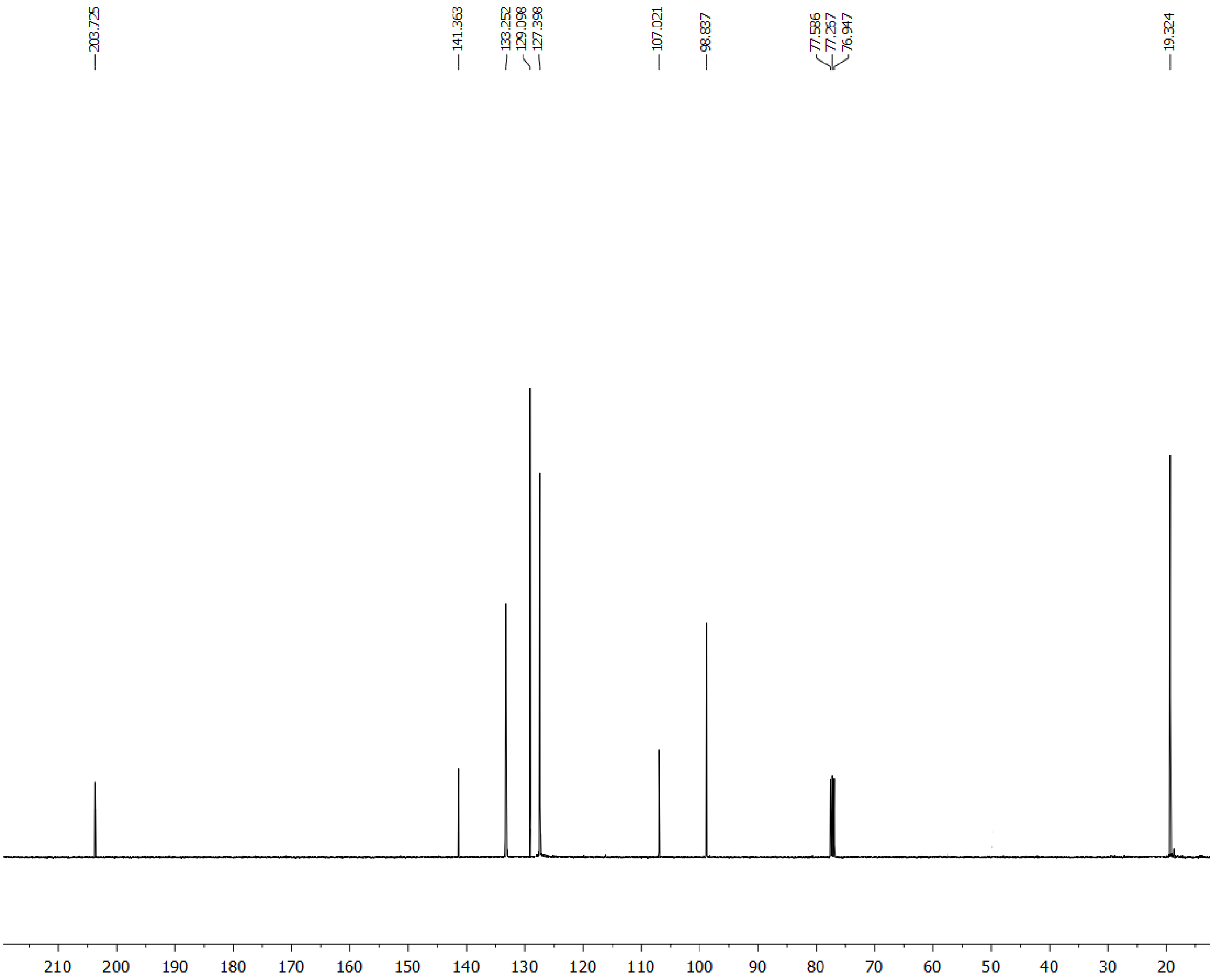

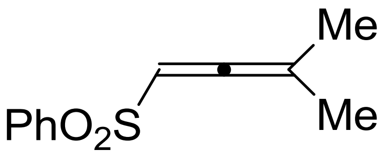

Fig. S2.^{13}C NMR spectrum of the compound **2a** (100 MHz, $CDCl_3$).

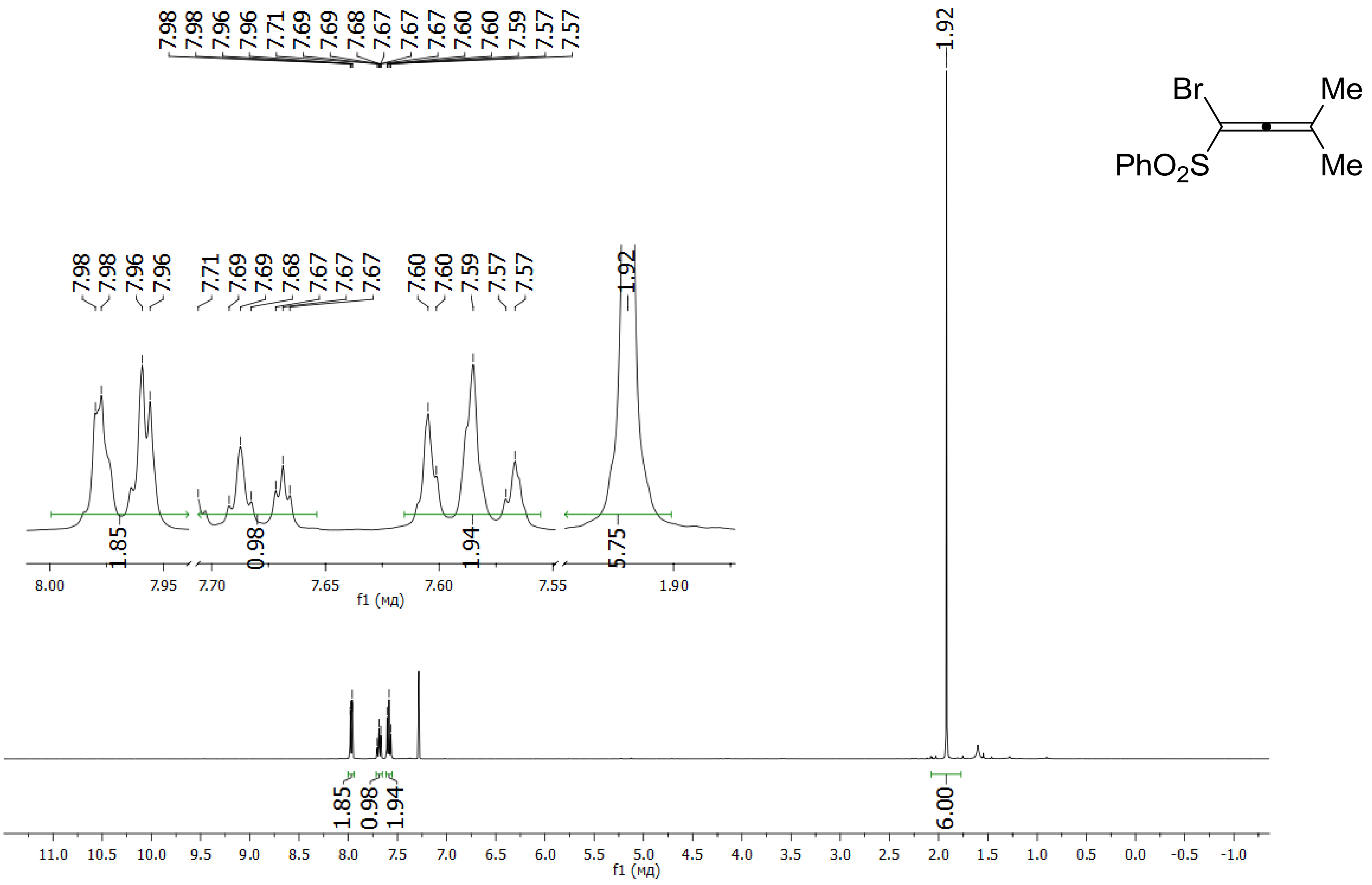

Fig. S3.^{1}H NMR spectrum of the compound **2d** (400 MHz, $CDCl_3$).

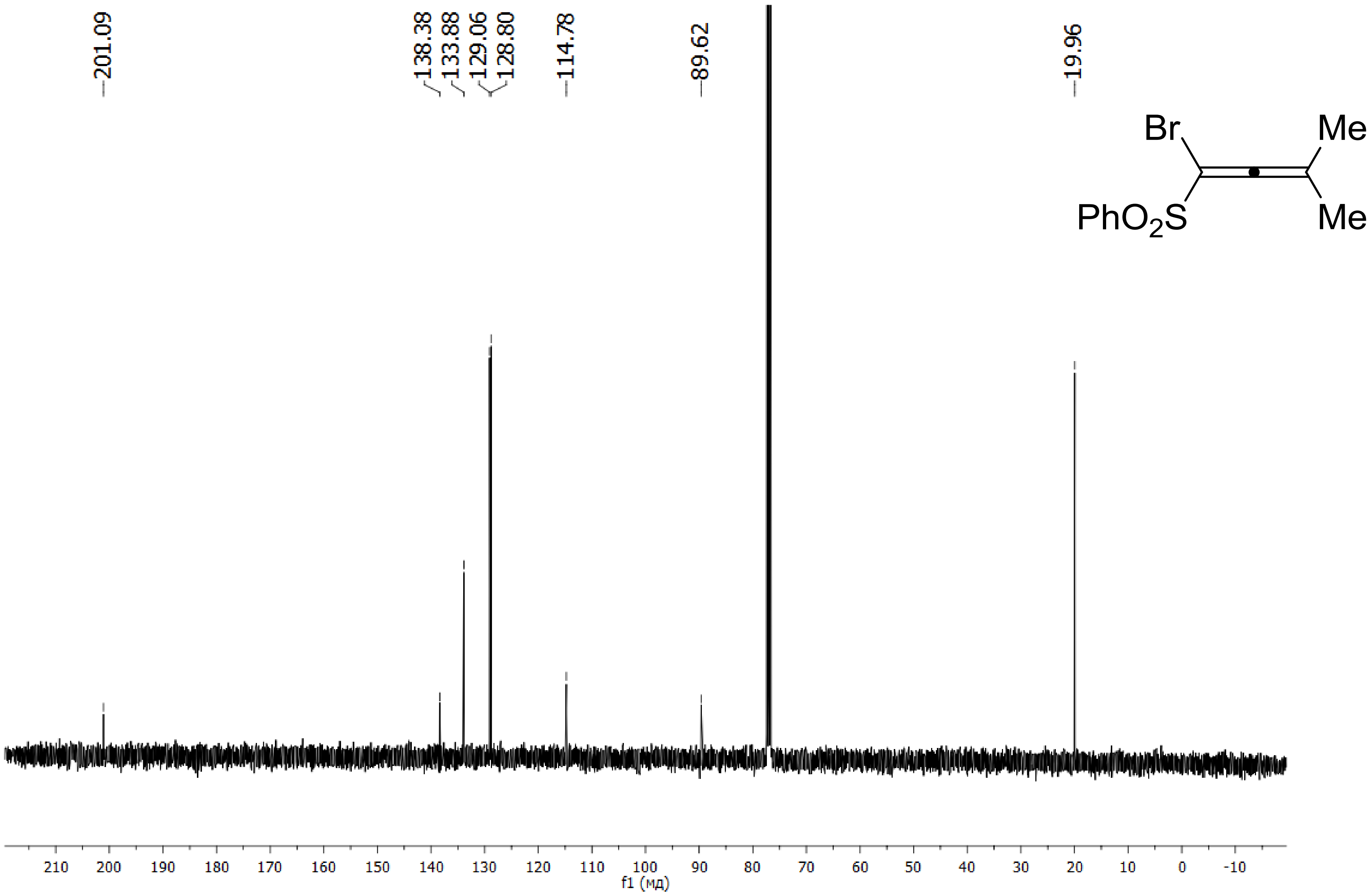

Fig. S4.^{13}C NMR spectrum of the compound **2d** (100 MHz, $CDCl_3$).

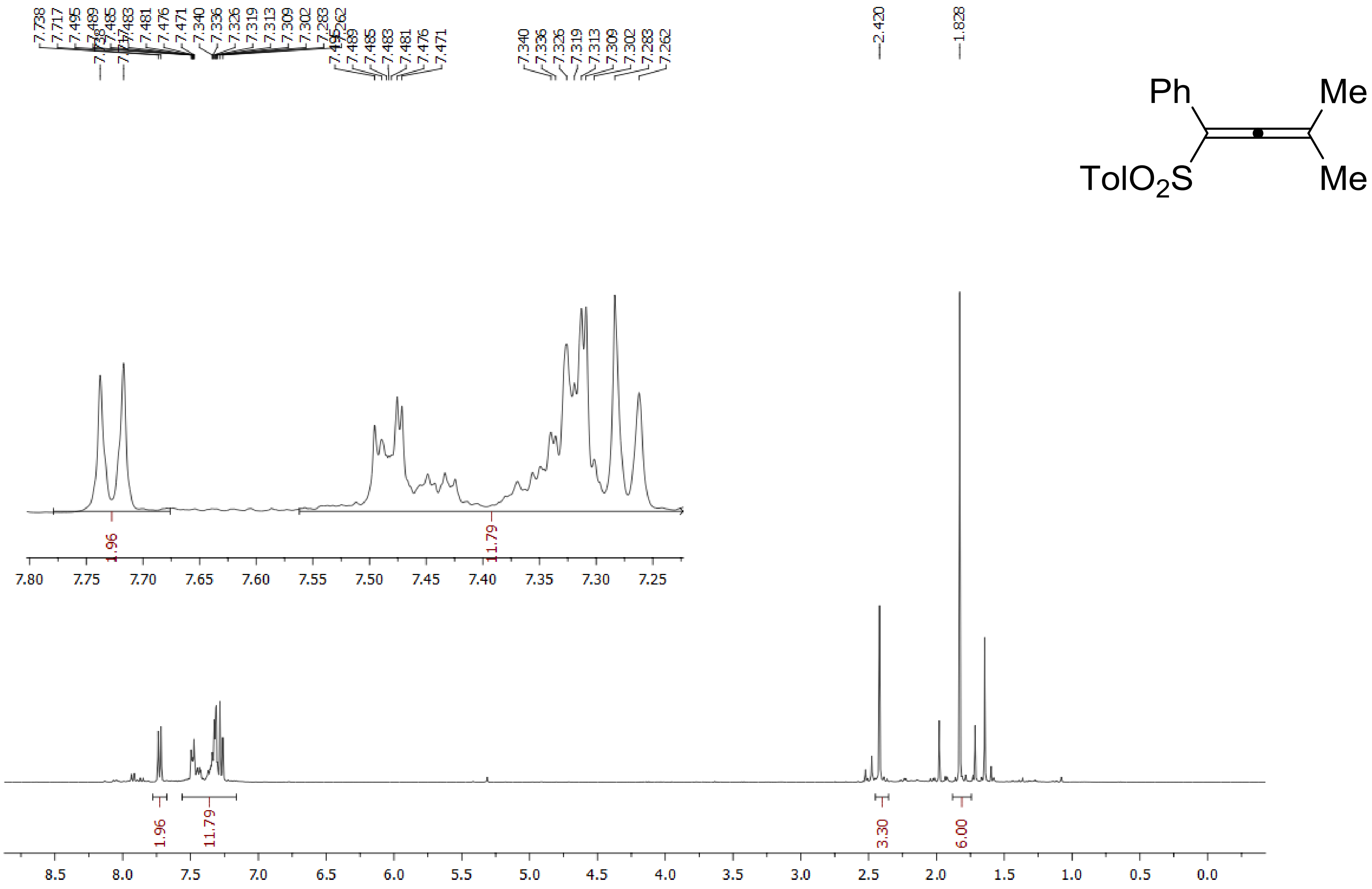

Fig. S5.^{1}H NMR spectrum of the compound **2e** (400 MHz, $CDCl_3$).

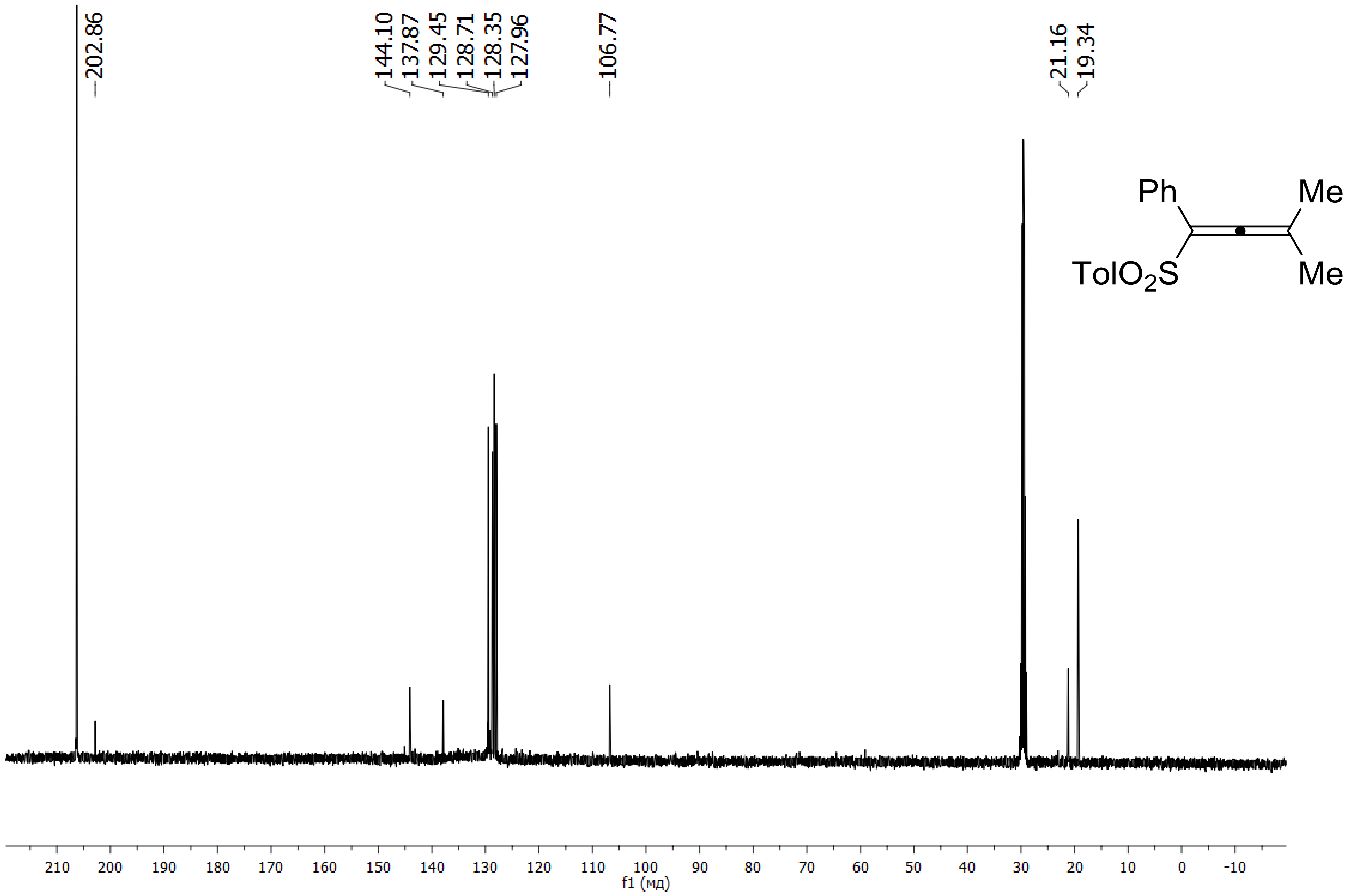

Fig. S6. ^{13}C NMR spectrum of the compound **2e** (100 MHz, acetone-d6).

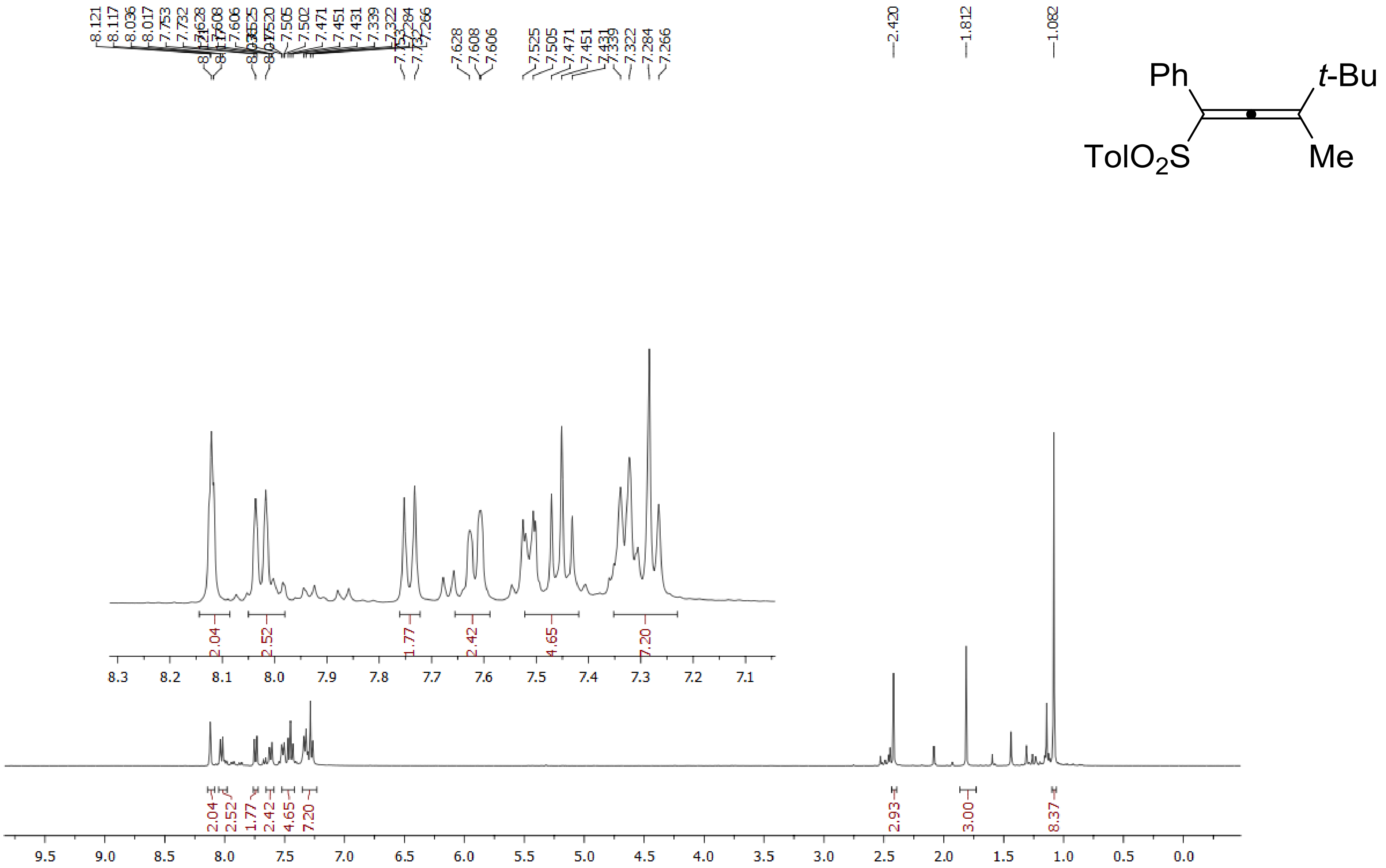

Fig. S7. ^{1}H NMR spectrum of the compound **2e** (400 MHz, $CDCl_3$).

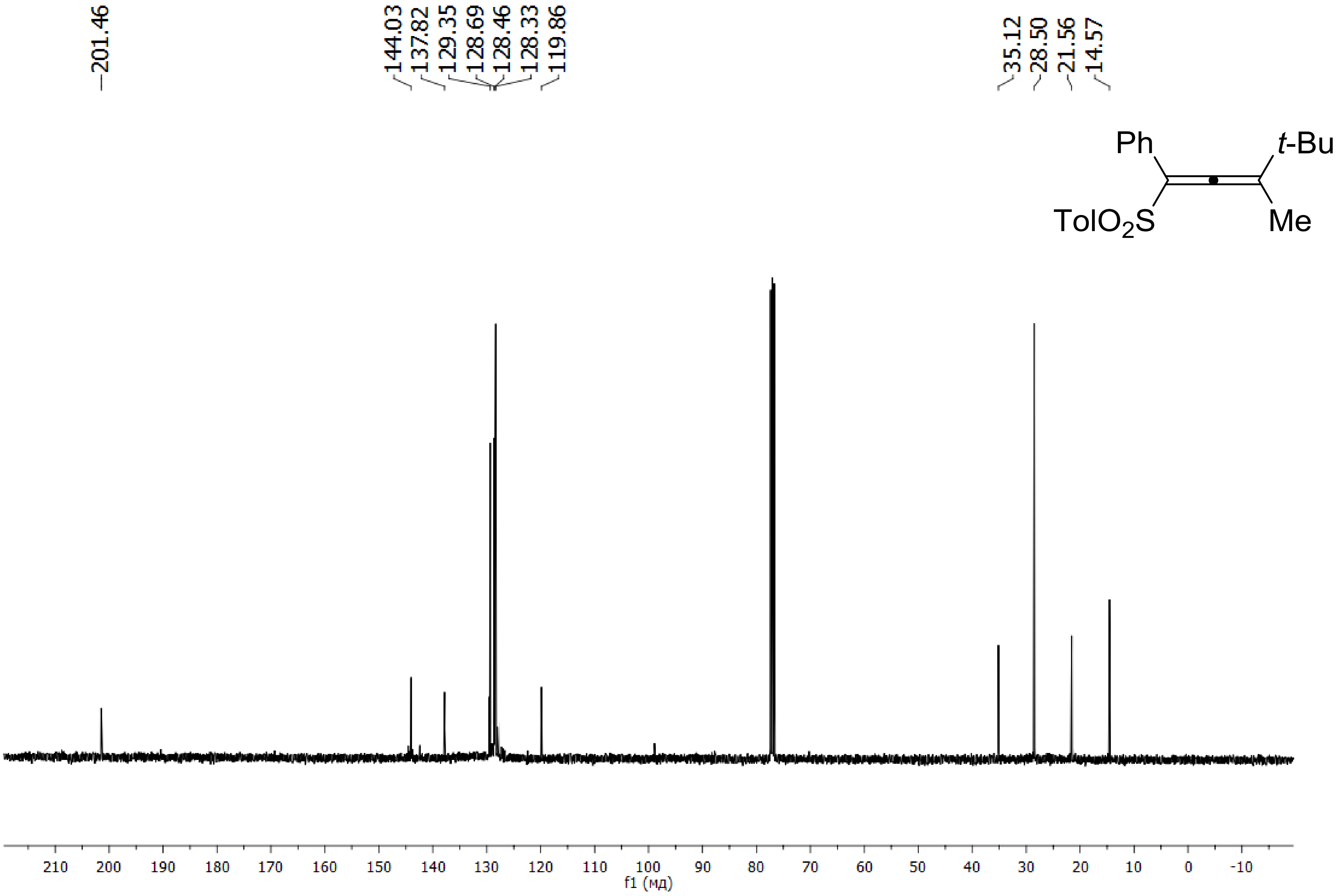

Fig. S8. ^{13}C NMR spectrum of the compound **2e** (100 MHz, acetone-d6).

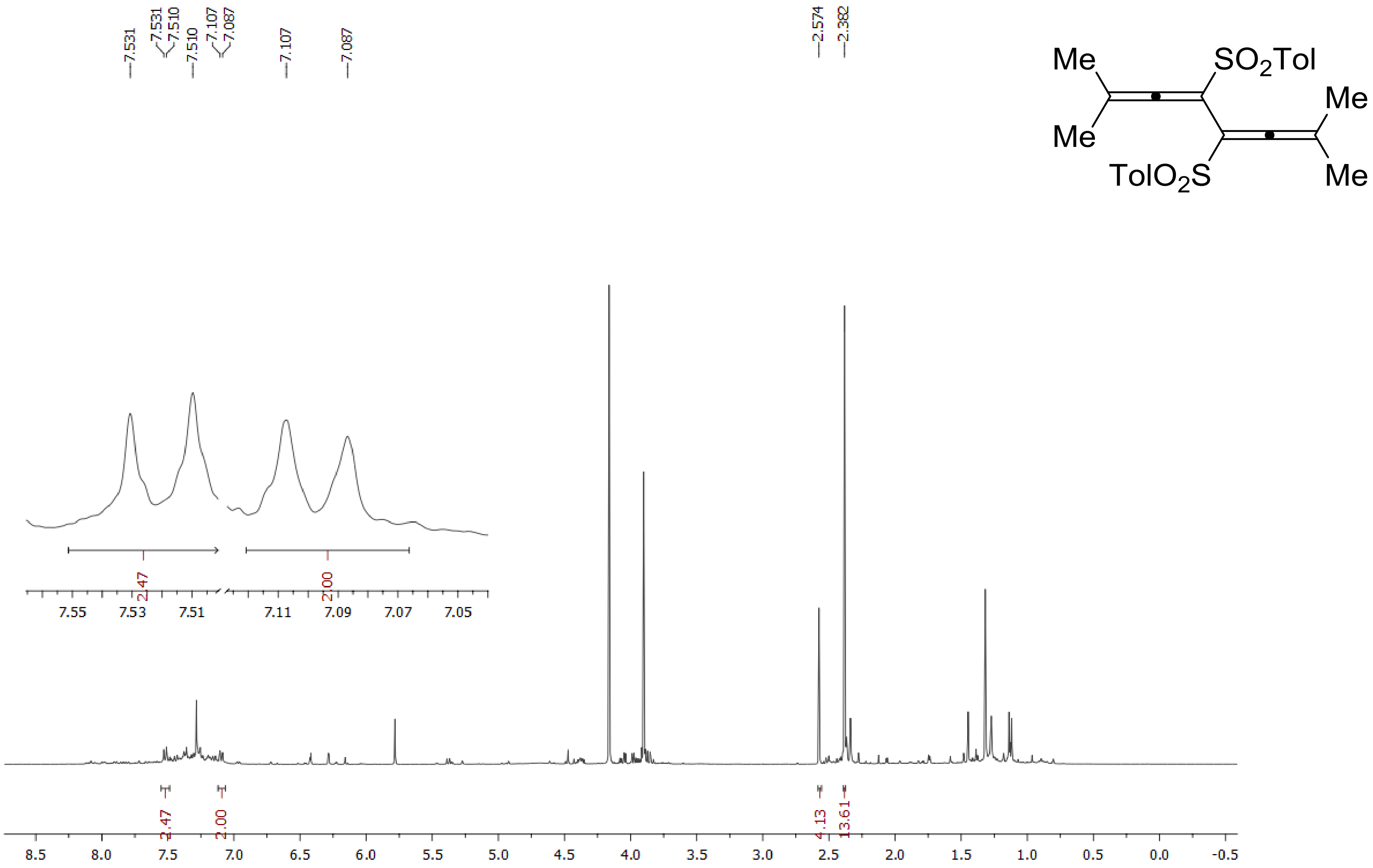

Fig. S9. ^{1}H NMR spectrum of the compound **2h** (400 MHz, $CDCl_3$).

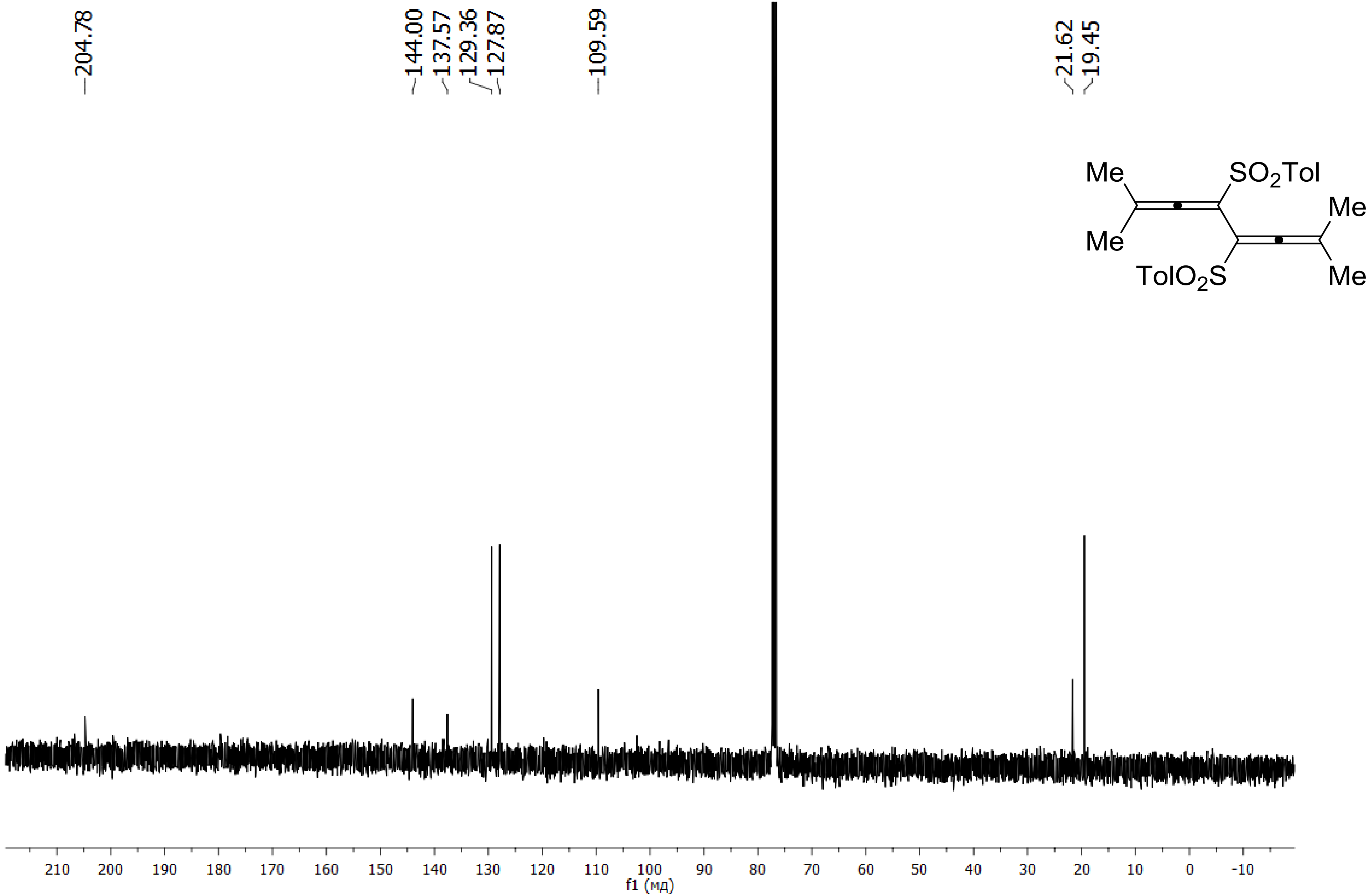

Fig. S10.^{13}C NMR spectrum of the compound **2h** (100 MHz, $CDCl_3$).

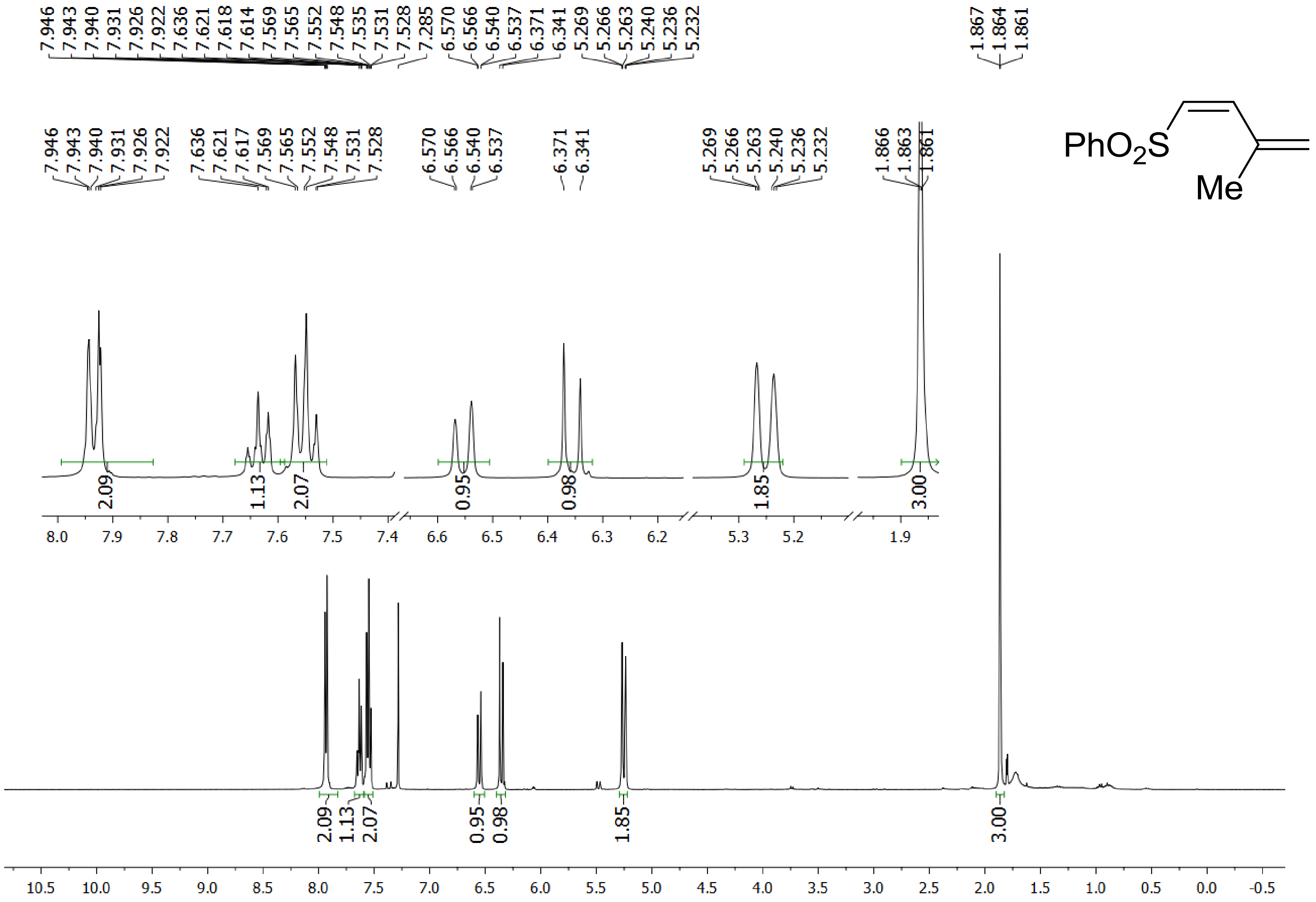

Fig. S11.^{1}H NMR spectrum of the compound **3a** (400 MHz, $CDCl_3$).

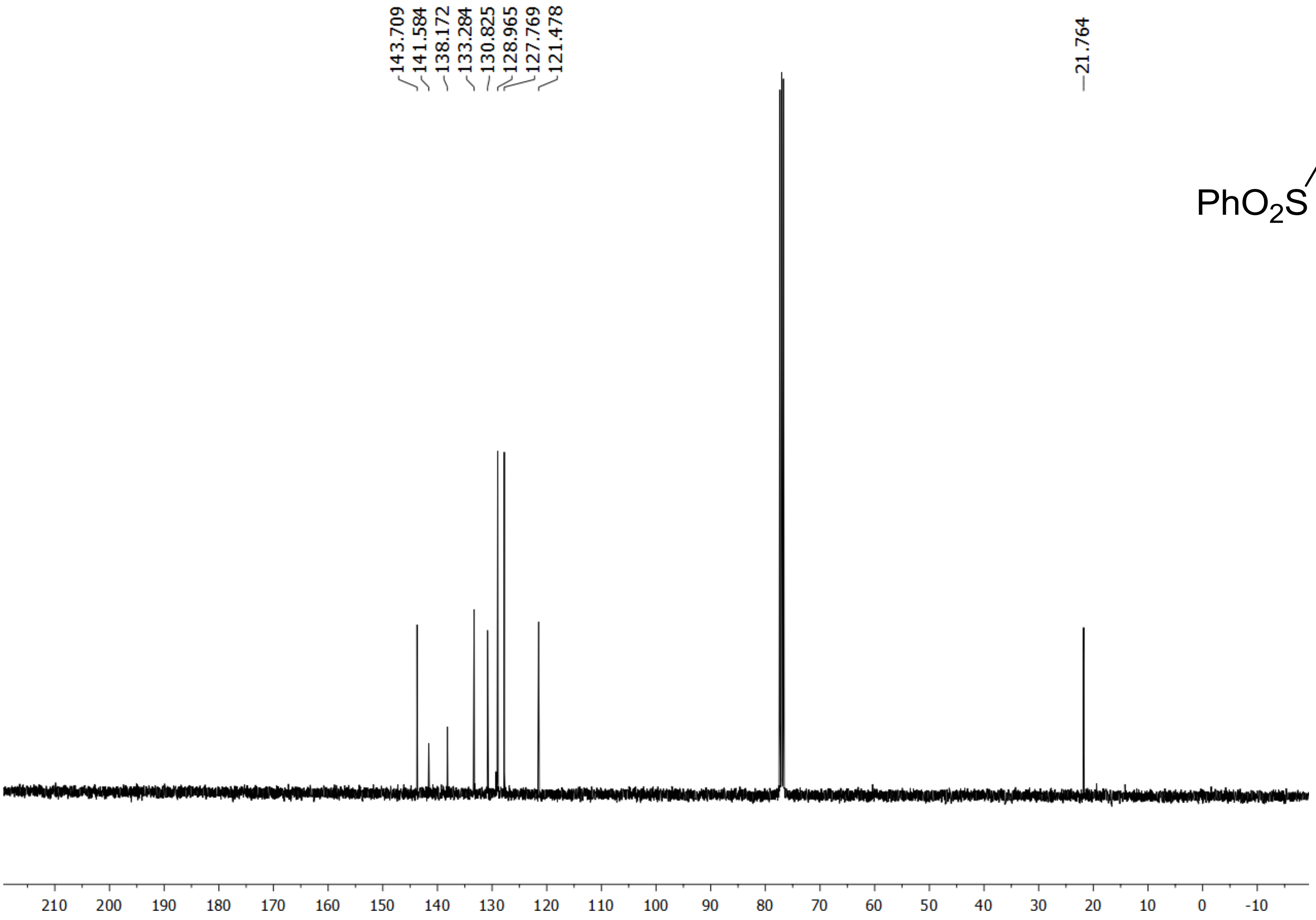

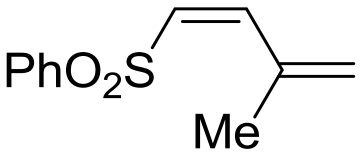

Fig. S12.^{13}C NMR spectrum of the compound **3a** (100 MHz, $CDCl_3$).

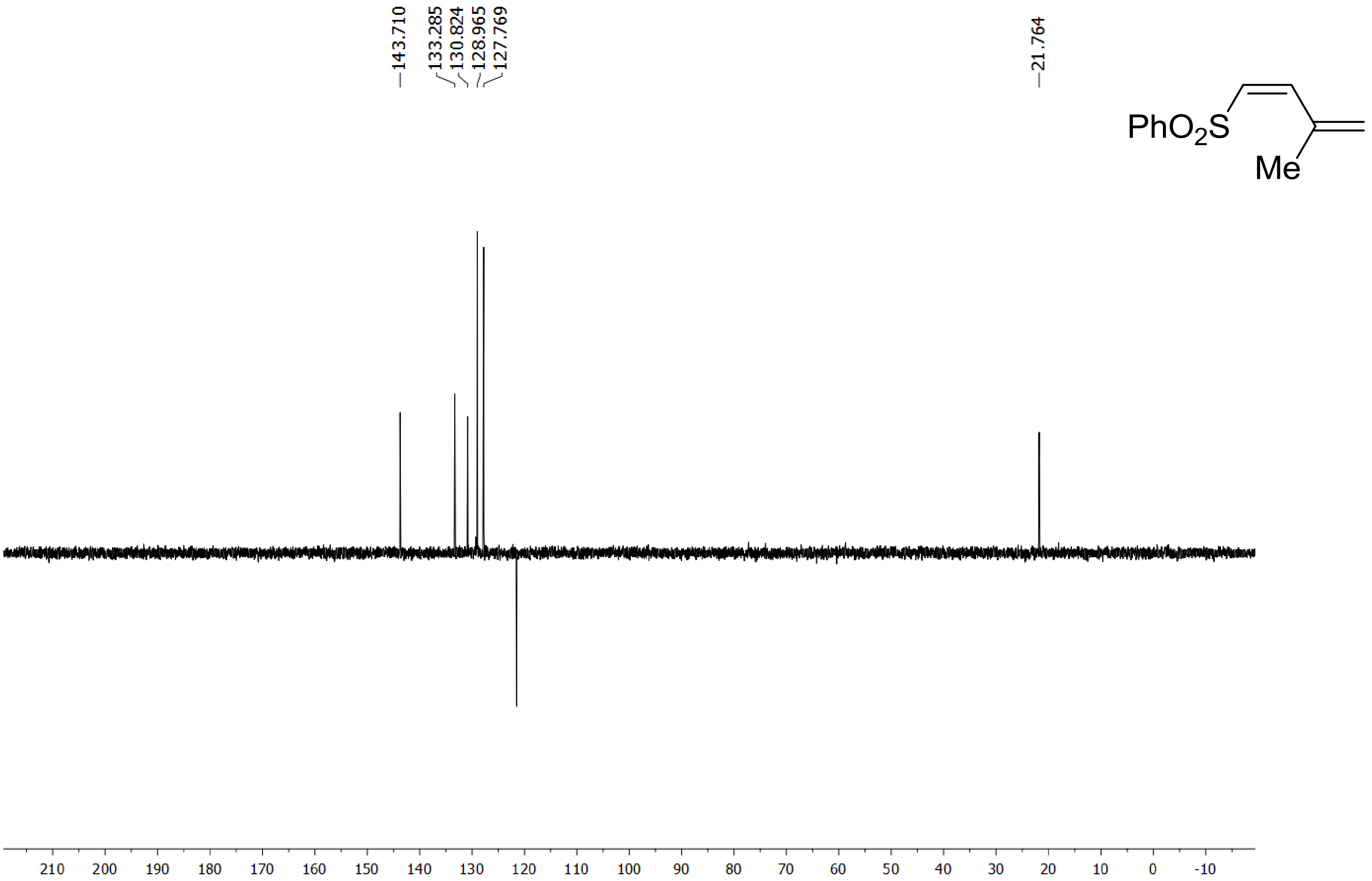

Fig. S13. DEPT NMR spectrum of the compound **3a** (100 MHz, $CDCl_3$).

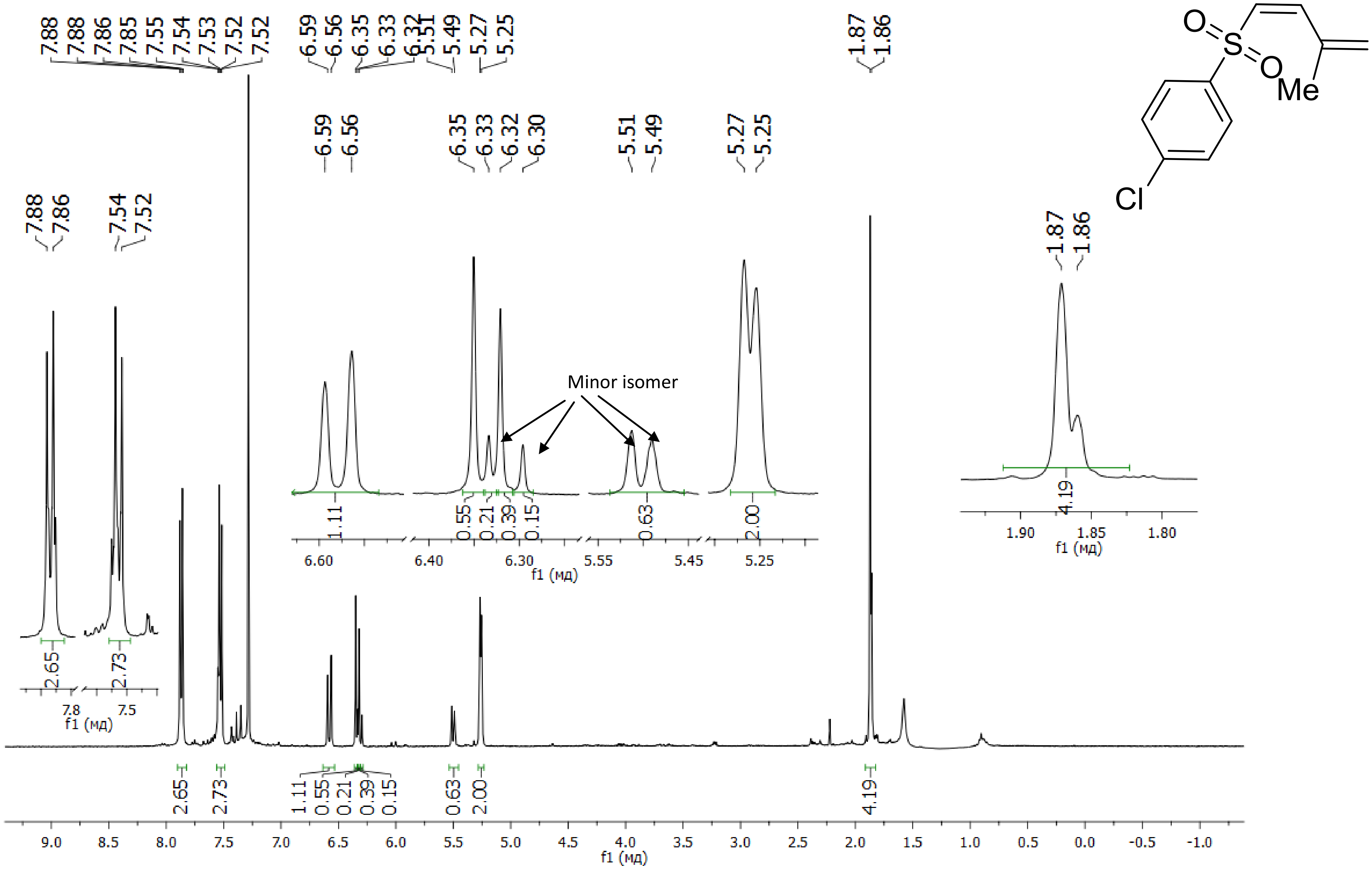

Fig. S14.^{1}H NMR spectrum of the compound **3b** (400 MHz,$CDCl_3$).

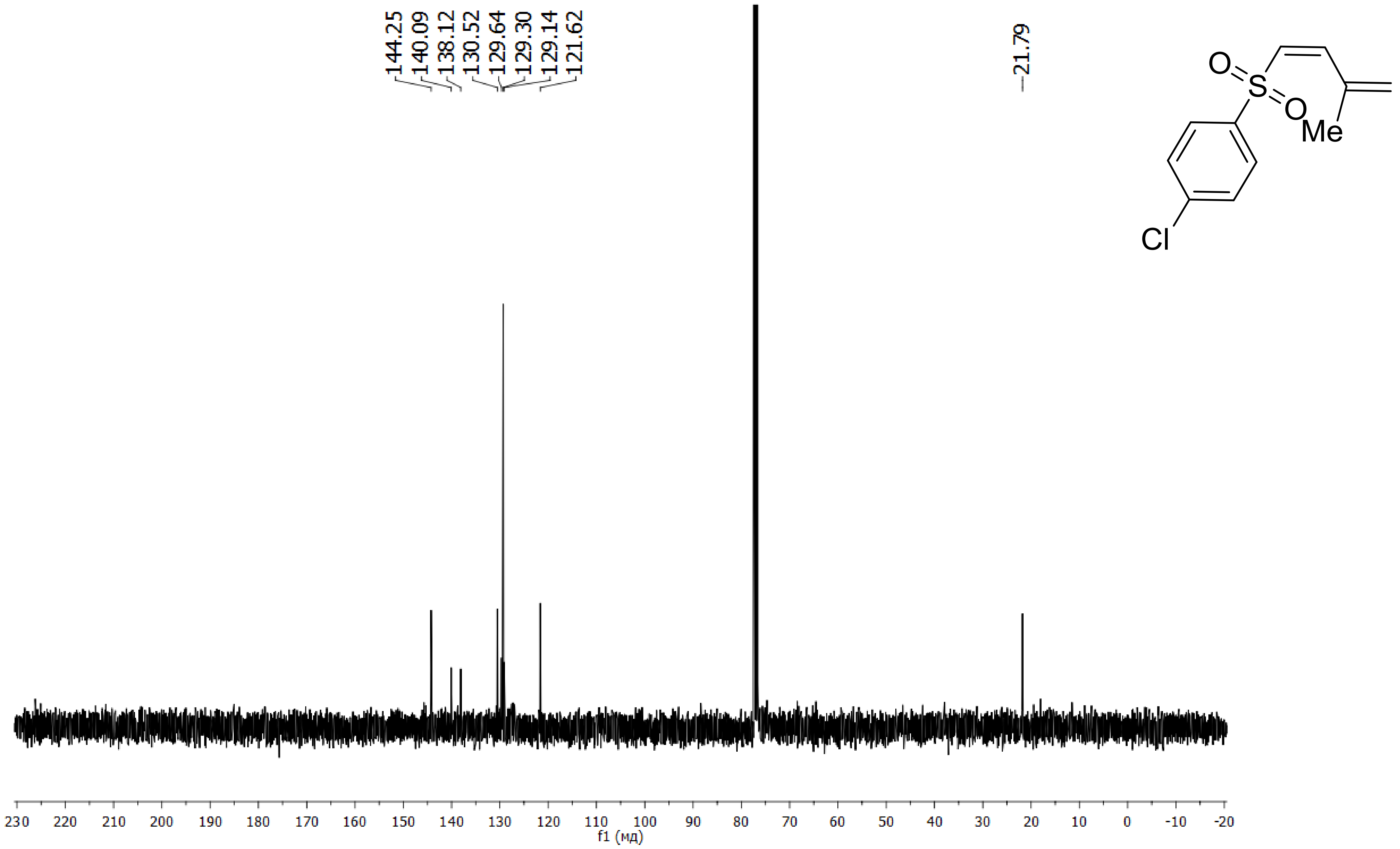

Fig. S15.^{13}C NMR spectrum of the compound **3b** (100 MHz,$CDCl_3$).

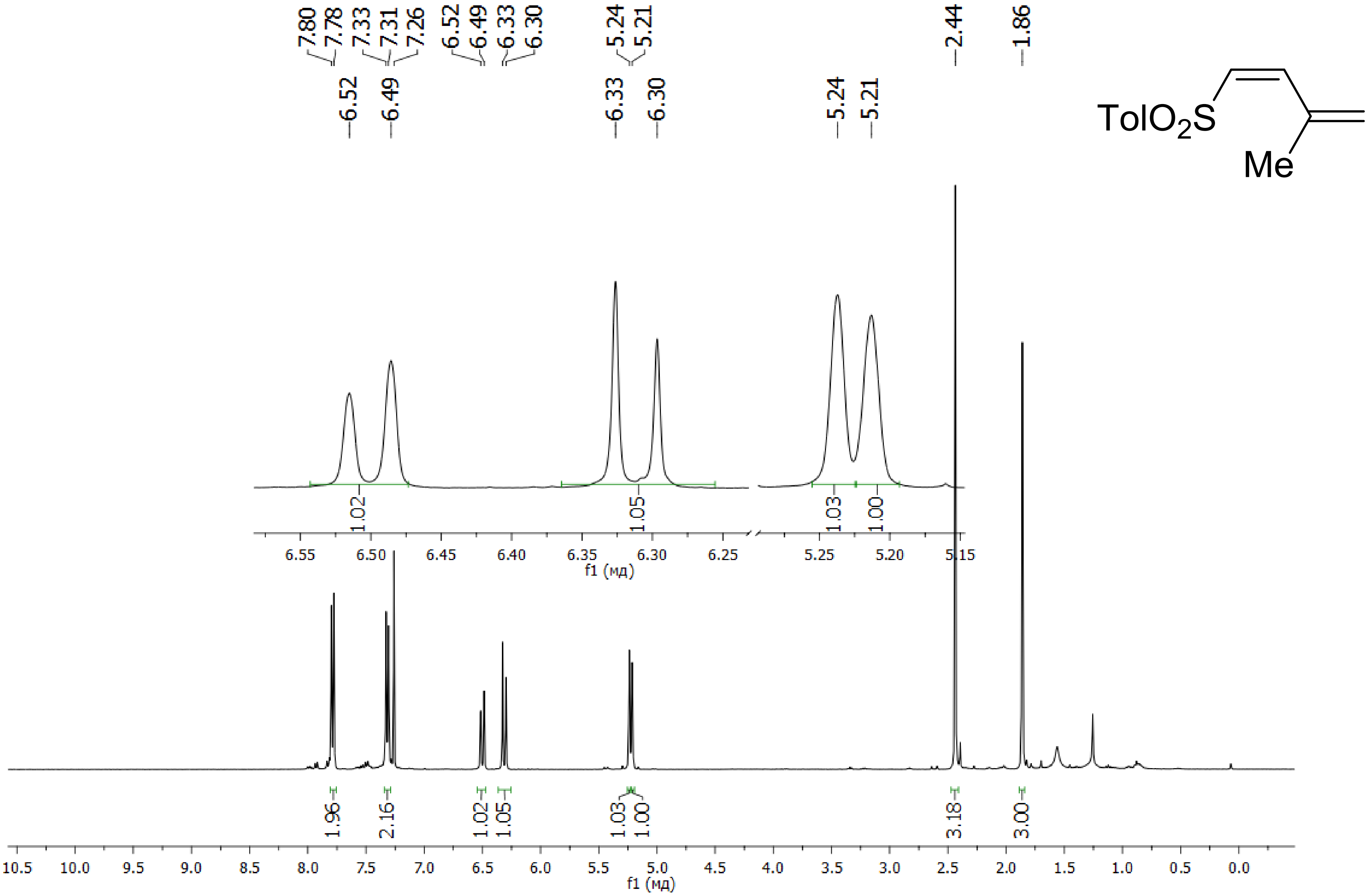

Fig. S16.^{1}H NMR spectrum of the compound **3c** (400 MHz, acetone-d6).

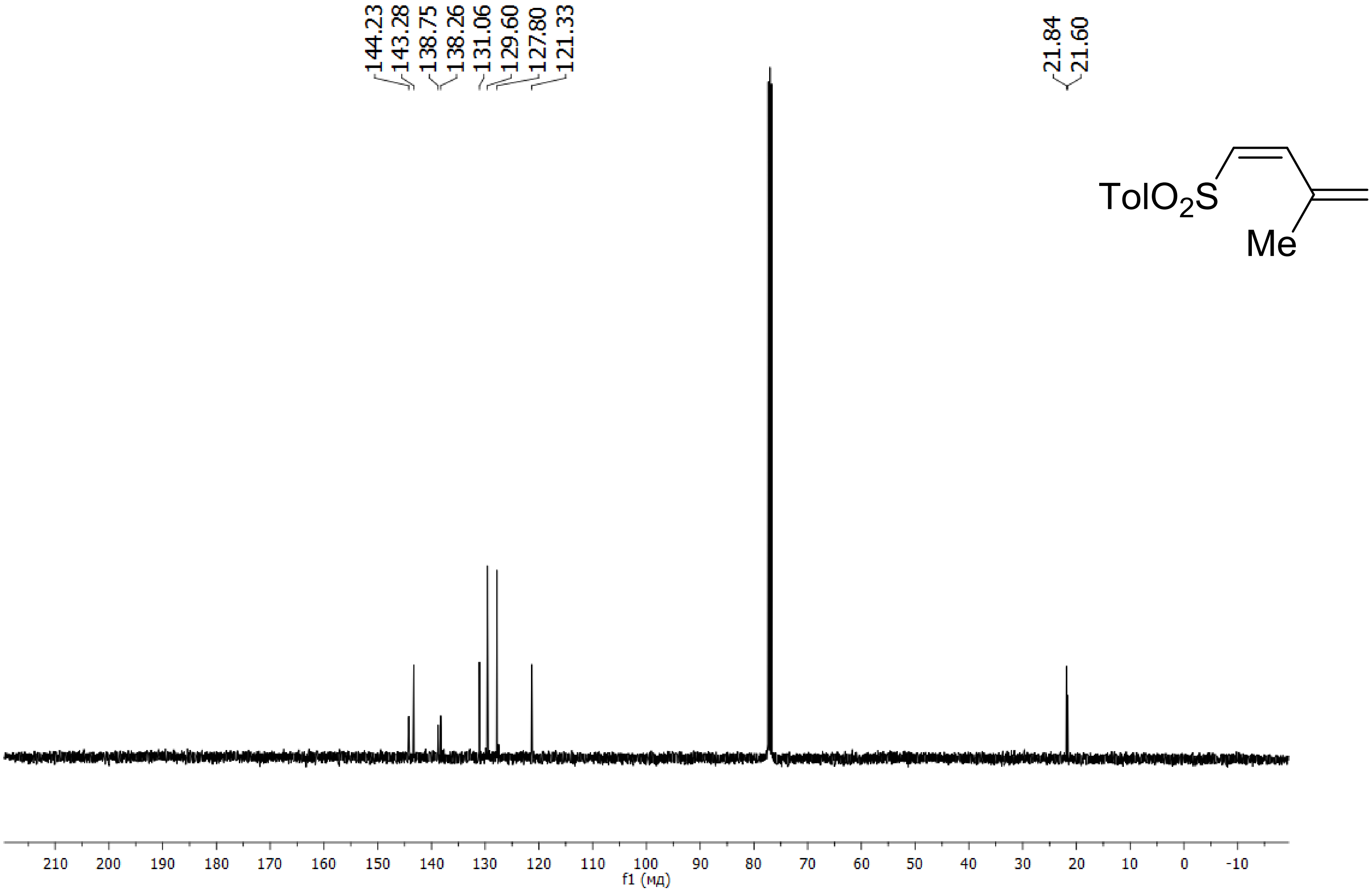

Fig. S17.^{13}C NMR spectrum of the compound **3c** (100 MHz, $CDCl_3$).

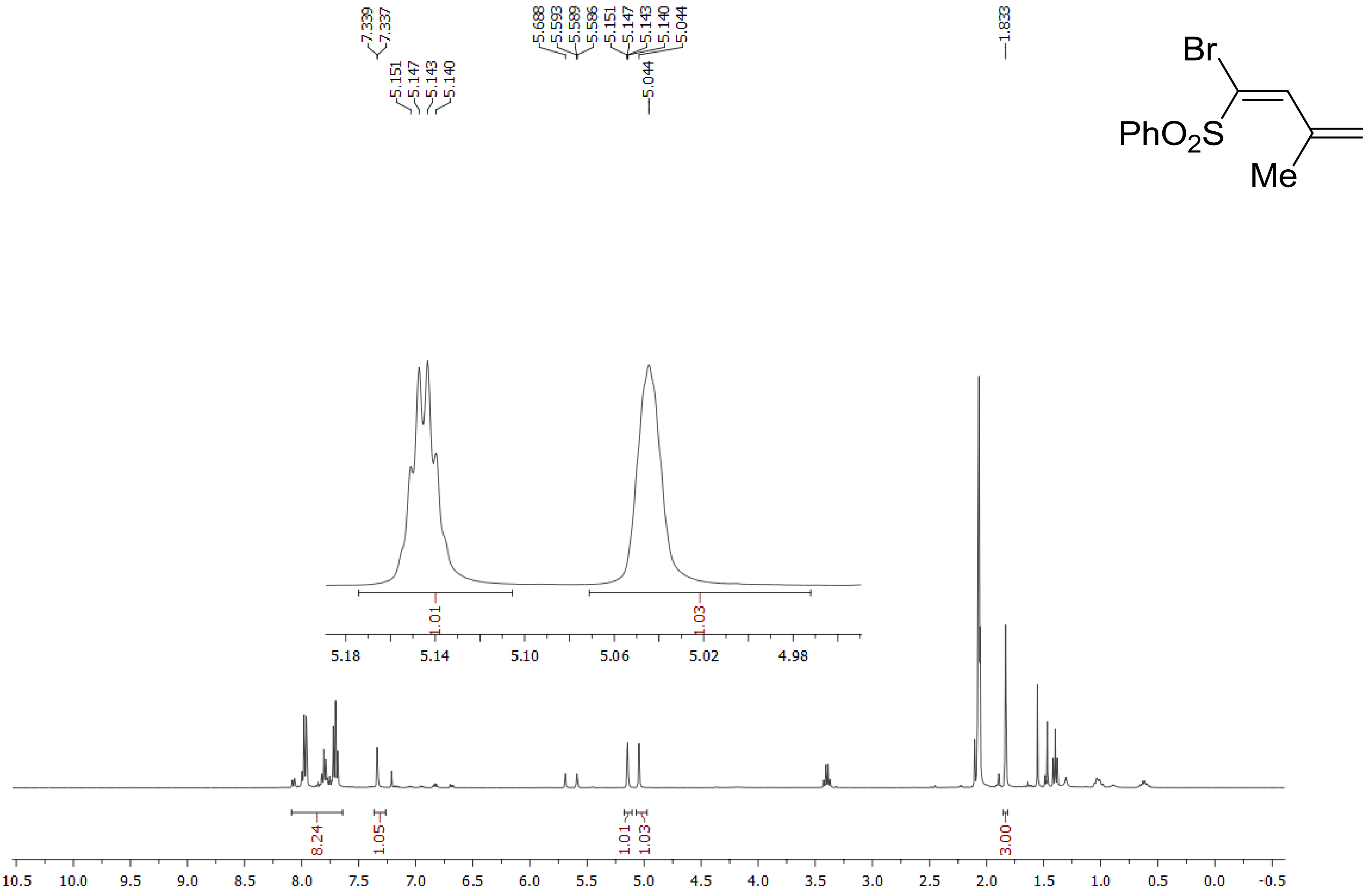

Fig. S18.^{1}H NMR spectrum of the compound **3d** (400 MHz, acetone-d6).

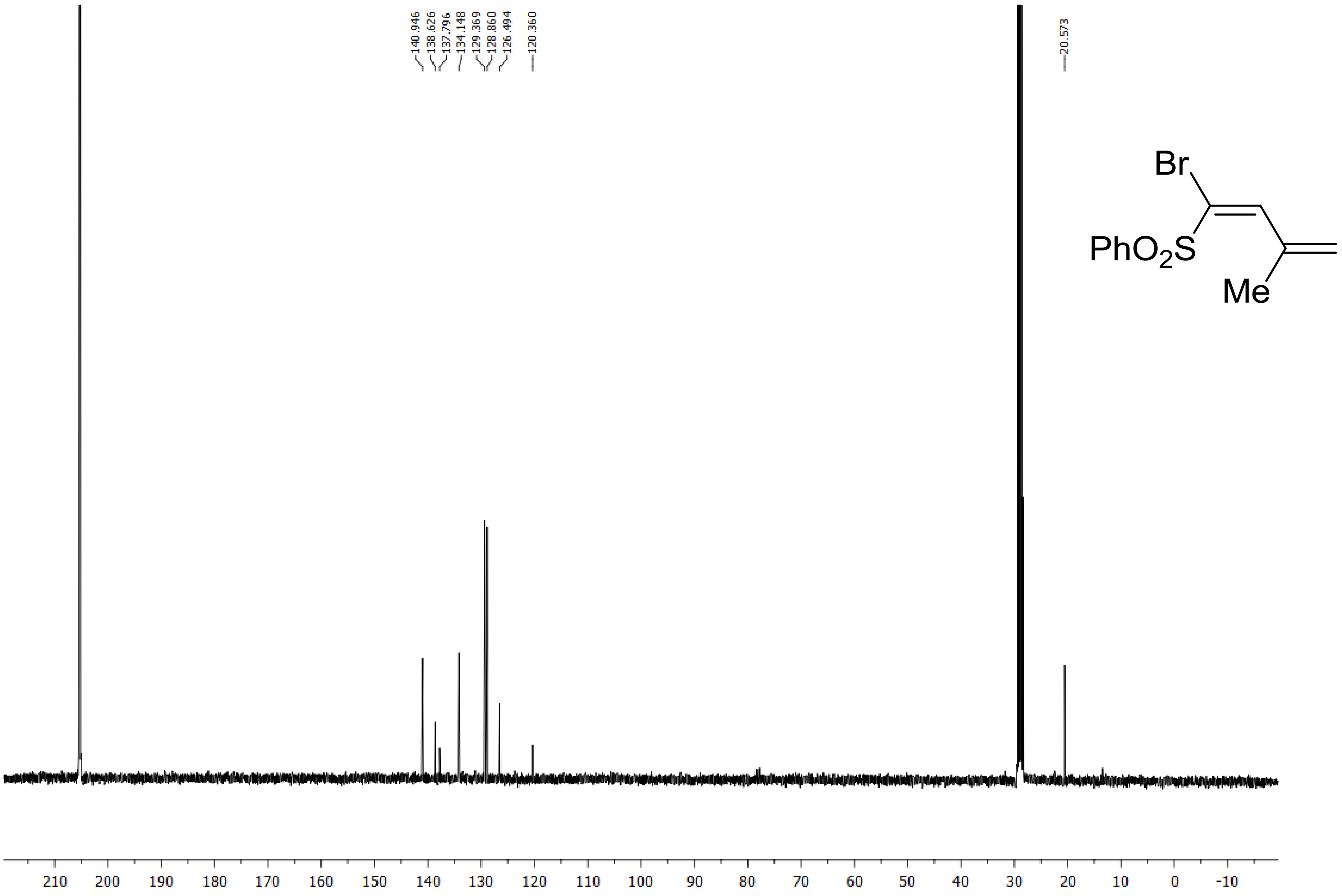

Fig. S19.^{13}C NMR spectrum of the compound **3d** (100 MHz, acetone-d6).

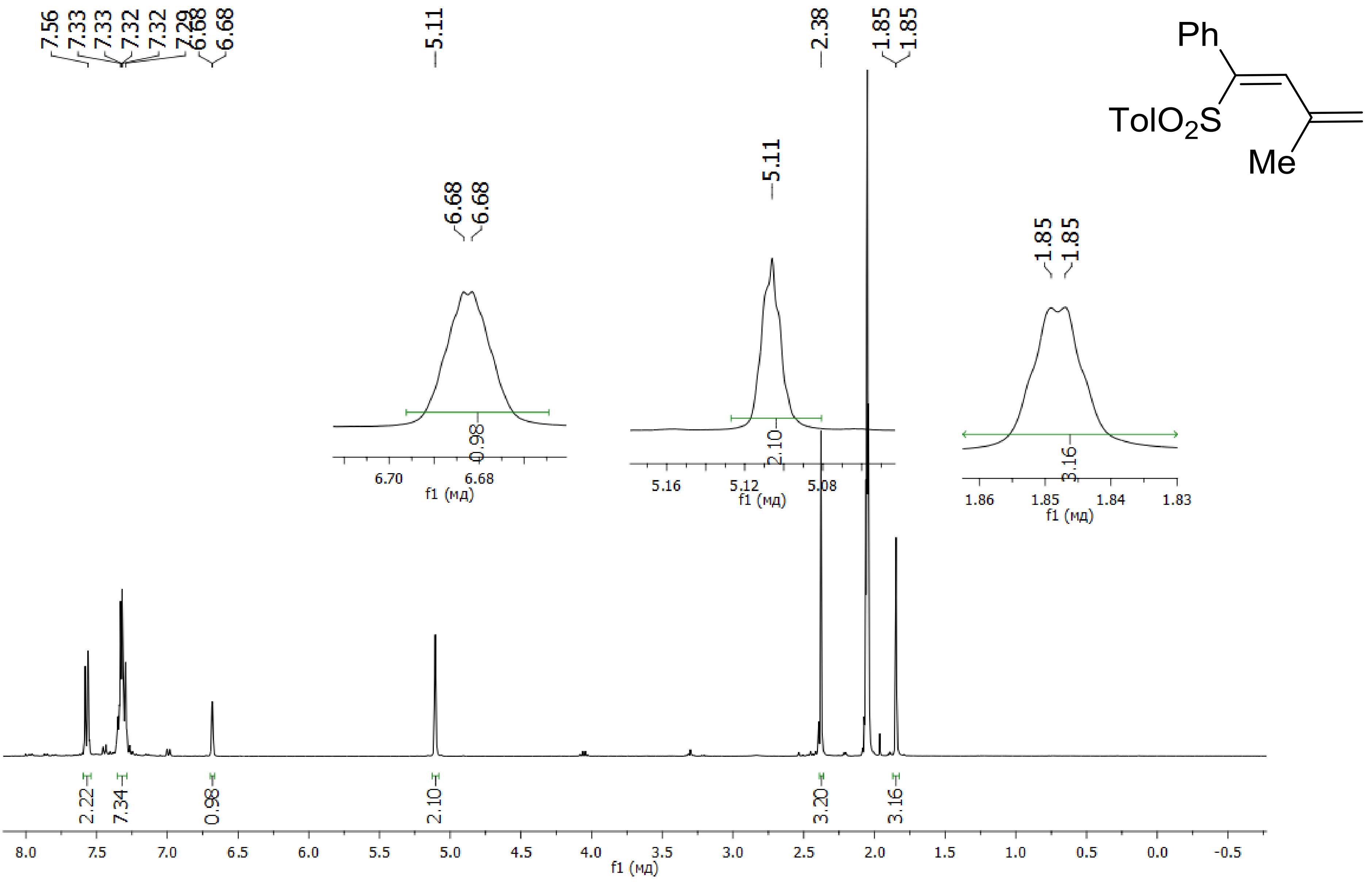

Fig. S20.^{1}H NMR spectrum of the compound **3e** (400 MHz, acetone-d6).

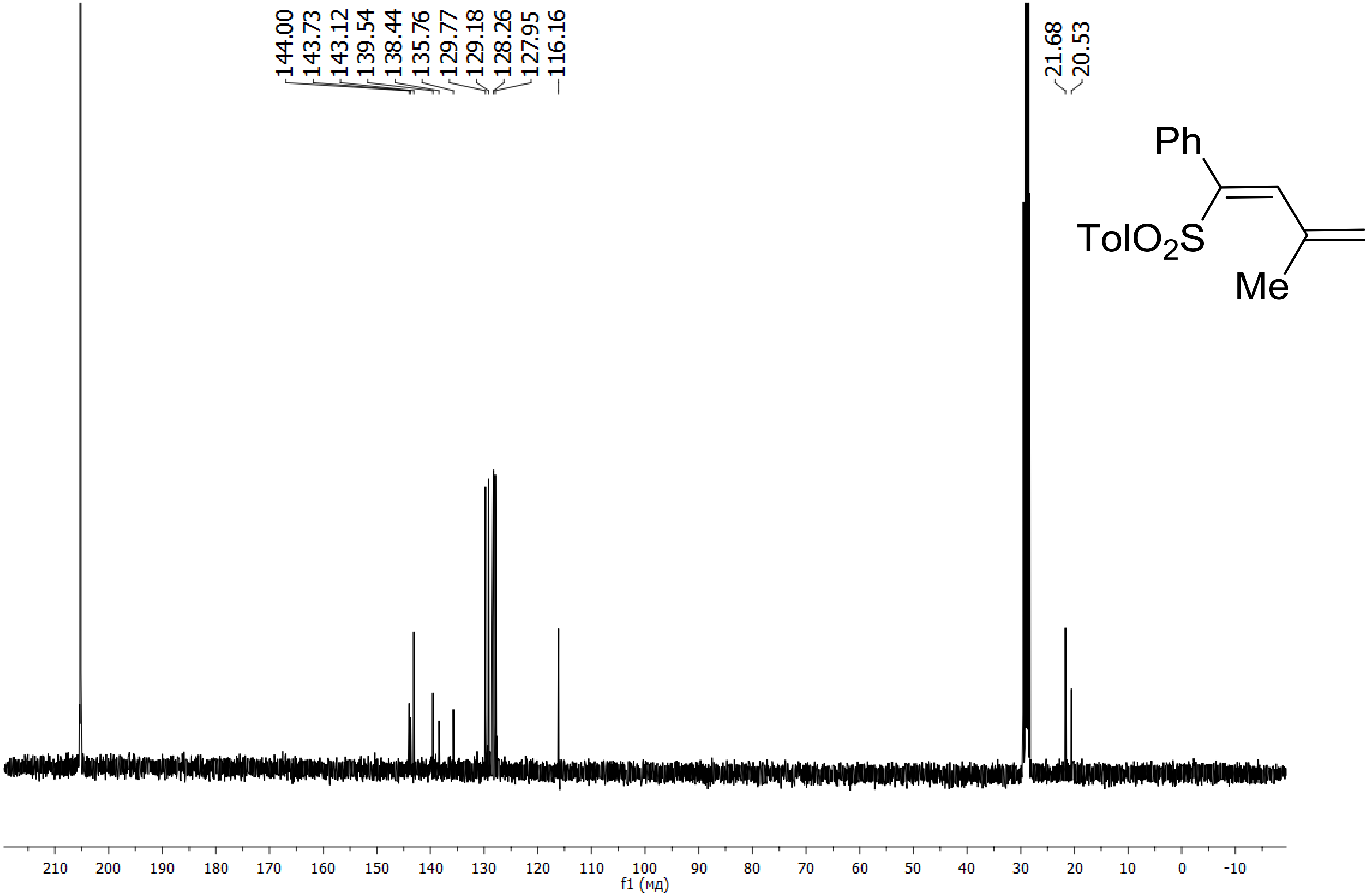

Fig. S21.^{13}C NMR spectrum of the compound **3e** (100 MHz, $CDCl_3$).

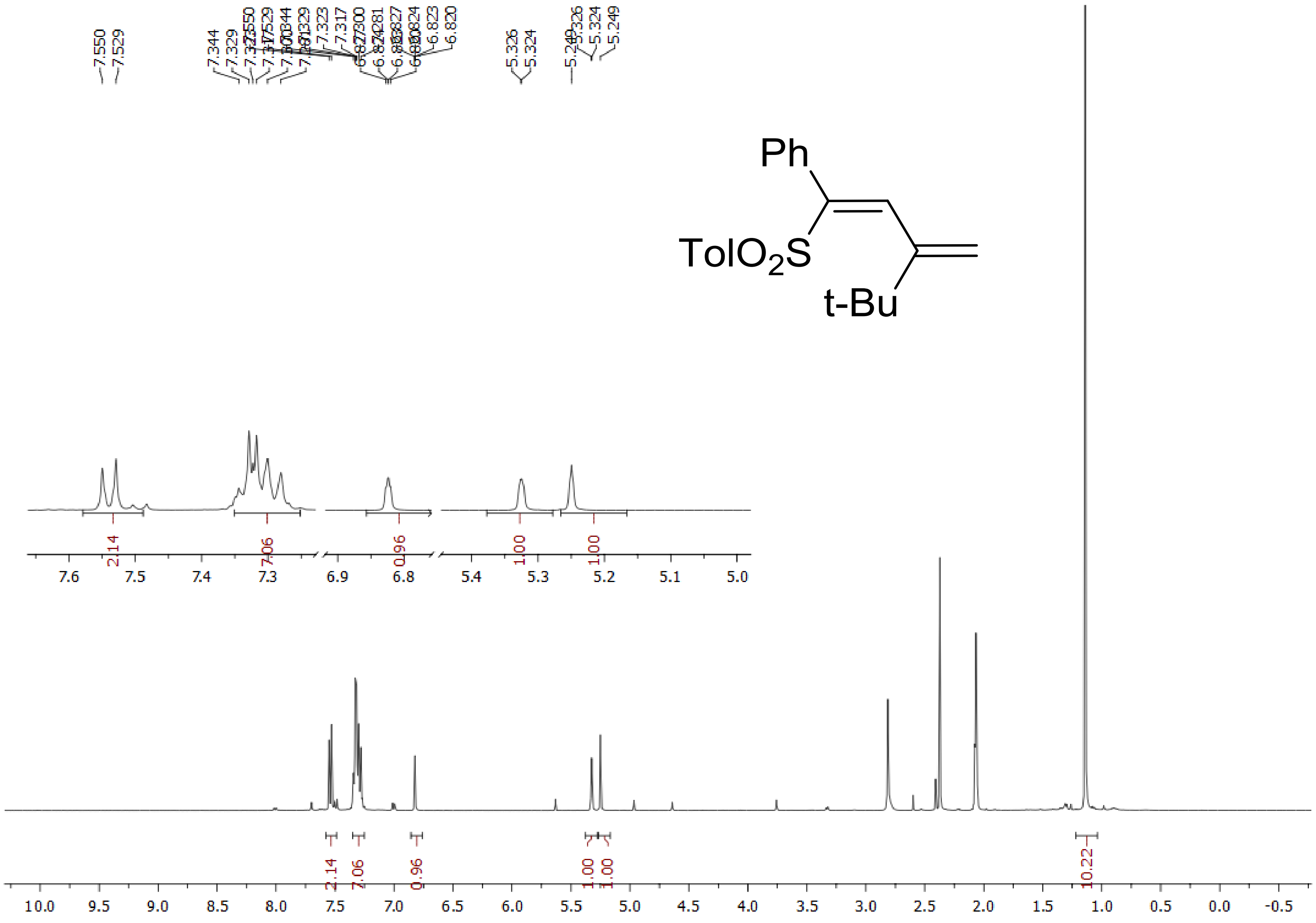

Fig. S22. ^{1}H NMR spectrum of the compound **3f** (400 MHz, acetone-d6).

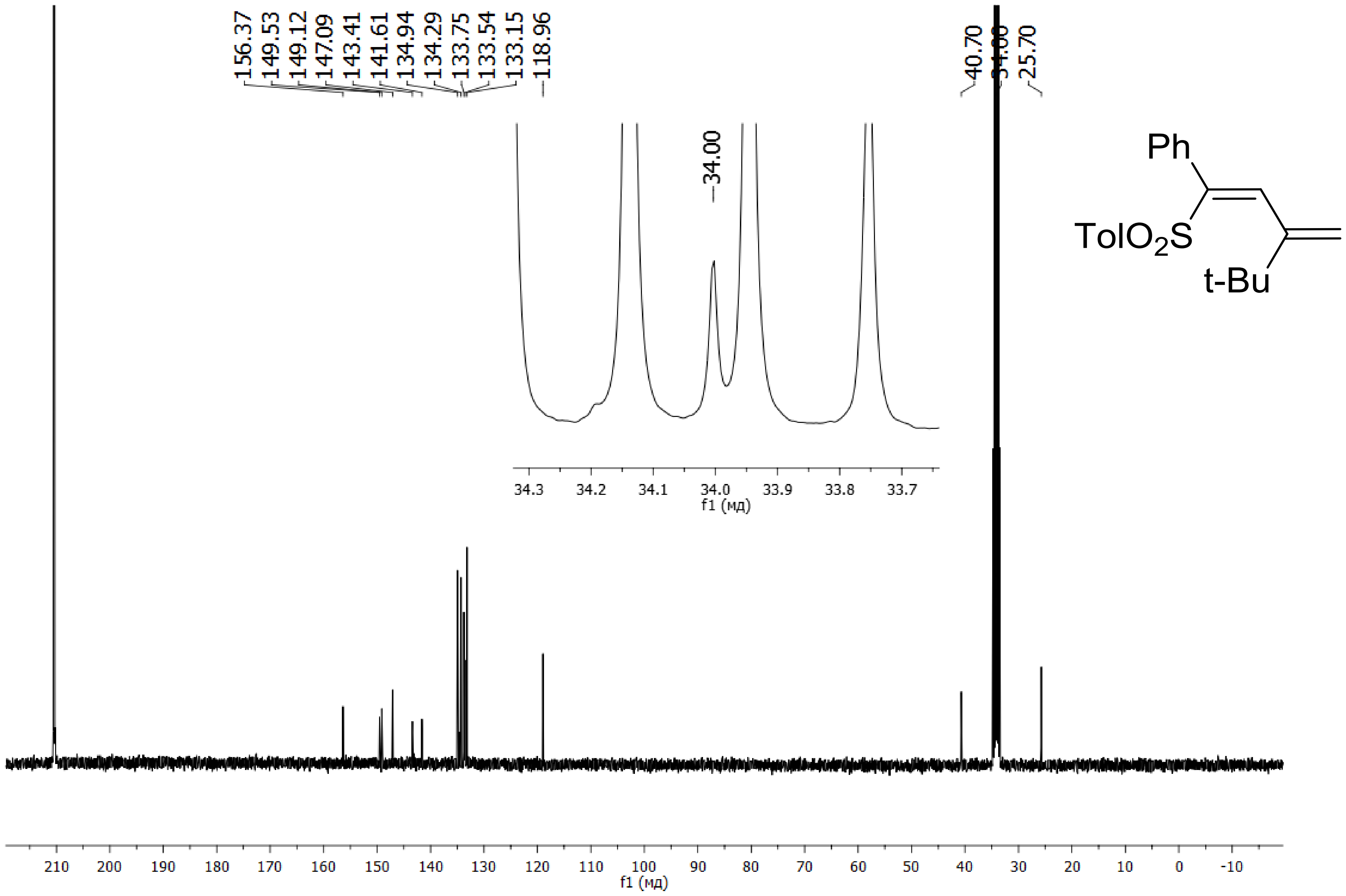

Fig. S23. ^{13}C NMR spectrum of the compound **3f** (100 MHz, acetone-d6).

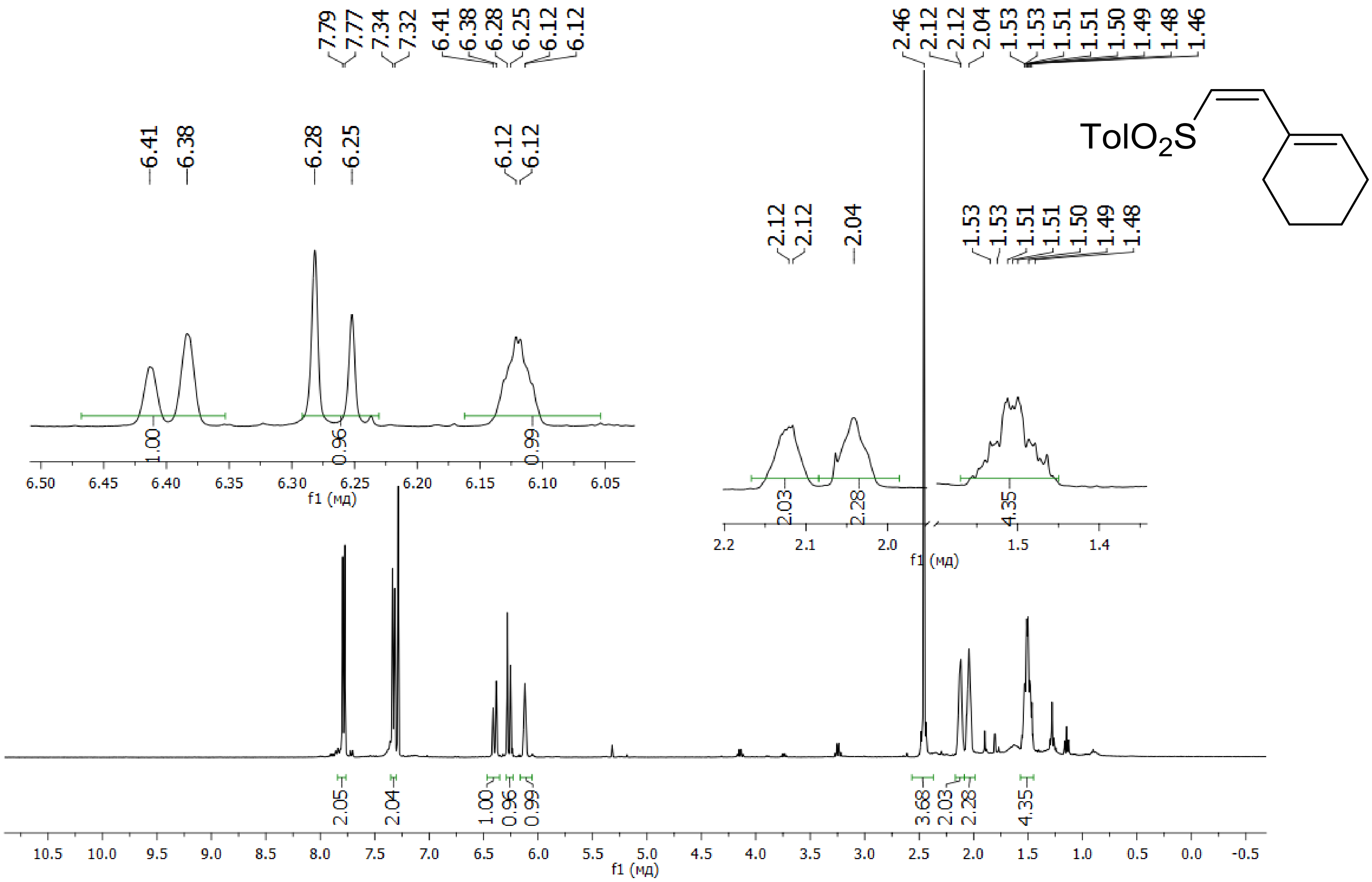

Fig. S24.^{1}H NMR spectrum of the compound **3g** (400 MHz, acetone-d6).

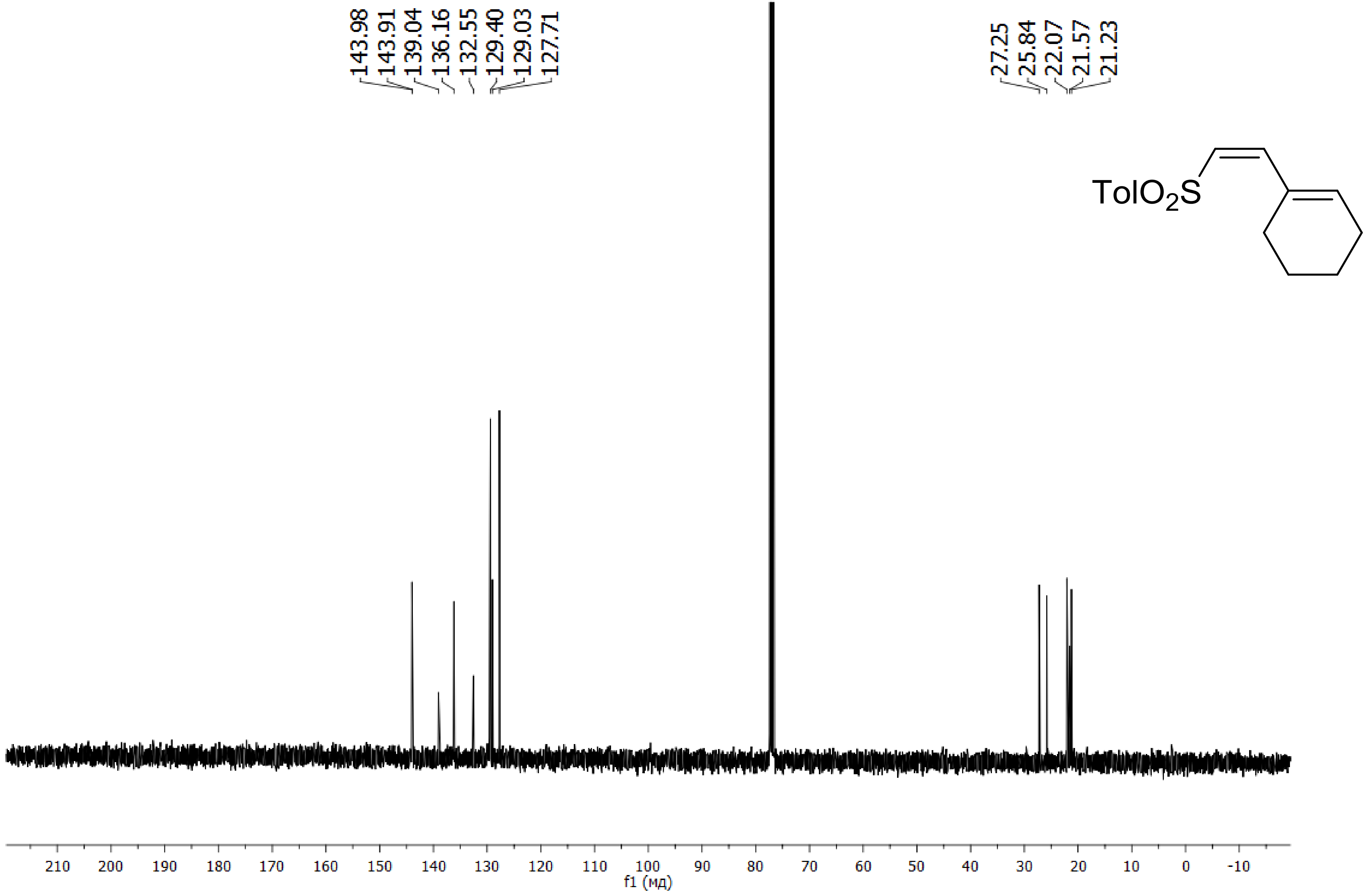

Fig. S25.^{13}C NMR spectrum of the compound **3g** (100 MHz, $CDCl_3$).

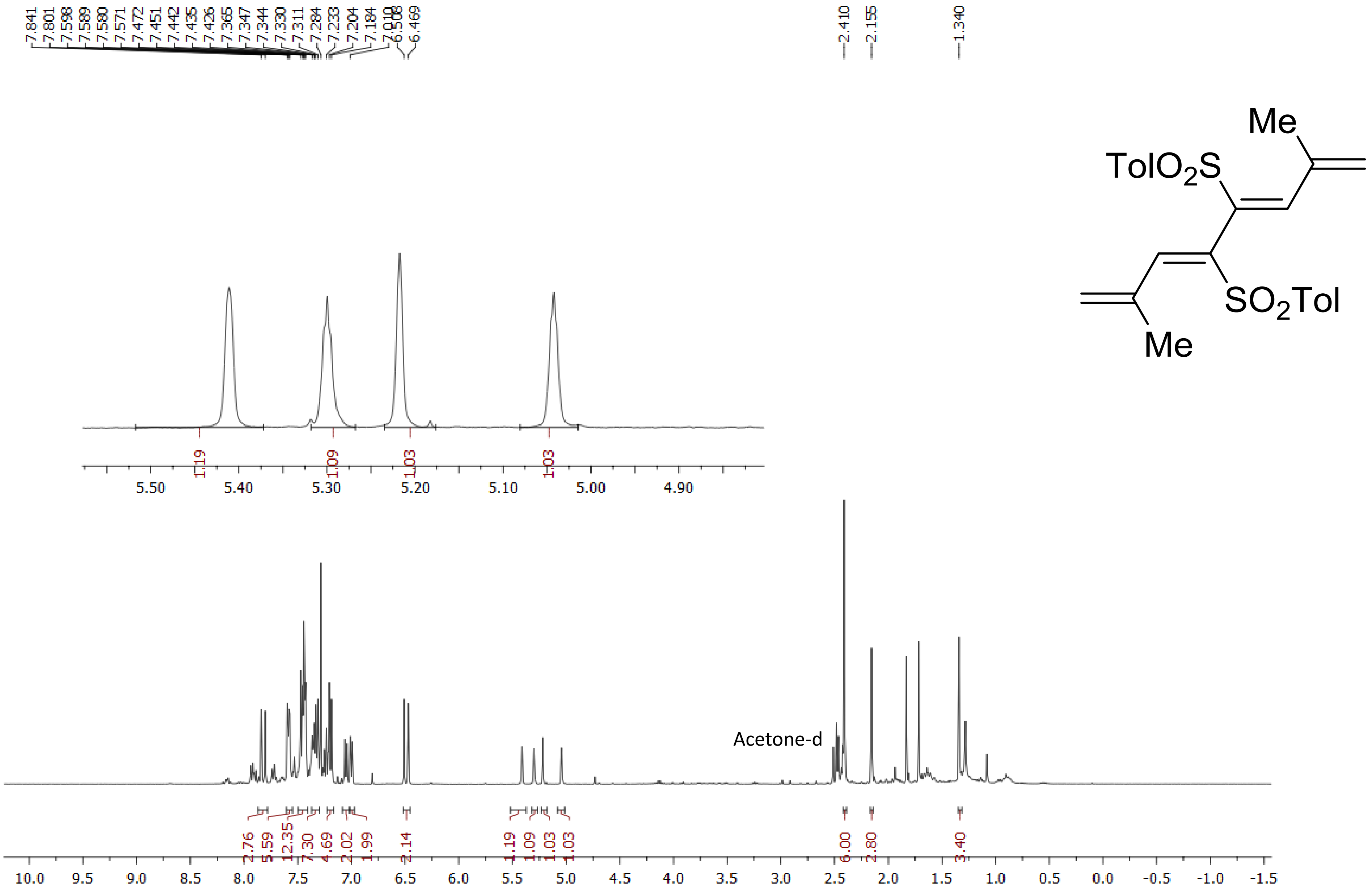

Fig. S26.^{1}H NMR spectrum of the compound **3h** (400 MHz, acetone-d6).

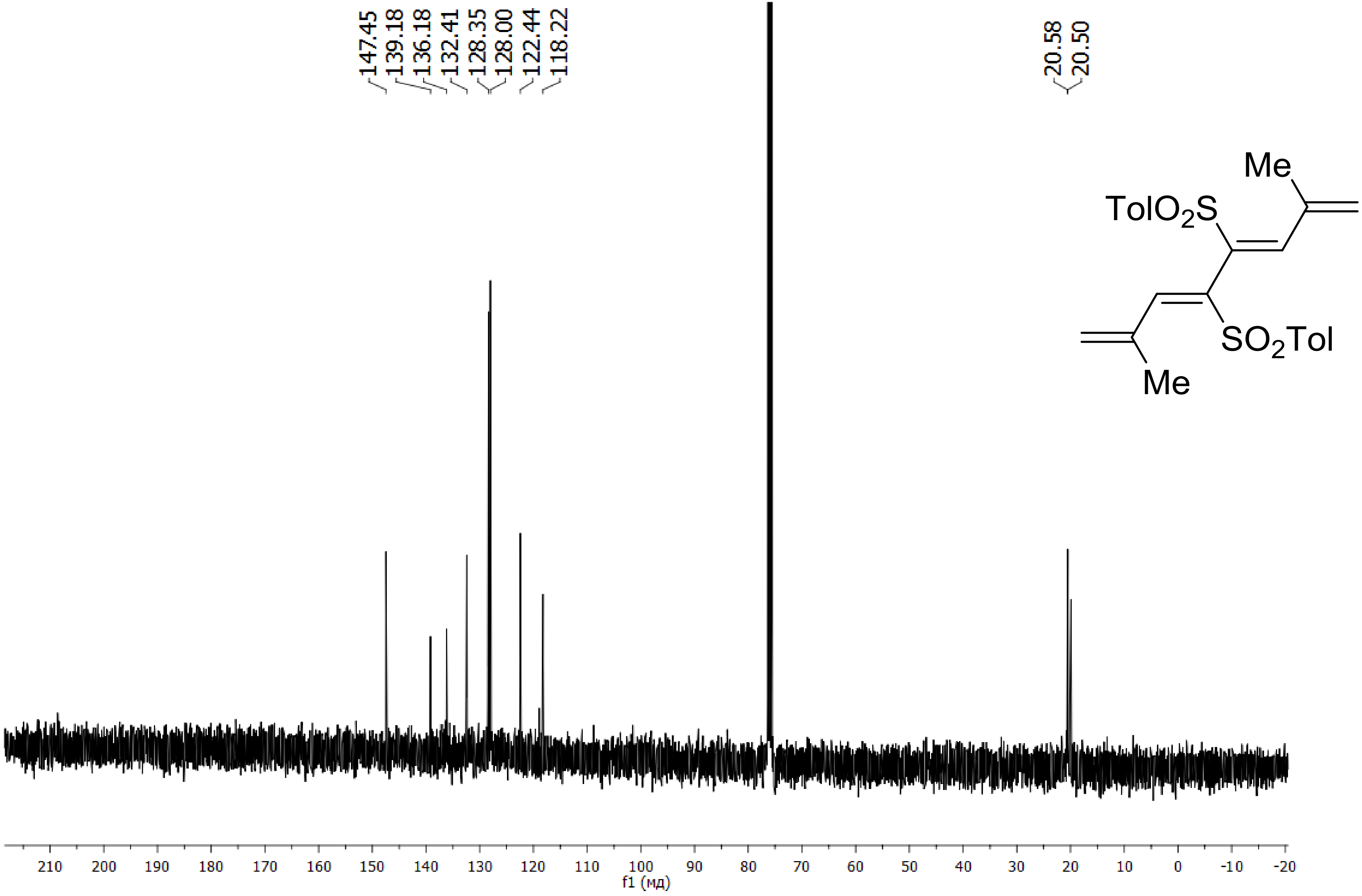

Fig. S27.^{13}C NMR spectrum of the compound **3h** (100 MHz, $CDCl_3$).

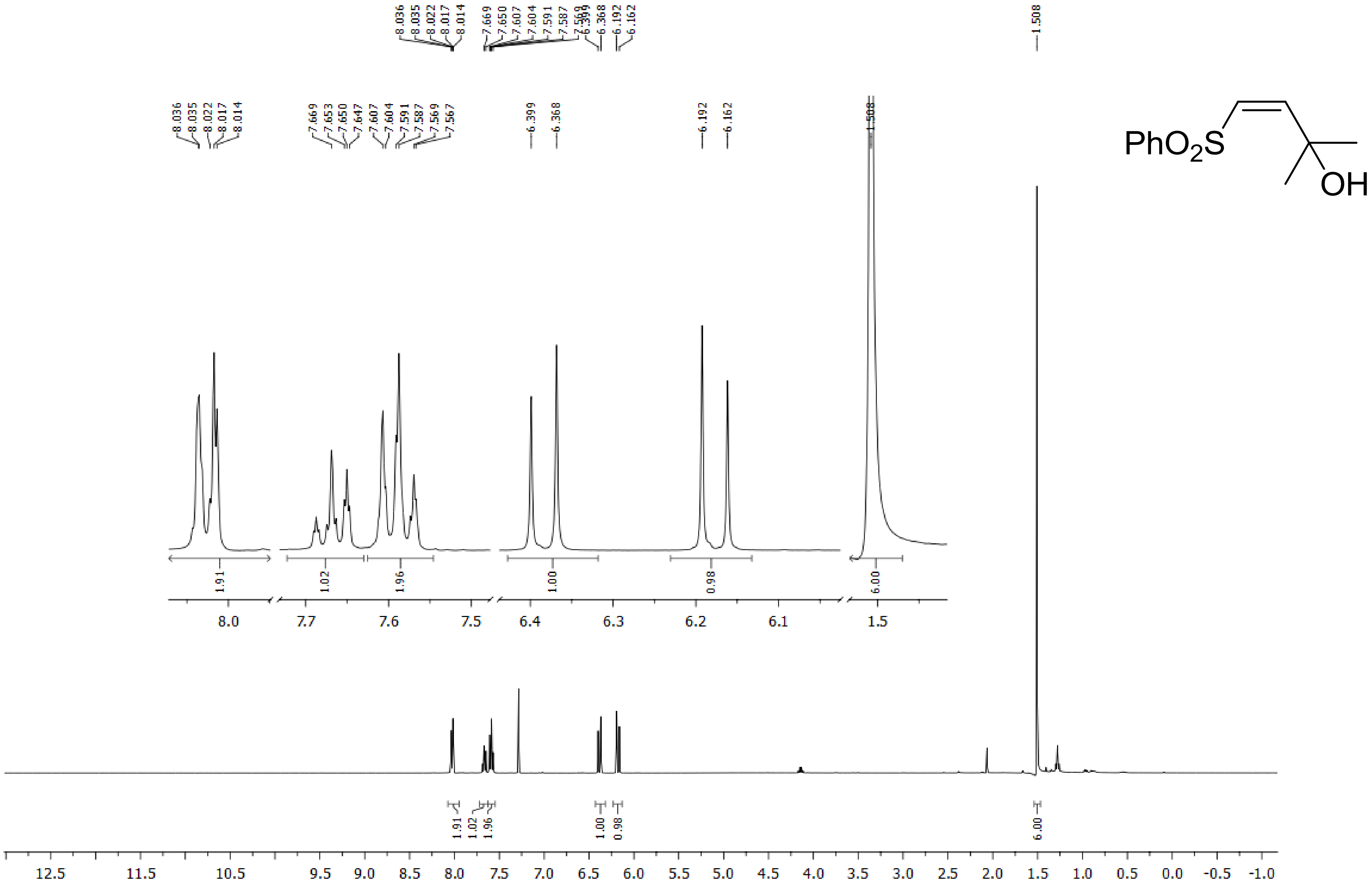

Fig. S28. ^{1}H NMR spectrum of the compound **4a** (400 MHz, $CDCl_3$).

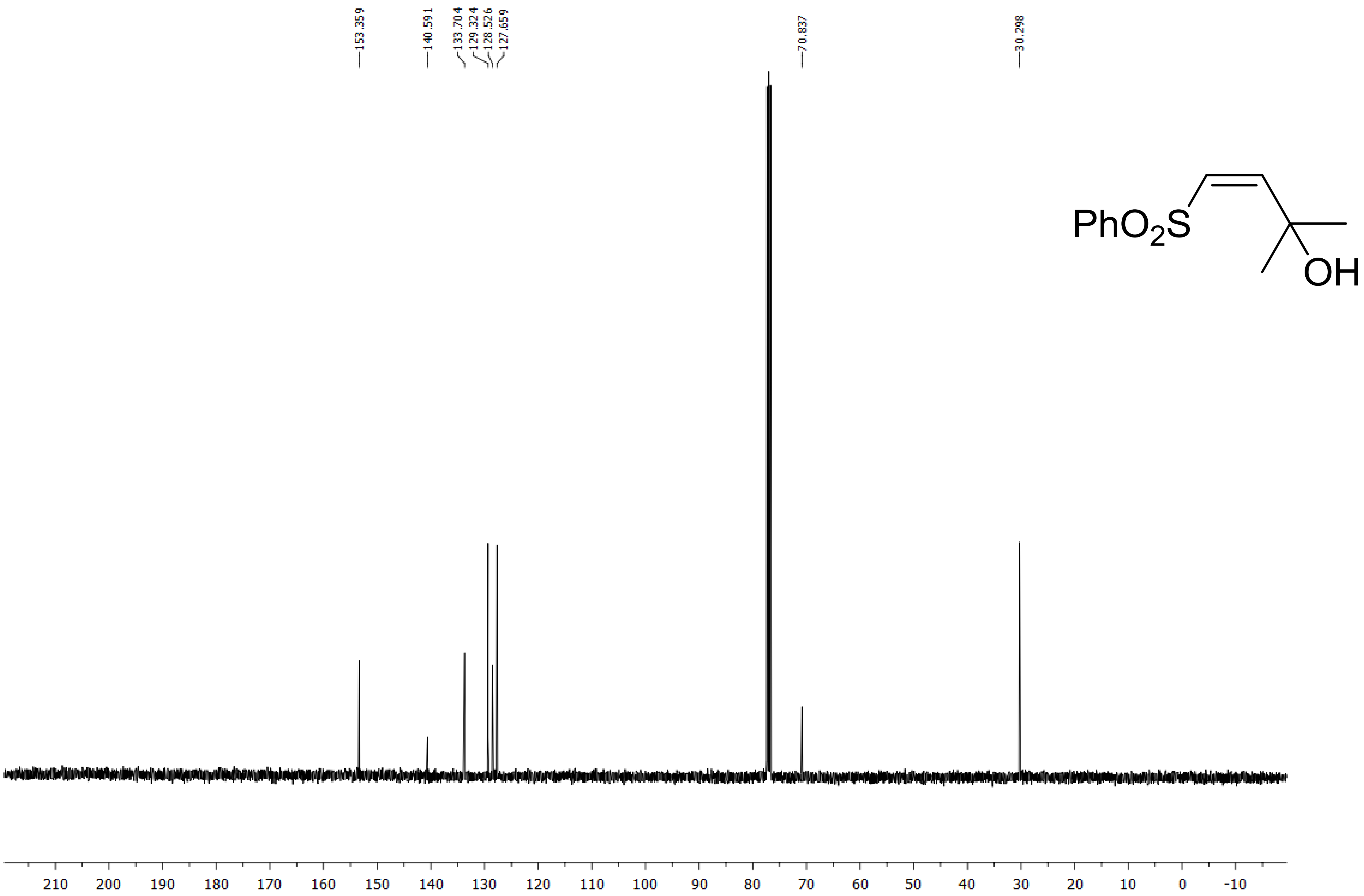

Fig. S29.^{13}C NMR spectrum of the compound **4a** (100 MHz, $CDCl_3$).

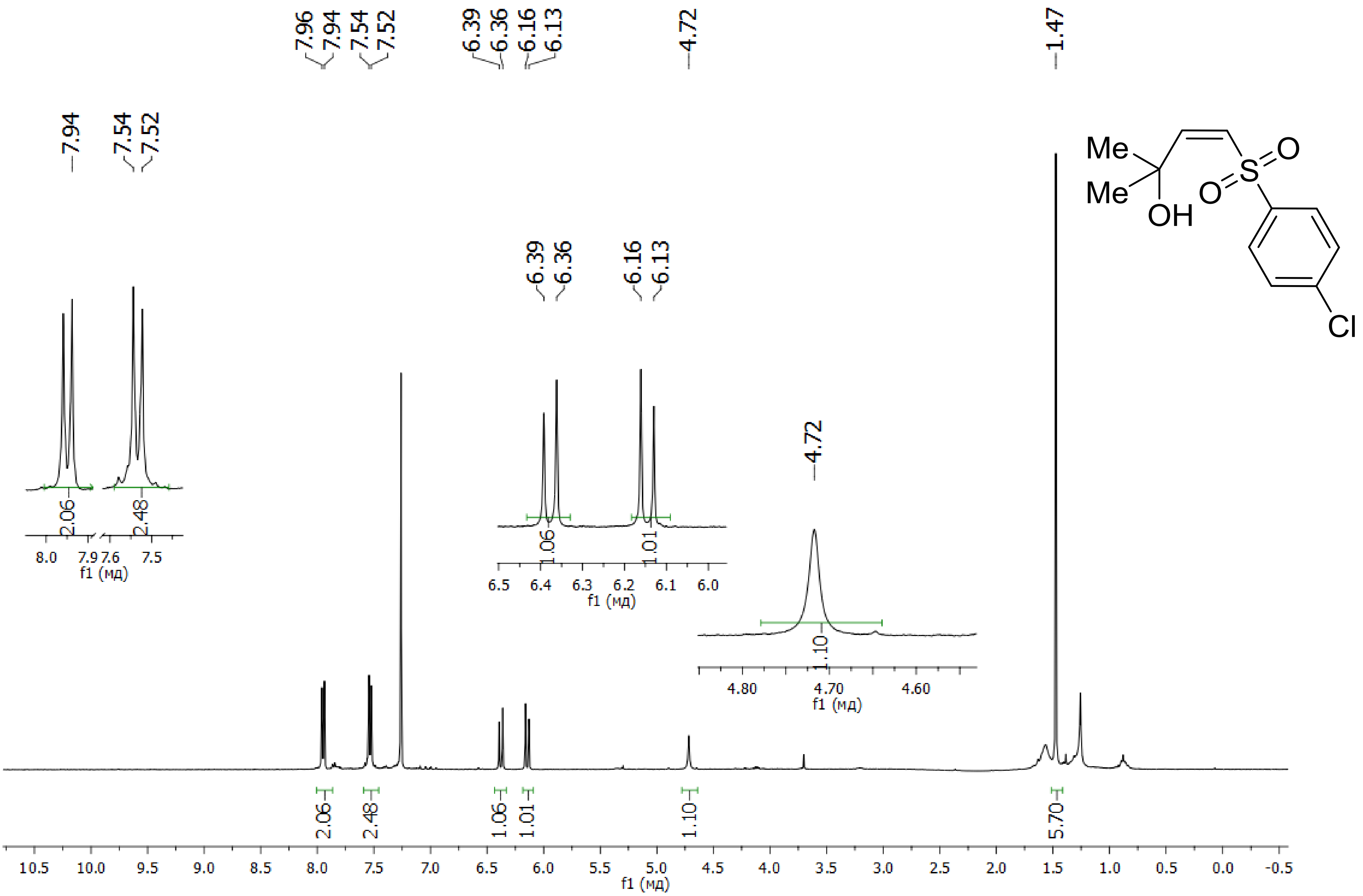

Fig. S30.^{1}H NMR spectrum of the compound **4b** (400 MHz,$CDCl_3$).

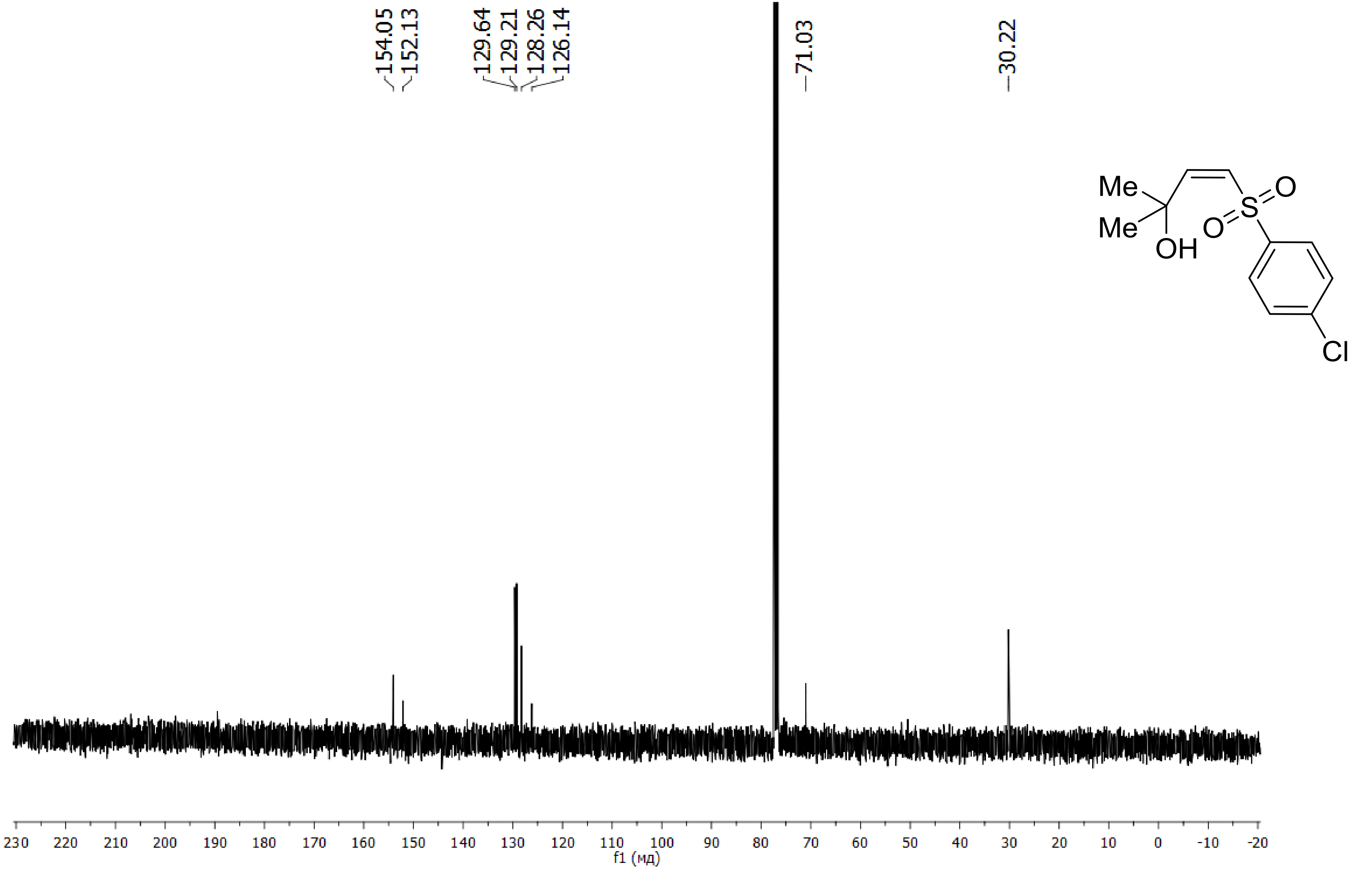

Fig. S31.^{13}C NMR spectrum of the compound **4b** (100 MHz,$CDCl_3$).

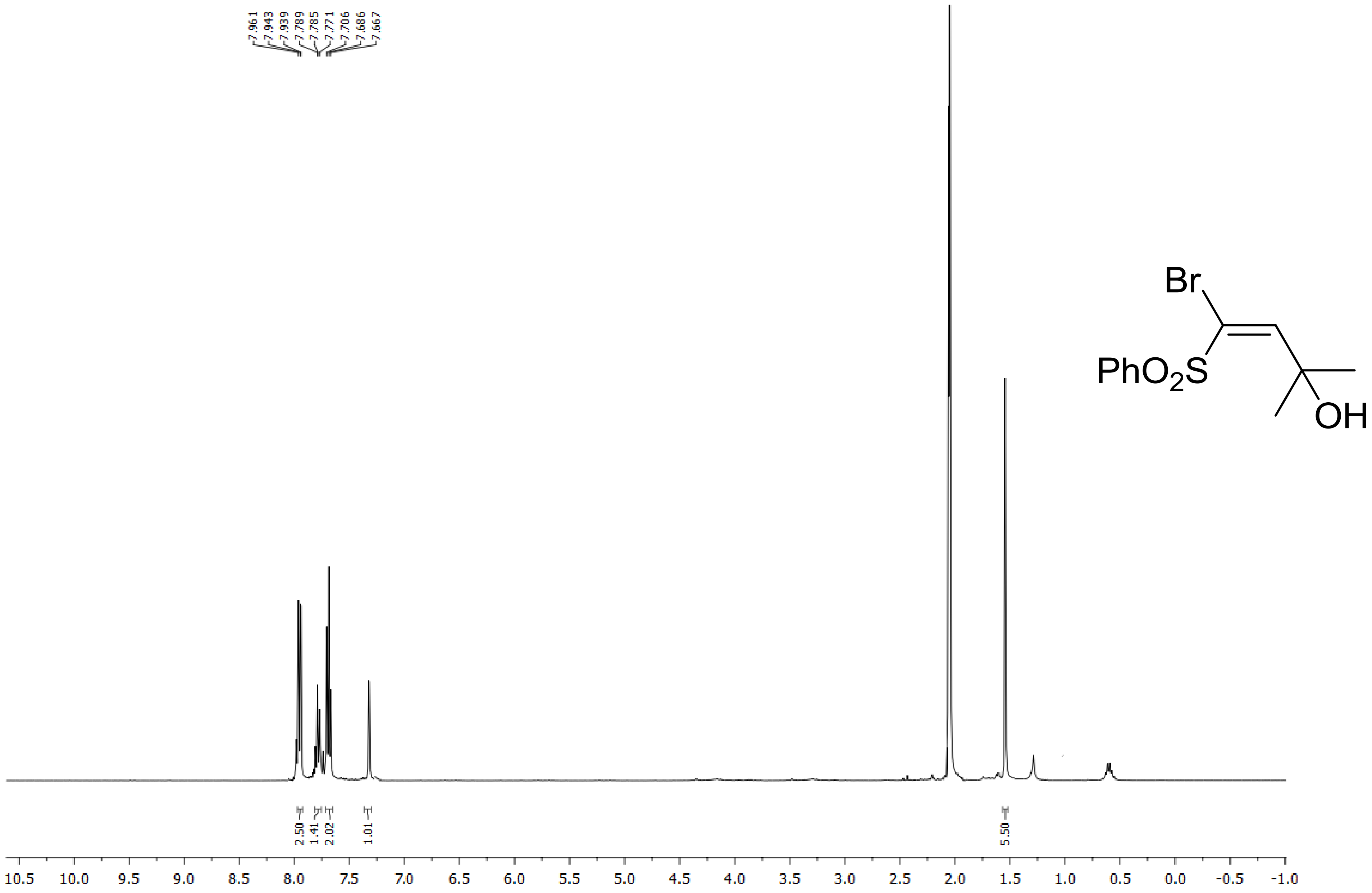

Fig. S32. ^{1}H NMR spectrum of the compound **4c** (400 MHz, acetone-d6).

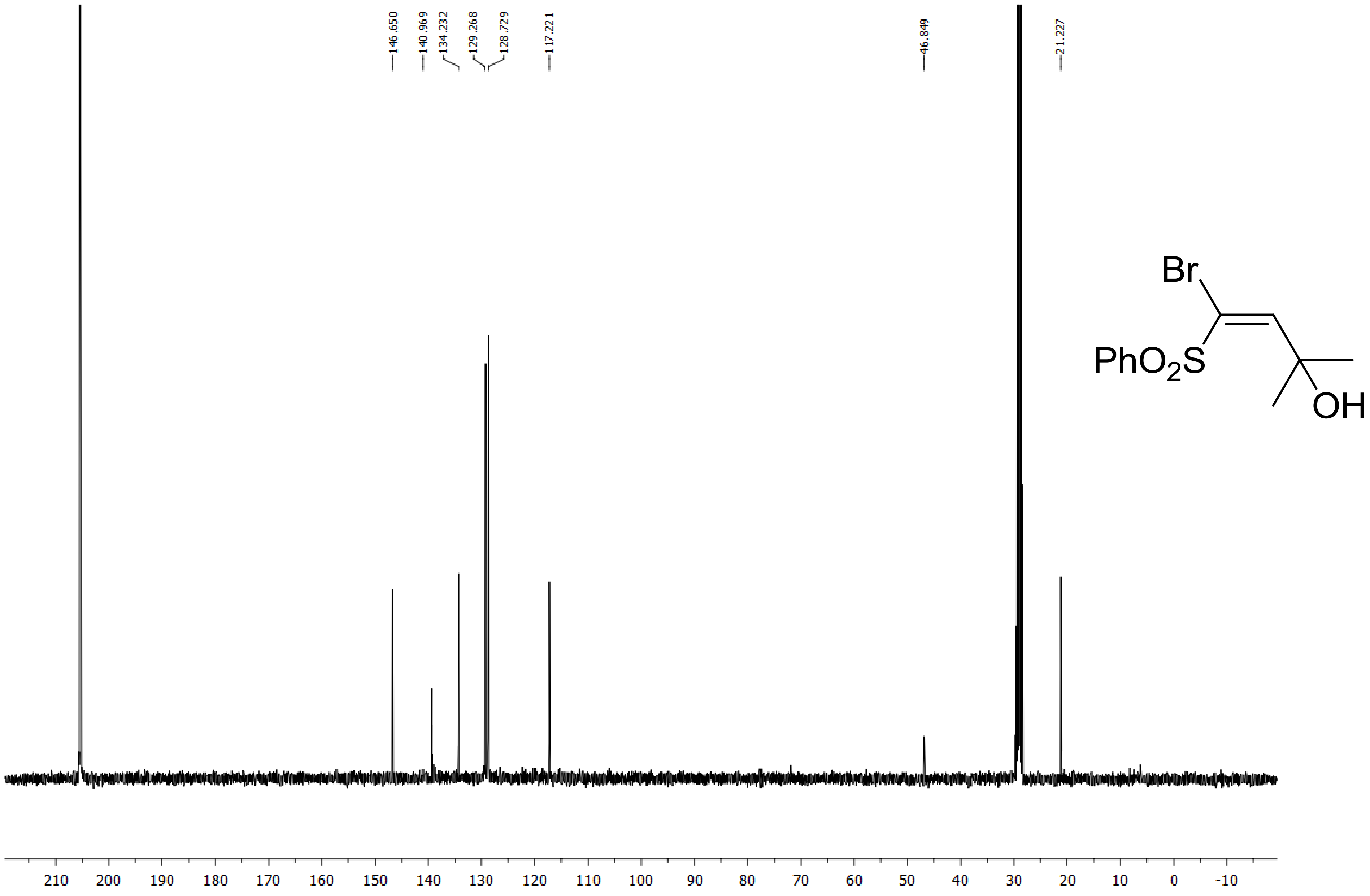

Fig. S33.^{13}C NMR spectrum of the compound **4c** (100 MHz, acetone-d6).

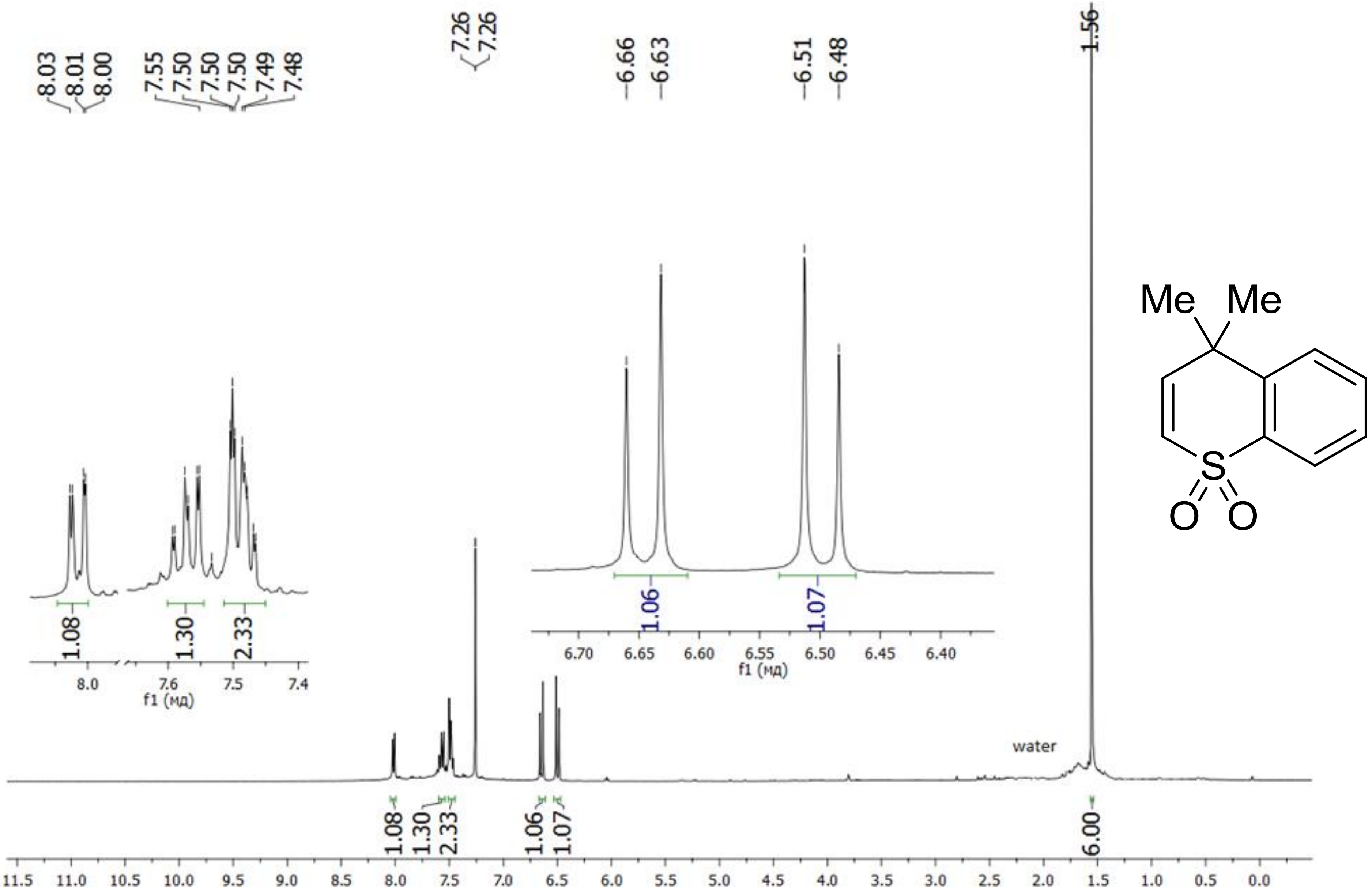

Fig. S34.^{1}H NMR spectrum of the compound **5a** (400 MHz, $CDCl_3$).

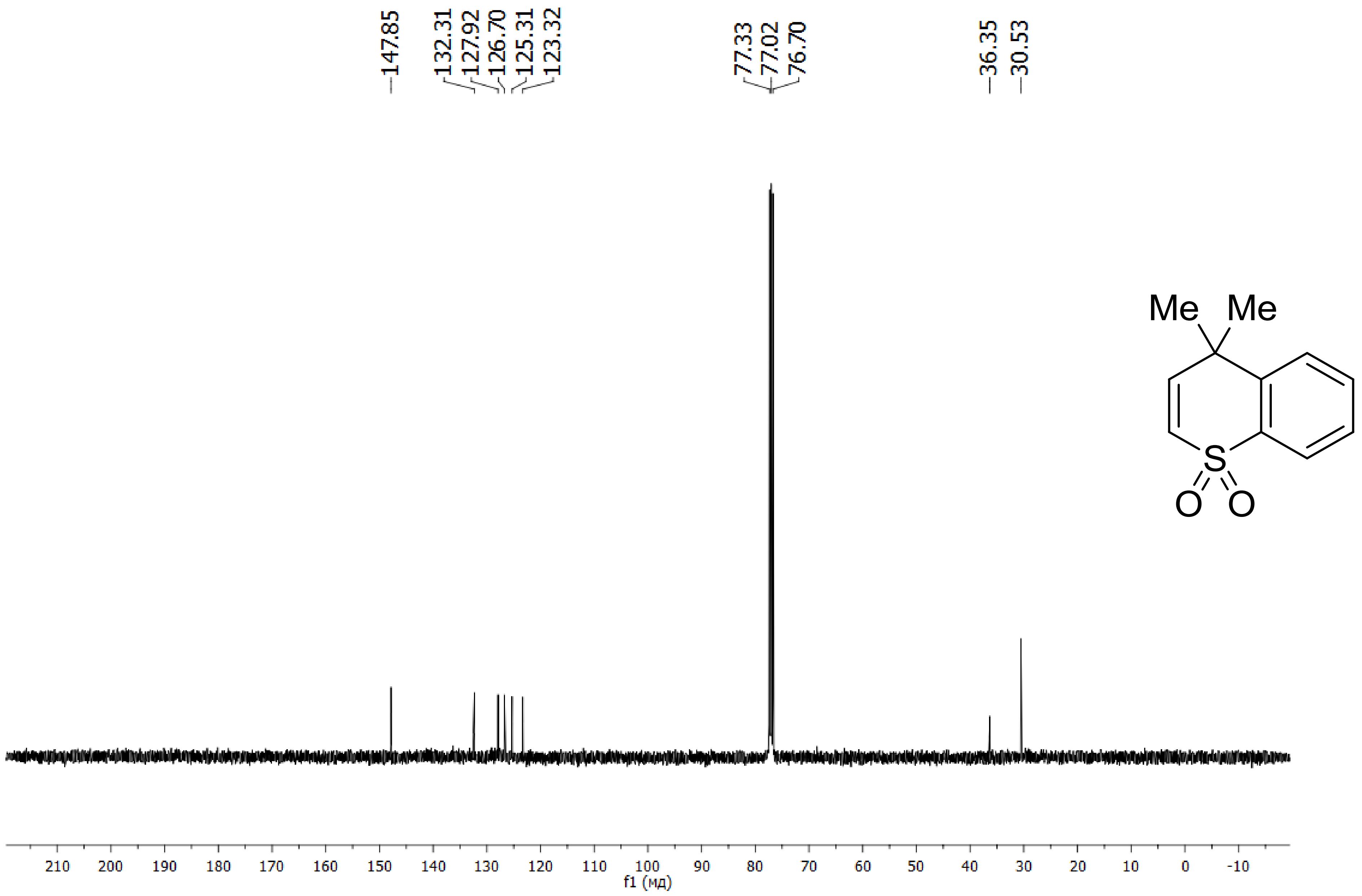

Fig. S35.^{13}C NMR spectrum of the compound **5a** (100 MHz, $CDCl_3$).

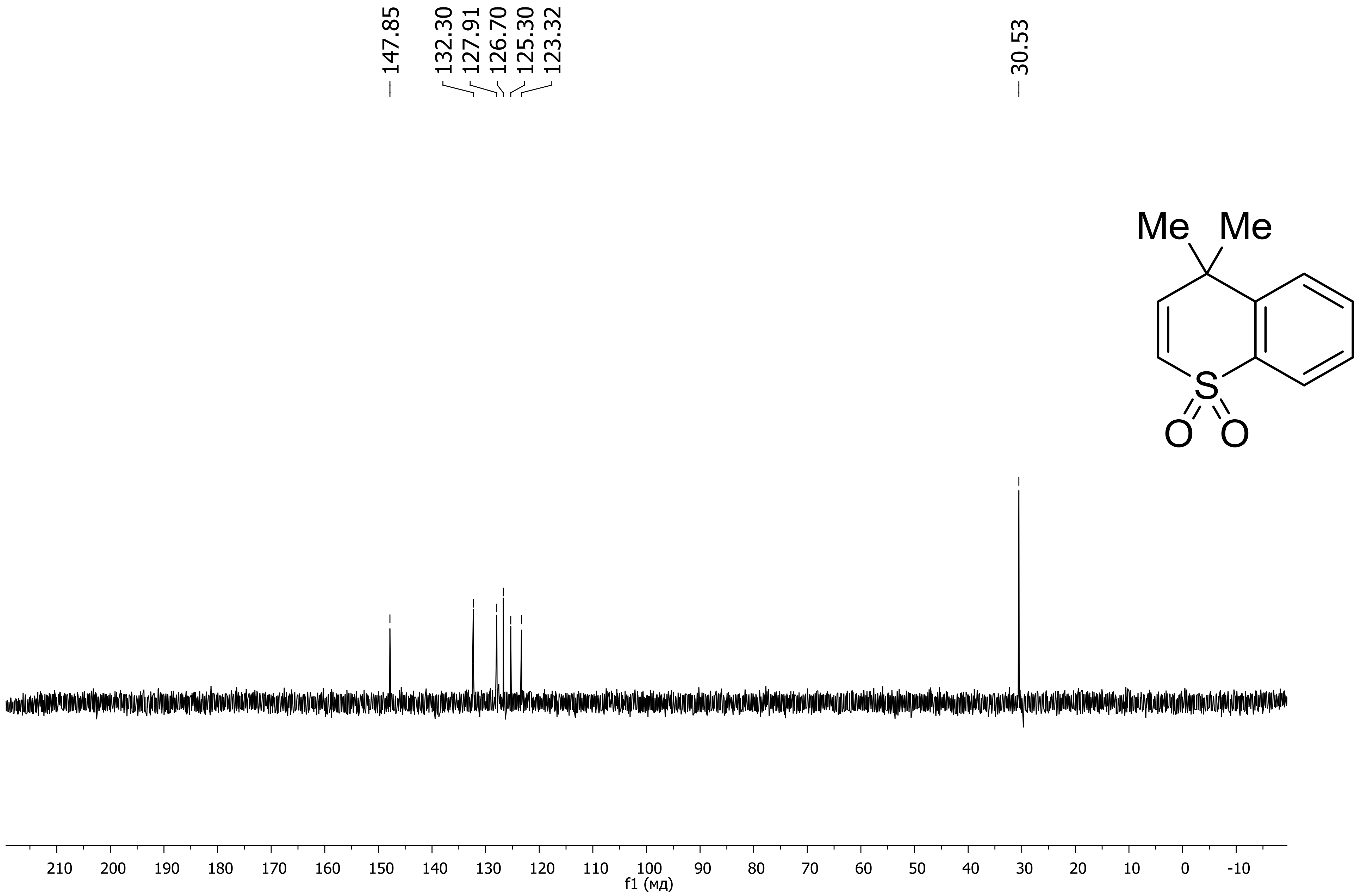

Fig. S36. DEPT NMR spectrum of the compound **5a** (100 MHz, $CDCl_3$).

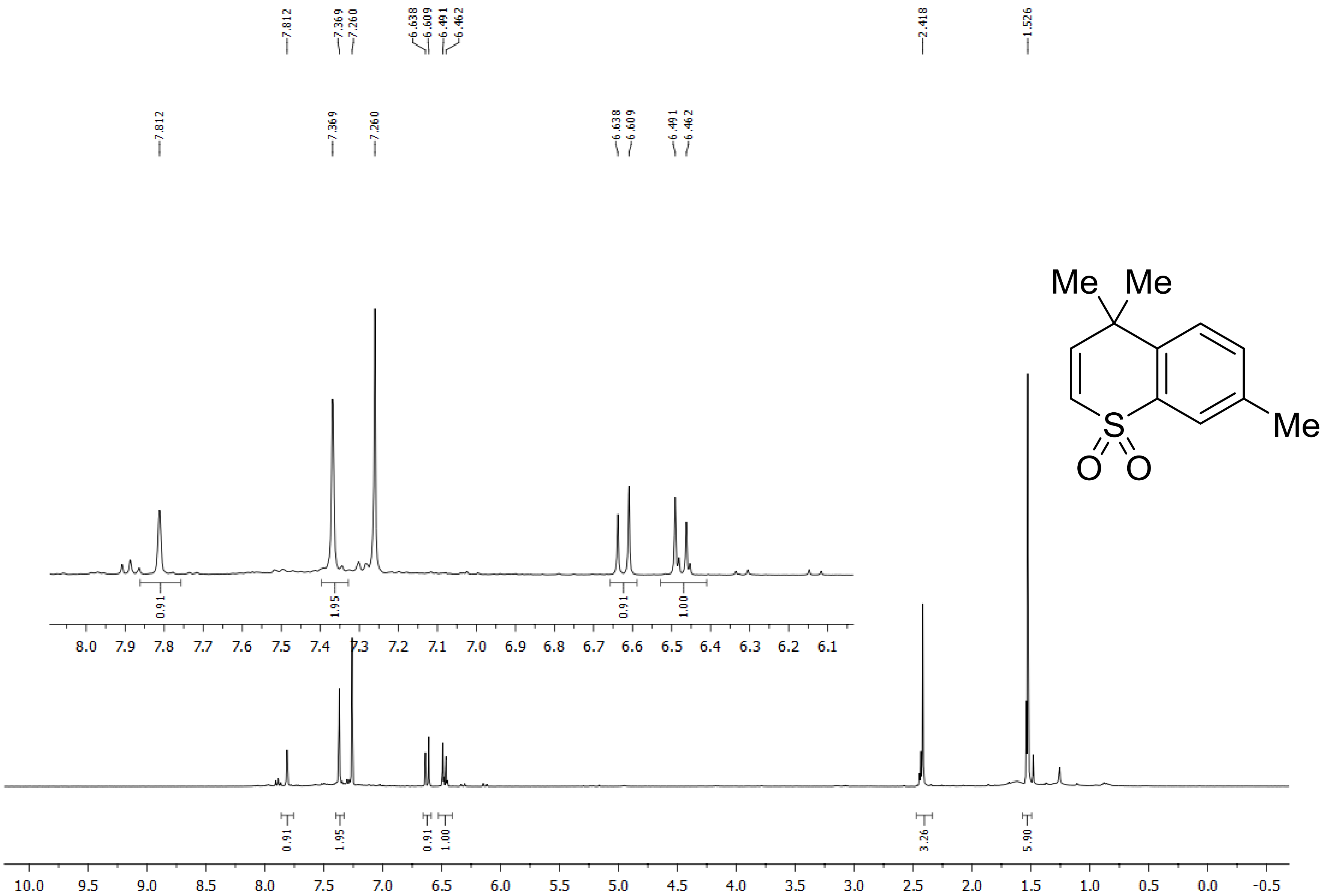

Fig. S37.^{1}H NMR spectrum of the compound **5b** (400 MHz, $CDCl_3$).

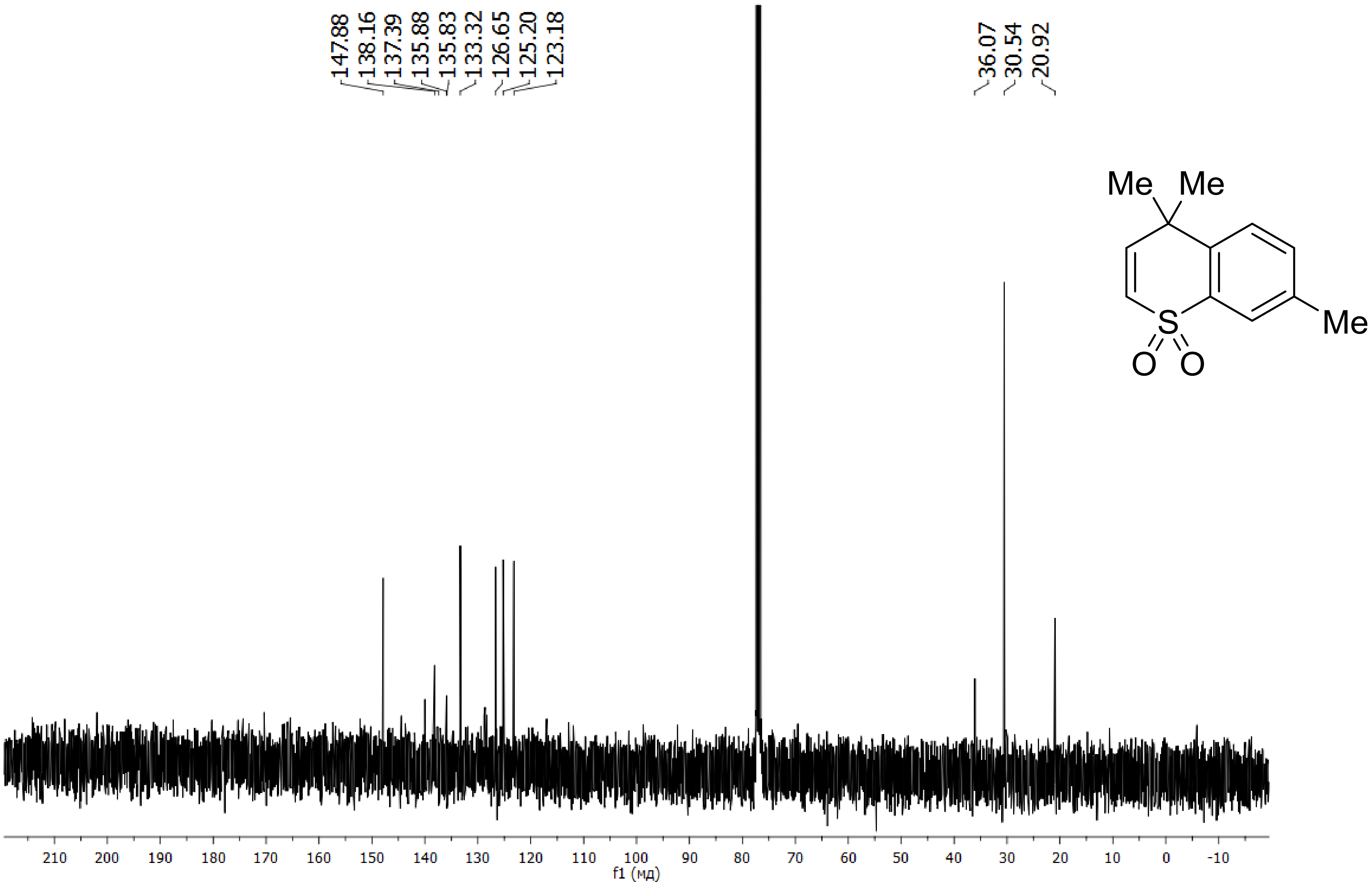

Fig. S38.^{13}C NMR spectrum of the compound **5b** (100 MHz, $CDCl_3$).

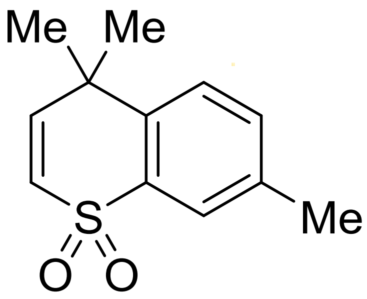

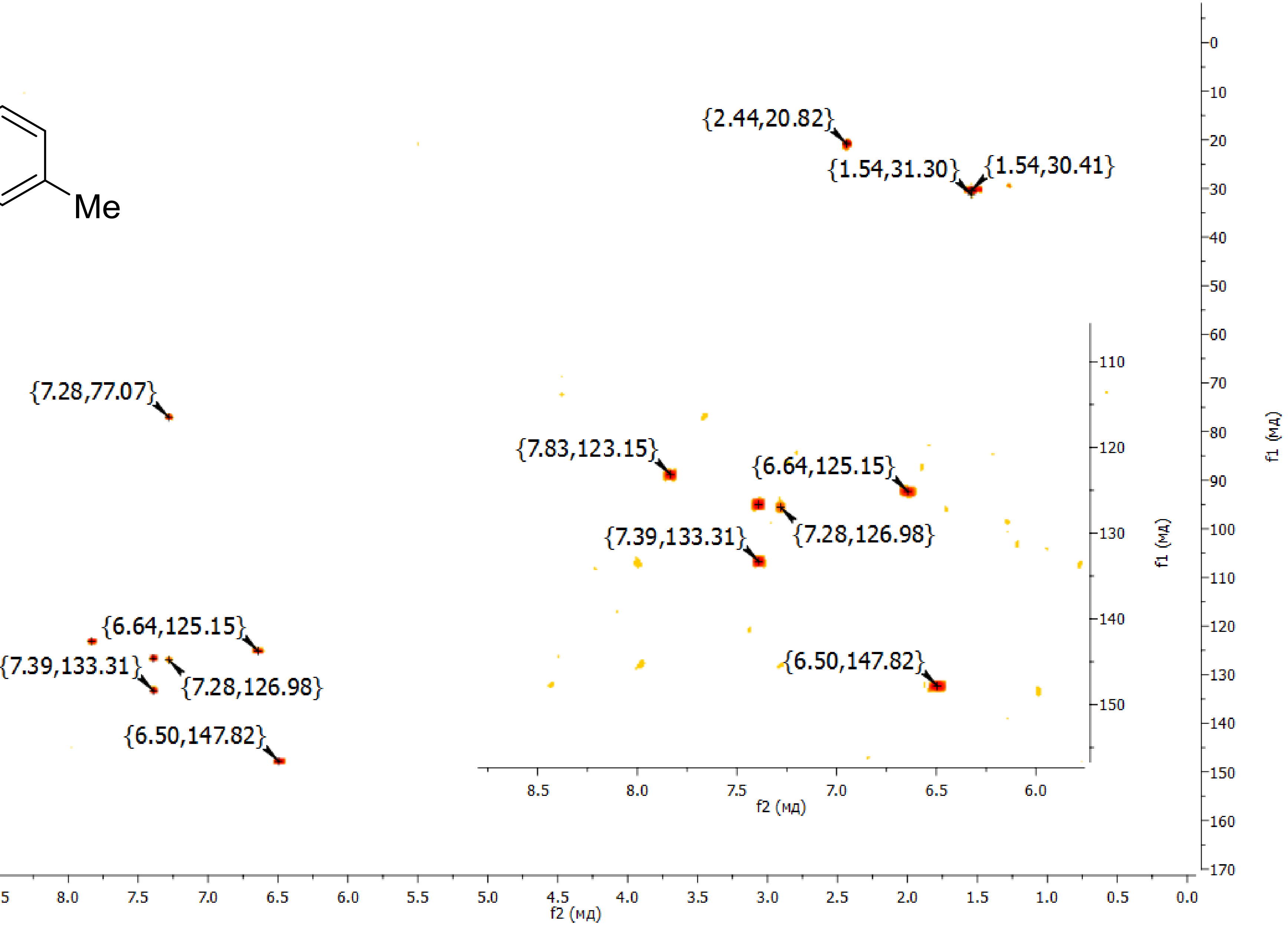

Fig. S39. HMQC NMR spectrum of the compound **5b** (100 MHz, $CDCl_3$).

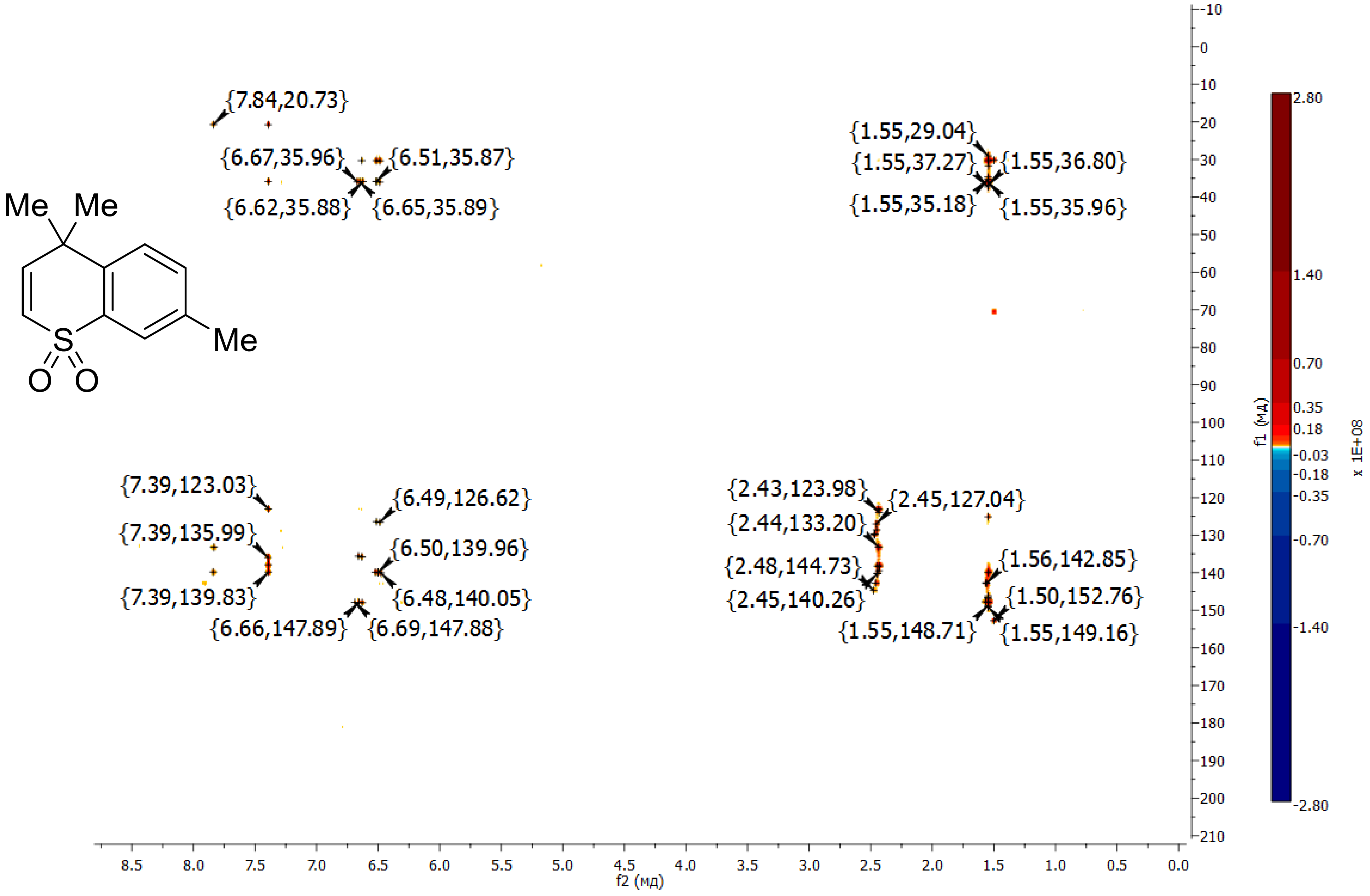

Fig. S40. HMBC NMR spectrum of the compound **5b** (100 MHz, $CDCl_3$).

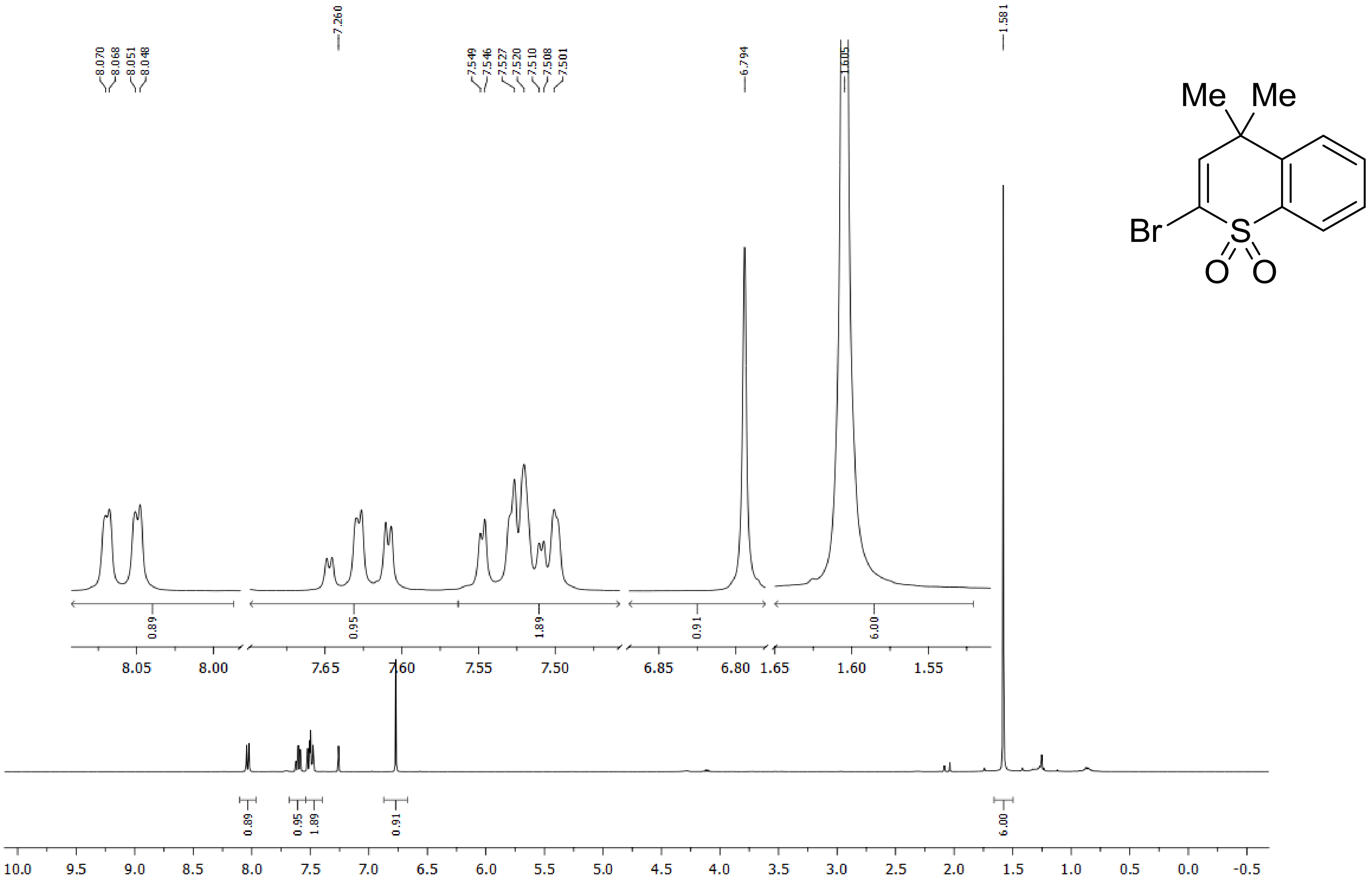

Fig. S41. ^{1}H NMR spectrum of the compound **5c** (400 MHz, $CDCl_3$).

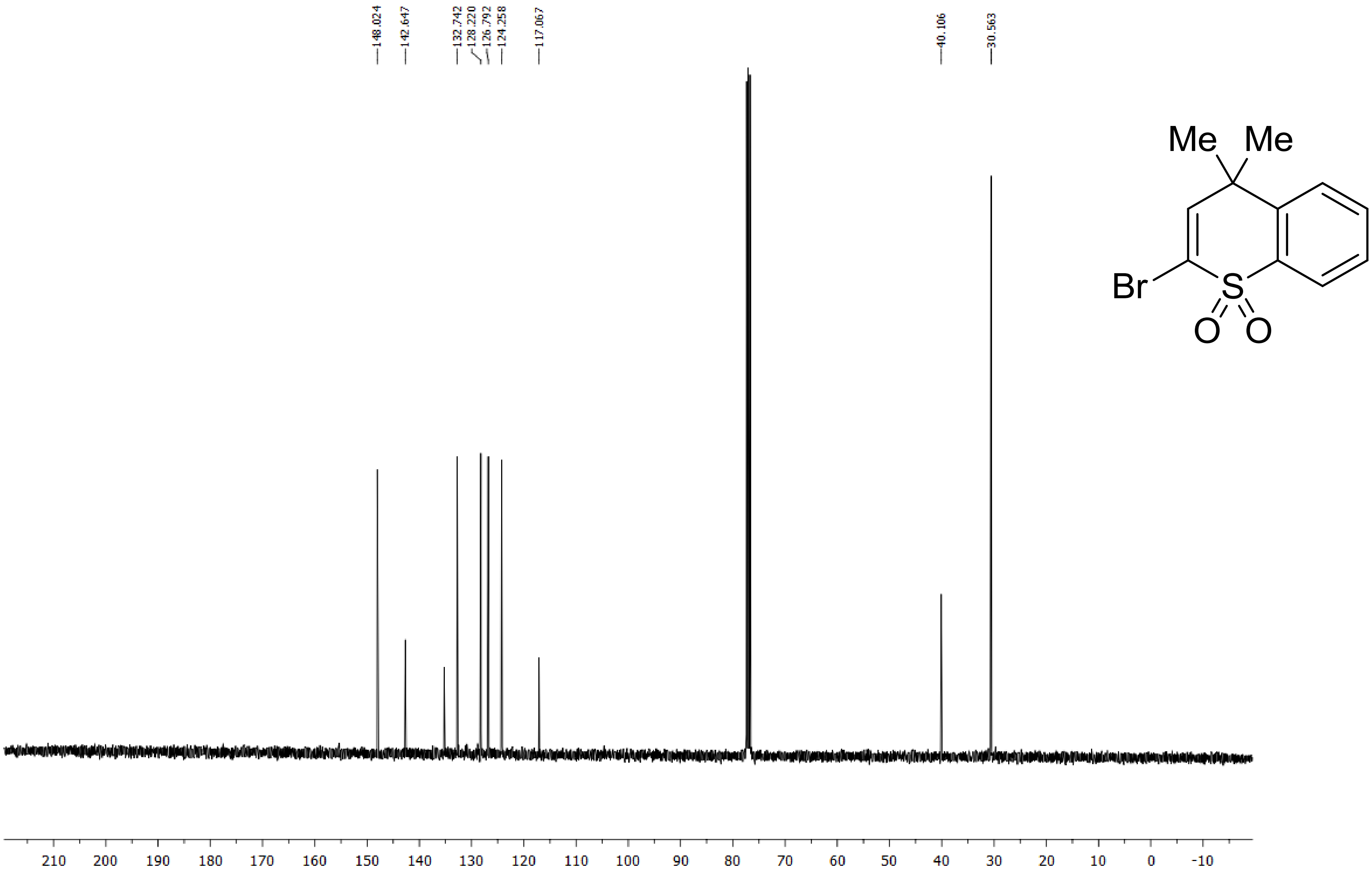

Fig. S42. ^{13}C NMR spectrum of the compound **5c** (100 MHz, $CDCl_3$).

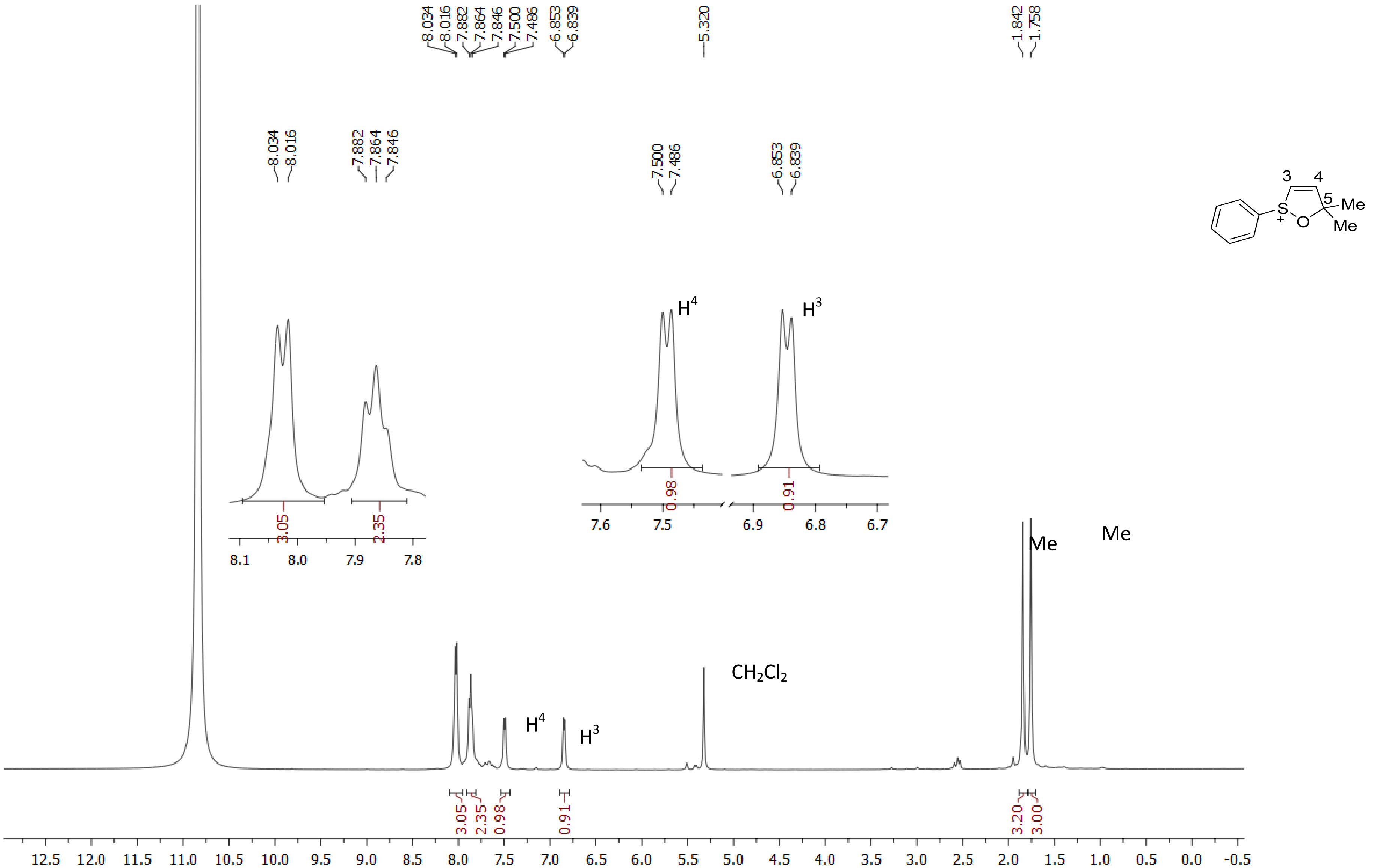

Fig. S43. ^{1}H NMR spectrum of the cation **Aa** (400 MHz, TfOH).

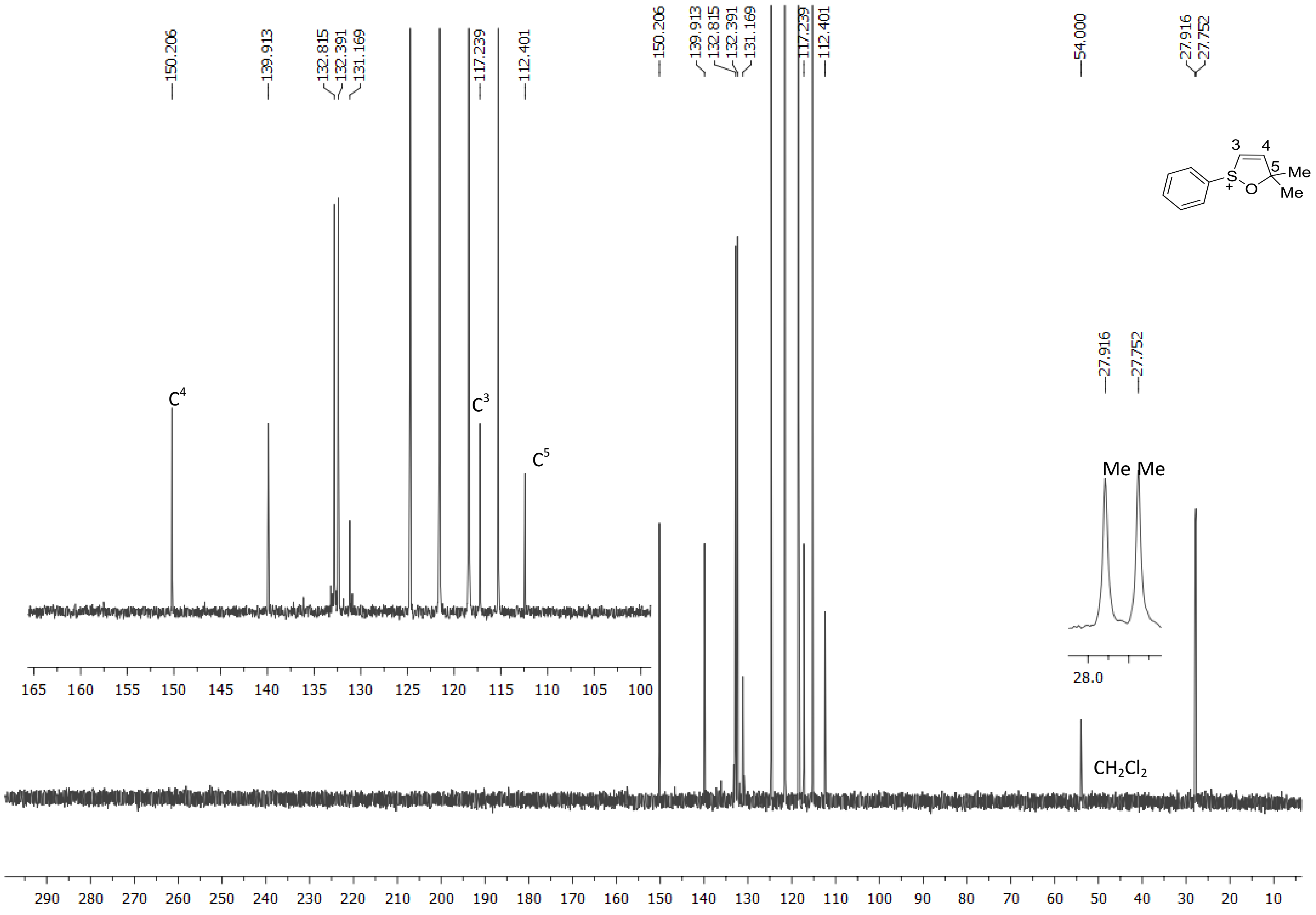

Fig. S44. ^{13}C NMR spectrum of the cation **Aa** (101 MHz, TfOH).

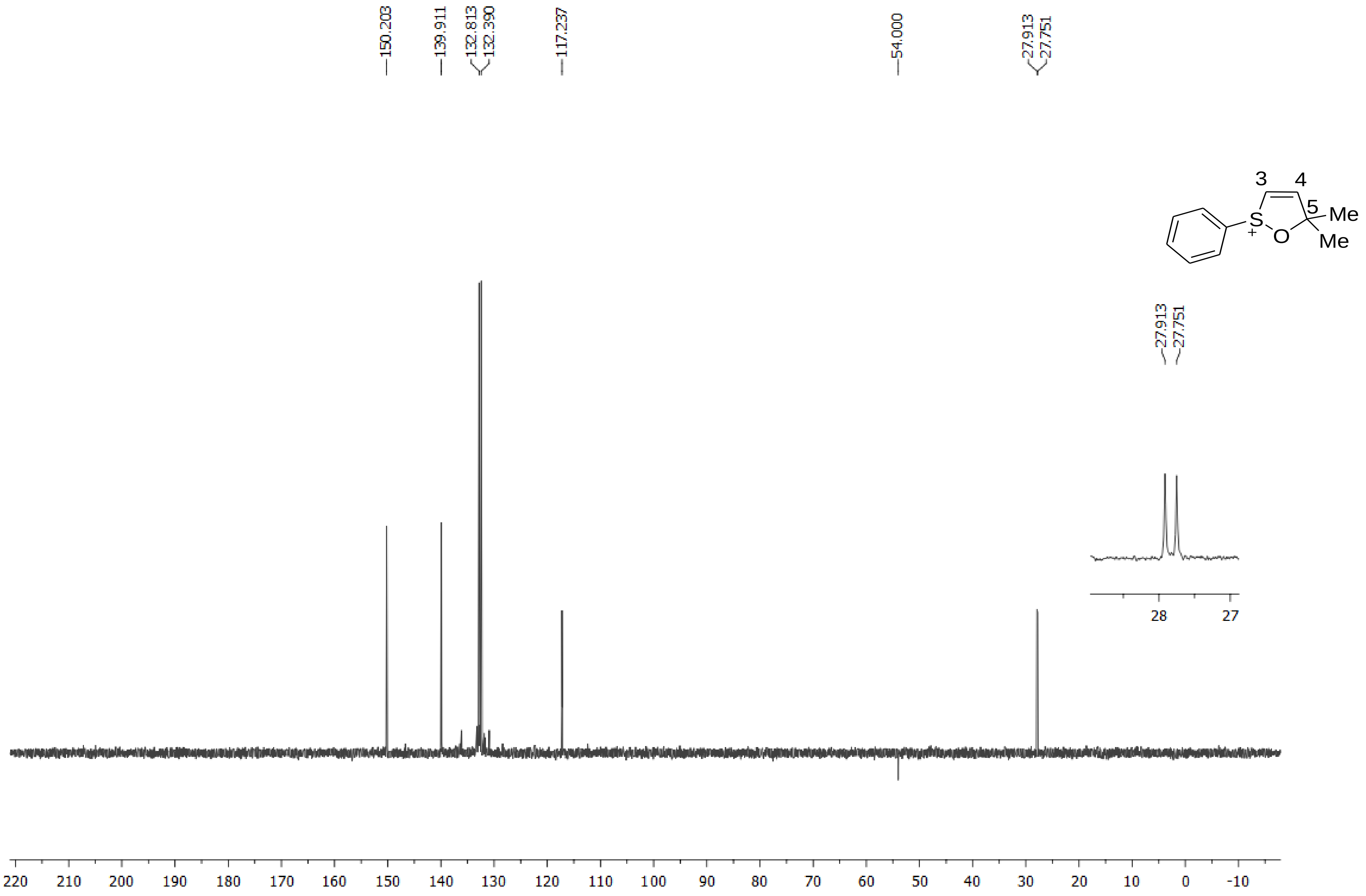

Fig. S45. DEPT NMR spectrum of the cation **Aa** (101 MHz, TfOH).

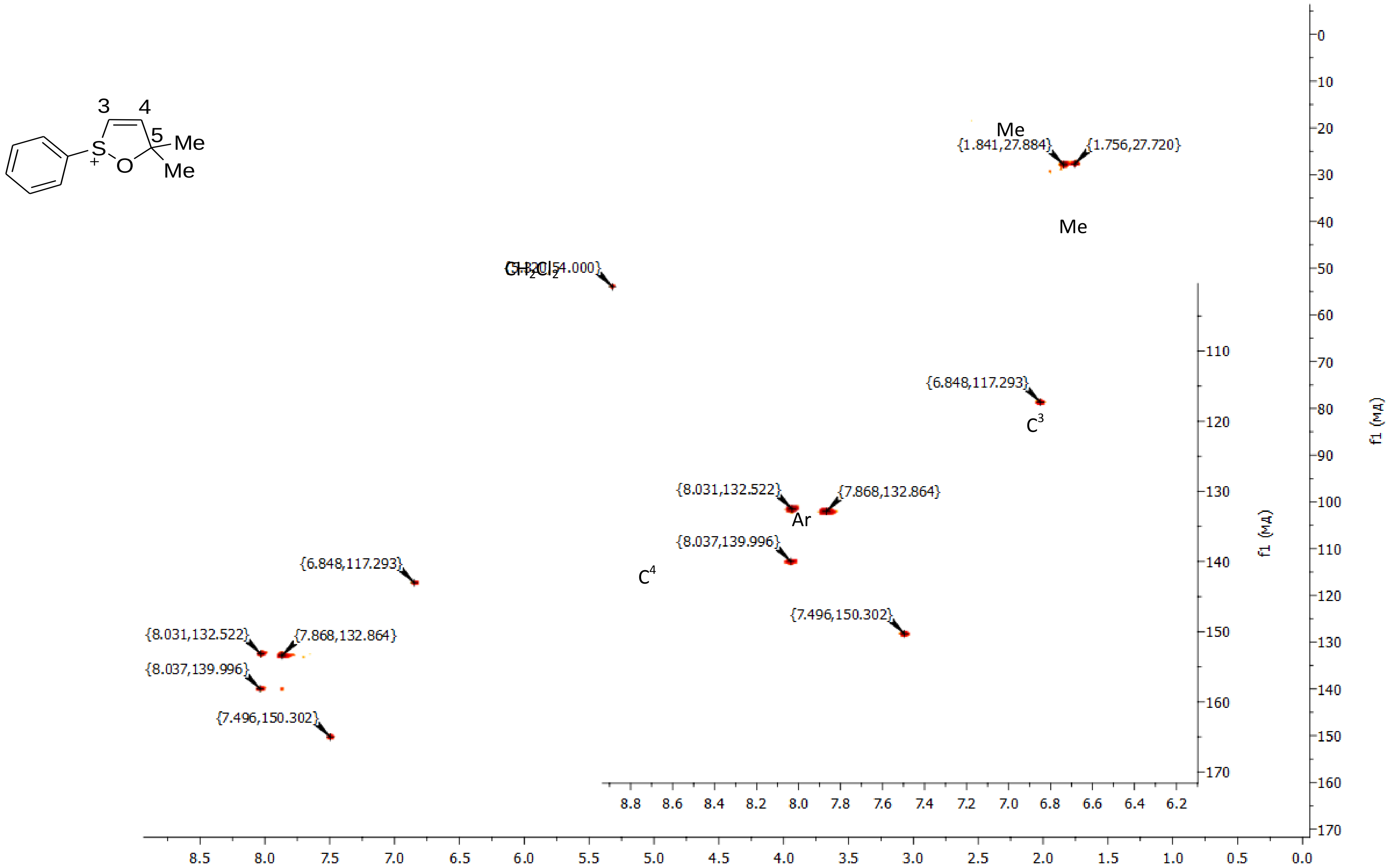

Fig. S46. DEPT NMR spectrum of the cation **Aa** (101 MHz, TfOH).

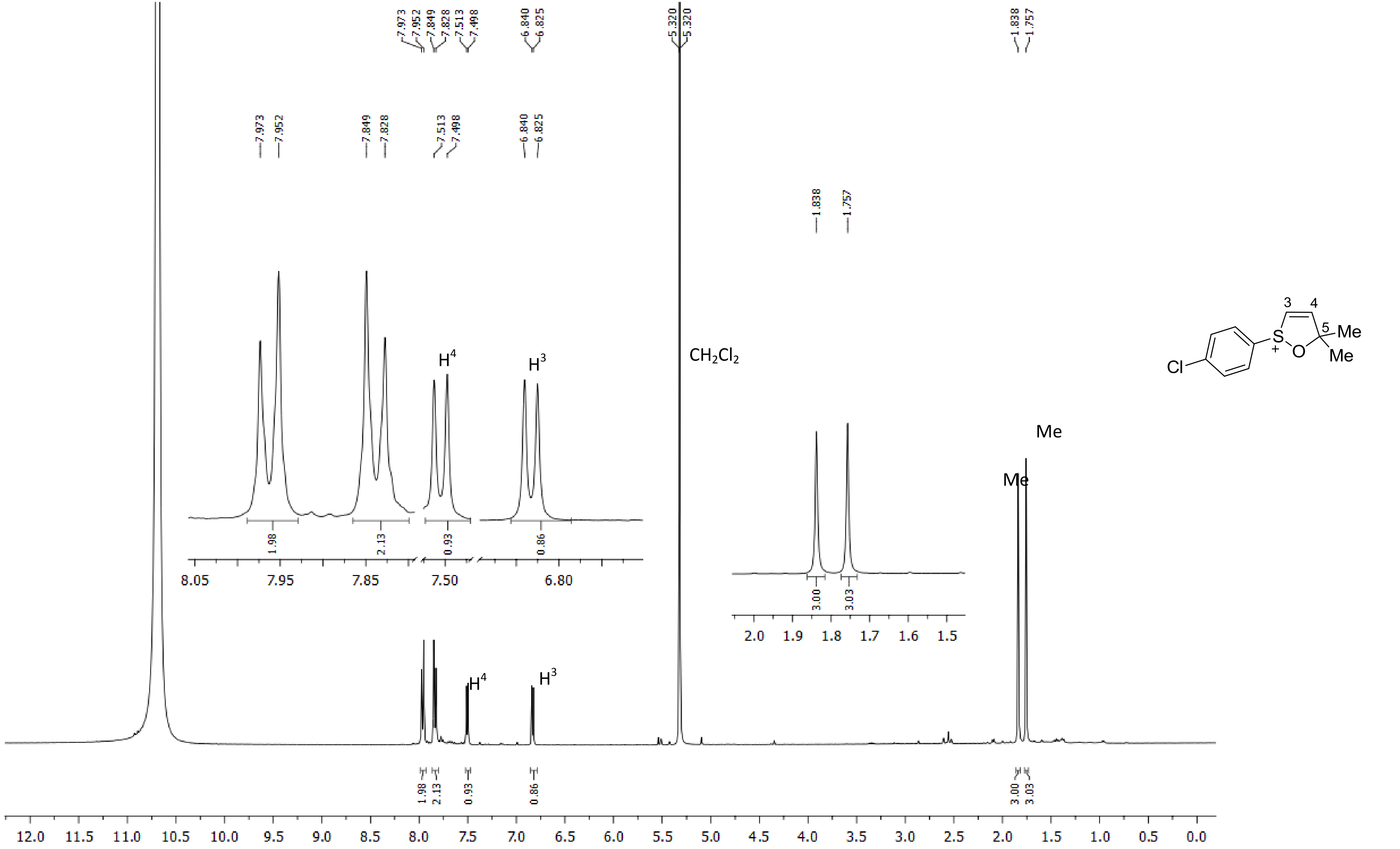

Fig. S47. ^{1}H NMR spectrum of the cation **Ab** (400 MHz, TfOH).

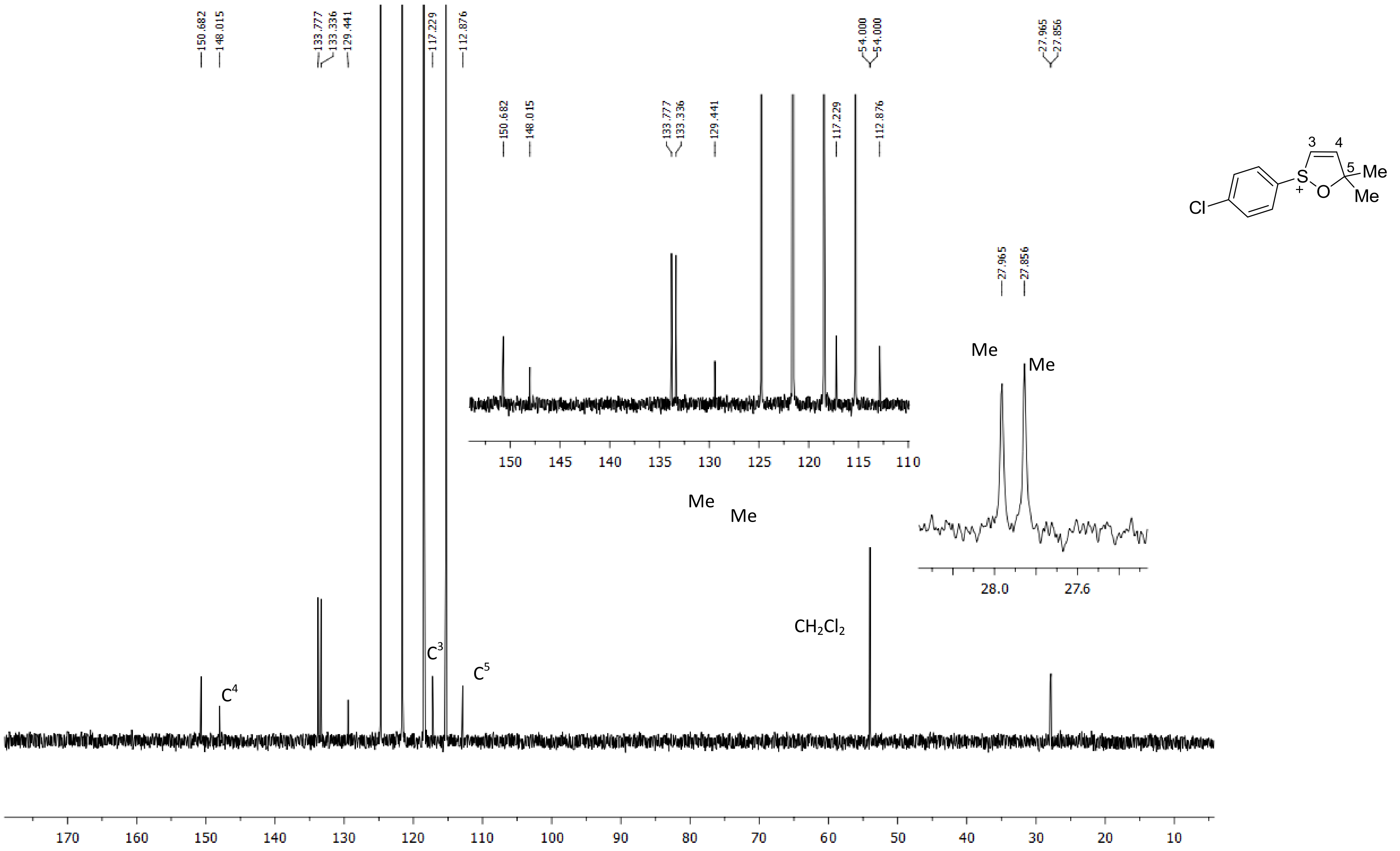

Fig. S48. ^{13}C NMR spectrum of the cation **Ab** (101 MHz, TfOH).

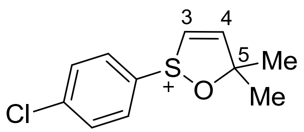

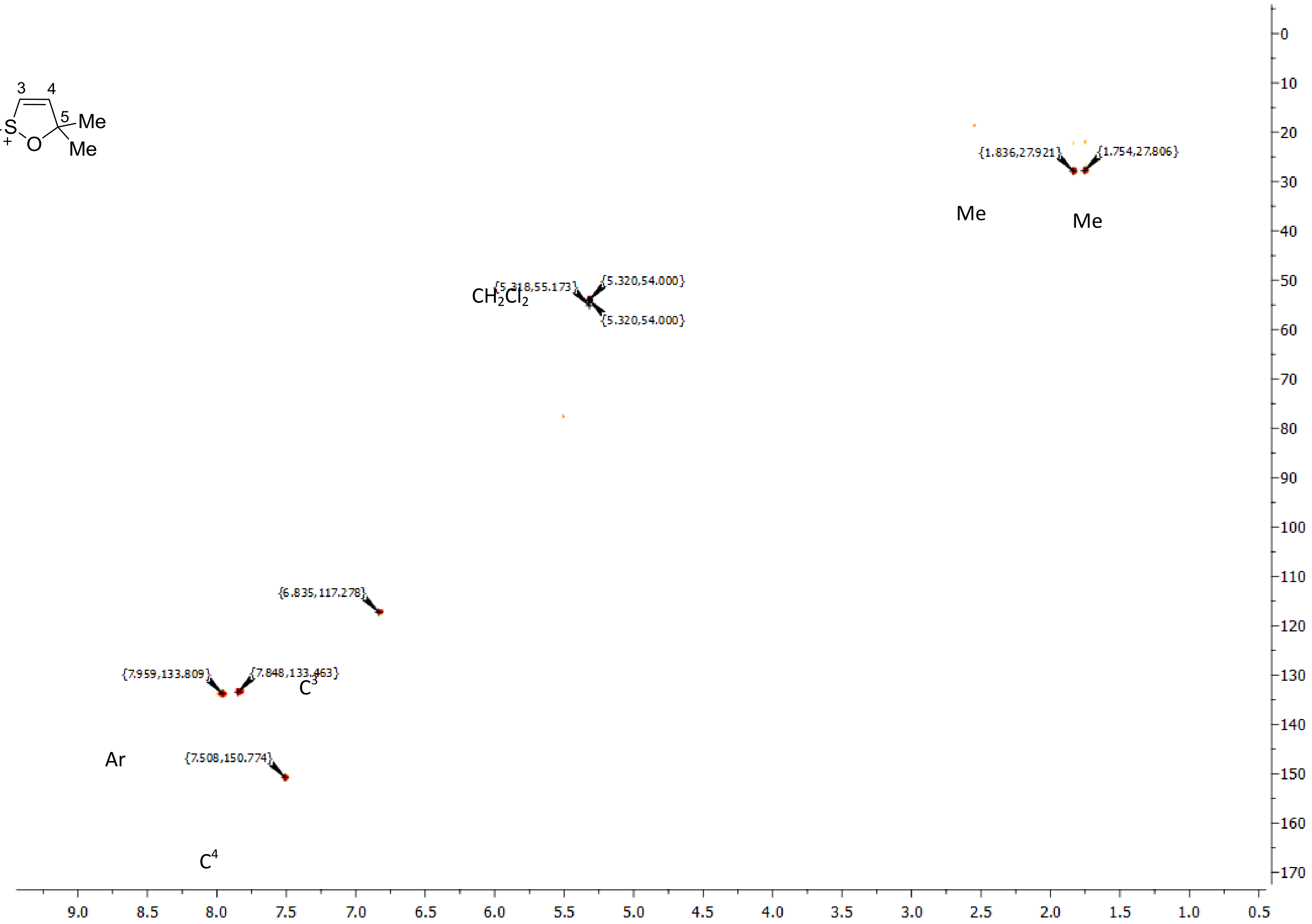

Fig. S49. HSQC NMR spectrum of the cation **Ab** (101 MHz, TfOH).

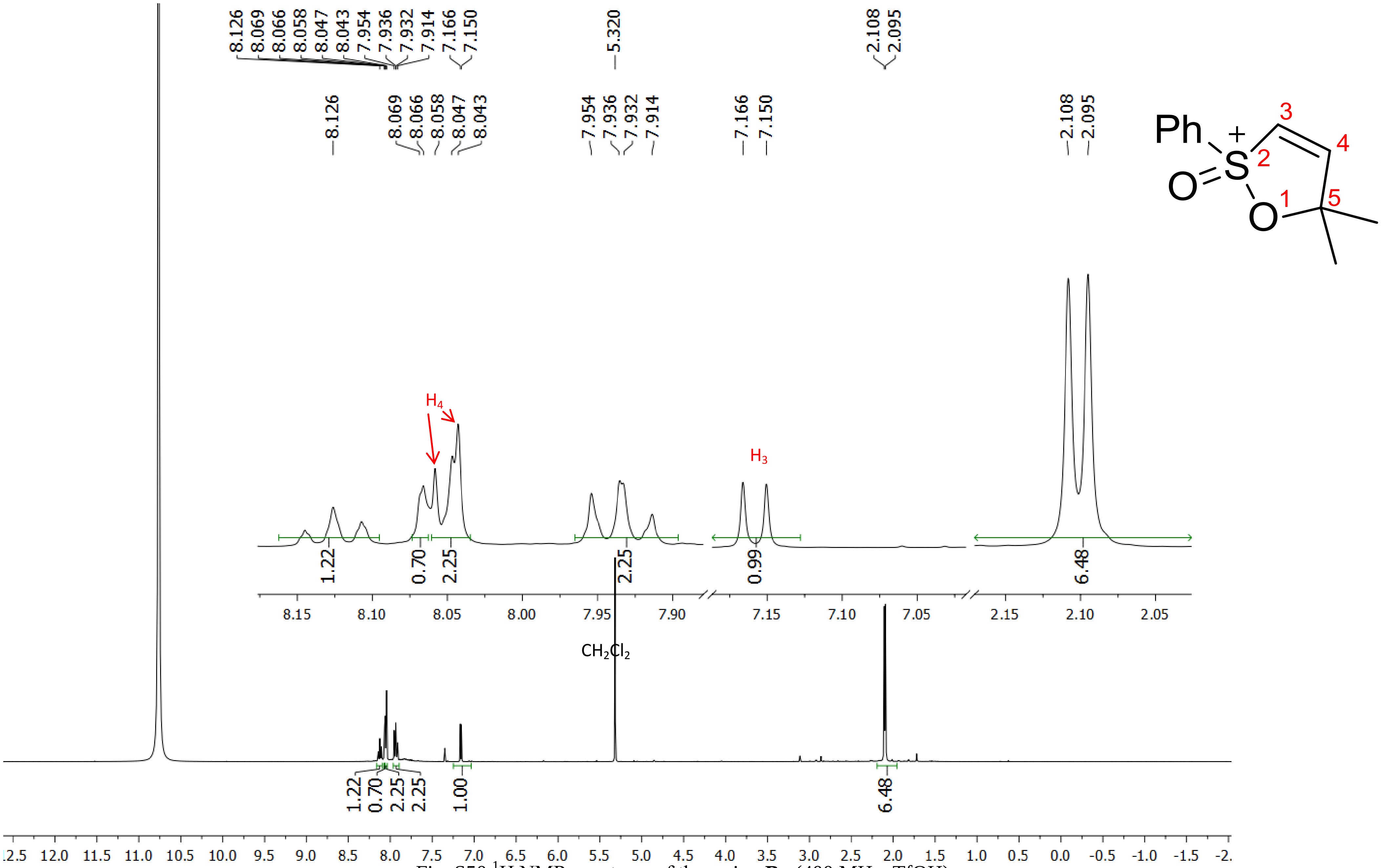

Fig. S50. ^{1}H NMR spectrum of the cation **Ba** (400 MHz, TfOH).

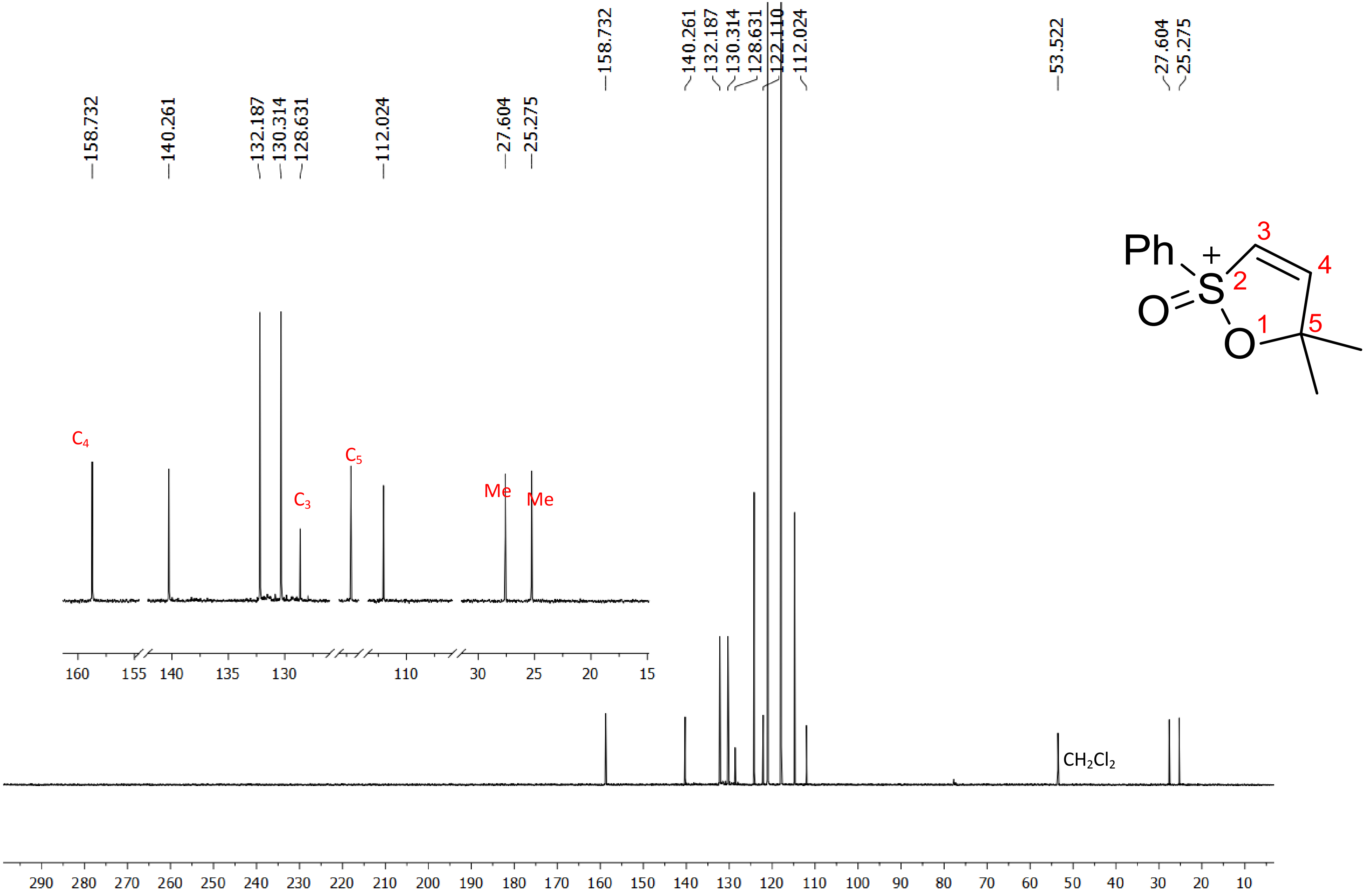

Fig. S51.^{13}C NMR spectrum of the cation **Ba** (100 MHz, TfOH).

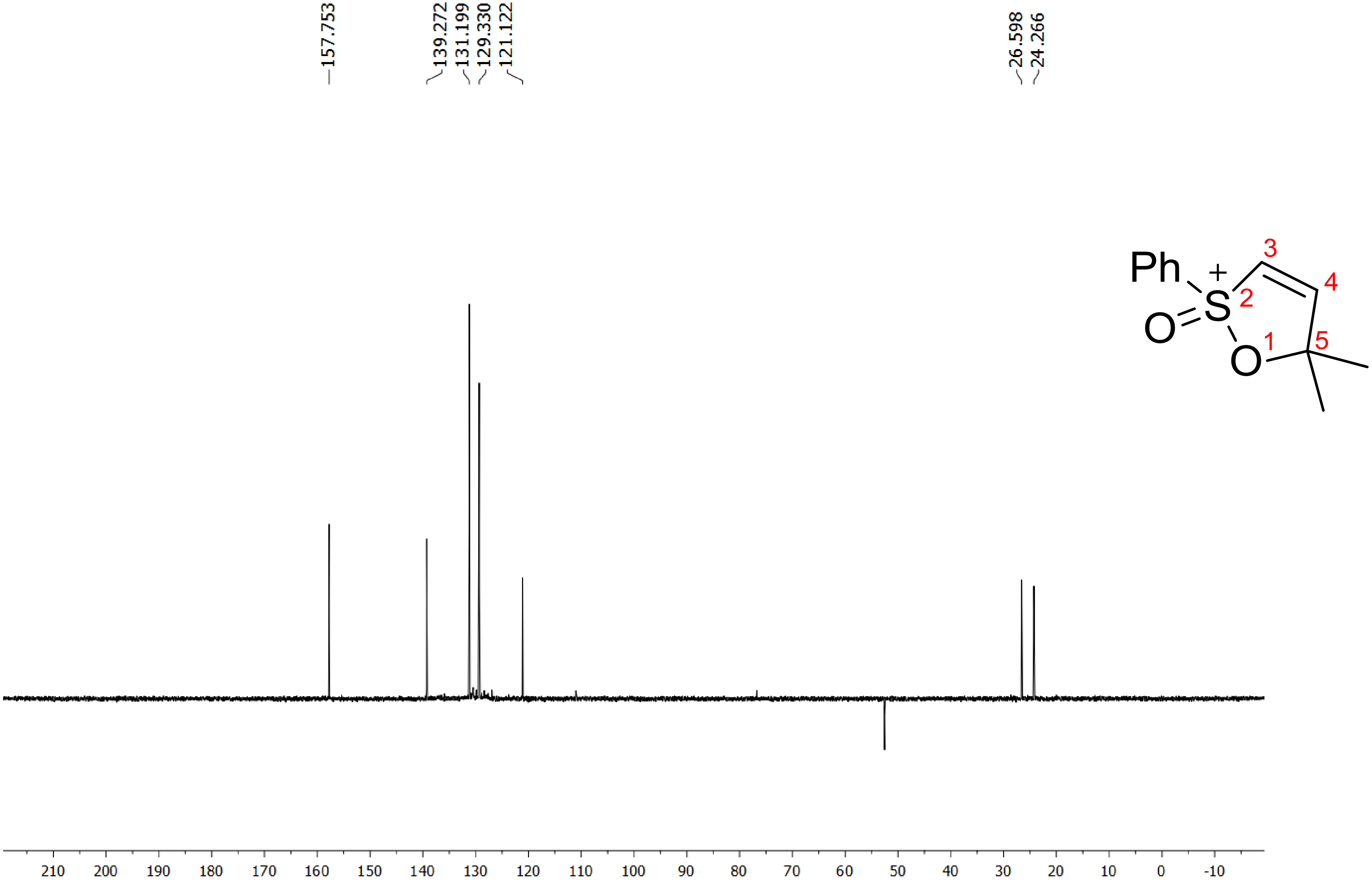

Fig. S52. DEPTNMR spectrum of the cation **Ba** (100 MHz, TfOH).

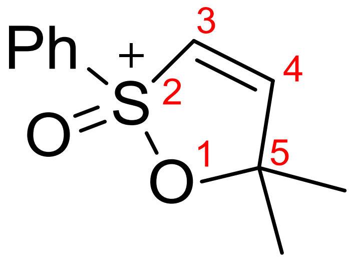

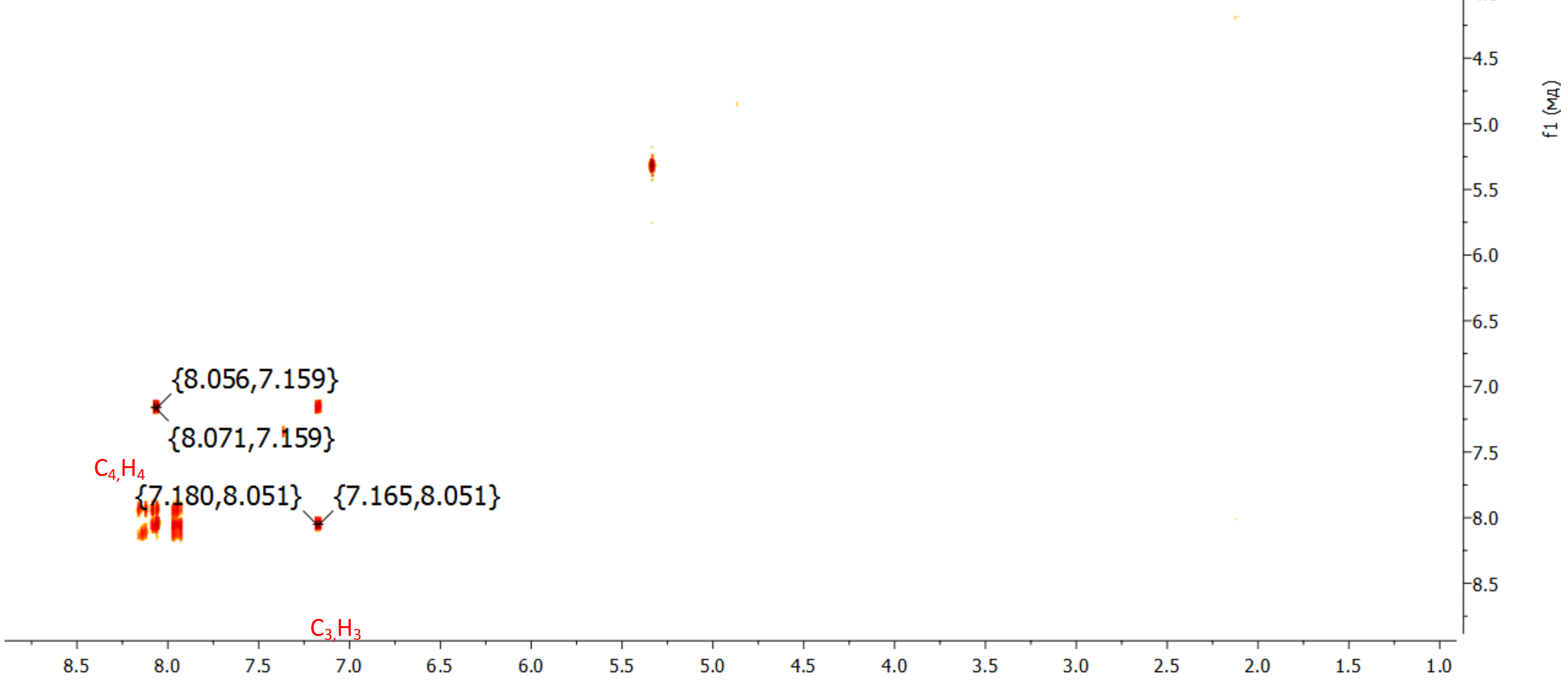

Fig. S53.COSY NMR spectrum of the cation **Ba** (100 MHz, TfOH).

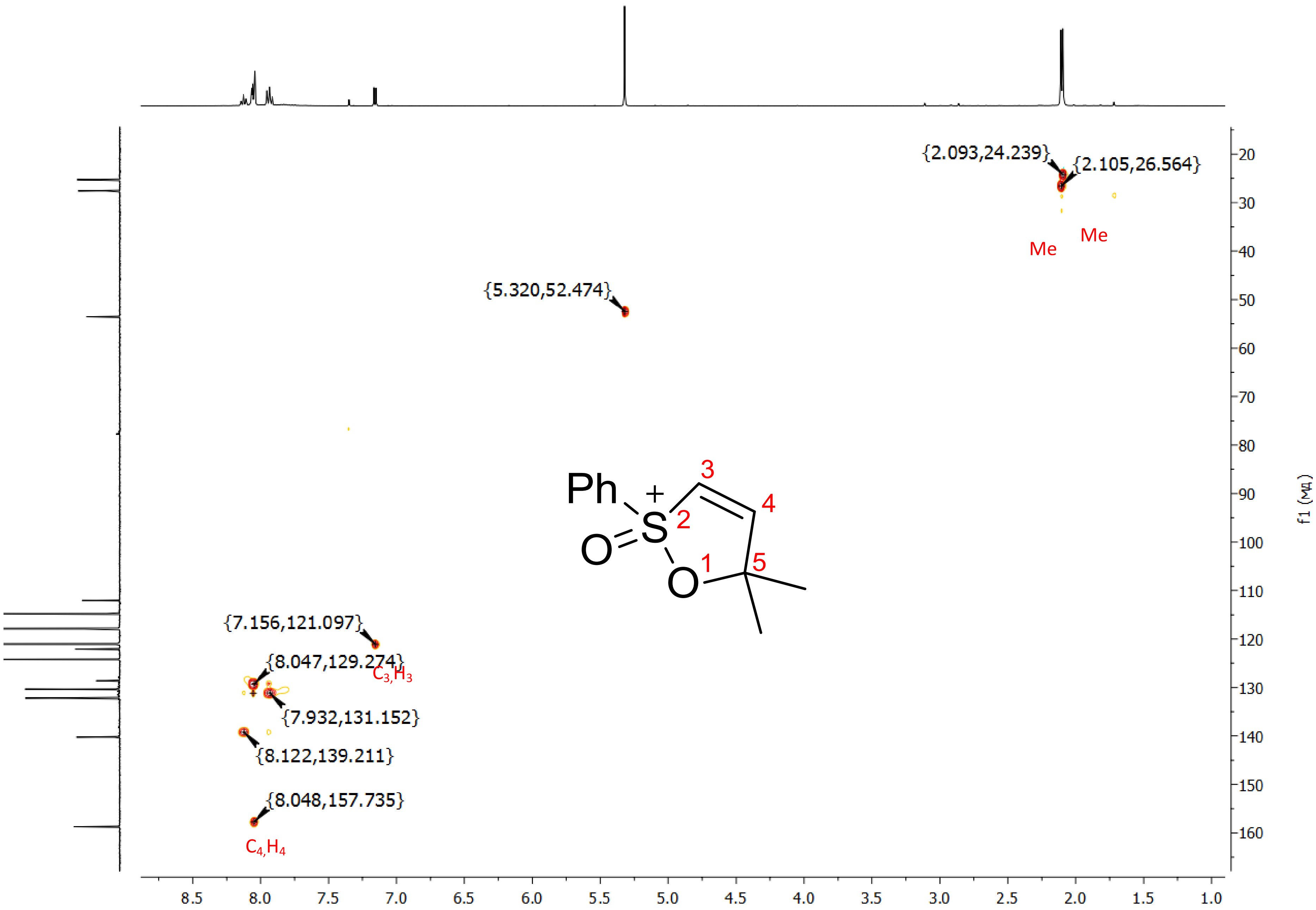

Fig. S54. HSQC NMR spectrum of the cation **Ba** (100 MHz, TfOH)

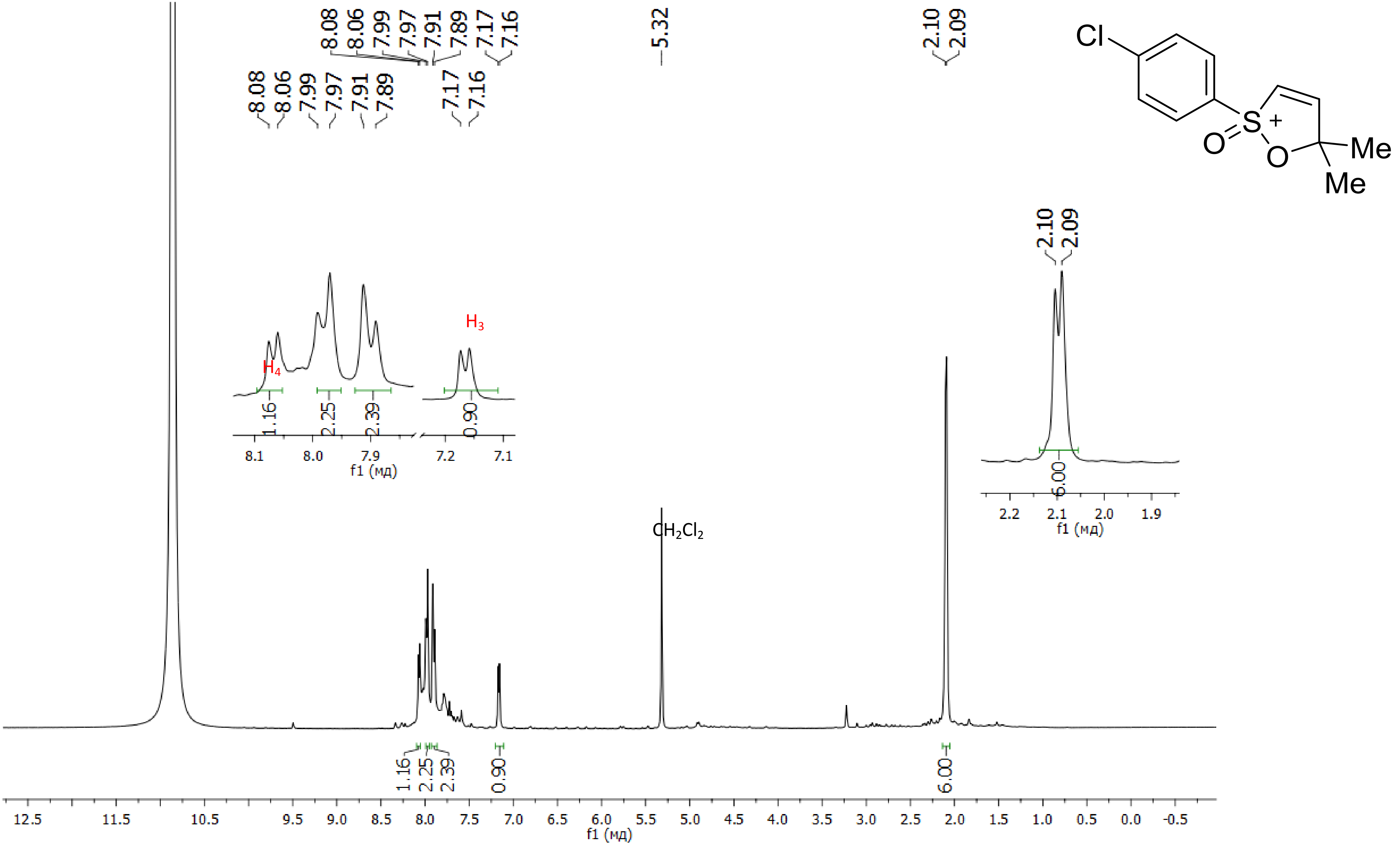

Fig. S55. ^{1}H NMR spectrum of the cation **Bb** (400 MHz,TfOH).

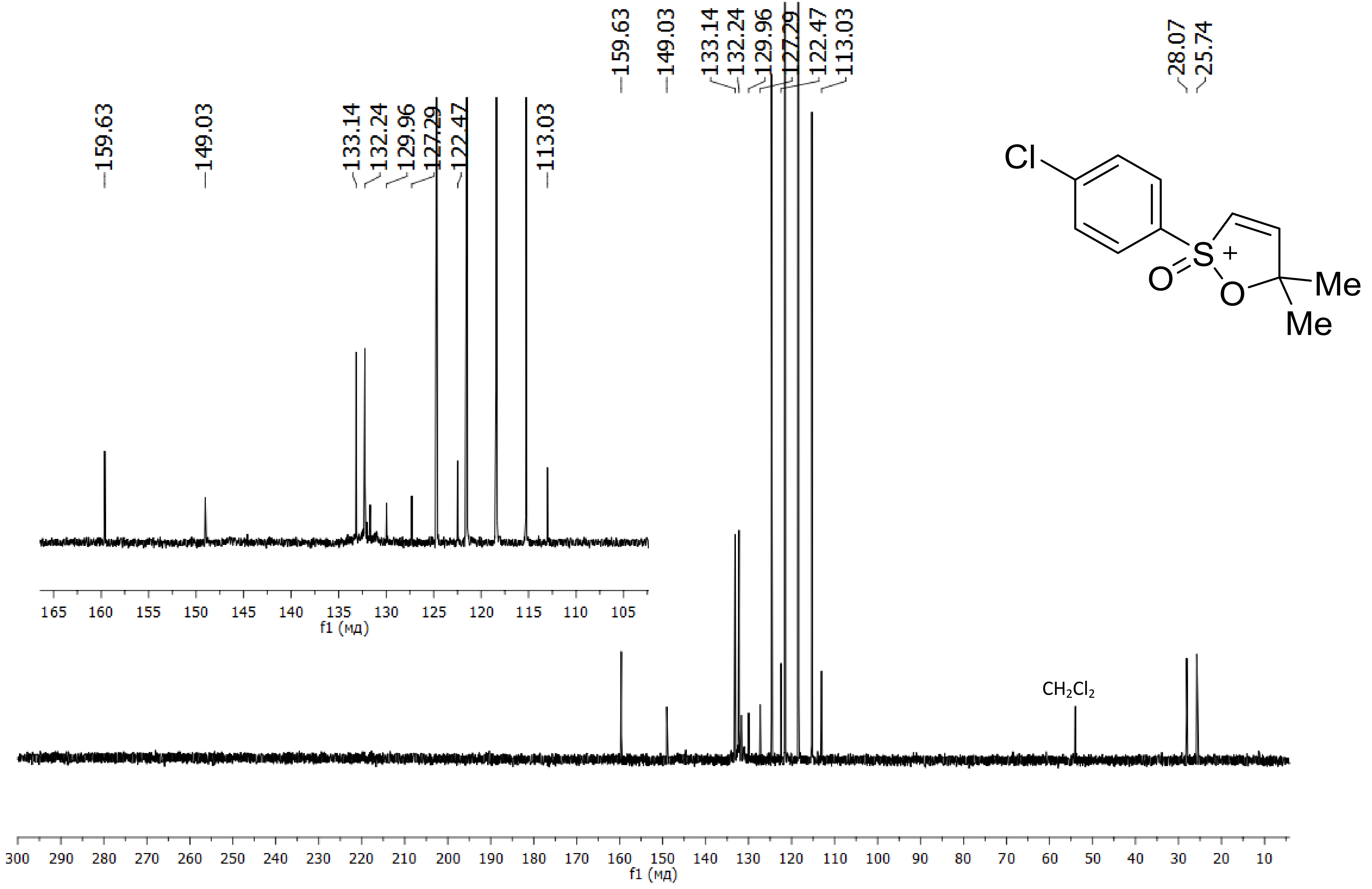

Fig. S56. ^{13}C NMR spectrum of the cation **Bb** (101 MHz, TfOH).

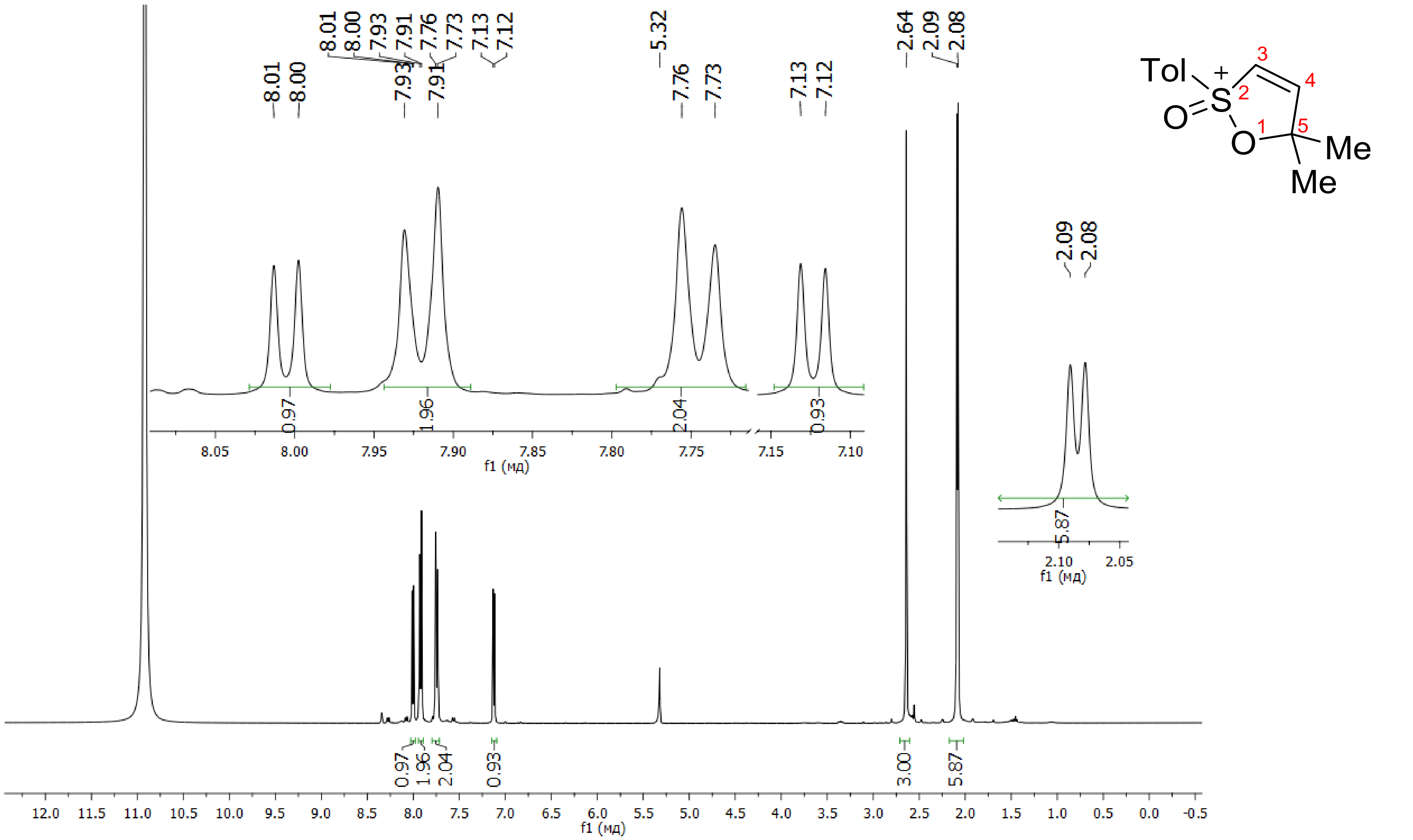

Fig. S57. ^{1}H NMR spectrum of the c cation **Bc** (400 MHz, TfOH).

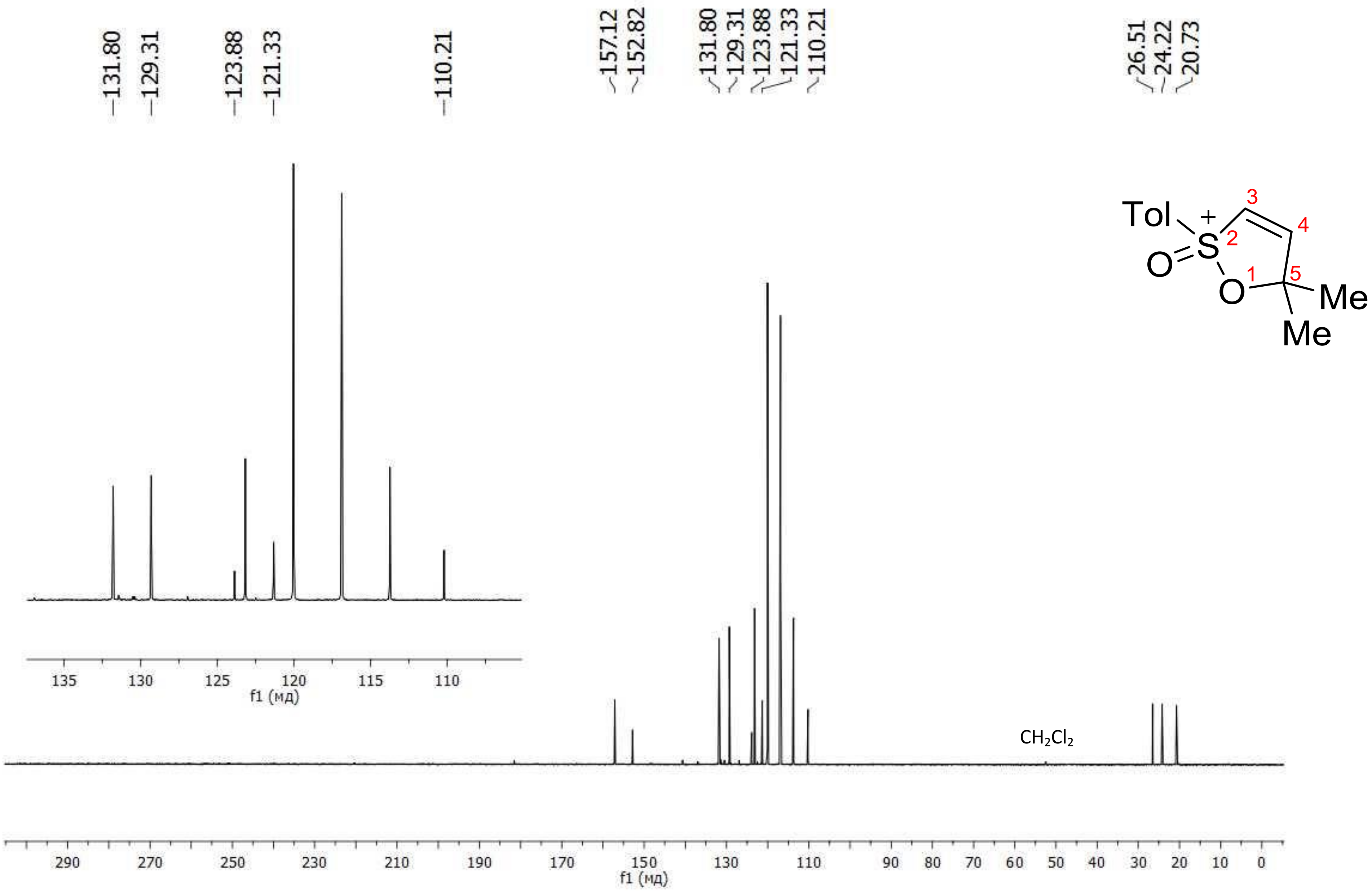

Fig. S58. ^{13}C NMR spectrum of the cation **Bc** (101 MHz, TfOH).

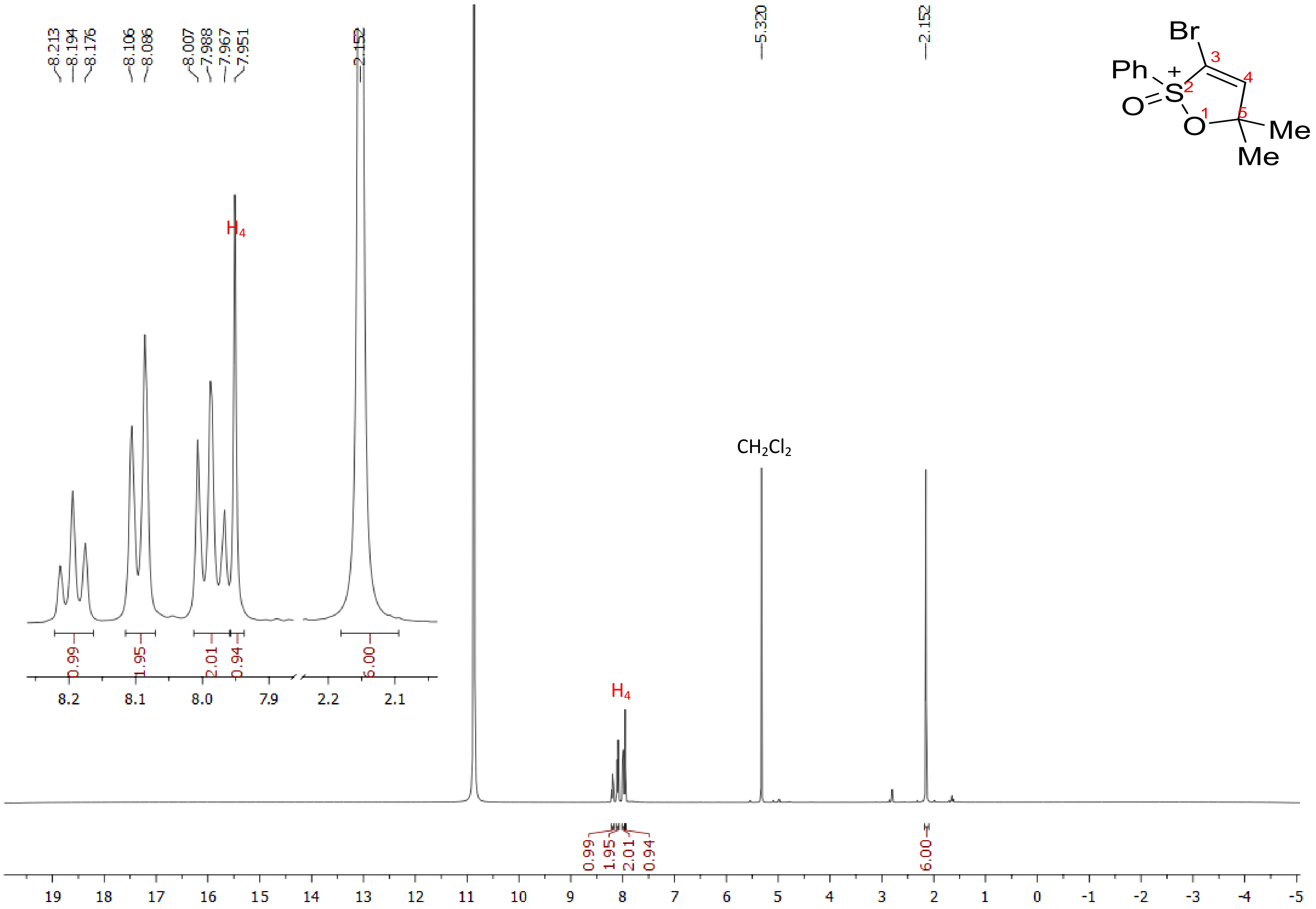

Fig. S59. ^{1}H NMR spectrum of the cation **Bd** (400 MHz,TfOH).

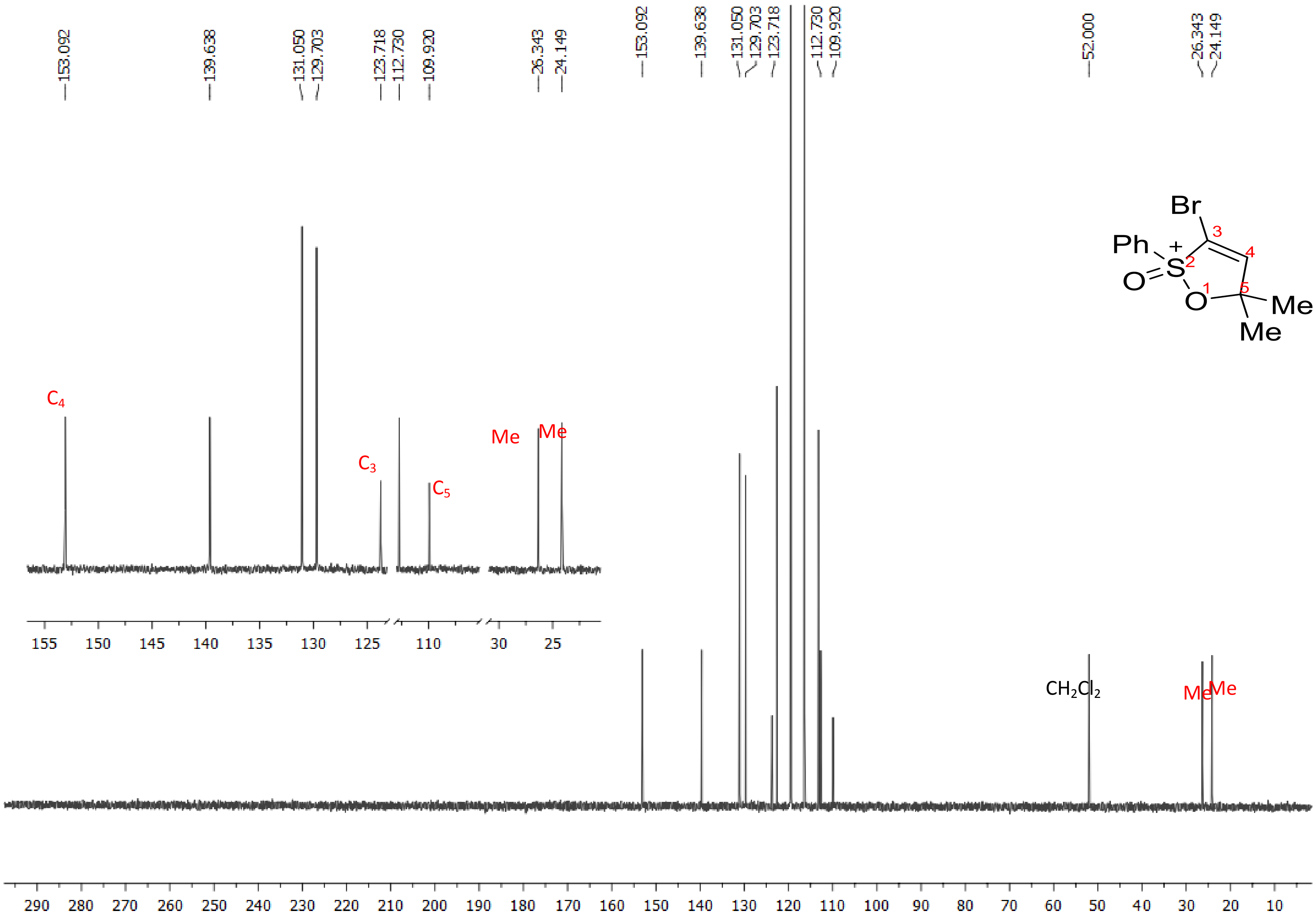

Fig. S60. ^{13}C NMR spectrum of the cation **Bd** (101 MHz,TfOH).

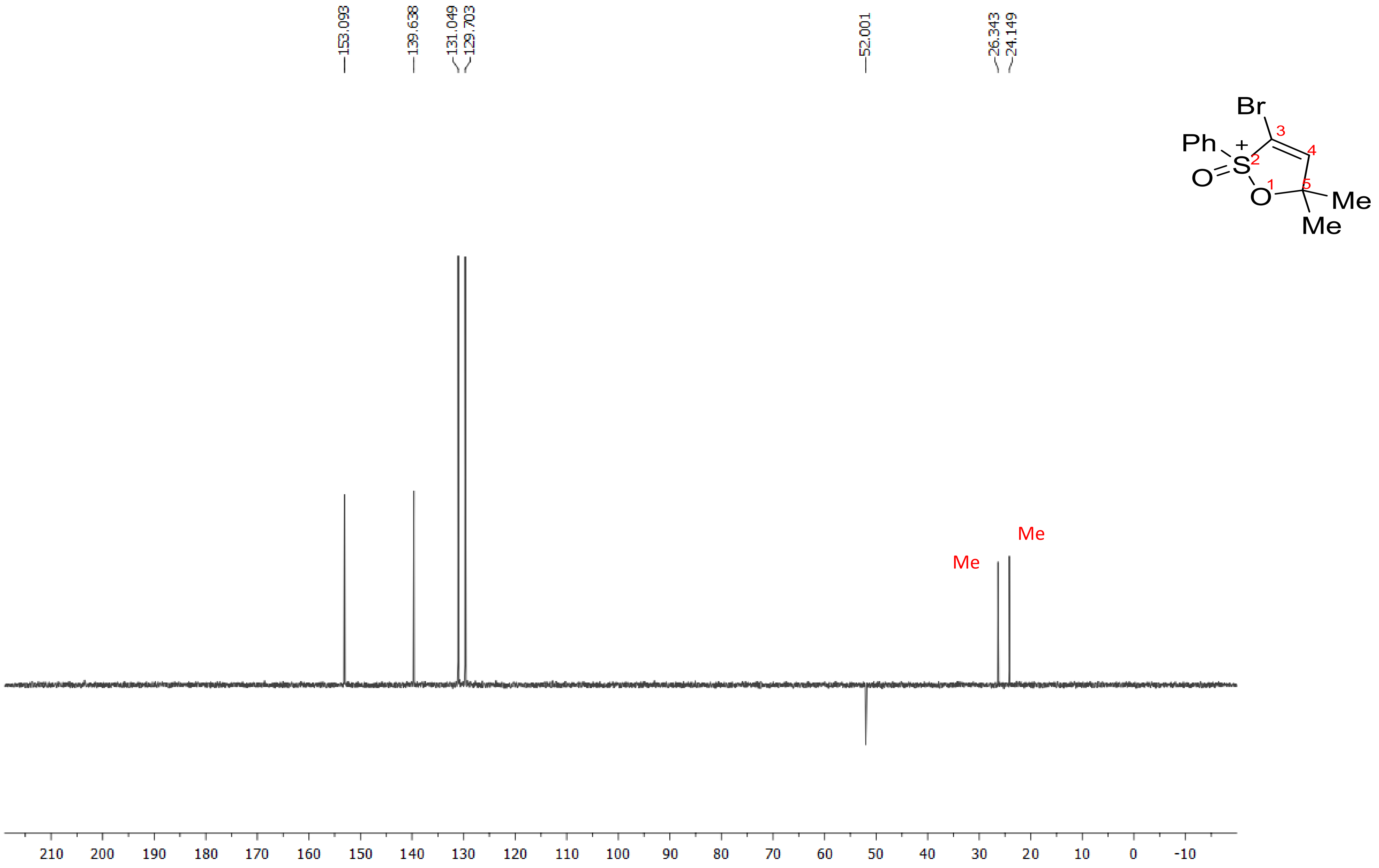

Fig. S61. DEPT NMR spectrum of the cation **Bd** (101MHz,TfOH).

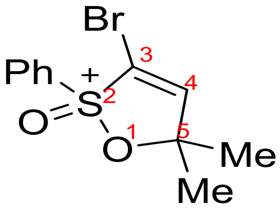

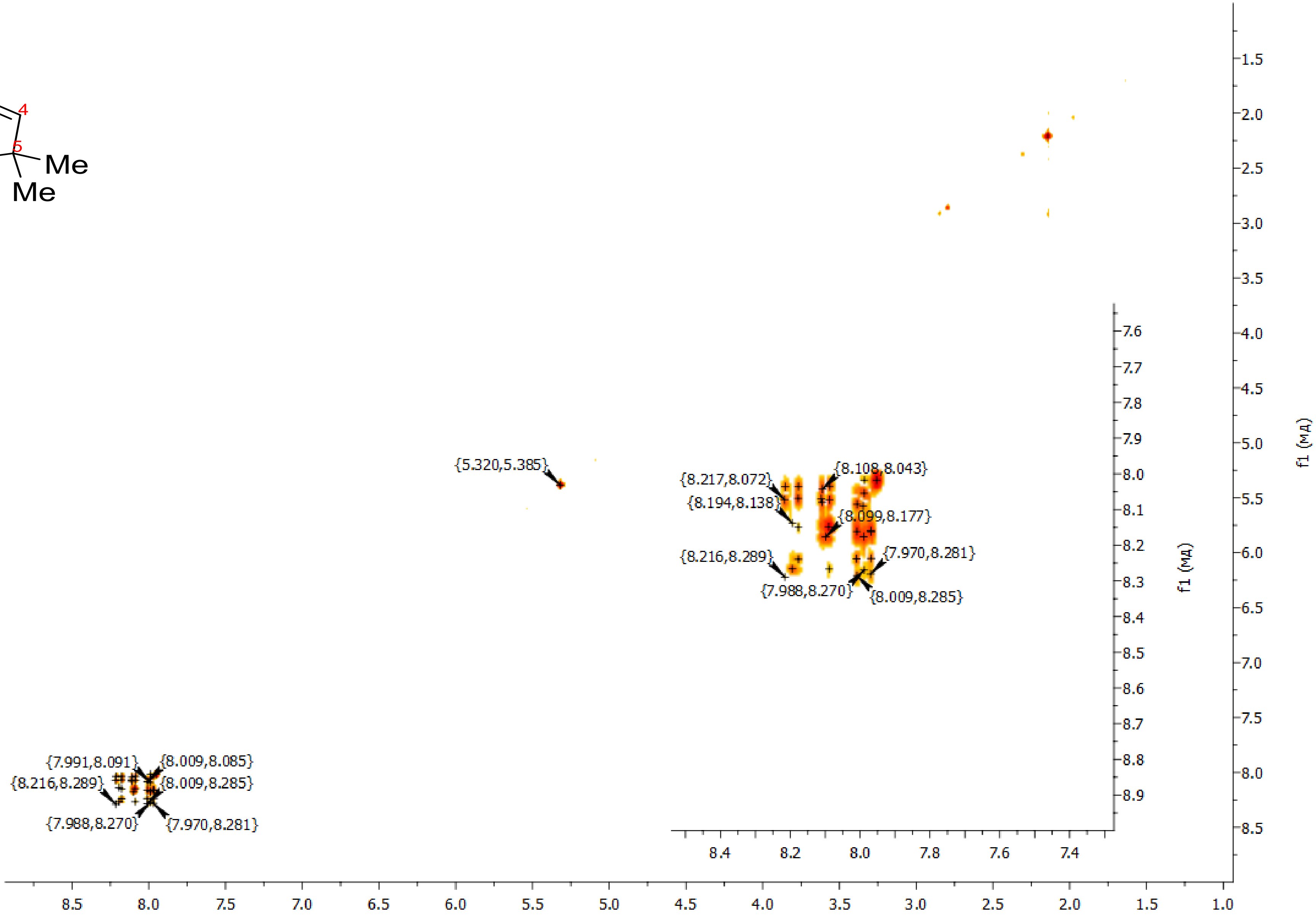

Fig. S62. COSY NMR spectrum of the cation **Bd** (101 MHz, TfOH).

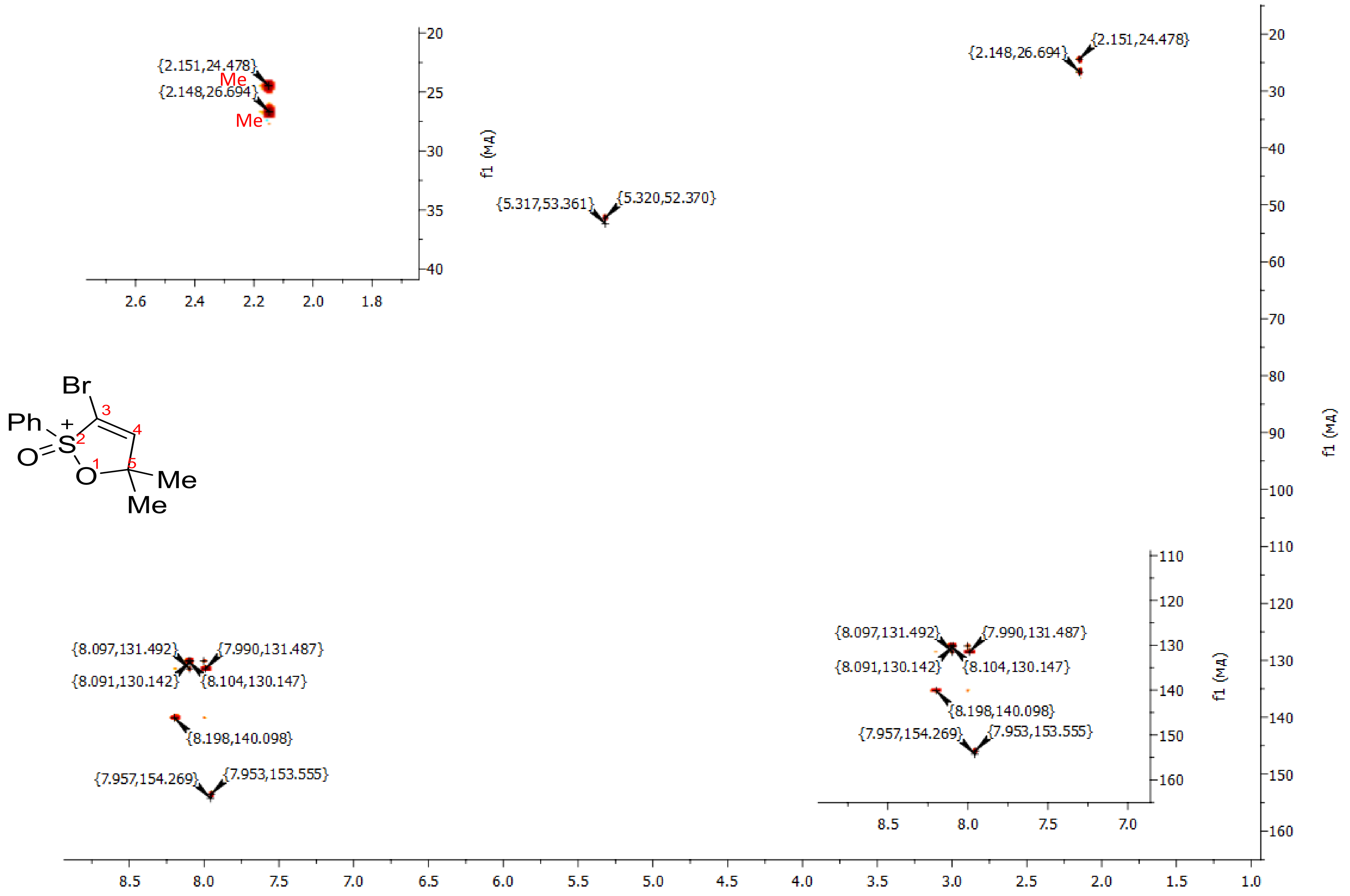

Fig. S63. HSQC NMR spectrum of the cation **Bd** (101 MHz,TfOH).

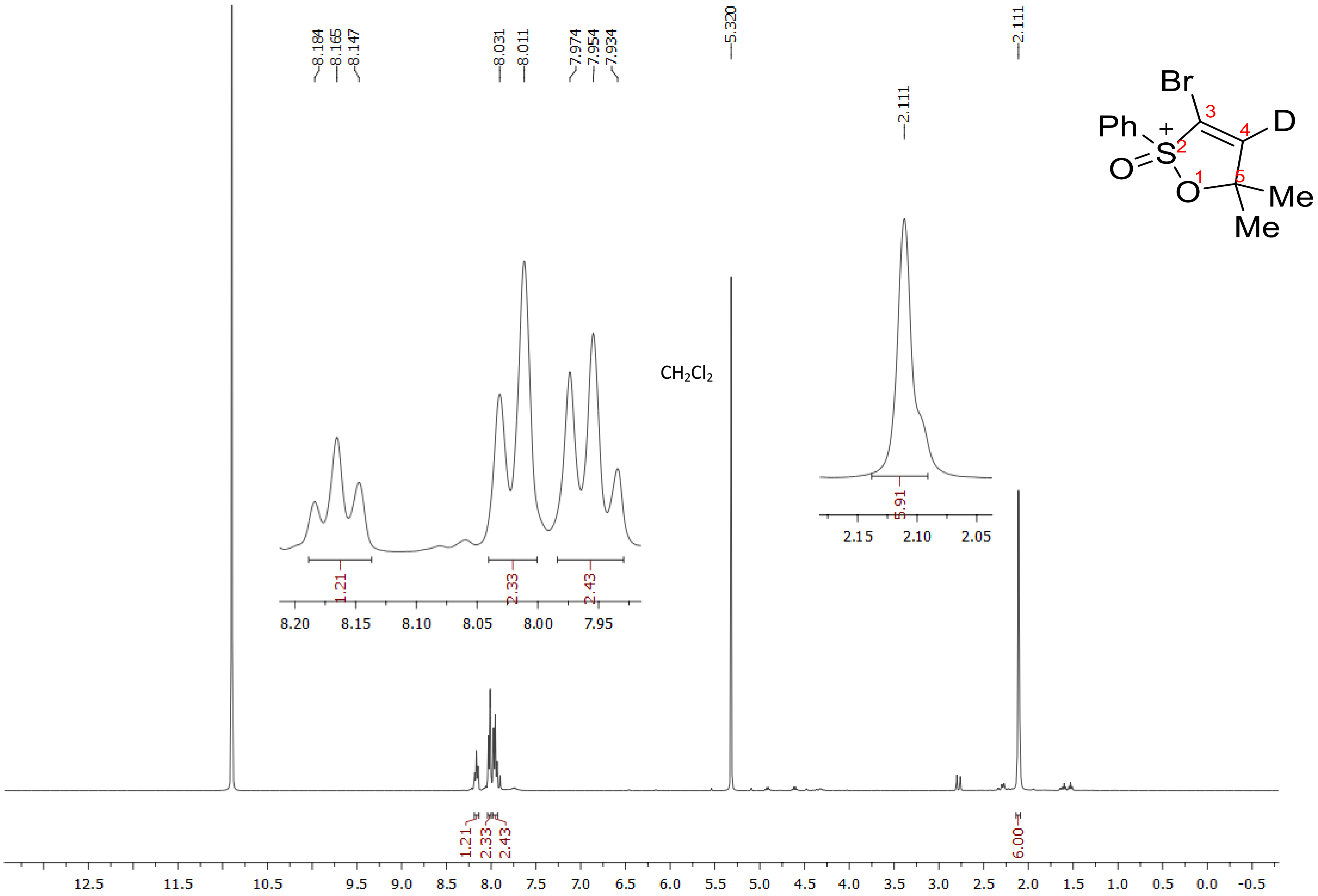

Fig. S 64. ^{1}H NMR spectrum of the cation **Bd**-*d* (400 MHz, D_2SO_4).

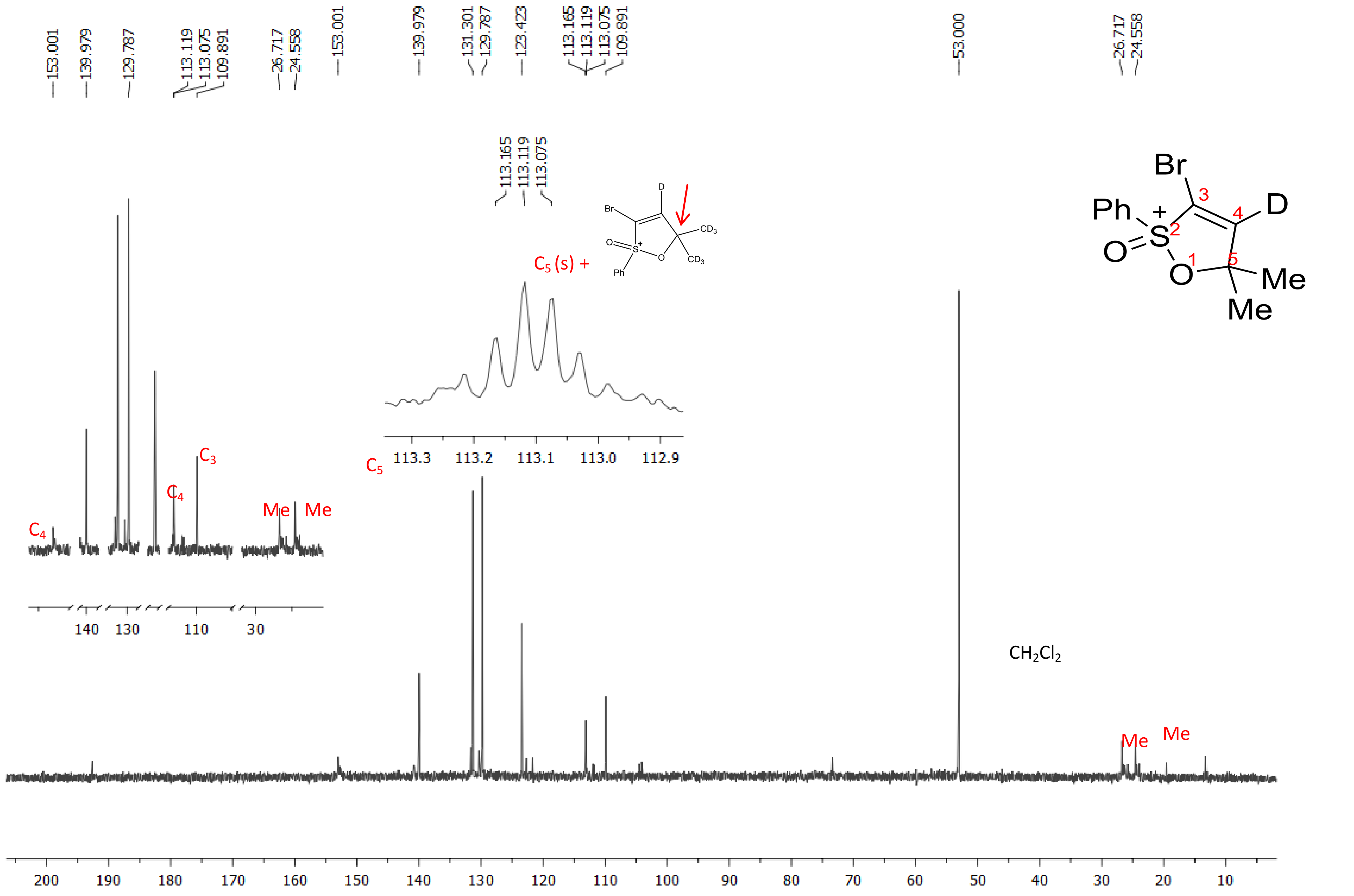

Fig. S65. ^{13}C NMR spectrum of the cation **Bd**-*d*(101 MHz, D_2SO_4).

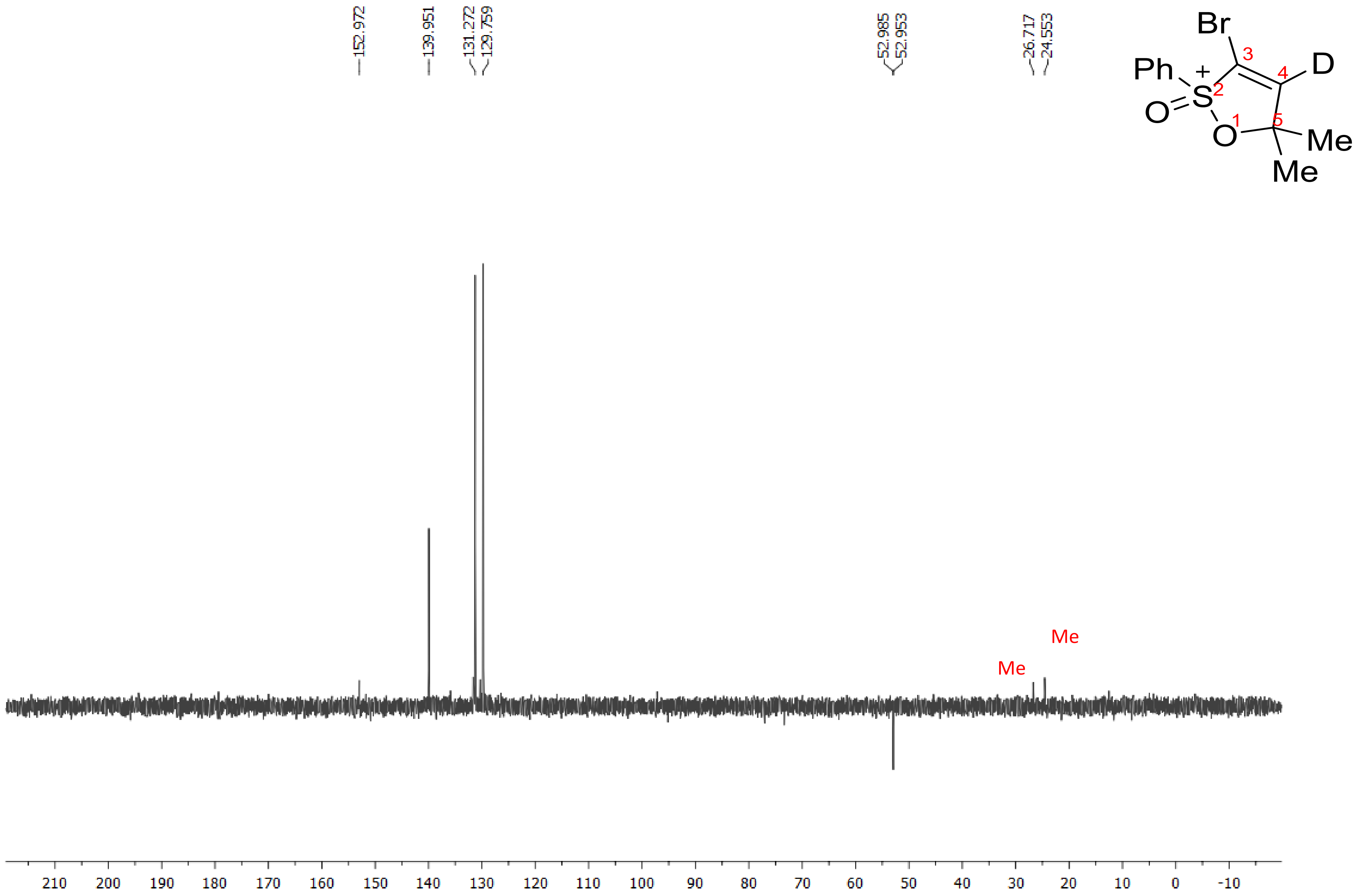

Fig. S66. DEPT NMR spectrum of the cation **Bd**-*d* (101 MHz,D_2SO_4).

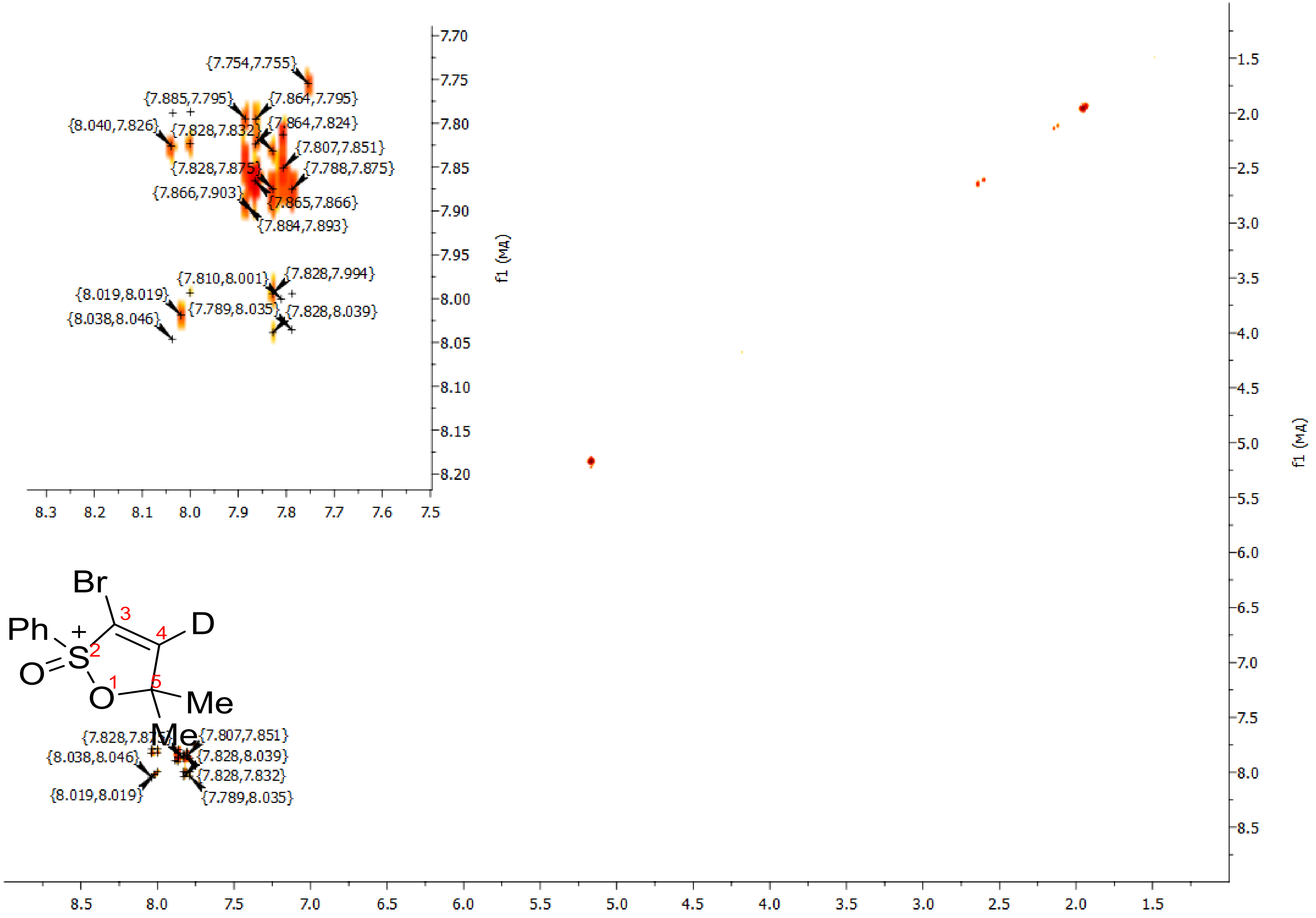

Fig. S67. COSY NMR spectrum of the cation **Bd**-*d* (101 MHz,D_2SO_4).

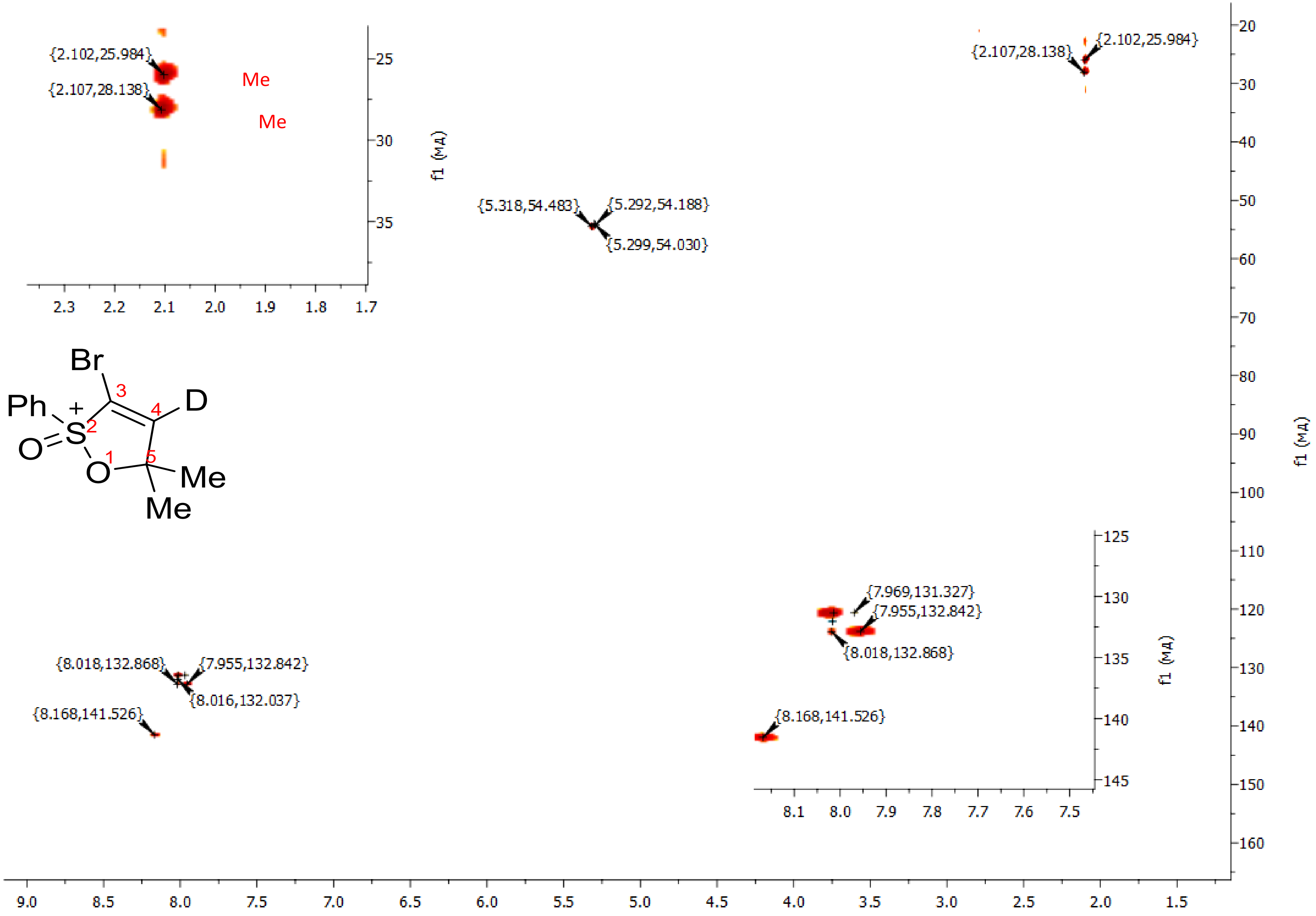

Fig. S68. HSQC NMR spectrum of the cation **Bd-*d*** (101 MHz, D_2SO_4).

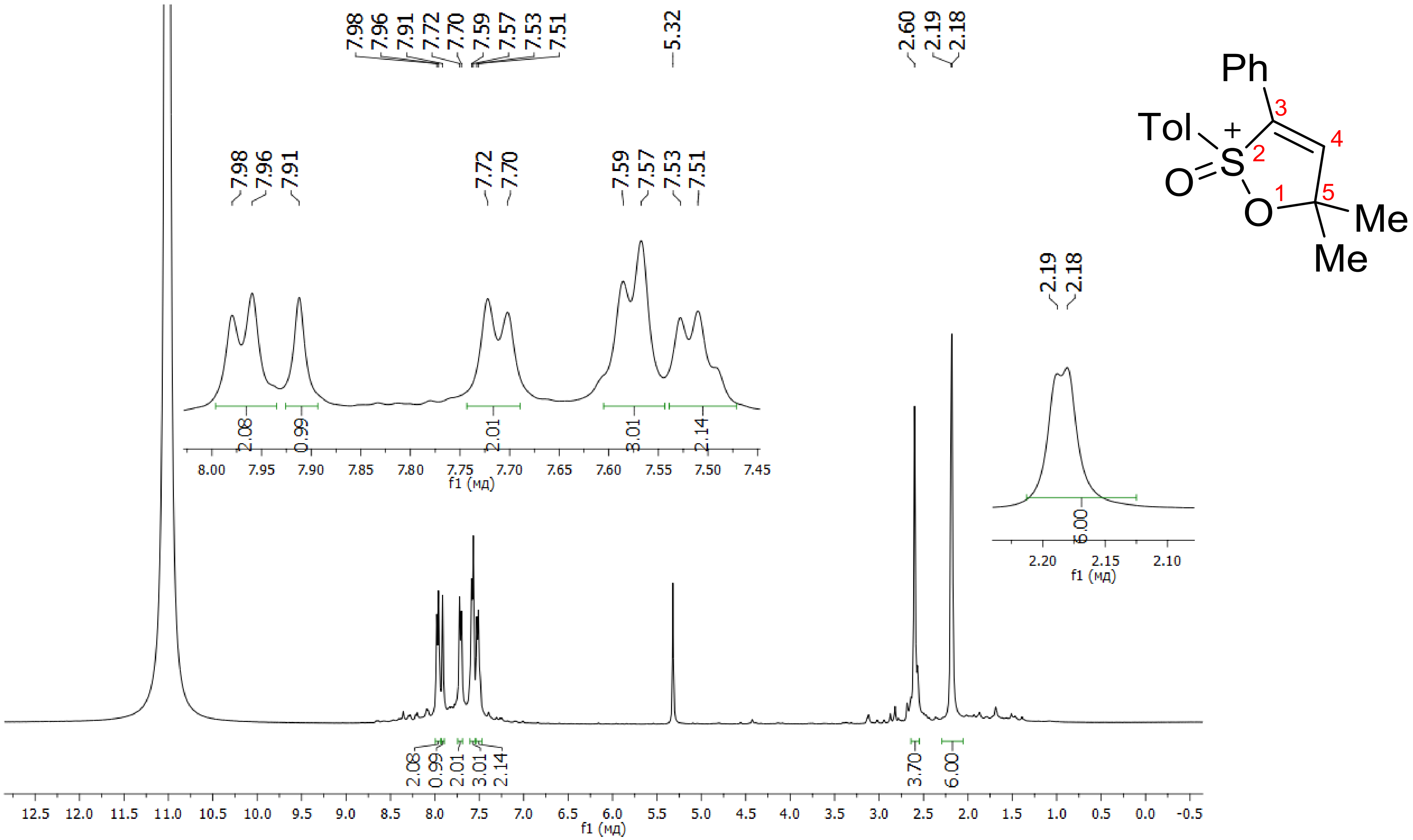

Fig. S69. ^{1}H NMR spectrum of the cation **Be** (400 MHz, TfOH).

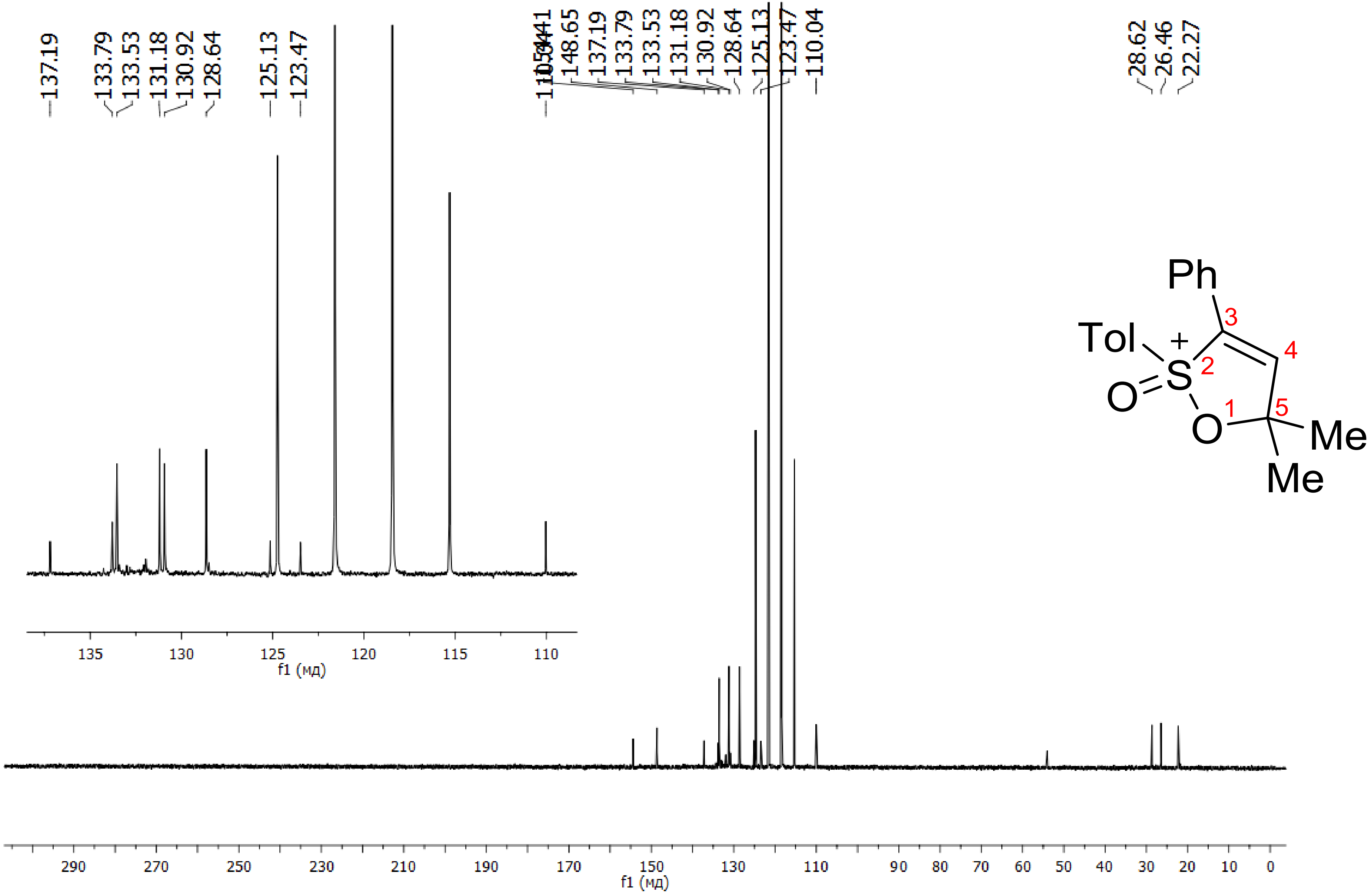

Fig. S70. ^{13}C NMR spectrum of the c cation **Be**(101 MHz, TfOH).

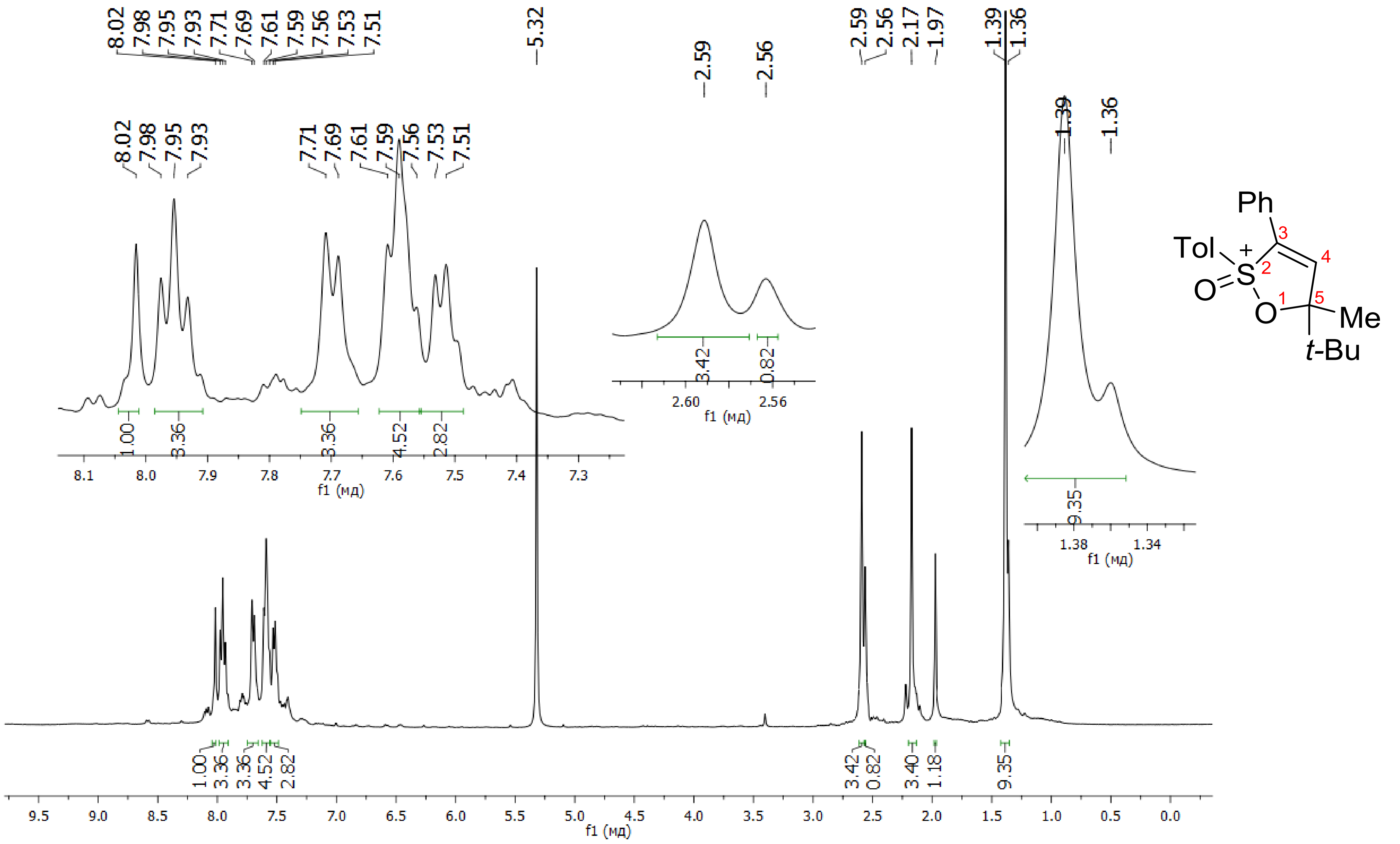

Fig. S71. ^{1}H NMR spectrum of the cation **Bf** with admixture of minor isomer (400 MHz, TfOH).

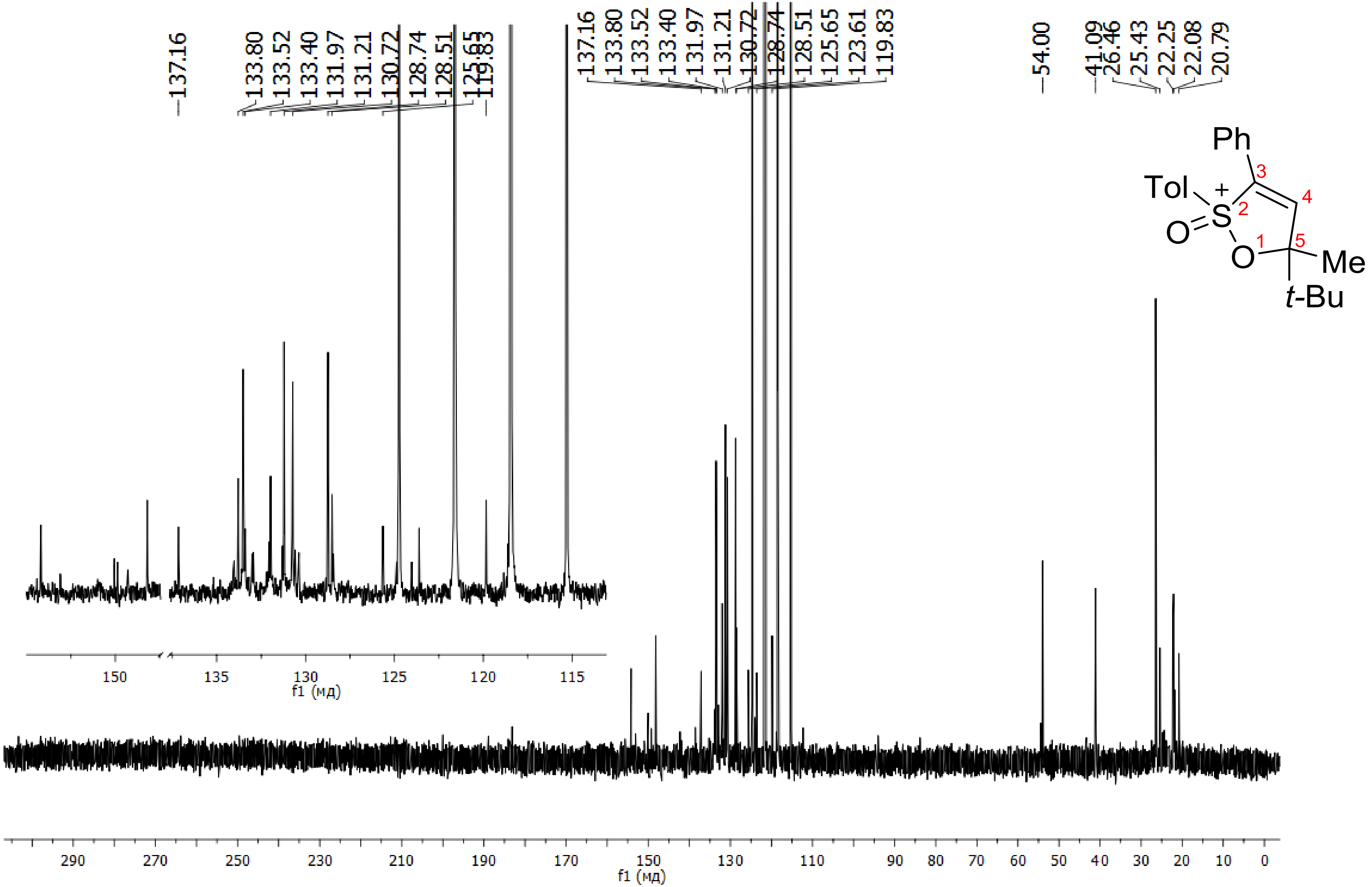

Fig. S72. ^{13}C NMR spectrum of the cation **Bf** with admixture of minor isomer (101 MHz, TfOH).

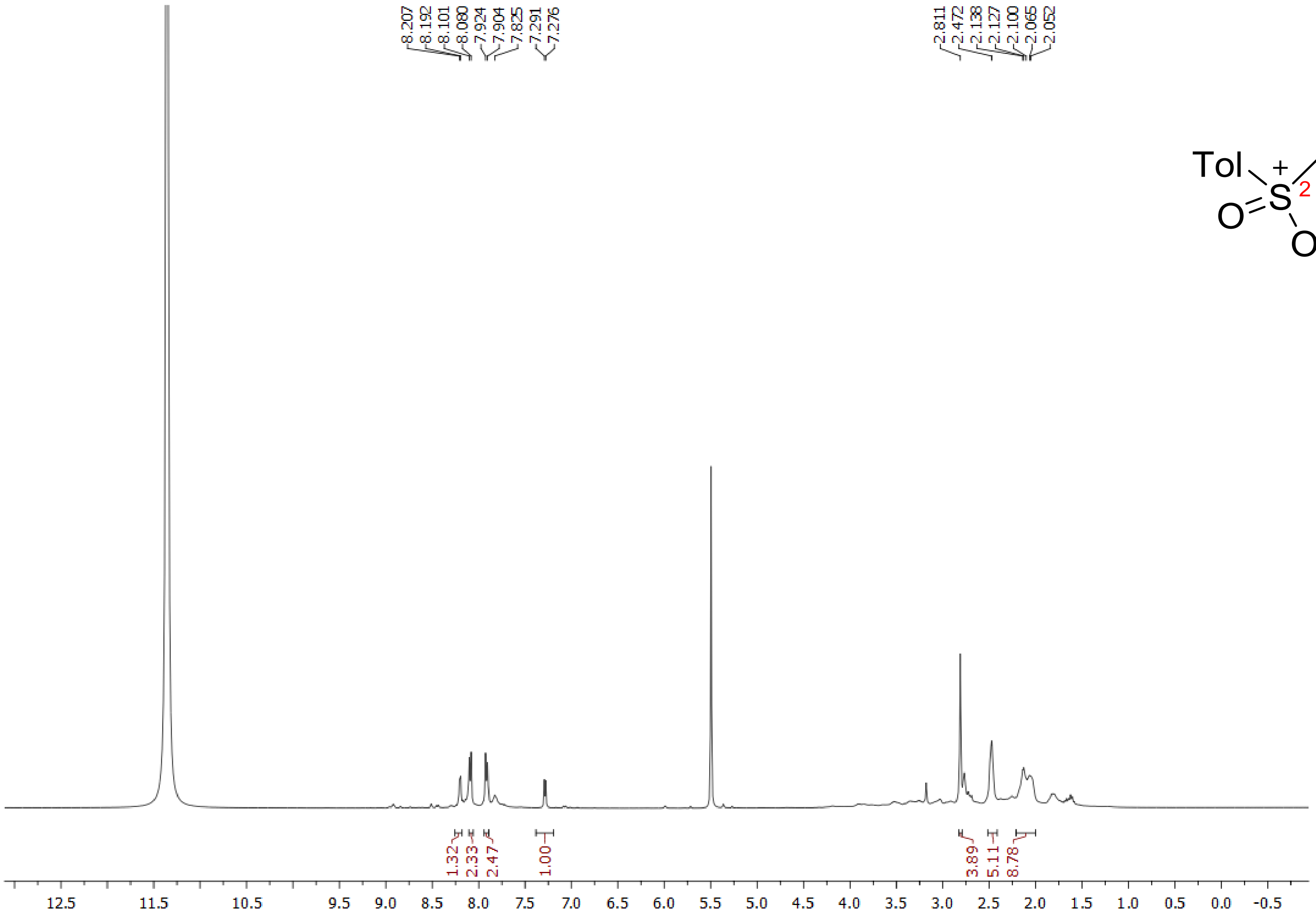

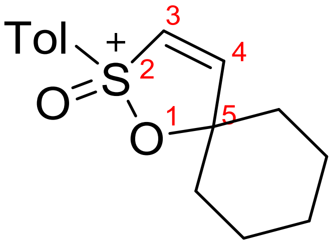

Fig. S73. ^{1}H NMR spectrum of the cation **Bg** (400 MHz, TfOH).

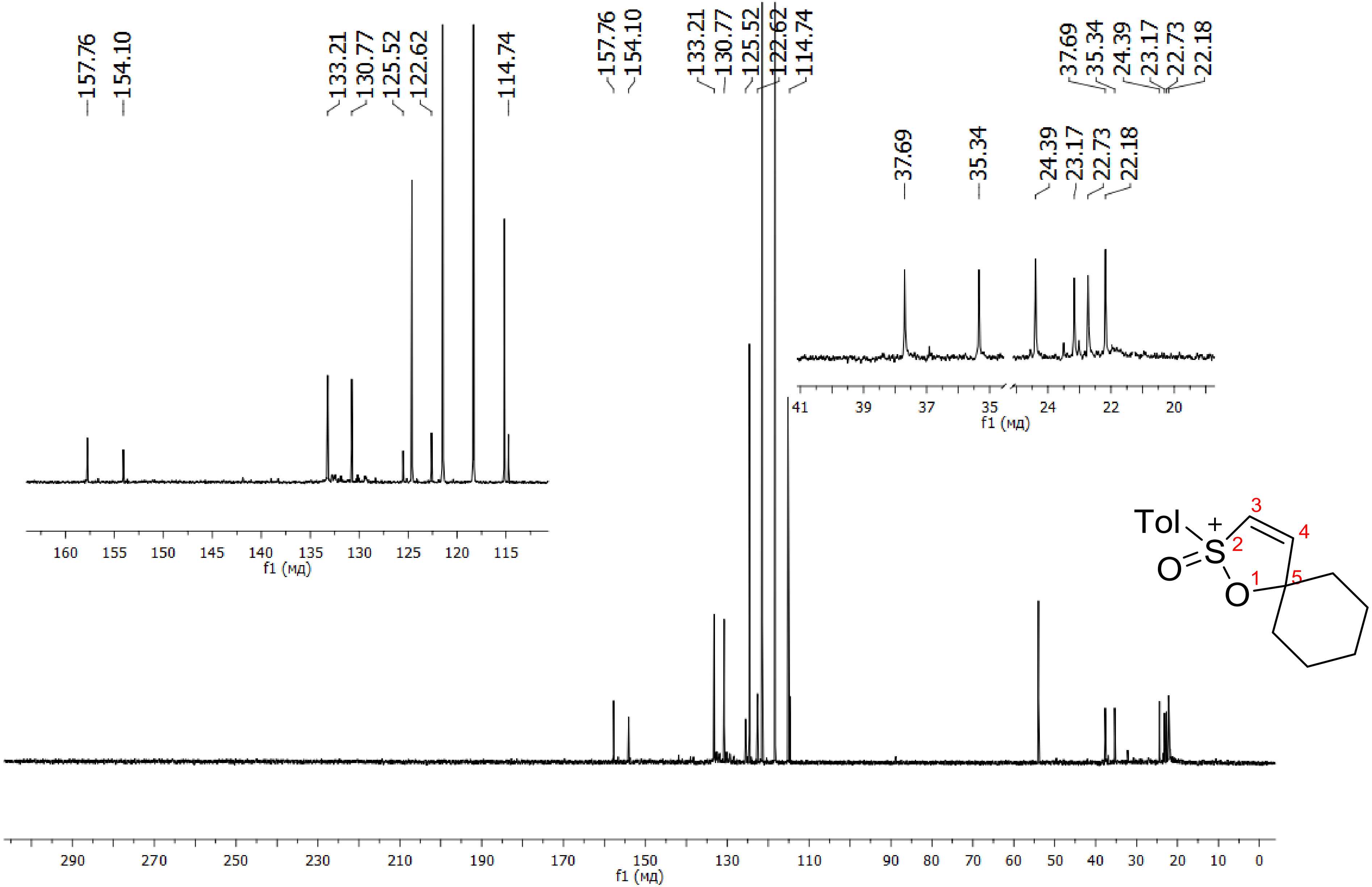

Fig. S74. ^{13}C NMR spectrum of the cation **Bg** (101 MHz, TfOH).

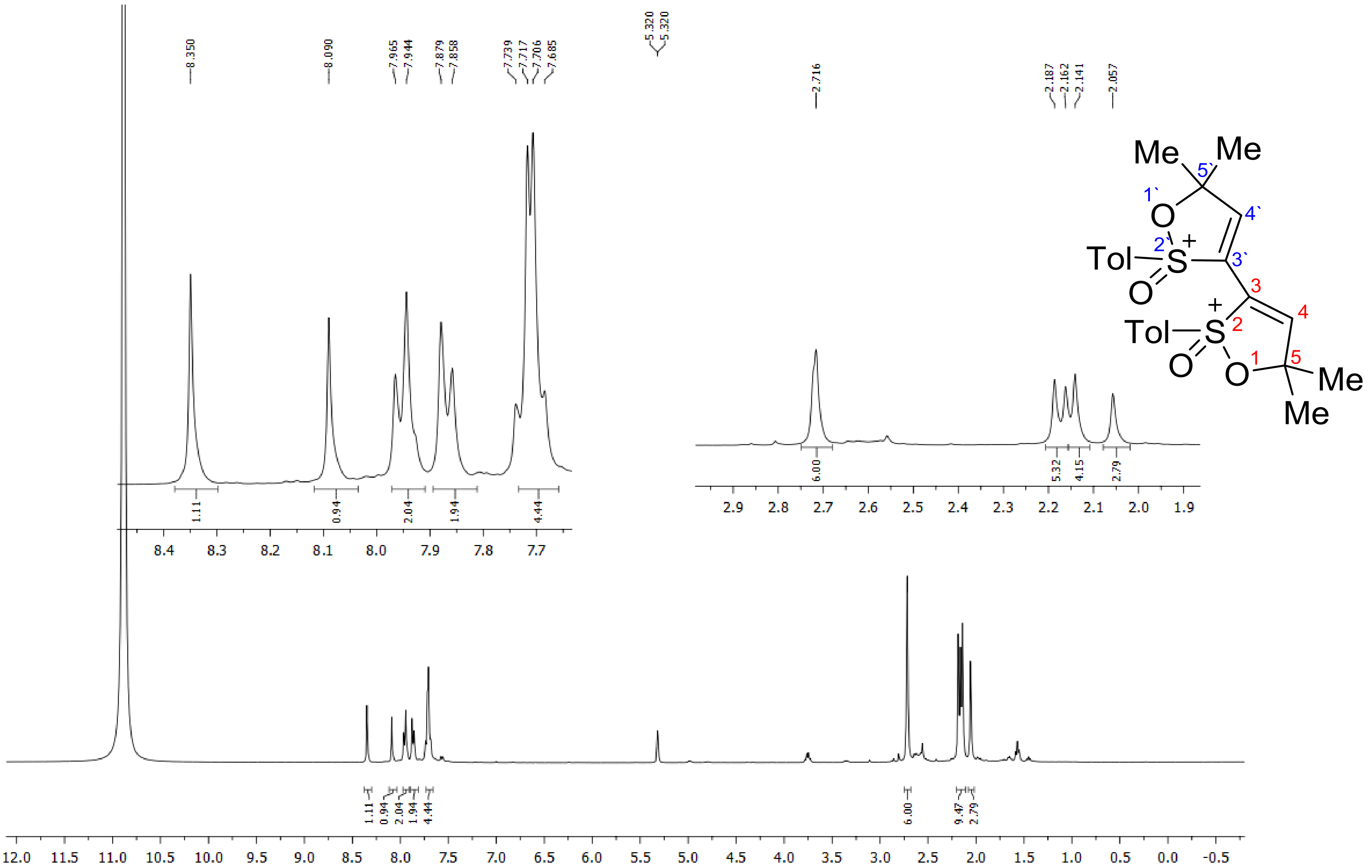

Fig. S75. ^{1}H NMR spectrum of the cation **Bh** (400 MHz, TfOH).

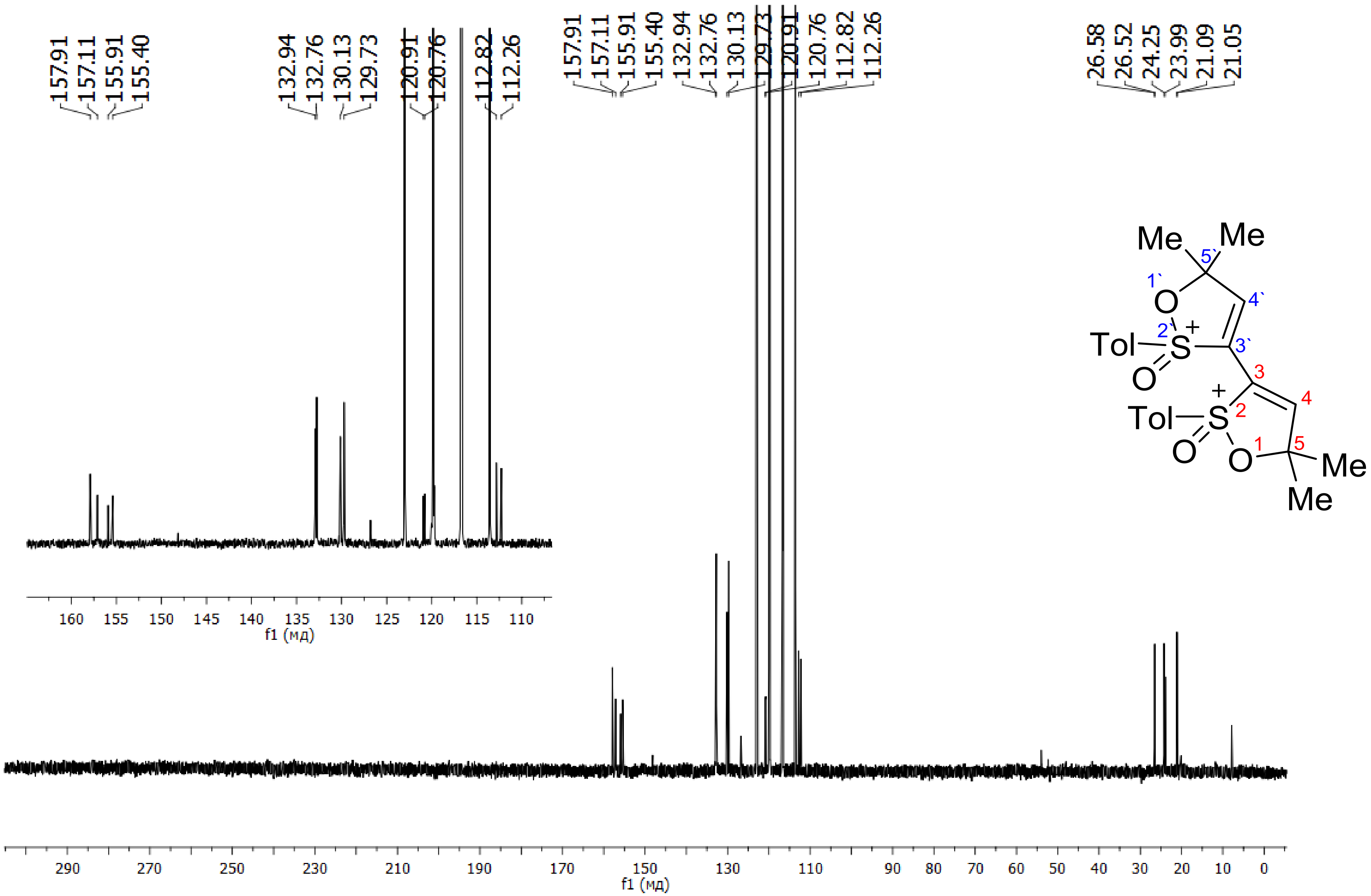

Fig. S76. ^{13}C NMR spectrum of the cation **Bh** (101 MHz, TfOH).

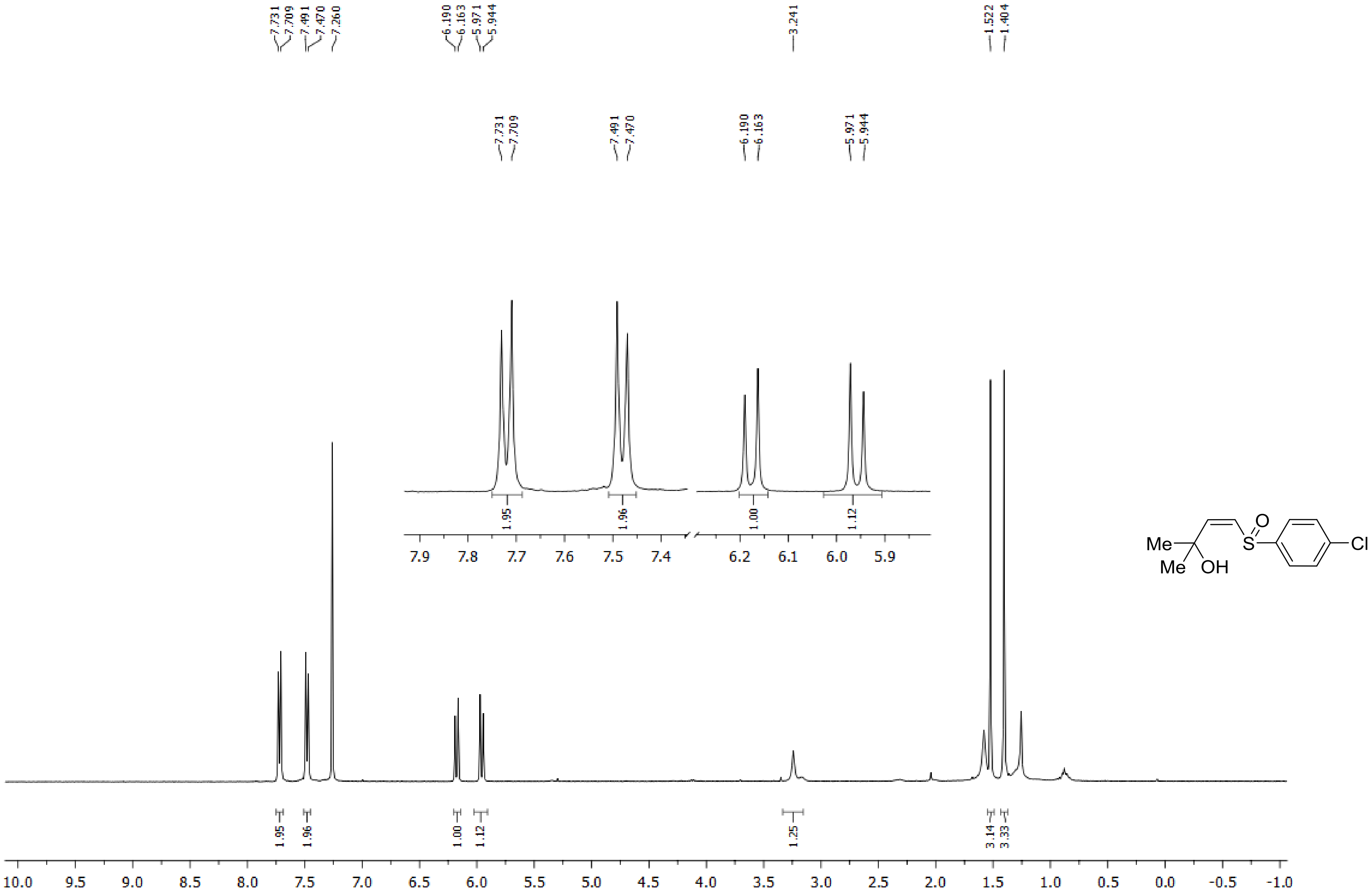

Fig. S77. ^{1}H NMR spectrum of the compound **7b** (400 MHz, $CDCl_3$).

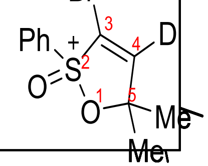

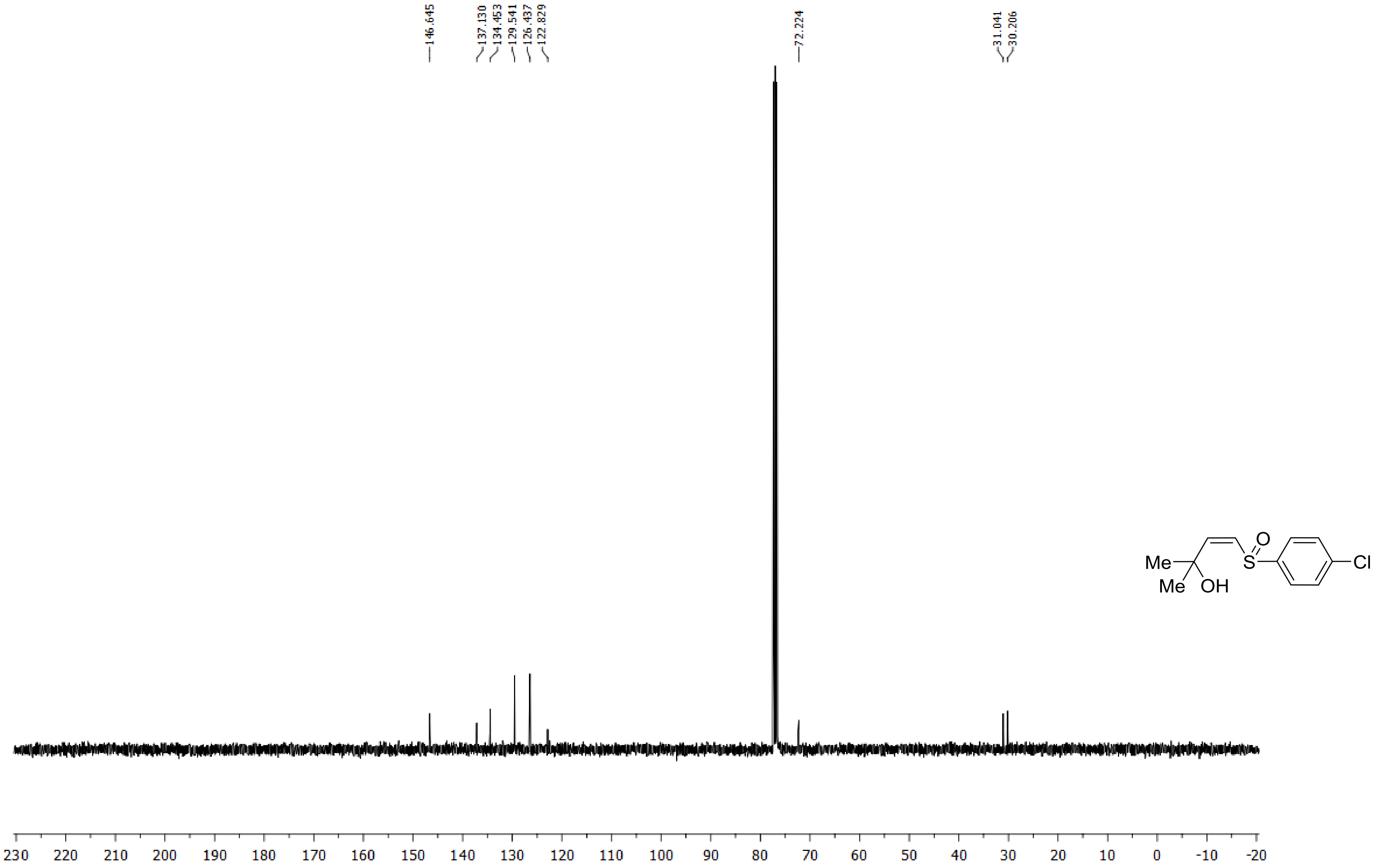

Fig. S78. ^{13}C NMR spectrum of the compound **7b** (101 MHz, $CDCl_3$).

III. X-ray data

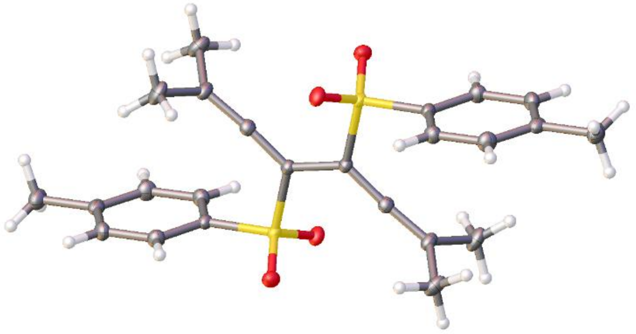

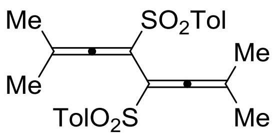

2h CCDC 1843276

Table S1. Crystal data and structure refinement for 2h

Identificationcode	LZA023_twin1_hklf4
Empiricalformula	$C_{24}H_{26}O_4S_2$
Formulaweight	442.57
Temperature/K	100(2)
Crystalsystem	triclinic
Spacegroup	P-1
a/Å	7.27098(19)
b/Å	8.65603(17)
c/Å	9.05158(16)
α/°	100.1837(16)
β/°	95.5031(18)
γ/°	93.7011(18)
Volume/Å^3	556.19(2)
Z	1
ρ_{calc}g/cm^3	1.321
μ/mm^{-1}	2.397
F(000)	234.0
Crystalsize/mm^3	0.17 × 0.15 × 0.15
Radiation	CuKα (λ = 1.54184)
2Θ range for data collection/°	9.988 to 139.966
Indexranges	$-8 \le h \le 8, -10 \le k \le 10, -10 \le l \le 11$
Reflectionscollected	4013
Independentreflections	4013 [R_{int} = ?, R_{sigma} = 0.0134]
Data/restraints/parameters	4013/0/140
Goodness-of-fit on F^2	1.105
Final R indexes [I>=2σ (I)]	R_1 = 0.0404, wR_2 = 0.1253
Final R indexes [all data]	R_1 = 0.0429, wR_2 = 0.1288
Largest diff. peak/hole / e Å^{-3}	0.35/-0.60

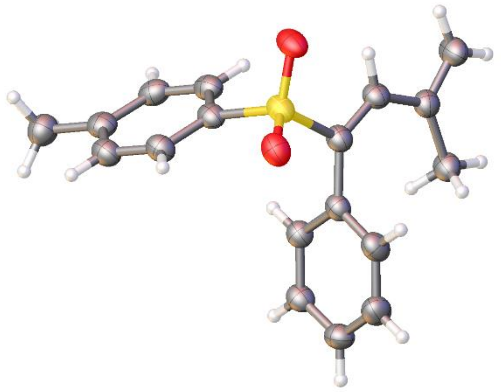

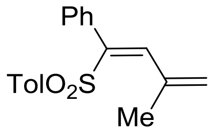

3e CCDC 1843277

Table S2. Crystal data and structure refinement for 3e

Identificationcode	LZA034
Empiricalformula	$C_{18}H_{18}O_2S$
Formulaweight	298.38
Temperature/K	100(2)
Crystalsystem	triclinic
Spacegroup	P-1
a/Å	9.1998(8)
b/Å	9.6465(7)
c/Å	10.0058(6)
α/°	105.703(6)
β/°	103.440(7)
γ/°	106.143(7)
Volume/Å^3	774.34(11)
Z	2
ρ_{calc}g/cm^3	1.280
μ/mm^{-1}	1.862
F(000)	316.0
Crystalsize/mm^3	? × ? × ?
Radiation	CuKα (λ = 1.54184)
2Θ range for data collection/°	9.738 to 143.94
Indexranges	-11 ≤ h ≤ 11, -11 ≤ k ≤ 11, -12 ≤ l ≤ 10
Reflectionscollected	8481
Independentreflections	3002 [R_{int} = 0.0344, R_{sigma} = 0.0368]
Data/restraints/parameters	3002/0/192
Goodness-of-fit on F^2	1.056
Final R indexes [I>=2σ (I)]	R_1 = 0.0508, wR_2 = 0.1441
Final R indexes [all data]	R_1 = 0.0589, wR_2 = 0.1516
Largest diff. peak/hole / e Å^{-3}	0.65/-0.4

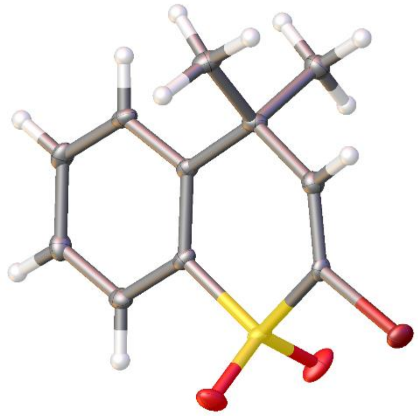

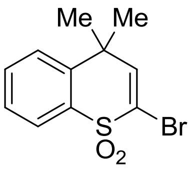

5c CCDC 1580895

Table S3. Crystal data and structure refinement for 5c

Identificationcode	AVV011
Empiricalformula	$C_{11}H_{11}BrO_2S$
Formulaweight	287.17
Temperature/K	100(2)
Crystalsystem	orthorhombic
Spacegroup	$Pna2_1$
a/Å	11.9294(3)
b/Å	7.97861(18)
c/Å	11.7594(3)
α/°	90
β/°	90
γ/°	90
Volume/Å^3	1119.26(5)
Z	4
ρ_{calc}g/cm^3	1.704
μ/mm^{-1}	3.835
F(000)	576.0
Crystalsize/mm^3	0.4 × 0.2 × 0.15
Radiation	MoKα (λ = 0.71073)
2Θ range for data collection/°	6.144 to 54.986
Indexranges	$-15 \leq h \leq 15$, $-9 \leq k \leq 10$, $-15 \leq l \leq 15$
Reflectionscollected	12208
Independentreflections	2570 [R_{int} = 0.0376, R_{sigma} = 0.0304]
Data/restraints/parameters	2570/1/138
Goodness-of-fit on F^2	1.055
Final R indexes [I>=2σ (I)]	R_1 = 0.0233, wR_2 = 0.0533
Final R indexes [all data]	R_1 = 0.0266, wR_2 = 0.0550
Largest diff. peak/hole / e Å^{-3}	0.29/-0.25
Flackparameter	-0.009(8)

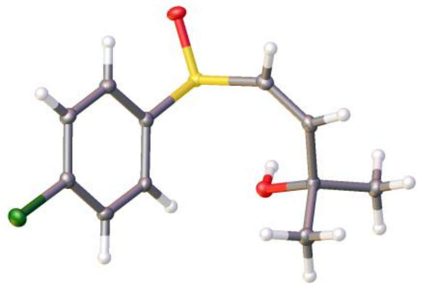

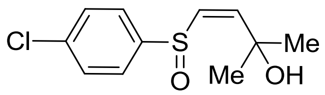

7b CCDC 1843239

Table S7. Crystal data and structure refinement for 7b

Identificationcode	LZA009
Empiricalformula	$C_{11}H_{14}ClO_2S$
Formulaweight	245.73
Temperature/K	100(2)
Crystalsystem	monoclinic
Spacegroup	C2/c
a/Å	20.1165(4)
b/Å	9.2661(2)
c/Å	12.5398(3)
α/°	90
β/°	106.712(2)
γ/°	90
Volume/Å^3	2238.71(9)
Z	8
ρ_{calc}g/cm^3	1.458
μ/mm^{-1}	4.580
F(000)	1032.0
Crystalsize/mm^3	0.29 × 0.22 × 0.12
Radiation	CuKα (λ = 1.54184)
2Θ range for data collection/°	9.18 to 143.988
Indexranges	$-17 \leq h \leq 24$, $-11 \leq k \leq 11$, $-15 \leq l \leq 15$
Reflectionscollected	6798
Independentreflections	2193 [R_{int} = 0.0311, R_{sigma} = 0.0275]
Data/restraints/parameters	2193/0/140
Goodness-of-fit on F^2	1.128
Final R indexes [I>=2σ (I)]	R_1 = 0.0325, wR_2 = 0.0885
Final R indexes [all data]	R_1 = 0.0341, wR_2 = 0.0892
Largest diff. peak/hole / e Å^{-3}	0.31/-0.78

IV. Data of DFT calculations

2-phenyl-5,5-dimethyl-2,5-dihydro-1,2-oxathiol-2-ium (Aa)

```
******************************************
 Gaussian 09:  IA32W-G09RevD.01 24-Apr-2013
              28-Apr-2018
 ******************************************
 %nprocshared=4
 Will use up to    4 processors via shared memory.
 %chk=C:\Users\chem\Desktop\LOZOVSKIY\lza 007 soph cation.chk
 ----------------------------------------------
 # opt freq b3lyp/6-311+g(2d,2p) geom=connectivity
 ----------------------------------------------
 1/14=-1,18=20,19=15,26=3,38=1,57=2/1,3;
 2/9=110,12=2,17=6,18=5,40=1/2;
 3/5=4,6=6,7=212,11=2,16=1,25=1,30=1,71=1,74=-5/1,2,3;
 4//1;
 5/5=2,38=5/2;
 6/7=2,8=2,9=2,10=2,28=1/1;
 7//1,2,3,16;
 1/14=-1,18=20,19=15,26=3/3(2);
 2/9=110/2;
 99//99;
 2/9=110/2;
 3/5=4,6=6,7=212,11=2,16=1,25=1,30=1,71=1,74=-5/1,2,3;
 4/5=5,16=3,69=1/1;
 5/5=2,38=5/2;
 7//1,2,3,16;
 1/14=-1,18=20,19=15,26=3/3(-5);
 2/9=110/2;
 6/7=2,8=2,9=2,10=2,19=2,28=1/1;
 99/9=1/99;
 -----------
 SOPh cation
 -----------
 Symbolic Z-matrix:
 Charge =  1 Multiplicity = 1
 C                    -6.4822   -0.0752   -1.4991
 C                    -5.4232    0.0999   -0.7115
 S                    -6.9139   -1.7944   -1.6035
 C                    -4.9194   -1.1583   -0.0896
 C                    -4.924    -1.0557    1.4351
 H                    -4.2428   -0.2751    1.7913
 H                    -4.6271   -2.0079    1.8895
 H                    -5.929    -0.8342    1.8143
 C                    -3.5284   -1.5246   -0.605
 H                    -2.7861   -0.761    -0.3489
 H                    -3.5295   -1.6515   -1.6942
 H                    -3.1996   -2.4823   -0.185
 O                    -5.8345   -2.2027   -0.4643
 C                    -8.3259   -1.9247   -0.4782
 C                    -8.5362   -3.1236    0.2152
 C                    -9.2573   -0.8907   -0.3394
```

C	-9.6408	-3.2756	1.0559
C	-10.3605	-1.0406	0.5058
C	-10.551	-2.2316	1.2039
H	-11.0753	-0.2288	0.6125
H	-9.1451	0.0391	-0.8878
H	-9.7909	-4.2103	1.5894
H	-11.4121	-2.3489	1.8565
H	-7.8368	-3.9486	0.1008
H	-6.9701	0.7314	-2.0317
H	-4.9686	1.0637	-0.5288

GradGradGradGradGradGradGradGradGradGradGradGradGradGradGradGradGradGrad
Berny optimization.
Initialization pass.

```
                 ----------------------------
                 !    Initial Parameters    !
                 ! (Angstroms and Degrees)  !
 --------------------------                            --------------------------
 ! Name  Definition              Value          Derivative Info.                !
 --------------------------------------------------------------------------------
 ! R1    R(1,2)                  1.3313         estimate D2E/DX2                !
 ! R2    R(1,3)                  1.7756         estimate D2E/DX2                !
 ! R3    R(1,25)                 1.0827         estimate D2E/DX2                !
 ! R4    R(2,4)                  1.4912         estimate D2E/DX2                !
 ! R5    R(2,26)                 1.0812         estimate D2E/DX2                !
 ! R6    R(3,13)                 1.6216         estimate D2E/DX2                !
 ! R7    R(3,14)                 1.8103         estimate D2E/DX2                !
 ! R8    R(4,5)                  1.5282         estimate D2E/DX2                !
 ! R9    R(4,9)                  1.528          estimate D2E/DX2                !
 ! R10   R(4,13)                 1.4383         estimate D2E/DX2                !
 ! R11   R(5,6)                  1.0956         estimate D2E/DX2                !
 ! R12   R(5,7)                  1.096          estimate D2E/DX2                !
 ! R13   R(5,8)                  1.0968         estimate D2E/DX2                !
 ! R14   R(9,10)                 1.0953         estimate D2E/DX2                !
 ! R15   R(9,11)                 1.0966         estimate D2E/DX2                !
 ! R16   R(9,12)                 1.0962         estimate D2E/DX2                !
 ! R17   R(14,15)                1.4009         estimate D2E/DX2                !
 ! R18   R(14,16)                1.3985         estimate D2E/DX2                !
 ! R19   R(15,17)                1.3964         estimate D2E/DX2                !
 ! R20   R(15,24)                1.0876         estimate D2E/DX2                !
 ! R21   R(16,18)                1.3978         estimate D2E/DX2                !
 ! R22   R(16,21)                1.0853         estimate D2E/DX2                !
 ! R23   R(17,19)                1.3929         estimate D2E/DX2                !
 ! R24   R(17,22)                1.0867         estimate D2E/DX2                !
 ! R25   R(18,19)                1.3936         estimate D2E/DX2                !
 ! R26   R(18,20)                1.0869         estimate D2E/DX2                !
 ! R27   R(19,23)                1.0868         estimate D2E/DX2                !
 ! A1    A(2,1,3)              110.825          estimate D2E/DX2                !
 ! A2    A(2,1,25)             123.4675         estimate D2E/DX2                !
 ! A3    A(3,1,25)             125.6483         estimate D2E/DX2                !
 ! A4    A(1,2,4)              113.8591         estimate D2E/DX2                !
 ! A5    A(1,2,26)             123.4815         estimate D2E/DX2                !
 ! A6    A(4,2,26)             122.658          estimate D2E/DX2                !
 ! A7    A(1,3,13)              92.3296         estimate D2E/DX2                !
```

! A8 A(1,3,14) 102.8723 estimate D2E/DX2 !
! A9 A(13,3,14) 93.6909 estimate D2E/DX2 !
! A10 A(2,4,5) 111.0045 estimate D2E/DX2 !
! A11 A(2,4,9) 111.6641 estimate D2E/DX2 !
! A12 A(2,4,13) 106.8034 estimate D2E/DX2 !
! A13 A(5,4,9) 110.8169 estimate D2E/DX2 !
! A14 A(5,4,13) 107.8649 estimate D2E/DX2 !
! A15 A(9,4,13) 108.4975 estimate D2E/DX2 !
! A16 A(4,5,6) 111.738 estimate D2E/DX2 !
! A17 A(4,5,7) 110.7628 estimate D2E/DX2 !
! A18 A(4,5,8) 111.179 estimate D2E/DX2 !
! A19 A(6,5,7) 108.4079 estimate D2E/DX2 !
! A20 A(6,5,8) 108.2674 estimate D2E/DX2 !
! A21 A(7,5,8) 106.2801 estimate D2E/DX2 !
! A22 A(4,9,10) 111.775 estimate D2E/DX2 !
! A23 A(4,9,11) 111.2153 estimate D2E/DX2 !
! A24 A(4,9,12) 110.6865 estimate D2E/DX2 !
! A25 A(10,9,11) 108.2766 estimate D2E/DX2 !
! A26 A(10,9,12) 108.4338 estimate D2E/DX2 !
! A27 A(11,9,12) 106.2459 estimate D2E/DX2 !
! A28 A(3,13,4) 115.0685 estimate D2E/DX2 !
! A29 A(3,14,15) 119.1037 estimate D2E/DX2 !
! A30 A(3,14,16) 121.8664 estimate D2E/DX2 !
! A31 A(15,14,16) 118.924 estimate D2E/DX2 !
! A32 A(14,15,17) 120.6574 estimate D2E/DX2 !
! A33 A(14,15,24) 120.0391 estimate D2E/DX2 !
! A34 A(17,15,24) 119.3034 estimate D2E/DX2 !
! A35 A(14,16,18) 120.42 estimate D2E/DX2 !
! A36 A(14,16,21) 120.9583 estimate D2E/DX2 !
! A37 A(18,16,21) 118.6202 estimate D2E/DX2 !
! A38 A(15,17,19) 119.9512 estimate D2E/DX2 !
! A39 A(15,17,22) 119.8984 estimate D2E/DX2 !
! A40 A(19,17,22) 120.1497 estimate D2E/DX2 !
! A41 A(16,18,19) 120.1608 estimate D2E/DX2 !
! A42 A(16,18,20) 119.8878 estimate D2E/DX2 !
! A43 A(19,18,20) 119.9497 estimate D2E/DX2 !
! A44 A(17,19,18) 119.8667 estimate D2E/DX2 !
! A45 A(17,19,23) 120.0423 estimate D2E/DX2 !
! A46 A(18,19,23) 120.0892 estimate D2E/DX2 !
! D1 D(3,1,2,4) -2.3507 estimate D2E/DX2 !
! D2 D(3,1,2,26) 178.0651 estimate D2E/DX2 !
! D3 D(25,1,2,4) -179.6907 estimate D2E/DX2 !
! D4 D(25,1,2,26) 0.7251 estimate D2E/DX2 !
! D5 D(2,1,3,13) 7.2605 estimate D2E/DX2 !
! D6 D(2,1,3,14) 101.5838 estimate D2E/DX2 !
! D7 D(25,1,3,13) -175.4702 estimate D2E/DX2 !
! D8 D(25,1,3,14) -81.1469 estimate D2E/DX2 !
! D9 D(1,2,4,5) -121.9421 estimate D2E/DX2 !
! D10 D(1,2,4,9) 113.8549 estimate D2E/DX2 !
! D11 D(1,2,4,13) -4.6058 estimate D2E/DX2 !
! D12 D(26,2,4,5) 57.646 estimate D2E/DX2 !
! D13 D(26,2,4,9) -66.557 estimate D2E/DX2 !
! D14 D(26,2,4,13) 174.9823 estimate D2E/DX2 !
! D15 D(1,3,13,4) -10.3584 estimate D2E/DX2 !
! D16 D(14,3,13,4) -113.4231 estimate D2E/DX2 !

! D17   D(1,3,14,15)   -148.6812   estimate D2E/DX2   !
! D18   D(1,3,14,16)   35.1007   estimate D2E/DX2   !
! D19   D(13,3,14,15)   -55.4409   estimate D2E/DX2   !
! D20   D(13,3,14,16)   128.3409   estimate D2E/DX2   !
! D21   D(2,4,5,6)   -63.4005   estimate D2E/DX2   !
! D22   D(2,4,5,7)   175.6202   estimate D2E/DX2   !
! D23   D(2,4,5,8)   57.6889   estimate D2E/DX2   !
! D24   D(9,4,5,6)   61.2826   estimate D2E/DX2   !
! D25   D(9,4,5,7)   -59.6967   estimate D2E/DX2   !
! D26   D(9,4,5,8)   -177.628   estimate D2E/DX2   !
! D27   D(13,4,5,6)   179.9131   estimate D2E/DX2   !
! D28   D(13,4,5,7)   58.9339   estimate D2E/DX2   !
! D29   D(13,4,5,8)   -58.9974   estimate D2E/DX2   !
! D30   D(2,4,9,10)   62.486   estimate D2E/DX2   !
! D31   D(2,4,9,11)   -58.6664   estimate D2E/DX2   !
! D32   D(2,4,9,12)   -176.5293   estimate D2E/DX2   !
! D33   D(5,4,9,10)   -61.8222   estimate D2E/DX2   !
! D34   D(5,4,9,11)   177.0254   estimate D2E/DX2   !
! D35   D(5,4,9,12)   59.1625   estimate D2E/DX2   !
! D36   D(13,4,9,10)   179.9309   estimate D2E/DX2   !
! D37   D(13,4,9,11)   58.7784   estimate D2E/DX2   !
! D38   D(13,4,9,12)   -59.0844   estimate D2E/DX2   !
! D39   D(2,4,13,3)   10.4272   estimate D2E/DX2   !
! D40   D(5,4,13,3)   129.8158   estimate D2E/DX2   !
! D41   D(9,4,13,3)   -110.0811   estimate D2E/DX2   !
! D42   D(3,14,15,17)   -177.779   estimate D2E/DX2   !
! D43   D(3,14,15,24)   2.125   estimate D2E/DX2   !
! D44   D(16,14,15,17)   -1.4484   estimate D2E/DX2   !
! D45   D(16,14,15,24)   178.4556   estimate D2E/DX2   !
! D46   D(3,14,16,18)   177.9049   estimate D2E/DX2   !
! D47   D(3,14,16,21)   -1.6511   estimate D2E/DX2   !
! D48   D(15,14,16,18)   1.6801   estimate D2E/DX2   !
! D49   D(15,14,16,21)   -177.8758   estimate D2E/DX2   !
! D50   D(14,15,17,19)   0.4275   estimate D2E/DX2   !
! D51   D(14,15,17,22)   -179.8795   estimate D2E/DX2   !
! D52   D(24,15,17,19)   -179.4772   estimate D2E/DX2   !
! D53   D(24,15,17,22)   0.2158   estimate D2E/DX2   !
! D54   D(14,16,18,19)   -0.8987   estimate D2E/DX2   !
! D55   D(14,16,18,20)   179.5742   estimate D2E/DX2   !
! D56   D(21,16,18,19)   178.6675   estimate D2E/DX2   !
! D57   D(21,16,18,20)   -0.8596   estimate D2E/DX2   !
! D58   D(15,17,19,18)   0.3796   estimate D2E/DX2   !
! D59   D(15,17,19,23)   179.8964   estimate D2E/DX2   !
! D60   D(22,17,19,18)   -179.3127   estimate D2E/DX2   !
! D61   D(22,17,19,23)   0.2042   estimate D2E/DX2   !
! D62   D(16,18,19,17)   -0.1458   estimate D2E/DX2   !
! D63   D(16,18,19,23)   -179.6625   estimate D2E/DX2   !
! D64   D(20,18,19,17)   179.381   estimate D2E/DX2   !
! D65   D(20,18,19,23)   -0.1356   estimate D2E/DX2   !

Trust Radius=3.00D-01 FncErr=1.00D-07 GrdErr=1.00D-06
Number of steps in this run=   148 maximum allowed number of steps=   156.
GradGradGradGradGradGradGradGradGradGradGradGradGradGradGradGradGradGrad

Input orientation:

Center Number	Atomic Number	Atomic Type	Coordinates (Angstroms) X	Y	Z
1	6	0	-6.482200	-0.075200	-1.499100
2	6	0	-5.423200	0.099900	-0.711500
3	16	0	-6.913900	-1.794400	-1.603500
4	6	0	-4.919400	-1.158300	-0.089600
5	6	0	-4.924000	-1.055700	1.435100
6	1	0	-4.242800	-0.275100	1.791300
7	1	0	-4.627100	-2.007900	1.889500
8	1	0	-5.929000	-0.834200	1.814300
9	6	0	-3.528400	-1.524600	-0.605000
10	1	0	-2.786100	-0.761000	-0.348900
11	1	0	-3.529500	-1.651500	-1.694200
12	1	0	-3.199600	-2.482300	-0.185000
13	8	0	-5.834500	-2.202700	-0.464300
14	6	0	-8.325900	-1.924700	-0.478200
15	6	0	-8.536200	-3.123600	0.215200
16	6	0	-9.257300	-0.890700	-0.339400
17	6	0	-9.640800	-3.275600	1.055900
18	6	0	-10.360500	-1.040600	0.505800
19	6	0	-10.551000	-2.231600	1.203900
20	1	0	-11.075300	-0.228800	0.612500
21	1	0	-9.145100	0.039100	-0.887800
22	1	0	-9.790900	-4.210300	1.589400
23	1	0	-11.412100	-2.348900	1.856500
24	1	0	-7.836800	-3.948600	0.100800
25	1	0	-6.970100	0.731400	-2.031700
26	1	0	-4.968600	1.063700	-0.528800

Distance matrix (angstroms):

		1	2	3	4	5
1	C	0.000000				
2	C	1.331336	0.000000			
3	S	1.775644	2.570257	0.000000		
4	C	2.366884	1.491188	2.583514	0.000000	
5	C	3.463942	2.488474	3.706544	1.528155	0.000000
6	H	3.985173	2.792486	4.579052	2.185318	1.095558
7	H	4.319645	3.441191	4.180441	2.173499	1.096045
8	H	3.443941	2.740081	3.684205	2.179259	1.096759
9	C	3.409561	2.498121	3.539972	1.527971	2.515865
10	H	3.931213	2.797664	4.436290	2.185418	2.800022
11	H	3.352793	2.760290	3.388630	2.179406	3.477373
12	H	4.277435	3.448095	4.035018	2.172508	2.762873
13	O	2.452872	2.352072	1.621601	1.438256	2.398404
14	C	2.803947	3.546703	1.810255	3.513207	3.998600
15	C	4.055920	4.576078	2.776022	4.127536	4.337321
16	C	3.116268	3.977445	2.811787	4.353319	4.685463
17	C	5.171783	5.683824	4.086850	5.299693	5.226849
18	C	4.471334	5.211477	4.110525	5.474845	5.515375
19	C	5.339607	5.949704	4.615315	5.877077	5.753201
20	H	5.057570	5.814401	4.967797	6.265143	6.260905
21	H	2.734555	3.726569	2.975265	4.464015	4.940864
22	H	6.130692	6.553531	4.930346	5.988761	5.801898

23	H	6.382294	6.961190	5.702006	6.881859	6.629132
24	H	4.404299	4.782849	2.897751	4.041441	4.316669
25	H	1.082734	2.129465	2.562456	3.398248	4.404424
26	H	2.128276	1.081180	3.620484	2.265525	2.889766

		6	7	8	9	10
6	H	0.000000				
7	H	1.777618	0.000000			
8	H	1.776624	1.754472	0.000000		
9	C	2.795330	2.768258	3.477434	0.000000	
10	H	2.634109	3.155071	3.816099	1.095300	0.000000
11	H	3.814706	3.764924	4.328412	1.096568	1.776363
12	H	3.140981	2.562491	3.763384	1.096220	1.777841
13	O	3.367029	2.652572	2.659650	2.407844	3.374101
14	C	4.954141	4.392499	3.491400	4.815824	5.662182
15	C	5.388073	4.396491	3.820467	5.320488	6.242094
16	C	5.483070	5.258791	3.964744	5.769980	6.472507
17	C	6.219503	5.238238	4.507002	6.571607	7.435293
18	C	6.297996	5.976803	4.625253	6.938712	7.627596
19	C	6.630710	5.967636	4.867052	7.286212	8.054038
20	H	6.933597	6.809935	5.319327	7.753522	8.361720
21	H	5.595431	5.684710	4.290373	5.837161	6.431754
22	H	6.804996	5.621873	5.134482	7.158721	8.044992
23	H	7.463495	6.793644	5.688627	8.300071	9.043953
24	H	5.410104	4.155471	4.034262	4.993620	5.989373
25	H	4.802763	5.326277	4.280971	4.355492	4.750256
26	H	2.775254	3.924223	3.164571	2.962985	2.850474

		11	12	13	14	15
11	H	0.000000				
12	H	1.754066	0.000000			
13	O	2.670112	2.664373	0.000000		
14	C	4.955678	5.164866	2.506901	0.000000	
15	C	5.556971	5.389872	2.934103	1.400854	0.000000
16	C	5.934812	6.265202	3.667766	1.398545	2.411101
17	C	6.895559	6.607436	4.236749	2.430505	1.396432
18	C	7.202483	7.337179	4.772447	2.426826	2.784135
19	C	7.618199	7.485651	5.002909	2.806193	2.415080
20	H	8.017733	8.230487	5.702786	3.409531	3.871013
21	H	5.919744	6.496180	4.020584	2.166881	3.404415
22	H	7.518950	7.041286	4.888890	3.412501	2.154905
23	H	8.673478	8.463491	6.042939	3.892994	3.400710
24	H	5.201108	4.871893	2.716010	2.161164	1.087600
25	H	4.198791	5.287234	3.515007	3.362503	4.728874
26	H	3.286560	3.977647	3.379839	4.494948	5.551107

		16	17	18	19	20
16	C	0.000000				
17	C	2.789567	0.000000			
18	C	1.397814	2.411598	0.000000		
19	C	2.419390	1.392948	1.393598	0.000000	
20	H	2.156235	3.396672	1.086896	2.153103	0.000000
21	H	1.085292	3.874393	2.141278	3.392321	2.459338
22	H	3.876176	1.086654	3.397886	2.154441	4.296087
23	H	3.404627	2.153424	2.154507	1.086802	2.481087
24	H	3.400345	2.149316	3.871634	3.395850	4.958491
25	H	3.275114	5.720311	4.590610	5.663155	4.976590
26	H	4.716832	6.570407	5.879716	6.710025	6.345463

		21	22	23	24	25
21	H	0.000000				
22	H	4.960945	0.000000			
23	H	4.286376	2.482829	0.000000		
24	H	4.311697	2.470410	4.292353	0.000000	
25	H	2.553118	6.744604	6.658657	5.215470	0.000000
26	H	4.315303	7.453618	7.671648	5.809141	2.524903

		26
26	H	0.000000

Stoichiometry C11H13OS(1+)
Framework group C1[X(C11H13OS)]
Deg. of freedom 72
Full point group C1 NOp 1
Largest Abelian subgroup C1 NOp 1
Largest concise Abelian subgroup C1 NOp 1

Standard orientation:

Center Number	Atomic Number	Atomic Type	Coordinates (Angstroms) X	Y	Z
1	6	0	1.150475	1.707343	0.228486
2	6	0	2.058316	0.968777	0.863152
3	16	0	0.423352	0.798917	-1.112769
4	6	0	2.186379	-0.418339	0.331035
5	6	0	1.918482	-1.451471	1.424713
6	1	0	2.658808	-1.391107	2.230022
7	1	0	1.937115	-2.467019	1.012854
8	1	0	0.922210	-1.315555	1.862717
9	6	0	3.552063	-0.651443	-0.313375
10	1	0	4.366588	-0.546020	0.411274
11	1	0	3.728907	0.053502	-1.134496
12	1	0	3.606766	-1.654194	-0.752916
13	8	0	1.173385	-0.571613	-0.678385
14	6	0	-1.171320	0.280231	-0.430855
15	6	0	-1.733249	-0.930208	-0.856843
16	6	0	-1.895814	1.089260	0.450342
17	6	0	-2.983021	-1.339394	-0.387098
18	6	0	-3.144668	0.677797	0.924625
19	6	0	-3.686872	-0.536261	0.507260
20	1	0	-3.697201	1.310226	1.614614
21	1	0	-1.507082	2.049250	0.774635
22	1	0	-3.406448	-2.281664	-0.724222
23	1	0	-4.660397	-0.852481	0.872491
24	1	0	-1.196664	-1.561413	-1.561490
25	1	0	0.934326	2.737161	0.483568
26	1	0	2.647734	1.331802	1.693664

Rotational constants (GHZ): 1.5300094 0.4671305 0.4411246
Standard basis: 6-311+G(2d,2p) (5D, 7F)
There are 502 symmetry adapted cartesian basis functions of A symmetry.
There are 476 symmetry adapted basis functions of A symmetry.
476 basis functions, 703 primitive gaussians, 502 cartesian basis functions
51 alpha electrons 51 beta electrons
nuclear repulsion energy 904.9648232301 Hartrees.
NAtoms= 26 NActive= 26 NUniq= 26 SFac= 1.00D+00 NAtFMM= 60 NAOKFM=F Big=F

Integral buffers will be 262144 words long.
Raffenetti 2 integral format.
Two-electron integral symmetry is turned on.
One-electron integrals computed using PRISM.
NBasis= 476 RedAO= T EigKep= 2.17D-06 NBF= 476
NBsUse= 476 1.00D-06 EigRej= -1.00D+00 NBFU= 476
ExpMin= 4.05D-02 ExpMax= 9.34D+04 ExpMxC= 3.17D+03 IAcc=2 IRadAn= 4 AccDes= 0.00D+00
Harris functional with IExCor= 402 and IRadAn= 4 diagonalized for initial guess.
HarFok: IExCor= 402 AccDes= 0.00D+00 IRadAn= 4 IDoV= 1 UseB2=F ITyADJ=14
ICtDFT= 3500011 ScaDFX= 1.000000 1.000000 1.000000 1.000000
FoFCou: FMM=F IPFlag= 0 FMFlag= 100000 FMFlg1= 0
NFxFlg= 0 DoJE=T BraDBF=F KetDBF=T FulRan=T
wScrn= 0.000000 ICntrl= 500 IOpCl= 0 I1Cent= 200000004 NGrid= 0
NMat0= 1 NMatS0= 1 NMatT0= 0 NMatD0= 1 NMtDS0= 0 NMtDT0= 0
Petite list used in FoFCou.
Requested convergence on RMS density matrix=1.00D-08 within 128 cycles.
Requested convergence on MAX density matrix=1.00D-06.
Requested convergence on energy=1.00D-06.
No special actions if energy rises.
SCF Done: E(RB3LYP) = -900.279596569 A.U. after 15 cycles
NFock= 15 Conv=0.66D-08 -V/T= 2.0031
****
Gaussian 09: IA32W-G09RevD.01 24-Apr-2013
05-May-2018
****
%mem=1000mb
%nproc=4
Will use up to 4 processors via shared memory.

#N B3LYP/6-311+G(2d,2p) pop=nboread

1/38=1/1;
2/12=2,17=6,18=5,40=1/2;
3/5=4,6=6,7=212,11=2,16=1,25=1,30=1,74=-5/1,2,3;
4//1;
5/5=2,38=5/2;
6/7=2,8=2,9=2,10=2,28=1,40=2/1,7;
99/5=1,9=1/99;

SOphcat NBOnaomo

Symbolic Z-matrix:
Charge = 1 Multiplicity = 1

6	1.09681	-0.43423	-1.62892
6	2.11316	0.30345	-1.21542
16	0.28726	-1.19528	-0.23342
6	2.36644	0.35615	0.26344
6	2.17582	1.74686	0.85785
1	2.9373	2.42201	0.46817
1	2.2841	1.70115	1.93983
1	1.19567	2.15426	0.62053
6	3.70908	-0.26438	0.64093
1	4.52077	0.35644	0.26268
1	3.81238	-1.26512	0.2265
1	3.79672	-0.31896	1.72422

8	1.32024	-0.55082	0.84958
6	-1.27563	-0.36881	-0.0946
6	-2.2864	-1.14347	0.48179
6	-1.50593	0.94072	-0.52335
6	-3.54965	-0.58826	0.63862
6	-2.77227	1.47762	-0.3615
6	-3.78874	0.71618	0.2192
1	-2.97314	2.4866	-0.69143
1	-0.72068	1.5221	-0.98274
1	-4.34146	-1.17318	1.08315
1	-4.77382	1.1447	0.33851
1	-2.08921	-2.15812	0.79947
1	0.74231	-0.64097	-2.62511
1	2.75481	0.84928	-1.89395

Input orientation:

Center Number	Atomic Number	Atomic Type	Coordinates (Angstroms) X	Y	Z
1	6	0	1.096810	-0.434230	-1.628920
2	6	0	2.113160	0.303450	-1.215420
3	16	0	0.287260	-1.195280	-0.233420
4	6	0	2.366440	0.356150	0.263440
5	6	0	2.175820	1.746860	0.857850
6	1	0	2.937300	2.422010	0.468170
7	1	0	2.284100	1.701150	1.939830
8	1	0	1.195670	2.154260	0.620530
9	6	0	3.709080	-0.264380	0.640930
10	1	0	4.520770	0.356440	0.262680
11	1	0	3.812380	-1.265120	0.226500
12	1	0	3.796720	-0.318960	1.724220
13	8	0	1.320240	-0.550820	0.849580
14	6	0	-1.275630	-0.368810	-0.094600
15	6	0	-2.286400	-1.143470	0.481790
16	6	0	-1.505930	0.940720	-0.523350
17	6	0	-3.549650	-0.588260	0.638620
18	6	0	-2.772270	1.477620	-0.361500
19	6	0	-3.788740	0.716180	0.219200
20	1	0	-2.973140	2.486600	-0.691430
21	1	0	-0.720680	1.522100	-0.982740
22	1	0	-4.341460	-1.173180	1.083150
23	1	0	-4.773820	1.144700	0.338510
24	1	0	-2.089210	-2.158120	0.799470
25	1	0	0.742310	-0.640970	-2.625110
26	1	0	2.754810	0.849280	-1.893950

Distance matrix (angstroms):

		1	2	3	4	5
1	C	0.000000				
2	C	1.322165	0.000000			
3	S	1.783813	2.558208	0.000000		
4	C	2.411988	1.501318	2.641362	0.000000	
5	C	3.479287	2.527015	3.662474	1.524380	0.000000
6	H	3.992906	2.828777	4.538692	2.153038	1.089738

7	H	4.324983	3.455197	4.135177	2.150834	1.088345
8	H	3.430755	2.763693	3.574053	2.175182	1.087653
9	C	3.464826	2.513051	3.652385	1.526512	2.538312
10	H	3.990841	2.825628	4.536139	2.154330	2.790392
11	H	3.392238	2.725233	3.555682	2.172700	3.485535
12	H	4.306547	3.444307	4.112980	2.152987	2.765054
13	O	2.491280	2.371228	1.629499	1.503559	2.451820
14	C	2.826108	3.632089	1.773400	3.730742	4.158813
15	C	4.050213	4.932568	2.671691	4.893410	5.329806
16	C	3.144364	3.739370	2.803941	3.994497	4.014082
17	C	5.172529	6.024950	3.981307	6.002732	6.187233
18	C	4.497922	5.096595	4.064668	5.296658	5.103224
19	C	5.348606	6.087767	4.524635	6.165859	6.086555
20	H	5.096531	5.559780	4.939253	5.827668	5.427640
21	H	2.747371	3.093525	2.993589	3.527423	3.439186
22	H	6.121777	7.008993	4.812369	6.928687	7.145091
23	H	6.389687	7.110056	5.605100	7.184063	6.994984
24	H	4.361146	5.270581	2.764332	5.144094	5.782971
25	H	1.077407	2.181372	2.496900	3.460602	4.459562
26	H	2.113434	1.081688	3.609209	2.246851	2.951827

		6	7	8	9	10
6	H	0.000000				
7	H	1.764112	0.000000			
8	H	1.768666	1.769333	0.000000		
9	C	2.800390	2.753365	3.488189	0.000000	
10	H	2.610782	3.102220	3.796907	1.089647	0.000000
11	H	3.797249	3.750986	4.323724	1.088073	1.769910
12	H	3.135152	2.532854	3.755051	1.088199	1.765365
13	O	3.405595	2.681239	2.717616	2.414982	3.378012
14	C	5.084699	4.592964	3.603417	5.039766	5.852511
15	C	6.324545	5.577384	4.797821	6.061675	6.973902
16	C	4.787444	4.583649	3.174867	5.477604	6.105763
17	C	7.153412	6.400561	5.480858	7.265952	8.134216
18	C	5.846316	5.559943	4.143280	6.785818	7.405071
19	C	6.943447	6.388280	5.203219	7.573418	8.317407
20	H	6.023466	5.931192	4.382997	7.348138	7.848987
21	H	4.036805	4.195495	2.577304	5.044858	5.512044
22	H	8.141495	7.272807	6.476549	8.113733	9.030616
23	H	7.817270	7.258657	6.060822	8.604450	9.328264
24	H	6.808315	5.943082	5.423936	6.101766	7.092460
25	H	4.875265	5.357361	4.307322	4.428381	4.859107
26	H	2.843658	3.955391	3.233651	2.928564	2.830646

		11	12	13	14	15
11	H	0.000000				
12	H	1.771618	0.000000			
13	O	2.666311	2.636609	0.000000		
14	C	5.176323	5.388815	2.768239	0.000000	
15	C	6.105333	6.263210	3.673466	1.397848	0.000000
16	C	5.806240	5.895461	3.478055	1.397045	2.441984
17	C	7.404557	7.431030	4.874601	2.399362	1.388760
18	C	7.157233	7.122468	4.725455	2.391751	2.795947
19	C	7.855102	7.802295	5.301353	2.755249	2.405052
20	H	7.807770	7.716057	5.480305	3.375072	3.876269
21	H	5.457059	5.578892	3.437986	2.161551	3.420759
22	H	8.199232	8.207962	5.700591	3.381334	2.141445

23	H	8.918666	8.804354	6.346144	3.836096	3.382826
24	H	5.996208	6.235531	3.769652	2.159376	1.081351
25	H	4.236340	5.324451	3.523578	3.248020	4.367885
26	H	3.175760	3.942273	3.397827	4.578848	5.918529
		16	17	18	19	20
16	C	0.000000				
17	C	2.804415	0.000000			
18	C	1.384945	2.423308	0.000000		
19	C	2.411021	1.390914	1.396501	0.000000	
20	H	2.137920	3.399435	1.080390	2.151473	0.000000
21	H	1.079657	3.884006	2.144047	3.392220	2.467529
22	H	3.884539	1.080140	3.402369	2.149788	4.291320
23	H	3.385781	2.142851	2.146404	1.080855	2.470611
24	H	3.419485	2.150167	3.877245	3.389206	4.957574
25	H	3.460307	5.392183	4.686643	5.519273	5.227368
26	H	4.476696	6.944541	5.769907	6.877584	6.077523
		21	22	23	24	25
21	H	0.000000				
22	H	4.964120	0.000000			
23	H	4.279729	2.472649	0.000000		
24	H	4.311979	2.474512	4.281149	0.000000	
25	H	3.084897	6.314995	6.511478	4.695423	0.000000
26	H	3.655410	7.956791	7.858207	6.305830	2.608754
		26				
26	H	0.000000				

Stoichiometry C11H13OS(1+)
Framework group C1[X(C11H13OS)]
Deg. of freedom 72
Full point group C1 NOp 1
Largest Abelian subgroup C1 NOp 1
Largest concise Abelian subgroup C1 NOp 1

Standard orientation:

Center Number	Atomic Number	Atomic Type	Coordinates (Angstroms) X	Y	Z
1	6	0	-1.096810	-0.434232	1.628920
2	6	0	-2.113160	0.303447	1.215420
3	16	0	-0.287259	-1.195281	0.233420
4	6	0	-2.366441	0.356147	-0.263440
5	6	0	-2.175821	1.746857	-0.857850
6	1	0	-2.937302	2.422007	-0.468170
7	1	0	-2.284101	1.701147	-1.939830
8	1	0	-1.195672	2.154258	-0.620530
9	6	0	-3.709080	-0.264384	-0.640930
10	1	0	-4.520771	0.356436	-0.262680
11	1	0	-3.812379	-1.265124	-0.226500
12	1	0	-3.796720	-0.318964	-1.724220
13	8	0	-1.320240	-0.550822	-0.849580
14	6	0	1.275630	-0.368810	0.094600
15	6	0	2.286400	-1.143470	-0.481790
16	6	0	1.505929	0.940720	0.523350
17	6	0	3.549650	-0.588259	-0.638620
18	6	0	2.772269	1.477621	0.361500
19	6	0	3.788739	0.716181	-0.219200

20	1	0	2.973138	2.486601	0.691430
21	1	0	0.720679	1.522099	0.982740
22	1	0	4.341460	-1.173178	-1.083150
23	1	0	4.773819	1.144702	-0.338510
24	1	0	2.089211	-2.158120	-0.799470
25	1	0	-0.742310	-0.640972	2.625110
26	1	0	-2.754811	0.849277	1.893950

Rotational constants (GHZ): 1.6756382 0.4379831 0.4104631
Standard basis: 6-311+G(2d,2p) (5D, 7F)
There are 502 symmetry adapted cartesian basis functions of A symmetry.
There are 476 symmetry adapted basis functions of A symmetry.
476 basis functions, 703 primitive gaussians, 502 cartesian basis functions
51 alpha electrons 51 beta electrons
nuclear repulsion energy 897.7508680452 Hartrees.
NAtoms= 26 NActive= 26 NUniq= 26 SFac= 1.00D+00 NAtFMM= 60 NAOKFM=F Big=F
Integral buffers will be 262144 words long.
Raffenetti 2 integral format.
Two-electron integral symmetry is turned on.
One-electron integrals computed using PRISM.
NBasis= 476 RedAO= T EigKep= 1.41D-06 NBF= 476
NBsUse= 476 1.00D-06 EigRej= -1.00D+00 NBFU= 476
ExpMin= 4.05D-02 ExpMax= 9.34D+04 ExpMxC= 3.17D+03 IAcc=2 IRadAn= 4 AccDes= 0.00D+00
Harris functional with IExCor= 402 and IRadAn= 4 diagonalized for initial guess.
HarFok: IExCor= 402 AccDes= 0.00D+00 IRadAn= 4 IDoV= 1 UseB2=F ITyADJ=14
ICtDFT= 3500011 ScaDFX= 1.000000 1.000000 1.000000 1.000000
FoFCou: FMM=F IPFlag= 0 FMFlag= 100000 FMFlg1= 0
NFxFlg= 0 DoJE=T BraDBF=F KetDBF=T FulRan=T
wScrn= 0.000000 ICntrl= 500 IOpCl= 0 I1Cent= 200000004 NGrid= 0
NMat0= 1 NMatS0= 1 NMatT0= 0 NMatD0= 1 NMtDS0= 0 NMtDT0= 0
Petite list used in FoFCou.
Requested convergence on RMS density matrix=1.00D-08 within 128 cycles.
Requested convergence on MAX density matrix=1.00D-06.
Requested convergence on energy=1.00D-06.
No special actions if energy rises.
SCF Done: E(RB3LYP) = -900.298495135 A.U. after 15 cycles
NFock= 15 Conv=0.35D-08 -V/T= 2.0030

****

Population analysis using the SCF density.

****

Orbital symmetries:
Occupied (A) (A) (A) (A) (A) (A) (A) (A) (A) (A) (A) (A)
(A) (A) (A) (A) (A) (A) (A) (A) (A) (A) (A) (A)
(A) (A) (A) (A) (A) (A) (A) (A) (A) (A) (A) (A)
(A) (A) (A) (A) (A) (A) (A) (A) (A) (A) (A) (A)
(A) (A) (A)
Virtual (A) (A) (A) (A) (A) (A) (A) (A) (A) (A) (A) (A)
(A) (A) (A) (A) (A) (A) (A) (A) (A) (A) (A) (A)
(A) (A) (A) (A) (A) (A) (A) (A) (A) (A) (A) (A)
(A) (A) (A) (A) (A) (A) (A) (A) (A) (A) (A) (A)
(A) (A) (A) (A) (A) (A) (A) (A) (A) (A) (A) (A)

(A) (A) (A) (A) (A) (A) (A) (A) (A) (A) (A) (A)
(A) (A) (A) (A) (A) (A) (A) (A) (A) (A) (A) (A)
(A) (A) (A) (A) (A) (A) (A) (A) (A) (A) (A) (A)
(A) (A) (A) (A) (A) (A) (A) (A) (A) (A) (A) (A)
(A) (A) (A) (A) (A) (A) (A) (A) (A) (A) (A) (A)
(A) (A) (A) (A) (A) (A) (A) (A) (A) (A) (A) (A)
(A) (A) (A) (A) (A) (A) (A) (A) (A) (A) (A) (A)
(A) (A) (A) (A) (A) (A) (A) (A) (A) (A) (A) (A)
(A) (A) (A) (A) (A) (A) (A) (A) (A) (A) (A) (A)
(A) (A) (A) (A) (A) (A) (A) (A) (A) (A) (A) (A)
(A) (A) (A) (A) (A) (A) (A) (A) (A) (A) (A) (A)
(A) (A) (A) (A) (A) (A) (A) (A) (A) (A) (A) (A)
(A) (A) (A) (A) (A) (A) (A) (A) (A) (A) (A) (A)
(A) (A) (A) (A) (A) (A) (A) (A) (A) (A) (A) (A)
(A) (A) (A) (A) (A) (A) (A) (A) (A) (A) (A) (A)
(A) (A) (A) (A) (A) (A) (A) (A) (A) (A) (A) (A)
(A) (A) (A) (A) (A) (A) (A) (A) (A) (A) (A) (A)
(A) (A) (A) (A) (A) (A) (A) (A) (A) (A) (A) (A)
(A) (A) (A) (A) (A) (A) (A) (A) (A) (A) (A) (A)
(A) (A) (A) (A) (A) (A) (A) (A) (A) (A) (A) (A)
(A) (A) (A) (A) (A) (A) (A) (A) (A) (A) (A) (A)
(A) (A) (A) (A) (A) (A) (A) (A) (A) (A) (A) (A)
(A) (A) (A) (A) (A) (A) (A) (A) (A) (A) (A) (A)
(A) (A) (A) (A) (A) (A) (A) (A) (A) (A) (A) (A)
(A) (A) (A) (A) (A) (A) (A) (A) (A) (A) (A) (A)
(A) (A) (A) (A) (A) (A) (A) (A) (A) (A) (A) (A)
(A) (A) (A) (A) (A) (A) (A) (A) (A) (A) (A) (A)
(A) (A) (A) (A) (A) (A) (A) (A) (A) (A) (A) (A)
(A) (A) (A) (A) (A) (A) (A) (A) (A) (A) (A) (A)
(A) (A) (A) (A) (A) (A) (A) (A) (A) (A) (A) (A)
(A) (A) (A) (A) (A)
The electronic state is 1-A.
Alpha occ. eigenvalues -- -89.14370 -19.33400 -10.41245 -10.37898 -10.36931
Alpha occ. eigenvalues -- -10.36721 -10.34241 -10.34177 -10.32938 -10.32699
Alpha occ. eigenvalues -- -10.32478 -10.30763 -10.30470 -8.21980 -6.18392
Alpha occ. eigenvalues --   -6.18040 -6.17511 -1.24180 -1.03702 -0.99692
Alpha occ. eigenvalues --   -0.98864 -0.92045 -0.91082 -0.86323 -0.84438
Alpha occ. eigenvalues --   -0.78264 -0.76745 -0.75803 -0.74294 -0.69648
Alpha occ. eigenvalues --   -0.66778 -0.63869 -0.62999 -0.61497 -0.60346
Alpha occ. eigenvalues --   -0.60067 -0.58422 -0.57827 -0.55754 -0.55406
Alpha occ. eigenvalues --   -0.54065 -0.53222 -0.52418 -0.51711 -0.50738
Alpha occ. eigenvalues --   -0.50628 -0.50218 -0.47899 -0.43579 -0.42305
Alpha occ. eigenvalues --   -0.41454
Alpha virt. eigenvalues --   -0.23008 -0.19746 -0.16671 -0.14675 -0.13082
Alpha virt. eigenvalues --   -0.11965 -0.10385 -0.09703 -0.09498 -0.08608
Alpha virt. eigenvalues --   -0.07691 -0.07492 -0.07094 -0.06198 -0.05892
Alpha virt. eigenvalues --   -0.05387 -0.05135 -0.04796 -0.04063 -0.03670
Alpha virt. eigenvalues --   -0.03506 -0.03036 -0.02659 -0.02047 -0.01684
Alpha virt. eigenvalues --   -0.01394 -0.00801   0.00079   0.00163   0.01036
Alpha virt. eigenvalues --   0.01353   0.01852   0.02324   0.02446   0.03200
Alpha virt. eigenvalues --   0.03432   0.04005   0.04686   0.05378   0.05478
Alpha virt. eigenvalues --   0.05673   0.06290   0.06579   0.06909   0.07317
Alpha virt. eigenvalues --   0.07638   0.07825   0.08360   0.08772   0.08845
Alpha virt. eigenvalues --   0.09187   0.09642   0.09850   0.10085   0.10604
Alpha virt. eigenvalues --   0.11235   0.11514   0.11671   0.12276   0.12638

Alpha virt. eigenvalues --	0.12921	0.13284	0.13699	0.14238	0.14835
Alpha virt. eigenvalues --	0.15469	0.16026	0.16559	0.16943	0.17458
Alpha virt. eigenvalues --	0.18004	0.18643	0.19415	0.19737	0.19962
Alpha virt. eigenvalues --	0.21186	0.21723	0.22758	0.23745	0.24429
Alpha virt. eigenvalues --	0.25111	0.26558	0.27792	0.28104	0.29257
Alpha virt. eigenvalues --	0.29809	0.30963	0.31543	0.33278	0.34169
Alpha virt. eigenvalues --	0.34622	0.35683	0.36389	0.36827	0.37436
Alpha virt. eigenvalues --	0.37941	0.38366	0.38878	0.39402	0.39980
Alpha virt. eigenvalues --	0.40386	0.40894	0.41882	0.42139	0.43113
Alpha virt. eigenvalues --	0.43618	0.44472	0.45225	0.45624	0.46261
Alpha virt. eigenvalues --	0.46869	0.47367	0.48005	0.48297	0.49274
Alpha virt. eigenvalues --	0.49343	0.49806	0.50247	0.50468	0.51288
Alpha virt. eigenvalues --	0.52258	0.52383	0.52812	0.53291	0.53904
Alpha virt. eigenvalues --	0.54155	0.55037	0.55722	0.56223	0.56523
Alpha virt. eigenvalues --	0.57415	0.57774	0.58603	0.59064	0.59613
Alpha virt. eigenvalues --	0.60635	0.61129	0.61482	0.62214	0.63316
Alpha virt. eigenvalues --	0.65078	0.65187	0.66022	0.66554	0.67002
Alpha virt. eigenvalues --	0.68516	0.69808	0.70601	0.71366	0.71964
Alpha virt. eigenvalues --	0.72341	0.73494	0.75442	0.76385	0.77112
Alpha virt. eigenvalues --	0.79104	0.80727	0.81419	0.81827	0.84555
Alpha virt. eigenvalues --	0.85029	0.86093	0.86453	0.87061	0.89073
Alpha virt. eigenvalues --	0.90599	0.91245	0.92351	0.92879	0.93042
Alpha virt. eigenvalues --	0.94066	0.95741	0.96259	0.96565	0.97869
Alpha virt. eigenvalues --	1.00306	1.01359	1.02087	1.03050	1.03604
Alpha virt. eigenvalues --	1.04210	1.05194	1.05607	1.06051	1.08622
Alpha virt. eigenvalues --	1.09934	1.10525	1.11391	1.11912	1.13055
Alpha virt. eigenvalues --	1.13807	1.14113	1.15239	1.16286	1.16655
Alpha virt. eigenvalues --	1.18020	1.18219	1.18513	1.19766	1.20886
Alpha virt. eigenvalues --	1.21433	1.21525	1.21984	1.22596	1.23155
Alpha virt. eigenvalues --	1.23918	1.25240	1.25868	1.26469	1.26965
Alpha virt. eigenvalues --	1.27370	1.27858	1.28929	1.30637	1.31298
Alpha virt. eigenvalues --	1.32582	1.33660	1.33822	1.35771	1.36983
Alpha virt. eigenvalues --	1.40083	1.40091	1.42745	1.43508	1.44211
Alpha virt. eigenvalues --	1.45814	1.47114	1.49004	1.49663	1.50656
Alpha virt. eigenvalues --	1.51894	1.53710	1.55042	1.56116	1.58131
Alpha virt. eigenvalues --	1.61298	1.62567	1.64239	1.64814	1.66541
Alpha virt. eigenvalues --	1.68353	1.69437	1.71452	1.78014	1.79783
Alpha virt. eigenvalues --	1.83604	1.85087	1.86479	1.87716	1.88448
Alpha virt. eigenvalues --	1.92962	1.98025	2.05437	2.13298	2.15748
Alpha virt. eigenvalues --	2.25022	2.29451	2.30937	2.41789	2.46196
Alpha virt. eigenvalues --	2.50477	2.54316	2.57553	2.59398	2.61646
Alpha virt. eigenvalues --	2.64091	2.65628	2.66471	2.67601	2.70198
Alpha virt. eigenvalues --	2.70396	2.73206	2.73355	2.74273	2.76845
Alpha virt. eigenvalues --	2.79988	2.83306	2.86108	2.87543	2.88617
Alpha virt. eigenvalues --	2.90087	2.91845	2.96337	2.96857	2.98528
Alpha virt. eigenvalues --	2.99327	3.00310	3.02586	3.05204	3.06004
Alpha virt. eigenvalues --	3.08432	3.09992	3.10632	3.13283	3.14140
Alpha virt. eigenvalues --	3.15096	3.16367	3.16698	3.17025	3.18082
Alpha virt. eigenvalues --	3.18535	3.19248	3.21404	3.21920	3.23252
Alpha virt. eigenvalues --	3.24755	3.25568	3.26463	3.27860	3.28448
Alpha virt. eigenvalues --	3.31247	3.32585	3.33672	3.34770	3.36443
Alpha virt. eigenvalues --	3.37155	3.39552	3.39884	3.40466	3.41468
Alpha virt. eigenvalues --	3.42419	3.42853	3.43481	3.43880	3.44486
Alpha virt. eigenvalues --	3.45812	3.46819	3.48232	3.50632	3.51397
Alpha virt. eigenvalues --	3.53464	3.54585	3.54756	3.56584	3.56668

Alpha virt. eigenvalues -- 3.57997 3.58505 3.60423 3.61028 3.62774
Alpha virt. eigenvalues -- 3.65119 3.65995 3.67731 3.70850 3.71923
Alpha virt. eigenvalues -- 3.73711 3.75340 3.76201 3.76328 3.79325
Alpha virt. eigenvalues -- 3.80006 3.81992 3.82394 3.84261 3.85114
Alpha virt. eigenvalues -- 3.85780 3.86766 3.88479 3.89830 3.90379
Alpha virt. eigenvalues -- 3.92281 3.92576 3.94698 3.95100 3.96686
Alpha virt. eigenvalues -- 3.97465 3.99222 4.00905 4.02902 4.04376
Alpha virt. eigenvalues -- 4.06199 4.08517 4.09216 4.10132 4.12623
Alpha virt. eigenvalues -- 4.14213 4.14691 4.16525 4.17304 4.23656
Alpha virt. eigenvalues -- 4.26607 4.27319 4.30598 4.38291 4.52512
Alpha virt. eigenvalues -- 4.53779 4.57641 4.58771 4.66957 4.69217
Alpha virt. eigenvalues -- 4.89880 4.90314 4.90870 4.91742 4.93463
Alpha virt. eigenvalues -- 4.95697 4.99986 5.01787 5.03714 5.04374
Alpha virt. eigenvalues -- 5.12952 5.14956 5.37288 6.74601 6.90150
Alpha virt. eigenvalues -- 6.93013 7.00668 7.25766 8.17895 17.35064
Alpha virt. eigenvalues -- 17.49648 17.65088 23.46337 23.54704 23.67290
Alpha virt. eigenvalues -- 23.84932 23.86102 23.86652 23.88263 23.94309
Alpha virt. eigenvalues -- 23.95318 23.99086 24.07014 49.87207 189.33222

Condensed to atoms (all electrons):

		1	2	3	4	5	6
1	C	5.870158	-0.261471	-0.688679	0.530121	0.148050	0.002270
2	C	-0.261471	6.149082	0.416185	-0.700562	-0.131881	0.000900
3	S	-0.688679	0.416185	18.078275	-1.087420	-0.311393	0.013143
4	C	0.530121	-0.700562	-1.087420	6.758058	0.292898	-0.058335
5	C	0.148050	-0.131881	-0.311393	0.292898	5.385836	0.411638
6	H	0.002270	0.000900	0.013143	-0.058335	0.411638	0.524987
7	H	-0.001551	0.004392	-0.014437	0.016264	0.398693	-0.021272
8	H	-0.025388	0.024085	0.032175	-0.011813	0.360931	-0.024222
9	C	-0.004816	0.104178	0.298270	-0.265108	-0.246541	0.013744
10	H	0.004719	-0.018583	0.001680	-0.000065	0.017042	0.000316
11	H	0.004163	-0.008919	-0.018798	-0.004697	0.016151	0.000036
12	H	-0.009084	0.024793	-0.001418	0.016997	-0.025202	0.000027
13	O	-0.054205	0.060123	0.058362	0.183253	-0.077866	0.009067
14	C	-0.033159	0.297318	-1.287500	-0.230003	-0.109374	0.005955
15	C	-0.608102	0.318545	1.346426	-0.380820	-0.086179	0.003237
16	C	0.762924	-0.441378	-1.283765	0.642604	0.247236	-0.008964
17	C	-0.066160	0.034086	0.104250	-0.030176	-0.012696	-0.000013
18	C	-0.000233	-0.116531	-0.035519	0.154179	0.047551	-0.003636
19	C	0.008466	-0.002536	-0.047880	-0.007124	0.016955	-0.000047
20	H	-0.000718	0.000756	0.002522	-0.001160	0.000131	0.000001
21	H	0.036229	-0.017769	-0.043794	0.021962	-0.008023	-0.000038
22	H	0.000299	0.000014	-0.002550	0.000100	-0.000083	0.000000
23	H	-0.000089	0.000002	0.000730	-0.000055	0.000016	0.000000
24	H	-0.001338	-0.000271	0.033856	-0.002960	0.000760	0.000000
25	H	0.406071	0.013209	-0.010768	-0.021894	0.001488	-0.000034
26	H	-0.003982	0.429223	0.017171	-0.092862	0.003341	0.001123

		7	8	9	10	11	12
1	C	-0.001551	-0.025388	-0.004816	0.004719	0.004163	-0.009084
2	C	0.004392	0.024085	0.104178	-0.018583	-0.008919	0.024793
3	S	-0.014437	0.032175	0.298270	0.001680	-0.018798	-0.001418
4	C	0.016264	-0.011813	-0.265108	-0.000065	-0.004697	0.016997
5	C	0.398693	0.360931	-0.246541	0.017042	0.016151	-0.025202
6	H	-0.021272	-0.024222	0.013744	0.000316	0.000036	0.000027
7	H	0.516359	-0.020517	-0.014105	-0.000118	-0.000165	0.001922
8	H	-0.020517	0.543505	0.007743	0.000032	-0.000275	0.000030

9	C	-0.014105	0.007743	5.366868	0.374173	0.404179	0.386204
10	H	-0.000118	0.000032	0.374173	0.527164	-0.024384	-0.021424
11	H	-0.000165	-0.000275	0.404179	-0.024384	0.530719	-0.021085
12	H	0.001922	0.000030	0.386204	-0.021424	-0.021085	0.520414
13	O	-0.006579	-0.000431	-0.089605	0.010055	-0.005913	-0.007660
14	C	0.006422	-0.005693	0.098679	-0.002821	0.006868	0.001105
15	C	0.000224	0.001473	0.053034	-0.000355	-0.000295	0.001854
16	C	-0.001563	-0.007183	-0.160278	0.002034	-0.001355	-0.001616
17	C	0.000189	0.000511	0.004830	0.000004	0.000058	0.000049
18	C	-0.000834	0.008518	-0.030750	0.000294	-0.000372	-0.000161
19	C	-0.000115	-0.001400	-0.003284	-0.000005	0.000016	0.000014
20	H	0.000000	0.000045	-0.000010	0.000000	0.000000	0.000000
21	H	0.000031	-0.000054	0.003133	0.000000	0.000002	-0.000003
22	H	0.000000	0.000000	0.000024	0.000000	0.000000	0.000000
23	H	0.000000	0.000000	-0.000006	0.000000	0.000000	0.000000
24	H	-0.000001	0.000008	-0.000041	0.000000	-0.000001	-0.000001
25	H	0.000017	0.000093	0.001055	-0.000015	-0.000039	0.000020
26	H	-0.000137	0.000072	0.009212	0.001466	-0.000001	-0.000146
		13	14	15	16	17	18
1	C	-0.054205	-0.033159	-0.608102	0.762924	-0.066160	-0.000233
2	C	0.060123	0.297318	0.318545	-0.441378	0.034086	-0.116531
3	S	0.058362	-1.287500	1.346426	-1.283765	0.104250	-0.035519
4	C	0.183253	-0.230003	-0.380820	0.642604	-0.030176	0.154179
5	C	-0.077866	-0.109374	-0.086179	0.247236	-0.012696	0.047551
6	H	0.009067	0.005955	0.003237	-0.008964	-0.000013	-0.003636
7	H	-0.006579	0.006422	0.000224	-0.001563	0.000189	-0.000834
8	H	-0.000431	-0.005693	0.001473	-0.007183	0.000511	0.008518
9	C	-0.089605	0.098679	0.053034	-0.160278	0.004830	-0.030750
10	H	0.010055	-0.002821	-0.000355	0.002034	0.000004	0.000294
11	H	-0.005913	0.006868	-0.000295	-0.001355	0.000058	-0.000372
12	H	-0.007660	0.001105	0.001854	-0.001616	0.000049	-0.000161
13	O	8.096476	0.250428	0.034804	-0.195275	0.009629	-0.033444
14	C	0.250428	10.317868	-1.653629	-0.323725	-0.204772	-0.684717
15	C	0.034804	-1.653629	8.968634	-2.405806	0.577940	-0.674348
16	C	-0.195275	-0.323725	-2.405806	9.659310	-0.742181	-0.226566
17	C	0.009629	-0.204772	0.577940	-0.742181	5.476529	0.269309
18	C	-0.033444	-0.684717	-0.674348	-0.226566	0.269309	6.913697
19	C	-0.001531	-0.911314	0.217275	0.131687	0.391810	0.527674
20	H	0.000027	0.032602	0.007764	-0.023257	0.006201	0.357384
21	H	0.000117	-0.046330	-0.030521	0.465099	-0.003930	-0.013805
22	H	-0.000008	0.020156	-0.012605	-0.000113	0.369865	0.007027
23	H	0.000004	-0.003125	0.011205	0.015845	-0.016695	-0.023046
24	H	-0.001046	-0.118180	0.428081	-0.015672	0.037121	-0.009670
25	H	0.000212	0.054508	0.009248	-0.049250	-0.000992	-0.008714
26	H	0.006341	-0.001854	0.000249	0.001360	0.000089	-0.002717
		19	20	21	22	23	24
1	C	0.008466	-0.000718	0.036229	0.000299	-0.000089	-0.001338
2	C	-0.002536	0.000756	-0.017769	0.000014	0.000002	-0.000271
3	S	-0.047880	0.002522	-0.043794	-0.002550	0.000730	0.033856
4	C	-0.007124	-0.001160	0.021962	0.000100	-0.000055	-0.002960
5	C	0.016955	0.000131	-0.008023	-0.000083	0.000016	0.000760
6	H	-0.000047	0.000001	-0.000038	0.000000	0.000000	0.000000
7	H	-0.000115	0.000000	0.000031	0.000000	0.000000	-0.000001
8	H	-0.001400	0.000045	-0.000054	0.000000	0.000000	0.000008
9	C	-0.003284	-0.000010	0.003133	0.000024	-0.000006	-0.000041

10	H	-0.000005	0.000000	0.000000	0.000000	0.000000	0.000000
11	H	0.000016	0.000000	0.000002	0.000000	0.000000	-0.000001
12	H	0.000014	0.000000	-0.000003	0.000000	0.000000	-0.000001
13	O	-0.001531	0.000027	0.000117	-0.000008	0.000004	-0.001046
14	C	-0.911314	0.032602	-0.046330	0.020156	-0.003125	-0.118180
15	C	0.217275	0.007764	-0.030521	-0.012605	0.011205	0.428081
16	C	0.131687	-0.023257	0.465099	-0.000113	0.015845	-0.015672
17	C	0.391810	0.006201	-0.003930	0.369865	-0.016695	0.037121
18	C	0.527674	0.357384	-0.013805	0.007027	-0.023046	-0.009670
19	C	5.334179	-0.010804	0.007568	-0.008973	0.389062	0.017144
20	H	-0.010804	0.508204	-0.003725	-0.000152	-0.004984	0.000064
21	H	0.007568	-0.003725	0.495849	0.000065	-0.000189	-0.000085
22	H	-0.008973	-0.000152	0.000065	0.506095	-0.005114	-0.004673
23	H	0.389062	-0.004984	-0.000189	-0.005114	0.501749	-0.000165
24	H	0.017144	0.000064	-0.000085	-0.004673	-0.000165	0.497564
25	H	-0.001037	0.000004	-0.000139	0.000000	0.000000	-0.000055
26	H	0.000020	-0.000001	0.000200	0.000000	0.000000	0.000002

		25	26
1	C	0.406071	-0.003982
2	C	0.013209	0.429223
3	S	-0.010768	0.017171
4	C	-0.021894	-0.092862
5	C	0.001488	0.003341
6	H	-0.000034	0.001123
7	H	0.000017	-0.000137
8	H	0.000093	0.000072
9	C	0.001055	0.009212
10	H	-0.000015	0.001466
11	H	-0.000039	-0.000001
12	H	0.000020	-0.000146
13	O	0.000212	0.006341
14	C	0.054508	-0.001854
15	C	0.009248	0.000249
16	C	-0.049250	0.001360
17	C	-0.000992	0.000089
18	C	-0.008714	-0.002717
19	C	-0.001037	0.000020
20	H	0.000004	-0.000001
21	H	-0.000139	0.000200
22	H	0.000000	0.000000
23	H	0.000000	0.000000
24	H	-0.000055	0.000002
25	H	0.494535	-0.004590
26	H	-0.004590	0.505785

Mulliken charges:

		1
1	C	-0.014493
2	C	-0.176988
3	S	0.430877
4	C	0.278617
5	C	-0.339480
6	H	0.130118
7	H	0.136880
8	H	0.117756
9	C	-0.310780

```
  10  H    0.128793
  11  H    0.124109
  12  H    0.134370
  13  O   -0.245335
  14  C    0.524289
  15  C   -0.127334
  16  C   -0.040154
  17  C   -0.204854
  18  C   -0.420569
  19  C   -0.045820
  20  H    0.129105
  21  H    0.138150
  22  H    0.130625
  23  H    0.134854
  24  H    0.139559
  25  H    0.117067
  26  H    0.130636
Sum of Mulliken charges =   1.00000
Mulliken charges with hydrogens summed into heavy atoms:
             1
   1  C    0.102574
   2  C   -0.046352
   3  S    0.430877
   4  C    0.278617
   5  C    0.045274
   9  C    0.076492
  13  O   -0.245335
  14  C    0.524289
  15  C    0.012225
  16  C    0.097997
  17  C   -0.074229
  18  C   -0.291463
  19  C    0.089035
Electronic spatial extent (au):  <R**2>=           2860.8794
Charge=              1.0000 electrons
Dipole moment (field-independent basis, Debye):
   X=             -0.5636    Y=              0.7437    Z=              1.9212  Tot=              2.1359
Quadrupole moment (field-independent basis, Debye-Ang):
  XX=            -40.5178   YY=            -71.1443   ZZ=            -75.7998
  XY=             -2.4639   XZ=             -4.4038   YZ=              1.2053
Traceless Quadrupole moment (field-independent basis, Debye-Ang):
  XX=             21.9695   YY=             -8.6570   ZZ=            -13.3125
  XY=             -2.4639   XZ=             -4.4038   YZ=              1.2053
Octapole moment (field-independent basis, Debye-Ang**2):
 XXX=             18.7208  YYY=              1.6265  ZZZ=             10.8098  XYY=              6.4962
 XXY=             19.5084  XXZ=            -18.2848  XZZ=            -18.1506  YZZ=             -2.4104
 YYZ=             -0.5882  XYZ=              8.2171
Hexadecapole moment (field-independent basis, Debye-Ang**3):
XXXX=          -2240.1767 YYYY=           -562.5120 ZZZZ=           -350.4796 XXXY=             11.6694
XXXZ=            -58.0375 YYYX=              3.4930 YYYZ=             15.0163 ZZZX=             -3.0851
ZZZY=             -7.6199 XXYY=           -500.2979 XXZZ=           -524.2323 YYZZ=           -158.8690
XXYZ=             32.3651 YYXZ=              0.4952 ZZXY=            -14.7343
N-N= 8.977508680452D+02 E-N=-3.892480984268D+03  KE= 8.975928966246D+02
******************************Gaussian NBO Version 3.1******************************
          N A T U R A L   A T O M I C   O R B I T A L   A N D
```

N A T U R A L   B O N D   O R B I T A L   A N A L Y S I S
*****************************Gaussian NBO Version 3.1******************************

/RESON / : Allow strongly delocalized NBO set
/NAOMO / : Print all MOs in the NAO basis

Analyzing the SCF density

Job title: SOphcat NBOnaomo

Storage needed: 1135936 in NPA,   908443 in NBO (131069952 available)

NATURAL POPULATIONS: Natural atomic orbital occupancies

NAO	Atom	No	lang	Type(AO)	Occupancy	Energy
1	C	1	S	Cor(1S)	1.99888	-10.24683
2	C	1	S	Val(2S)	1.05503	-0.44578
3	C	1	S	Ryd(4S)	0.00233	0.91684
4	C	1	S	Ryd(3S)	0.00043	0.58534
5	C	1	S	Ryd(5S)	0.00001	22.46567
6	C	1	px	Val(2p)	1.10078	-0.30702
7	C	1	px	Ryd(4p)	0.00304	0.54978
8	C	1	px	Ryd(3p)	0.00028	0.38908
9	C	1	px	Ryd(5p)	0.00013	3.14753
10	C	1	py	Val(2p)	1.08389	-0.31648
11	C	1	py	Ryd(4p)	0.00204	0.47360
12	C	1	py	Ryd(3p)	0.00023	0.34661
13	C	1	py	Ryd(5p)	0.00010	2.95945
14	C	1	pz	Val(2p)	1.12526	-0.28831
15	C	1	pz	Ryd(4p)	0.00536	0.81716
16	C	1	pz	Ryd(3p)	0.00031	0.34741
17	C	1	pz	Ryd(5p)	0.00007	3.34640
18	C	1	dxy	Ryd(3d)	0.00177	1.44058
19	C	1	dxy	Ryd(4d)	0.00009	3.35869
20	C	1	dxz	Ryd(3d)	0.00199	1.40585
21	C	1	dxz	Ryd(4d)	0.00034	3.46737
22	C	1	dyz	Ryd(3d)	0.00185	1.20655
23	C	1	dyz	Ryd(4d)	0.00018	3.33027
24	C	1	dx2y2	Ryd(3d)	0.00054	0.99449
25	C	1	dx2y2	Ryd(4d)	0.00011	3.17335
26	C	1	dz2	Ryd(3d)	0.00169	1.37305
27	C	1	dz2	Ryd(4d)	0.00025	3.58331
28	C	2	S	Cor(1S)	1.99889	-10.21256
29	C	2	S	Val(2S)	0.97045	-0.36748
30	C	2	S	Ryd(3S)	0.00274	0.82484
31	C	2	S	Ryd(4S)	0.00014	1.27914
32	C	2	S	Ryd(5S)	0.00001	21.44553
33	C	2	px	Val(2p)	1.05465	-0.26845
34	C	2	px	Ryd(4p)	0.00355	0.69278
35	C	2	px	Ryd(3p)	0.00028	0.43860
36	C	2	px	Ryd(5p)	0.00011	3.13680
37	C	2	py	Val(2p)	0.99145	-0.27671

38	C	2	py	Ryd(4p)	0.00359	0.59760
39	C	2	py	Ryd(3p)	0.00028	0.41806
40	C	2	py	Ryd(5p)	0.00008	2.91654
41	C	2	pz	Val(2p)	1.09842	-0.24341
42	C	2	pz	Ryd(4p)	0.00480	0.70361
43	C	2	pz	Ryd(3p)	0.00042	0.47877
44	C	2	pz	Ryd(5p)	0.00007	3.25659
45	C	2	dxy	Ryd(3d)	0.00145	1.65731
46	C	2	dxy	Ryd(4d)	0.00011	3.34835
47	C	2	dxz	Ryd(3d)	0.00066	1.51030
48	C	2	dxz	Ryd(4d)	0.00036	3.48474
49	C	2	dyz	Ryd(3d)	0.00052	1.39570
50	C	2	dyz	Ryd(4d)	0.00030	3.37485
51	C	2	dx2y2	Ryd(3d)	0.00124	1.20643
52	C	2	dx2y2	Ryd(4d)	0.00006	3.16365
53	C	2	dz2	Ryd(3d)	0.00166	1.49041
54	C	2	dz2	Ryd(4d)	0.00027	3.44690
55	S	3	S	Cor(1S)	2.00000	-87.98245
56	S	3	S	Cor(2S)	1.99882	-9.13015
57	S	3	S	Val(3S)	1.52258	-0.87425
58	S	3	S	Ryd(5S)	0.00335	1.75833
59	S	3	S	Ryd(4S)	0.00015	0.76176
60	S	3	S	Ryd(6S)	0.00001	14.95847
61	S	3	S	Ryd(7S)	0.00000	181.76655
62	S	3	px	Cor(2p)	1.99982	-6.17201
63	S	3	px	Val(3p)	0.87289	-0.35381
64	S	3	px	Ryd(5p)	0.00492	0.74797
65	S	3	px	Ryd(6p)	0.00045	1.88385
66	S	3	px	Ryd(4p)	0.00025	0.49669
67	S	3	px	Ryd(7p)	0.00001	15.45610
68	S	3	py	Cor(2p)	1.99990	-6.16858
69	S	3	py	Val(3p)	1.47572	-0.42618
70	S	3	py	Ryd(5p)	0.00582	0.58418
71	S	3	py	Ryd(6p)	0.00048	1.58775
72	S	3	py	Ryd(4p)	0.00026	0.38390
73	S	3	py	Ryd(7p)	0.00001	16.13224
74	S	3	pz	Cor(2p)	1.99984	-6.17408
75	S	3	pz	Val(3p)	0.82711	-0.37364
76	S	3	pz	Ryd(5p)	0.00417	0.59592
77	S	3	pz	Ryd(6p)	0.00053	1.72152
78	S	3	pz	Ryd(4p)	0.00041	0.47646
79	S	3	pz	Ryd(7p)	0.00000	15.98077
80	S	3	dxy	Ryd(3d)	0.01478	0.88677
81	S	3	dxy	Ryd(4d)	0.00006	2.75718
82	S	3	dxz	Ryd(3d)	0.01555	0.79116
83	S	3	dxz	Ryd(4d)	0.00014	2.28564
84	S	3	dyz	Ryd(3d)	0.01210	0.69237
85	S	3	dyz	Ryd(4d)	0.00004	2.44252
86	S	3	dx2y2	Ryd(3d)	0.01389	0.60107
87	S	3	dx2y2	Ryd(4d)	0.00007	2.31014
88	S	3	dz2	Ryd(3d)	0.00988	0.64017
89	S	3	dz2	Ryd(4d)	0.00007	2.28215
90	C	4	S	Cor(1S)	1.99908	-10.28439

91	C	4	S	Val(2S)	0.93022	-0.39500
92	C	4	S	Ryd(3S)	0.00100	0.93394
93	C	4	S	Ryd(4S)	0.00019	1.73542
94	C	4	S	Ryd(5S)	0.00000	22.60168
95	C	4	px	Val(2p)	0.87667	-0.26416
96	C	4	px	Ryd(3p)	0.00455	0.72784
97	C	4	px	Ryd(4p)	0.00029	1.11977
98	C	4	px	Ryd(5p)	0.00004	2.80905
99	C	4	py	Val(2p)	0.93951	-0.26655
100	C	4	py	Ryd(3p)	0.00372	0.73023
101	C	4	py	Ryd(4p)	0.00032	1.05537
102	C	4	py	Ryd(5p)	0.00003	2.82173
103	C	4	pz	Val(2p)	0.97259	-0.26337
104	C	4	pz	Ryd(3p)	0.00493	0.70182
105	C	4	pz	Ryd(4p)	0.00030	0.99232
106	C	4	pz	Ryd(5p)	0.00003	2.73535
107	C	4	dxy	Ryd(3d)	0.00164	2.02385
108	C	4	dxy	Ryd(4d)	0.00029	3.09934
109	C	4	dxz	Ryd(3d)	0.00044	1.92418
110	C	4	dxz	Ryd(4d)	0.00052	2.97476
111	C	4	dyz	Ryd(3d)	0.00054	1.90777
112	C	4	dyz	Ryd(4d)	0.00038	2.98440
113	C	4	dx2y2	Ryd(3d)	0.00134	1.91809
114	C	4	dx2y2	Ryd(4d)	0.00016	2.96981
115	C	4	dz2	Ryd(3d)	0.00155	1.97738
116	C	4	dz2	Ryd(4d)	0.00016	3.18058

117	C	5	S	Cor(1S)	1.99921	-10.15997
118	C	5	S	Val(2S)	1.08422	-0.39463
119	C	5	S	Ryd(4S)	0.00072	0.93581
120	C	5	S	Ryd(3S)	0.00010	0.55109
121	C	5	S	Ryd(5S)	0.00000	22.77971
122	C	5	px	Val(2p)	1.21637	-0.23730
123	C	5	px	Ryd(4p)	0.00312	0.59137
124	C	5	px	Ryd(3p)	0.00010	0.24982
125	C	5	px	Ryd(5p)	0.00001	3.38657
126	C	5	py	Val(2p)	1.06659	-0.23761
127	C	5	py	Ryd(4p)	0.00206	0.52202
128	C	5	py	Ryd(3p)	0.00021	0.18560
129	C	5	py	Ryd(5p)	0.00004	3.19502
130	C	5	pz	Val(2p)	1.20512	-0.24170
131	C	5	pz	Ryd(4p)	0.00235	0.55685
132	C	5	pz	Ryd(3p)	0.00013	0.20155
133	C	5	pz	Ryd(5p)	0.00001	3.34302
134	C	5	dxy	Ryd(3d)	0.00117	1.37083
135	C	5	dxy	Ryd(4d)	0.00017	3.49916
136	C	5	dxz	Ryd(3d)	0.00152	1.24224
137	C	5	dxz	Ryd(4d)	0.00004	3.36360
138	C	5	dyz	Ryd(3d)	0.00113	1.33460
139	C	5	dyz	Ryd(4d)	0.00008	3.34320
140	C	5	dx2y2	Ryd(3d)	0.00196	1.36248
141	C	5	dx2y2	Ryd(4d)	0.00007	3.36465
142	C	5	dz2	Ryd(3d)	0.00216	1.33886
143	C	5	dz2	Ryd(4d)	0.00006	3.50194

144	H	6	S	Val(1S)	0.76958	-0.09675
145	H	6	S	Ryd(3S)	0.00035	1.43662
146	H	6	S	Ryd(2S)	0.00005	1.29027
147	H	6	px	Ryd(2p)	0.00025	2.50872
148	H	6	px	Ryd(3p)	0.00011	2.77771
149	H	6	py	Ryd(2p)	0.00019	2.50713
150	H	6	py	Ryd(3p)	0.00015	2.75895
151	H	6	pz	Ryd(2p)	0.00015	2.23764
152	H	6	pz	Ryd(3p)	0.00007	2.69486
153	H	7	S	Val(1S)	0.76391	-0.08541
154	H	7	S	Ryd(3S)	0.00060	1.52311
155	H	7	S	Ryd(2S)	0.00006	1.21287
156	H	7	px	Ryd(2p)	0.00012	1.96965
157	H	7	px	Ryd(3p)	0.00005	2.85069
158	H	7	py	Ryd(2p)	0.00024	1.96645
159	H	7	py	Ryd(3p)	0.00006	2.88168
160	H	7	pz	Ryd(2p)	0.00033	2.74703
161	H	7	pz	Ryd(3p)	0.00022	3.05677
162	H	8	S	Val(1S)	0.78474	-0.09713
163	H	8	S	Ryd(2S)	0.00080	1.36051
164	H	8	S	Ryd(3S)	0.00008	1.44789
165	H	8	px	Ryd(2p)	0.00036	2.48626
166	H	8	px	Ryd(3p)	0.00017	3.18053
167	H	8	py	Ryd(2p)	0.00023	1.89385
168	H	8	py	Ryd(3p)	0.00009	3.11715
169	H	8	pz	Ryd(2p)	0.00016	1.77813
170	H	8	pz	Ryd(3p)	0.00006	3.06379
171	C	9	S	Cor(1S)	1.99920	-10.15730
172	C	9	S	Val(2S)	1.08629	-0.39221
173	C	9	S	Ryd(4S)	0.00075	0.95802
174	C	9	S	Ryd(3S)	0.00007	0.52118
175	C	9	S	Ryd(5S)	0.00000	22.78494
176	C	9	px	Val(2p)	1.06682	-0.23427
177	C	9	px	Ryd(4p)	0.00203	0.52944
178	C	9	px	Ryd(3p)	0.00019	0.16872
179	C	9	px	Ryd(5p)	0.00004	3.22953
180	C	9	py	Val(2p)	1.19353	-0.23472
181	C	9	py	Ryd(4p)	0.00317	0.57681
182	C	9	py	Ryd(3p)	0.00011	0.23462
183	C	9	py	Ryd(5p)	0.00001	3.36283
184	C	9	pz	Val(2p)	1.22164	-0.23785
185	C	9	pz	Ryd(4p)	0.00259	0.57415
186	C	9	pz	Ryd(3p)	0.00009	0.19355
187	C	9	pz	Ryd(5p)	0.00001	3.36854
188	C	9	dxy	Ryd(3d)	0.00165	1.37245
189	C	9	dxy	Ryd(4d)	0.00011	3.41184
190	C	9	dxz	Ryd(3d)	0.00076	1.30883
191	C	9	dxz	Ryd(4d)	0.00012	3.36601
192	C	9	dyz	Ryd(3d)	0.00166	1.26045
193	C	9	dyz	Ryd(4d)	0.00003	3.38012
194	C	9	dx2y2	Ryd(3d)	0.00142	1.36085
195	C	9	dx2y2	Ryd(4d)	0.00013	3.40818

196	C	9	dz2	Ryd(3d)	0.00243	1.33918
197	C	9	dz2	Ryd(4d)	0.00004	3.48349
198	H	10	S	Val(1S)	0.77135	-0.09372
199	H	10	S	Ryd(3S)	0.00032	1.44874
200	H	10	S	Ryd(2S)	0.00006	1.27430
201	H	10	px	Ryd(2p)	0.00020	2.59473
202	H	10	px	Ryd(3p)	0.00017	2.84714
203	H	10	py	Ryd(2p)	0.00027	2.30837
204	H	10	py	Ryd(3p)	0.00010	2.79519
205	H	10	pz	Ryd(2p)	0.00013	2.20768
206	H	10	pz	Ryd(3p)	0.00006	2.73645
207	H	11	S	Val(1S)	0.77638	-0.08830
208	H	11	S	Ryd(3S)	0.00069	1.48479
209	H	11	S	Ryd(2S)	0.00007	1.27229
210	H	11	px	Ryd(2p)	0.00026	1.85142
211	H	11	px	Ryd(3p)	0.00007	2.99683
212	H	11	py	Ryd(2p)	0.00028	2.51983
213	H	11	py	Ryd(3p)	0.00019	3.12784
214	H	11	pz	Ryd(2p)	0.00018	1.93484
215	H	11	pz	Ryd(3p)	0.00007	2.98609
216	H	12	S	Val(1S)	0.76496	-0.08227
217	H	12	S	Ryd(3S)	0.00058	1.54187
218	H	12	S	Ryd(2S)	0.00008	1.18184
219	H	12	px	Ryd(2p)	0.00020	1.99403
220	H	12	px	Ryd(3p)	0.00006	2.87517
221	H	12	py	Ryd(2p)	0.00013	1.97086
222	H	12	py	Ryd(3p)	0.00005	2.85001
223	H	12	pz	Ryd(2p)	0.00034	2.73678
224	H	12	pz	Ryd(3p)	0.00021	3.05686
225	O	13	S	Cor(1S)	1.99979	-19.14548
226	O	13	S	Val(2S)	1.71349	-1.11115
227	O	13	S	Ryd(4S)	0.00162	1.71326
228	O	13	S	Ryd(3S)	0.00004	1.49181
229	O	13	S	Ryd(5S)	0.00000	49.37235
230	O	13	px	Val(2p)	1.56956	-0.50278
231	O	13	px	Ryd(3p)	0.00403	0.56745
232	O	13	px	Ryd(4p)	0.00069	1.30846
233	O	13	px	Ryd(5p)	0.00004	4.47858
234	O	13	py	Val(2p)	1.72523	-0.49961
235	O	13	py	Ryd(3p)	0.00290	0.46114
236	O	13	py	Ryd(4p)	0.00061	1.01785
237	O	13	py	Ryd(5p)	0.00004	4.62492
238	O	13	pz	Val(2p)	1.67647	-0.51450
239	O	13	pz	Ryd(3p)	0.00223	0.40824
240	O	13	pz	Ryd(4p)	0.00075	1.00317
241	O	13	pz	Ryd(5p)	0.00009	4.65782
242	O	13	dxy	Ryd(3d)	0.00154	1.71279
243	O	13	dxy	Ryd(4d)	0.00001	6.77461
244	O	13	dxz	Ryd(3d)	0.00546	1.90968
245	O	13	dxz	Ryd(4d)	0.00004	6.86077
246	O	13	dyz	Ryd(3d)	0.00412	1.70135

247	O	13	dyz	Ryd(4d)	0.00002	6.76284
248	O	13	dx2y2	Ryd(3d)	0.00056	1.71851
249	O	13	dx2y2	Ryd(4d)	0.00003	6.78915
250	O	13	dz2	Ryd(3d)	0.00400	1.70703
251	O	13	dz2	Ryd(4d)	0.00002	6.79354
252	C	14	S	Cor(1S)	1.99878	-10.23608
253	C	14	S	Val(2S)	0.96450	-0.38420
254	C	14	S	Ryd(4S)	0.00193	0.98203
255	C	14	S	Ryd(3S)	0.00026	0.76963
256	C	14	S	Ryd(5S)	0.00001	22.32440
257	C	14	px	Val(2p)	1.02980	-0.30420
258	C	14	px	Ryd(4p)	0.00592	0.93859
259	C	14	px	Ryd(3p)	0.00014	0.88607
260	C	14	px	Ryd(5p)	0.00012	3.20618
261	C	14	py	Val(2p)	1.10880	-0.27118
262	C	14	py	Ryd(4p)	0.00357	0.84119
263	C	14	py	Ryd(3p)	0.00018	0.82998
264	C	14	py	Ryd(5p)	0.00014	3.17431
265	C	14	pz	Val(2p)	1.15563	-0.29863
266	C	14	pz	Ryd(3p)	0.00220	0.49841
267	C	14	pz	Ryd(4p)	0.00022	0.73735
268	C	14	pz	Ryd(5p)	0.00007	2.59203
269	C	14	dxy	Ryd(3d)	0.00150	1.88676
270	C	14	dxy	Ryd(4d)	0.00049	3.55064
271	C	14	dxz	Ryd(3d)	0.00124	1.21749
272	C	14	dxz	Ryd(4d)	0.00018	3.36356
273	C	14	dyz	Ryd(3d)	0.00098	1.39468
274	C	14	dyz	Ryd(4d)	0.00029	3.30811
275	C	14	dx2y2	Ryd(3d)	0.00069	1.79630
276	C	14	dx2y2	Ryd(4d)	0.00056	3.54219
277	C	14	dz2	Ryd(3d)	0.00135	1.32254
278	C	14	dz2	Ryd(4d)	0.00015	3.36292
279	C	15	S	Cor(1S)	1.99903	-10.19091
280	C	15	S	Val(2S)	0.94899	-0.32564
281	C	15	S	Ryd(4S)	0.00166	1.02990
282	C	15	S	Ryd(3S)	0.00016	0.67056
283	C	15	S	Ryd(5S)	0.00001	21.31155
284	C	15	px	Val(2p)	1.06144	-0.21841
285	C	15	px	Ryd(4p)	0.00516	0.91018
286	C	15	px	Ryd(3p)	0.00038	0.40266
287	C	15	px	Ryd(5p)	0.00018	3.15615
288	C	15	py	Val(2p)	1.16070	-0.22600
289	C	15	py	Ryd(4p)	0.00637	0.93610
290	C	15	py	Ryd(3p)	0.00016	0.43657
291	C	15	py	Ryd(5p)	0.00007	3.31493
292	C	15	pz	Val(2p)	0.97000	-0.25779
293	C	15	pz	Ryd(4p)	0.00176	0.49497
294	C	15	pz	Ryd(3p)	0.00025	0.28328
295	C	15	pz	Ryd(5p)	0.00006	2.77414
296	C	15	dxy	Ryd(3d)	0.00097	1.94752
297	C	15	dxy	Ryd(4d)	0.00023	3.38002
298	C	15	dxz	Ryd(3d)	0.00067	1.63680
299	C	15	dxz	Ryd(4d)	0.00017	2.96074

300	C	15	dyz	Ryd(3d)	0.00067	1.54040
301	C	15	dyz	Ryd(4d)	0.00010	3.06640
302	C	15	dx2y2	Ryd(3d)	0.00074	1.94833
303	C	15	dx2y2	Ryd(4d)	0.00057	3.41018
304	C	15	dz2	Ryd(3d)	0.00089	1.67687
305	C	15	dz2	Ryd(4d)	0.00007	3.04612
306	C	16	S	Cor(1S)	1.99900	-10.18944
307	C	16	S	Val(2S)	0.94483	-0.32219
308	C	16	S	Ryd(4S)	0.00180	1.04728
309	C	16	S	Ryd(3S)	0.00017	0.87477
310	C	16	S	Ryd(5S)	0.00001	21.26348
311	C	16	px	Val(2p)	1.13551	-0.22083
312	C	16	px	Ryd(4p)	0.00624	1.09174
313	C	16	px	Ryd(3p)	0.00026	0.52732
314	C	16	px	Ryd(5p)	0.00011	3.27310
315	C	16	py	Val(2p)	1.07770	-0.22331
316	C	16	py	Ryd(4p)	0.00402	0.85865
317	C	16	py	Ryd(3p)	0.00030	0.56803
318	C	16	py	Ryd(5p)	0.00013	3.16108
319	C	16	pz	Val(2p)	0.99980	-0.25809
320	C	16	pz	Ryd(4p)	0.00210	0.53833
321	C	16	pz	Ryd(3p)	0.00033	0.34380
322	C	16	pz	Ryd(5p)	0.00005	2.81463
323	C	16	dxy	Ryd(3d)	0.00083	2.02082
324	C	16	dxy	Ryd(4d)	0.00056	3.49259
325	C	16	dxz	Ryd(3d)	0.00069	1.47643
326	C	16	dxz	Ryd(4d)	0.00018	3.10288
327	C	16	dyz	Ryd(3d)	0.00060	1.52629
328	C	16	dyz	Ryd(4d)	0.00015	3.10290
329	C	16	dx2y2	Ryd(3d)	0.00099	1.92758
330	C	16	dx2y2	Ryd(4d)	0.00025	3.43763
331	C	16	dz2	Ryd(3d)	0.00086	1.66303
332	C	16	dz2	Ryd(4d)	0.00010	3.10667
333	C	17	S	Cor(1S)	1.99915	-10.17831
334	C	17	S	Val(2S)	0.95729	-0.31783
335	C	17	S	Ryd(4S)	0.00150	1.04891
336	C	17	S	Ryd(3S)	0.00010	0.74594
337	C	17	S	Ryd(5S)	0.00000	21.05189
338	C	17	px	Val(2p)	1.11217	-0.20148
339	C	17	px	Ryd(4p)	0.00555	0.97939
340	C	17	px	Ryd(3p)	0.00026	0.39539
341	C	17	px	Ryd(5p)	0.00012	3.22041
342	C	17	py	Val(2p)	1.08878	-0.20407
343	C	17	py	Ryd(4p)	0.00495	0.90112
344	C	17	py	Ryd(3p)	0.00028	0.38684
345	C	17	py	Ryd(5p)	0.00014	3.13801
346	C	17	pz	Val(2p)	1.00351	-0.24423
347	C	17	pz	Ryd(4p)	0.00186	0.54623
348	C	17	pz	Ryd(3p)	0.00018	0.24416
349	C	17	pz	Ryd(5p)	0.00006	2.73333
350	C	17	dxy	Ryd(3d)	0.00090	2.05754
351	C	17	dxy	Ryd(4d)	0.00052	3.46223
352	C	17	dxz	Ryd(3d)	0.00055	1.48816

353	C	17	dxz	Ryd(4d)	0.00017	3.07620
354	C	17	dyz	Ryd(3d)	0.00067	1.58060
355	C	17	dyz	Ryd(4d)	0.00010	3.07791
356	C	17	dx2y2	Ryd(3d)	0.00090	1.89175
357	C	17	dx2y2	Ryd(4d)	0.00032	3.39012
358	C	17	dz2	Ryd(3d)	0.00096	1.71608
359	C	17	dz2	Ryd(4d)	0.00005	3.07404
360	C	18	S	Cor(1S)	1.99915	-10.18081
361	C	18	S	Val(2S)	0.95716	-0.31966
362	C	18	S	Ryd(4S)	0.00142	1.14207
363	C	18	S	Ryd(3S)	0.00012	0.69710
364	C	18	S	Ryd(5S)	0.00001	21.02825
365	C	18	px	Val(2p)	1.04870	-0.19671
366	C	18	px	Ryd(4p)	0.00392	0.91784
367	C	18	px	Ryd(3p)	0.00034	0.43288
368	C	18	px	Ryd(5p)	0.00018	3.14235
369	C	18	py	Val(2p)	1.15851	-0.21108
370	C	18	py	Ryd(4p)	0.00653	0.98471
371	C	18	py	Ryd(3p)	0.00018	0.37151
372	C	18	py	Ryd(5p)	0.00007	3.25235
373	C	18	pz	Val(2p)	0.99254	-0.24488
374	C	18	pz	Ryd(4p)	0.00159	0.53685
375	C	18	pz	Ryd(3p)	0.00021	0.24810
376	C	18	pz	Ryd(5p)	0.00007	2.73146
377	C	18	dxy	Ryd(3d)	0.00093	1.90601
378	C	18	dxy	Ryd(4d)	0.00030	3.42173
379	C	18	dxz	Ryd(3d)	0.00053	1.65998
380	C	18	dxz	Ryd(4d)	0.00017	3.02668
381	C	18	dyz	Ryd(3d)	0.00080	1.52657
382	C	18	dyz	Ryd(4d)	0.00010	3.13548
383	C	18	dx2y2	Ryd(3d)	0.00087	1.99980
384	C	18	dx2y2	Ryd(4d)	0.00053	3.46665
385	C	18	dz2	Ryd(3d)	0.00087	1.67295
386	C	18	dz2	Ryd(4d)	0.00004	3.08484
387	C	19	S	Cor(1S)	1.99917	-10.18304
388	C	19	S	Val(2S)	0.96416	-0.32242
389	C	19	S	Ryd(3S)	0.00117	1.01795
390	C	19	S	Ryd(4S)	0.00015	1.12403
391	C	19	S	Ryd(5S)	0.00000	20.95622
392	C	19	px	Val(2p)	1.15181	-0.20198
393	C	19	px	Ryd(4p)	0.00619	0.97853
394	C	19	px	Ryd(3p)	0.00022	0.44932
395	C	19	px	Ryd(5p)	0.00007	3.26788
396	C	19	py	Val(2p)	1.05757	-0.19770
397	C	19	py	Ryd(4p)	0.00420	0.89413
398	C	19	py	Ryd(3p)	0.00041	0.40119
399	C	19	py	Ryd(5p)	0.00013	3.12223
400	C	19	pz	Val(2p)	0.93393	-0.24033
401	C	19	pz	Ryd(4p)	0.00121	0.52848
402	C	19	pz	Ryd(3p)	0.00022	0.27141
403	C	19	pz	Ryd(5p)	0.00007	2.67944
404	C	19	dxy	Ryd(3d)	0.00086	1.89564
405	C	19	dxy	Ryd(4d)	0.00040	3.42837

406	C	19	dxz	Ryd(3d)	0.00072	1.57499
407	C	19	dxz	Ryd(4d)	0.00008	3.02761
408	C	19	dyz	Ryd(3d)	0.00073	1.77708
409	C	19	dyz	Ryd(4d)	0.00018	3.02518
410	C	19	dx2y2	Ryd(3d)	0.00089	1.92926
411	C	19	dx2y2	Ryd(4d)	0.00040	3.41627
412	C	19	dz2	Ryd(3d)	0.00076	1.63195
413	C	19	dz2	Ryd(4d)	0.00008	3.03896
414	H	20	S	Val(1S)	0.76719	-0.07774
415	H	20	S	Ryd(2S)	0.00038	1.38398
416	H	20	S	Ryd(3S)	0.00014	1.55193
417	H	20	px	Ryd(2p)	0.00013	1.29434
418	H	20	px	Ryd(3p)	0.00006	3.89612
419	H	20	py	Ryd(2p)	0.00045	1.84248
420	H	20	py	Ryd(3p)	0.00028	3.79988
421	H	20	pz	Ryd(2p)	0.00013	0.98227
422	H	20	pz	Ryd(3p)	0.00008	3.71698
423	H	21	S	Val(1S)	0.77227	-0.09878
424	H	21	S	Ryd(2S)	0.00086	1.40745
425	H	21	S	Ryd(3S)	0.00012	1.54391
426	H	21	px	Ryd(2p)	0.00041	1.50927
427	H	21	px	Ryd(3p)	0.00017	3.92701
428	H	21	py	Ryd(2p)	0.00022	1.37613
429	H	21	py	Ryd(3p)	0.00013	3.87868
430	H	21	pz	Ryd(2p)	0.00025	1.07947
431	H	21	pz	Ryd(3p)	0.00008	3.68495
432	H	22	S	Val(1S)	0.76423	-0.07390
433	H	22	S	Ryd(2S)	0.00037	1.33004
434	H	22	S	Ryd(3S)	0.00014	1.56596
435	H	22	px	Ryd(2p)	0.00033	1.64114
436	H	22	px	Ryd(3p)	0.00019	3.84764
437	H	22	py	Ryd(2p)	0.00022	1.42204
438	H	22	py	Ryd(3p)	0.00012	3.84917
439	H	22	pz	Ryd(2p)	0.00015	1.03411
440	H	22	pz	Ryd(3p)	0.00010	3.71157
441	H	23	S	Val(1S)	0.76793	-0.07280
442	H	23	S	Ryd(3S)	0.00021	1.73972
443	H	23	S	Ryd(2S)	0.00011	1.18521
444	H	23	px	Ryd(2p)	0.00041	1.80743
445	H	23	px	Ryd(3p)	0.00026	3.81442
446	H	23	py	Ryd(2p)	0.00018	1.32446
447	H	23	py	Ryd(3p)	0.00009	3.86748
448	H	23	pz	Ryd(2p)	0.00007	0.92093
449	H	23	pz	Ryd(3p)	0.00006	3.72599
450	H	24	S	Val(1S)	0.76500	-0.09225
451	H	24	S	Ryd(3S)	0.00073	1.50191
452	H	24	S	Ryd(2S)	0.00012	1.37538
453	H	24	px	Ryd(2p)	0.00018	1.22565
454	H	24	px	Ryd(3p)	0.00008	3.88367
455	H	24	py	Ryd(2p)	0.00046	1.72571

456	H	24	py	Ryd(3p)	0.00025	3.91383
457	H	24	pz	Ryd(2p)	0.00020	1.07516
458	H	24	pz	Ryd(3p)	0.00005	3.60465
459	H	25	S	Val(1S)	0.73294	-0.10875
460	H	25	S	Ryd(3S)	0.00065	1.53532
461	H	25	S	Ryd(2S)	0.00024	1.09885
462	H	25	px	Ryd(2p)	0.00011	1.21641
463	H	25	px	Ryd(3p)	0.00014	3.75539
464	H	25	py	Ryd(2p)	0.00007	1.09023
465	H	25	py	Ryd(3p)	0.00008	3.63860
466	H	25	pz	Ryd(2p)	0.00060	1.76665
467	H	25	pz	Ryd(3p)	0.00032	3.90272
468	H	26	S	Val(1S)	0.74050	-0.10345
469	H	26	S	Ryd(3S)	0.00043	1.51088
470	H	26	S	Ryd(2S)	0.00008	1.28204
471	H	26	px	Ryd(2p)	0.00025	1.34617
472	H	26	px	Ryd(3p)	0.00014	3.77361
473	H	26	py	Ryd(2p)	0.00025	1.23346
474	H	26	py	Ryd(3p)	0.00010	3.67392
475	H	26	pz	Ryd(2p)	0.00028	1.48854
476	H	26	pz	Ryd(3p)	0.00017	3.87557

WARNING: 1 low occupancy (<1.9990e) core orbital found on C 1
1 low occupancy (<1.9990e) core orbital found on C 2
1 low occupancy (<1.9990e) core orbital found on S 3
1 low occupancy (<1.9990e) core orbital found on C 14
1 low occupancy (<1.9990e) core orbital found on C 16

WARNING: Population inversion found on atom C 1
Population inversion found on atom C 2
Population inversion found on atom S 3
Population inversion found on atom C 4
Population inversion found on atom C 5
Population inversion found on atom H 6
Population inversion found on atom H 7
Population inversion found on atom C 9
Population inversion found on atom H 10
Population inversion found on atom H 11
Population inversion found on atom H 12
Population inversion found on atom O 13
Population inversion found on atom C 14
Population inversion found on atom C 15
Population inversion found on atom C 16
Population inversion found on atom C 17
Population inversion found on atom C 18
Population inversion found on atom C 19
Population inversion found on atom H 23
Population inversion found on atom H 24
Population inversion found on atom H 25
Population inversion found on atom H 26

Summary of Natural Population Analysis:

Atom	No	Natural Charge	Core	Valence	Rydberg	Total
C	1	-0.38699	1.99888	4.36497	0.02314	6.38699
C	2	-0.13655	1.99889	4.11498	0.02268	6.13655
S	3	1.21592	9.99838	4.69831	0.08739	14.78408
C	4	0.25951	1.99908	3.71900	0.02241	5.74049
C	5	-0.58871	1.99921	4.57230	0.01720	6.58871
H	6	0.22910	0.00000	0.76958	0.00132	0.77090
H	7	0.23441	0.00000	0.76391	0.00168	0.76559
H	8	0.21331	0.00000	0.78474	0.00195	0.78669
C	9	-0.58487	1.99920	4.56827	0.01740	6.58487
H	10	0.22734	0.00000	0.77135	0.00131	0.77266
H	11	0.22181	0.00000	0.77638	0.00181	0.77819
H	12	0.23338	0.00000	0.76496	0.00166	0.76662
O	13	-0.71337	1.99979	6.68475	0.02883	8.71337
C	14	-0.27969	1.99878	4.25872	0.02219	6.27969
C	15	-0.16146	1.99903	4.14112	0.02130	6.16146
C	16	-0.17758	1.99900	4.15784	0.02074	6.17758
C	17	-0.18102	1.99915	4.16175	0.02012	6.18102
C	18	-0.17584	1.99915	4.15691	0.01978	6.17584
C	19	-0.12577	1.99917	4.10746	0.01914	6.12577
H	20	0.23117	0.00000	0.76719	0.00164	0.76883
H	21	0.22549	0.00000	0.77227	0.00224	0.77451
H	22	0.23414	0.00000	0.76423	0.00163	0.76586
H	23	0.23069	0.00000	0.76793	0.00139	0.76931
H	24	0.23294	0.00000	0.76500	0.00207	0.76706
H	25	0.26486	0.00000	0.73294	0.00221	0.73514
H	26	0.25780	0.00000	0.74050	0.00170	0.74220
* Total *		1.00000	33.98770	67.64736	0.36494	102.00000

Natural Population

Core	33.98770 (99.9638% of 34)
Valence	67.64736 (99.4814% of 68)
Natural Minimal Basis	101.63506 (99.6422% of 102)
Natural Rydberg Basis	0.36494 (0.3578% of 102)

Atom	No	Natural Electron Configuration
C	1	[core]2S(1.06)2p(3.31)3d(0.01)4p(0.01)
C	2	[core]2S(0.97)2p(3.14)3d(0.01)4p(0.01)
S	3	[core]3S(1.52)3p(3.18)3d(0.07)5p(0.01)
C	4	[core]2S(0.93)2p(2.79)3p(0.01)3d(0.01)
C	5	[core]2S(1.08)2p(3.49)3d(0.01)4p(0.01)
H	6	1S(0.77)
H	7	1S(0.76)
H	8	1S(0.78)
C	9	[core]2S(1.09)2p(3.48)3d(0.01)4p(0.01)
H	10	1S(0.77)
H	11	1S(0.78)

H 12 1S(0.76)
O 13 [core]2S(1.71)2p(4.97)3p(0.01)3d(0.02)
C 14 [core]2S(0.96)2p(3.29)3d(0.01)4p(0.01)
C 15 [core]2S(0.95)2p(3.19)4p(0.01)
C 16 [core]2S(0.94)2p(3.21)4p(0.01)
C 17 [core]2S(0.96)2p(3.20)4p(0.01)
C 18 [core]2S(0.96)2p(3.20)4p(0.01)
C 19 [core]2S(0.96)2p(3.14)4p(0.01)
H 20 1S(0.77)
H 21 1S(0.77)
H 22 1S(0.76)
H 23 1S(0.77)
H 24 1S(0.76)
H 25 1S(0.73)
H 26 1S(0.74)

2-oxo-5,5-dimethyl-2-phenyl-5*H*-λ^5-1,2-oxathiol-2-ium (Ba).

****

Gaussian 09: IA32W-G09RevD.01 24-Apr-2013
05-May-2018

****

%mem=1000mb
%nproc=4
Will use up to 4 processors via shared memory.

#N B3LYP/6-311+G(2d,2p) pop=nboread

1/38=1/1;
2/12=2,17=6,18=5,40=1/2;
3/5=4,6=6,7=212,11=2,16=1,25=1,30=1,74=-5/1,2,3;
4//1;
5/5=2,38=5/2;
6/7=2,8=2,9=2,10=2,28=1,40=2/1,7;
99/5=1,9=1/99;

SO2phcat NBOnaomo

Symbolic Z-matrix:
Charge = 1 Multiplicity = 1

6 1.1048 -0.5049 1.57714
6 2.22614 0.14129 1.29569
6 2.44954 0.50217 -0.14601
6 3.57739 -0.30451 -0.78079
6 2.58469 2.00163 -0.37165
16 0.25531 -0.85079 0.07067
1 0.73664 -0.91033 2.50501
1 3.64509 -0.06909 -1.84098
1 3.41596 -1.37409 -0.66342
1 1.75873 2.5523 0.07364
1 2.6214 2.21324 -1.4382
1 3.51381 2.34959 0.0784
8 1.15675 0.10031 -0.81704
8 0.2439 -2.24526 -0.27832
6 -1.35862 -0.16094 -0.01148
6 -1.55072 1.20937 0.17378
6 -2.41409 -1.04453 -0.23517

6	-2.84495	1.7015	0.13016
6	-3.70234	-0.52784	-0.27811
6	-3.91513	0.83512	-0.09476
1	-0.71759	1.87607	0.33708
1	-2.22752	-2.09786	-0.37856
1	-3.02033	2.75858	0.26612
1	-4.53604	-1.19112	-0.45589
1	2.97665	0.3874	2.03479
1	-4.92118	1.22861	-0.12875
1	4.52395	-0.04123	-0.30977

Input orientation:

Center Number	Atomic Number	Atomic Type	Coordinates (Angstroms) X	Y	Z
1	6	0	1.104800	-0.504900	1.577140
2	6	0	2.226140	0.141290	1.295690
3	6	0	2.449540	0.502170	-0.146010
4	6	0	3.577390	-0.304510	-0.780790
5	6	0	2.584690	2.001630	-0.371650
6	16	0	0.255310	-0.850790	0.070670
7	1	0	0.736640	-0.910330	2.505010
8	1	0	3.645090	-0.069090	-1.840980
9	1	0	3.415960	-1.374090	-0.663420
10	1	0	1.758730	2.552300	0.073640
11	1	0	2.621400	2.213240	-1.438200
12	1	0	3.513810	2.349590	0.078400
13	8	0	1.156750	0.100310	-0.817040
14	8	0	0.243900	-2.245260	-0.278320
15	6	0	-1.358620	-0.160940	-0.011480
16	6	0	-1.550720	1.209370	0.173780
17	6	0	-2.414090	-1.044530	-0.235170
18	6	0	-2.844950	1.701500	0.130160
19	6	0	-3.702340	-0.527840	-0.278110
20	6	0	-3.915130	0.835120	-0.094760
21	1	0	-0.717590	1.876070	0.337080
22	1	0	-2.227520	-2.097860	-0.378560
23	1	0	-3.020330	2.758580	0.266120
24	1	0	-4.536040	-1.191120	-0.455890
25	1	0	2.976650	0.387400	2.034790
26	1	0	-4.921180	1.228610	-0.128750
27	1	0	4.523950	-0.041230	-0.309770

Distance matrix (angstroms):

		1	2	3	4	5
1	C	0.000000				
2	C	1.324454	0.000000			
3	C	2.406608	1.502878	0.000000		
4	C	3.422527	2.517217	1.525032	0.000000	
5	C	3.502934	2.523776	1.522353	2.543842	0.000000
6	S	1.763725	2.523701	2.586909	3.472697	3.709172
7	H	1.077431	2.187914	3.457903	4.385585	4.491099
8	H	4.280952	3.449113	2.151418	1.088122	2.751592
9	H	3.334226	2.747755	2.173024	1.088043	3.488786

10	H	3.469093	2.743146	2.174511	3.492698	1.087994
11	H	4.333663	3.453022	2.151058	2.772214	1.087959
12	H	4.024632	2.831339	2.143826	2.790429	1.089443
13	O	2.470036	2.368312	1.510986	2.454525	2.419174
14	O	2.685653	3.478851	3.525723	3.889877	4.850163
15	C	2.951349	3.827602	3.867802	4.997664	4.511776
16	C	3.458313	4.082174	4.074859	5.431441	4.245796
17	C	3.994778	5.028065	5.104422	6.061614	5.855384
18	C	4.749998	5.432188	5.435650	6.789724	5.461033
19	C	5.152773	6.170207	6.238910	7.300482	6.777442
20	C	5.458077	6.334820	6.373579	7.609681	6.609468
21	H	3.244668	3.548794	3.485927	4.944837	3.379810
22	H	4.179311	5.258516	5.356223	6.088914	6.321648
23	H	5.420865	5.952787	5.931333	7.349044	5.691746
24	H	6.035162	7.111289	7.194552	8.168193	7.804200
25	H	2.123550	1.081714	2.246532	2.960932	2.924094
26	H	6.498273	7.368546	7.406452	8.660329	7.549480
27	H	3.932684	2.809047	2.150646	1.089565	2.817416

		6	7	8	9	10
6	S	0.000000				
7	H	2.482183	0.000000			
8	H	3.969392	5.296640	0.000000		
9	H	3.286706	4.175258	1.772618	0.000000	
10	H	3.720390	4.352704	3.754439	4.325067	0.000000
11	H	4.154919	5.371956	2.533615	3.755069	1.773364
12	H	4.567309	4.922214	3.090512	3.798113	1.766754
13	O	1.582787	3.497700	2.696106	2.702126	2.677302
14	O	1.437522	3.125981	4.329632	3.311979	5.043329
15	C	1.757103	3.359229	5.328473	4.969243	4.133617
16	C	2.741648	3.893556	5.717531	5.660661	3.572947
17	C	2.693839	4.177762	6.343798	5.855040	5.517697
18	C	4.016134	5.028852	7.010064	7.020543	4.681979
19	C	3.986093	5.253247	7.525805	7.178774	6.279672
20	C	4.501359	5.607527	7.811774	7.677816	5.930409
21	H	2.907451	3.818210	5.249816	5.352642	2.580474
22	H	2.814505	4.302493	6.382956	5.696828	6.141552
23	H	4.878070	5.708621	7.540783	7.705117	4.787381
24	H	4.832197	6.053670	8.373071	7.956812	7.342869
25	H	3.577230	2.631132	3.959392	3.252104	3.164843
26	H	5.582089	6.597172	8.831576	8.750304	6.812805
27	H	4.361354	4.798129	1.765722	1.768962	3.810491

		11	12	13	14	15
11	H	0.000000				
12	H	1.764954	0.000000			
13	O	2.644903	3.378876	0.000000		
14	O	5.184212	5.650859	2.573949	0.000000	
15	C	4.849002	5.481917	2.654103	2.642662	0.000000
16	C	4.583974	5.192173	3.089034	3.919123	1.396056
17	C	6.116903	6.838010	3.794750	2.916938	1.394556
18	C	5.709870	6.391911	4.413004	5.028396	2.387032
19	C	6.989207	7.776858	4.929074	4.303759	2.387201
20	C	6.813976	7.583716	5.175481	5.178800	2.744962
21	H	3.796598	4.265663	2.828156	4.276510	2.163752
22	H	6.574226	7.276778	4.059249	2.477840	2.154389
23	H	5.918718	6.549618	5.068297	5.999169	3.370748

24	H	7.986460	8.810345	5.848596	4.898017	3.369684
25	H	3.939740	2.822444	3.395201	4.444018	4.825191
26	H	7.718463	8.511672	6.219971	6.226411	3.825759
27	H	3.158431	2.624324	3.408136	4.814308	5.891344
		16	17	18	19	20
16	C	0.000000				
17	C	2.448002	0.000000			
18	C	1.385325	2.803531	0.000000		
19	C	2.802066	1.388669	2.423171	0.000000	
20	C	2.408861	2.409548	1.395166	1.391602	0.000000
21	H	1.079473	3.425710	2.144517	3.881491	3.390328
22	H	3.420659	1.079293	3.882673	2.156420	3.395724
23	H	2.137366	3.883615	1.080121	3.400277	2.151883
24	H	3.882135	2.138429	3.401542	1.080093	2.149789
25	H	4.963474	6.021895	6.264623	7.127137	7.227176
26	H	3.384065	3.385852	2.145085	2.143128	1.080799
27	H	6.220887	7.010604	7.584941	8.240730	8.487184
		21	22	23	24	25
21	H	0.000000				
22	H	4.310934	0.000000			
23	H	2.467077	4.962778	0.000000		
24	H	4.961554	2.481415	4.291714	0.000000	
25	H	4.329638	6.251733	6.686887	8.070671	0.000000
26	H	4.278595	4.287611	2.471834	2.471932	8.231905
27	H	5.618558	7.058102	8.067636	9.133839	2.841623
		26	27			
26	H	0.000000				
27	H	9.531828	0.000000			

Stoichiometry C11H13O2S(1+)
Framework group C1[X(C11H13O2S)]
Deg. of freedom 75
Full point group C1 NOp 1
Largest Abelian subgroup C1 NOp 1
Largest concise Abelian subgroup C1 NOp 1

Standard orientation:

Center Number	Atomic Number	Atomic Type	Coordinates (Angstroms) X	Y	Z
1	6	0	1.104800	-0.504899	1.577139
2	6	0	2.226140	0.141292	1.295689
3	6	0	2.449540	0.502172	-0.146011
4	6	0	3.577390	-0.304508	-0.780791
5	6	0	2.584689	2.001632	-0.371651
6	16	0	0.255311	-0.850790	0.070669
7	1	0	0.736641	-0.910329	2.505009
8	1	0	3.645090	-0.069087	-1.840981
9	1	0	3.415961	-1.374088	-0.663421
10	1	0	1.758728	2.552301	0.073639
11	1	0	2.621399	2.213242	-1.438201
12	1	0	3.513809	2.349592	0.078399
13	8	0	1.156750	0.100311	-0.817041
14	8	0	0.243902	-2.245260	-0.278321
15	6	0	-1.358620	-0.160941	-0.011481
16	6	0	-1.550721	1.209369	0.173779

17	6	0	-2.414089	-1.044532	-0.235171
18	6	0	-2.844951	1.701498	0.130159
19	6	0	-3.702340	-0.527842	-0.278111
20	6	0	-3.915130	0.835117	-0.094761
21	1	0	-0.717591	1.876070	0.337079
22	1	0	-2.227519	-2.097861	-0.378561
23	1	0	-3.020332	2.758578	0.266119
24	1	0	-4.536039	-1.191123	-0.455891
25	1	0	2.976650	0.387402	2.034789
26	1	0	-4.921181	1.228607	-0.128751
27	1	0	4.523950	-0.041227	-0.309771

Rotational constants (GHZ): 1.3818539 0.4215511 0.3653523
Standard basis: 6-311+G(2d,2p) (5D, 7F)
There are 531 symmetry adapted cartesian basis functions of A symmetry.
There are 503 symmetry adapted basis functions of A symmetry.
503 basis functions, 745 primitive gaussians, 531 cartesian basis functions
55 alpha electrons 55 beta electrons
nuclear repulsion energy 1046.3289590300 Hartrees.
NAtoms= 27 NActive= 27 NUniq= 27 SFac= 1.00D+00 NAtFMM= 60 NAOKFM=F Big=F
Integral buffers will be 262144 words long.
Raffenetti 2 integral format.
Two-electron integral symmetry is turned on.
One-electron integrals computed using PRISM.
NBasis= 503 RedAO= T EigKep= 1.25D-06 NBF= 503
NBsUse= 503 1.00D-06 EigRej= -1.00D+00 NBFU= 503
ExpMin= 4.05D-02 ExpMax= 9.34D+04 ExpMxC= 3.17D+03 IAcc=2 IRadAn= 4 AccDes= 0.00D+00
Harris functional with IExCor= 402 and IRadAn= 4 diagonalized for initial guess.
HarFok: IExCor= 402 AccDes= 0.00D+00 IRadAn= 4 IDoV= 1 UseB2=F ITyADJ=14
ICtDFT= 3500011 ScaDFX= 1.000000 1.000000 1.000000 1.000000
FoFCou: FMM=F IPFlag= 0 FMFlag= 100000 FMFlg1= 0
NFxFlg= 0 DoJE=T BraDBF=F KetDBF=T FulRan=T
wScrn= 0.000000 ICntrl= 500 IOpCl= 0 I1Cent= 200000004 NGrid= 0
NMat0= 1 NMatS0= 1 NMatT0= 0 NMatD0= 1 NMtDS0= 0 NMtDT0= 0
Petite list used in FoFCou.
Requested convergence on RMS density matrix=1.00D-08 within 128 cycles.
Requested convergence on MAX density matrix=1.00D-06.
Requested convergence on energy=1.00D-06.
No special actions if energy rises.
SCF Done: E(RB3LYP) = -975.521703801 A.U. after 15 cycles
NFock= 15 Conv=0.36D-08 -V/T= 2.0030

****

Population analysis using the SCF density.

****

Orbital symmetries:
Occupied (A) (A) (A) (A) (A) (A) (A) (A) (A) (A) (A) (A)
(A) (A) (A) (A) (A) (A) (A) (A) (A) (A) (A) (A)
(A) (A) (A) (A) (A) (A) (A) (A) (A) (A) (A) (A)
(A) (A) (A) (A) (A) (A) (A) (A) (A) (A) (A) (A)
(A) (A) (A) (A) (A) (A) (A)
Virtual (A) (A) (A) (A) (A) (A) (A) (A) (A) (A) (A) (A)

(A) (A) (A) (A) (A) (A) (A) (A) (A) (A) (A) (A)
(A) (A) (A) (A) (A) (A) (A) (A) (A) (A) (A) (A)
(A) (A) (A) (A) (A) (A) (A) (A) (A) (A) (A) (A)
(A) (A) (A) (A) (A) (A) (A) (A) (A) (A) (A) (A)
(A) (A) (A) (A) (A) (A) (A) (A) (A) (A) (A) (A)
(A) (A) (A) (A) (A) (A) (A) (A) (A) (A) (A) (A)
(A) (A) (A) (A) (A) (A) (A) (A) (A) (A) (A) (A)
(A) (A) (A) (A) (A) (A) (A) (A) (A) (A) (A) (A)
(A) (A) (A) (A) (A) (A) (A) (A) (A) (A) (A) (A)
(A) (A) (A) (A) (A) (A) (A) (A) (A) (A) (A) (A)
(A) (A) (A) (A) (A) (A) (A) (A) (A) (A) (A) (A)
(A) (A) (A) (A) (A) (A) (A) (A) (A) (A) (A) (A)
(A) (A) (A) (A) (A) (A) (A) (A) (A) (A) (A) (A)
(A) (A) (A) (A) (A) (A) (A) (A) (A) (A) (A) (A)
(A) (A) (A) (A) (A) (A) (A) (A) (A) (A) (A) (A)
(A) (A) (A) (A) (A) (A) (A) (A) (A) (A) (A) (A)
(A) (A) (A) (A) (A) (A) (A) (A) (A) (A) (A) (A)
(A) (A) (A) (A) (A) (A) (A) (A) (A) (A) (A) (A)
(A) (A) (A) (A) (A) (A) (A) (A) (A) (A) (A) (A)
(A) (A) (A) (A) (A) (A) (A) (A) (A) (A) (A) (A)
(A) (A) (A) (A) (A) (A) (A) (A) (A) (A) (A) (A)
(A) (A) (A) (A) (A) (A) (A) (A) (A) (A) (A) (A)
(A) (A) (A) (A) (A) (A) (A) (A) (A) (A) (A) (A)
(A) (A) (A) (A) (A) (A) (A) (A) (A) (A) (A) (A)
(A) (A) (A) (A) (A) (A) (A) (A) (A) (A) (A) (A)
(A) (A) (A) (A) (A) (A) (A) (A) (A) (A) (A) (A)
(A) (A) (A) (A) (A) (A) (A) (A) (A) (A) (A) (A)
(A) (A) (A) (A) (A) (A) (A) (A) (A) (A) (A) (A)
(A) (A) (A) (A) (A) (A) (A) (A) (A) (A) (A) (A)
(A) (A) (A) (A) (A) (A) (A) (A) (A) (A) (A) (A)
(A) (A) (A) (A) (A) (A) (A) (A) (A) (A) (A) (A)
(A) (A) (A) (A) (A) (A) (A) (A) (A) (A) (A) (A)
(A) (A) (A) (A) (A) (A) (A) (A) (A) (A) (A) (A)
(A) (A) (A) (A) (A) (A) (A) (A) (A) (A) (A) (A)
(A) (A) (A) (A) (A) (A) (A) (A) (A) (A) (A) (A)
(A) (A) (A) (A) (A) (A) (A) (A) (A) (A) (A) (A)
(A) (A) (A) (A)
The electronic state is 1-A.
Alpha occ. eigenvalues -- -89.22902 -19.35386 -19.30405 -10.42859 -10.38399
Alpha occ. eigenvalues -- -10.38242 -10.37477 -10.34630 -10.34212 -10.32967
Alpha occ. eigenvalues -- -10.32791 -10.32534 -10.31985 -10.31491 -8.29223
Alpha occ. eigenvalues --   -6.25635 -6.25338 -6.25249 -1.30679 -1.22253
Alpha occ. eigenvalues --   -1.03373 -1.01212 -0.98503 -0.92876 -0.91272
Alpha occ. eigenvalues --   -0.85957 -0.85629 -0.79732 -0.77689 -0.76875
Alpha occ. eigenvalues --   -0.75642 -0.71232 -0.67748 -0.65657 -0.64558
Alpha occ. eigenvalues --   -0.63130 -0.62001 -0.60956 -0.60418 -0.59360
Alpha occ. eigenvalues --   -0.58576 -0.57880 -0.55447 -0.55088 -0.53666
Alpha occ. eigenvalues --   -0.53002 -0.52306 -0.51892 -0.51754 -0.50749
Alpha occ. eigenvalues --   -0.49349 -0.48741 -0.47102 -0.42446 -0.41527
Alpha virt. eigenvalues --   -0.22119 -0.20198 -0.16678 -0.13364 -0.12441
Alpha virt. eigenvalues --   -0.11389 -0.10954 -0.10089 -0.09536 -0.08967
Alpha virt. eigenvalues --   -0.08464 -0.07571 -0.07338 -0.06893 -0.06404
Alpha virt. eigenvalues --   -0.05802 -0.05189 -0.05060 -0.04700 -0.04125
Alpha virt. eigenvalues --   -0.03900 -0.03789 -0.02987 -0.02508 -0.02007
Alpha virt. eigenvalues --   -0.01545 -0.00908 -0.00855 -0.00404   0.00381

Alpha virt. eigenvalues --	0.01003	0.01313	0.01564	0.01880	0.02644
Alpha virt. eigenvalues --	0.02950	0.03414	0.04235	0.04459	0.05067
Alpha virt. eigenvalues --	0.05312	0.05761	0.06088	0.06811	0.06959
Alpha virt. eigenvalues --	0.07134	0.07455	0.07699	0.08034	0.08425
Alpha virt. eigenvalues --	0.08746	0.09105	0.09513	0.10350	0.10516
Alpha virt. eigenvalues --	0.10835	0.11060	0.11165	0.11581	0.12232
Alpha virt. eigenvalues --	0.12861	0.12954	0.13290	0.14444	0.14537
Alpha virt. eigenvalues --	0.14967	0.15512	0.15596	0.16455	0.16542
Alpha virt. eigenvalues --	0.17269	0.18322	0.18873	0.19387	0.19542
Alpha virt. eigenvalues --	0.19856	0.20795	0.21010	0.21860	0.23203
Alpha virt. eigenvalues --	0.23488	0.24889	0.25295	0.25597	0.25971
Alpha virt. eigenvalues --	0.26254	0.28149	0.28706	0.29720	0.30684
Alpha virt. eigenvalues --	0.30885	0.32088	0.32917	0.33592	0.34214
Alpha virt. eigenvalues --	0.35368	0.36109	0.36479	0.37151	0.37758
Alpha virt. eigenvalues --	0.38466	0.38719	0.39234	0.39956	0.40302
Alpha virt. eigenvalues --	0.41641	0.41939	0.42194	0.42754	0.43951
Alpha virt. eigenvalues --	0.44426	0.44972	0.45088	0.46101	0.46670
Alpha virt. eigenvalues --	0.47087	0.47633	0.48808	0.49097	0.49214
Alpha virt. eigenvalues --	0.49809	0.50631	0.50852	0.51759	0.51945
Alpha virt. eigenvalues --	0.53478	0.53920	0.54324	0.54700	0.54844
Alpha virt. eigenvalues --	0.55630	0.55810	0.56680	0.56787	0.57520
Alpha virt. eigenvalues --	0.58129	0.58478	0.59081	0.60200	0.60703
Alpha virt. eigenvalues --	0.61346	0.61882	0.62777	0.63829	0.64620
Alpha virt. eigenvalues --	0.65092	0.65813	0.66483	0.67189	0.67821
Alpha virt. eigenvalues --	0.68834	0.70131	0.70452	0.72200	0.72738
Alpha virt. eigenvalues --	0.74640	0.75601	0.76062	0.76830	0.78634
Alpha virt. eigenvalues --	0.79384	0.80040	0.80937	0.83009	0.84435
Alpha virt. eigenvalues --	0.85382	0.86560	0.86830	0.88278	0.89076
Alpha virt. eigenvalues --	0.89928	0.90555	0.91211	0.91662	0.92619
Alpha virt. eigenvalues --	0.94360	0.95062	0.95571	0.96301	0.96650
Alpha virt. eigenvalues --	0.98081	0.99330	1.00673	1.02466	1.03027
Alpha virt. eigenvalues --	1.03827	1.04758	1.04968	1.05494	1.06044
Alpha virt. eigenvalues --	1.07219	1.08262	1.08566	1.09833	1.11307
Alpha virt. eigenvalues --	1.11714	1.12443	1.13216	1.13840	1.14055
Alpha virt. eigenvalues --	1.14482	1.15627	1.16114	1.18112	1.18293
Alpha virt. eigenvalues --	1.19398	1.19835	1.20428	1.21708	1.21950
Alpha virt. eigenvalues --	1.22415	1.23118	1.23529	1.24146	1.24843
Alpha virt. eigenvalues --	1.25405	1.26449	1.26899	1.28042	1.28794
Alpha virt. eigenvalues --	1.28985	1.29338	1.30945	1.32177	1.33574
Alpha virt. eigenvalues --	1.34444	1.35104	1.36153	1.36993	1.37060
Alpha virt. eigenvalues --	1.39426	1.40966	1.42744	1.43602	1.45126
Alpha virt. eigenvalues --	1.47378	1.48030	1.48169	1.48755	1.49928
Alpha virt. eigenvalues --	1.51885	1.53769	1.54421	1.55513	1.56784
Alpha virt. eigenvalues --	1.57776	1.60371	1.61754	1.64660	1.66158
Alpha virt. eigenvalues --	1.67719	1.69529	1.70784	1.73355	1.76870
Alpha virt. eigenvalues --	1.77632	1.82442	1.84661	1.86756	1.88690
Alpha virt. eigenvalues --	1.89994	1.91289	1.94469	1.98808	2.06424
Alpha virt. eigenvalues --	2.08762	2.14146	2.17929	2.25857	2.35889
Alpha virt. eigenvalues --	2.40789	2.43898	2.50224	2.53913	2.56580
Alpha virt. eigenvalues --	2.58166	2.60705	2.61585	2.62974	2.64466
Alpha virt. eigenvalues --	2.65767	2.66194	2.69523	2.72074	2.72755
Alpha virt. eigenvalues --	2.73651	2.74813	2.76367	2.78169	2.79911
Alpha virt. eigenvalues --	2.85479	2.86603	2.87341	2.89834	2.91892
Alpha virt. eigenvalues --	2.94897	2.96321	2.97117	2.99985	3.00918
Alpha virt. eigenvalues --	3.01524	3.04500	3.05182	3.06308	3.07296

Alpha virt. eigenvalues -- 3.10446 3.11129 3.13966 3.14748 3.16205
Alpha virt. eigenvalues -- 3.16719 3.17568 3.17880 3.18837 3.19842
Alpha virt. eigenvalues -- 3.20498 3.21669 3.22790 3.23975 3.25300
Alpha virt. eigenvalues -- 3.25960 3.26759 3.28307 3.28845 3.32052
Alpha virt. eigenvalues -- 3.32895 3.34160 3.36212 3.37639 3.39100
Alpha virt. eigenvalues -- 3.39634 3.40433 3.40835 3.41117 3.41743
Alpha virt. eigenvalues -- 3.43020 3.43534 3.44107 3.44689 3.45392
Alpha virt. eigenvalues -- 3.47721 3.49460 3.50315 3.50606 3.53759
Alpha virt. eigenvalues -- 3.54909 3.55474 3.56414 3.57316 3.57636
Alpha virt. eigenvalues -- 3.59553 3.60591 3.61854 3.62228 3.63950
Alpha virt. eigenvalues -- 3.65379 3.68394 3.71469 3.71679 3.73284
Alpha virt. eigenvalues -- 3.74604 3.77141 3.78611 3.78916 3.81277
Alpha virt. eigenvalues -- 3.82040 3.82180 3.82517 3.84986 3.85843
Alpha virt. eigenvalues -- 3.86692 3.87500 3.88267 3.89822 3.91007
Alpha virt. eigenvalues -- 3.91763 3.93811 3.94791 3.95844 3.98004
Alpha virt. eigenvalues -- 3.98705 4.00379 4.02152 4.03265 4.06190
Alpha virt. eigenvalues -- 4.08161 4.08949 4.11563 4.12244 4.13066
Alpha virt. eigenvalues -- 4.14900 4.16268 4.16905 4.23130 4.24465
Alpha virt. eigenvalues -- 4.29155 4.30862 4.37552 4.52635 4.54231
Alpha virt. eigenvalues -- 4.56704 4.57857 4.65994 4.69085 4.88236
Alpha virt. eigenvalues -- 4.88370 4.89769 4.90226 4.91705 4.92695
Alpha virt. eigenvalues -- 4.94259 4.99214 5.01106 5.02673 5.03918
Alpha virt. eigenvalues -- 5.05808 5.10662 5.14858 5.24834 5.39413
Alpha virt. eigenvalues -- 6.61844 6.64280 6.69024 6.80738 6.86111
Alpha virt. eigenvalues -- 6.93999 6.97081 7.05952 7.22193 7.31591
Alpha virt. eigenvalues -- 8.32389 17.34573 17.49714 17.65704 23.47660
Alpha virt. eigenvalues -- 23.57821 23.65852 23.84761 23.86597 23.87024
Alpha virt. eigenvalues -- 23.87361 23.95794 23.96415 23.99649 24.07229
Alpha virt. eigenvalues -- 49.80579 49.85943 189.50299

Condensed to atoms (all electrons):

		1	2	3	4	5	6
1	C	5.973919	-0.055291	0.446768	0.088892	0.032282	-1.343400
2	C	-0.055291	5.931821	-0.409245	-0.014890	-0.000772	0.220013
3	C	0.446768	-0.409245	6.605780	-0.344201	0.422624	-1.604540
4	C	0.088892	-0.014890	-0.344201	5.519829	-0.176199	0.225835
5	C	0.032282	-0.000772	0.422624	-0.176199	5.019128	-0.194940
6	S	-1.343400	0.220013	-1.604540	0.225835	-0.194940	20.223931
7	H	0.381671	0.018600	-0.021450	0.005520	-0.001444	-0.011268
8	H	-0.001490	0.011866	0.027536	0.377473	-0.012721	-0.015012
9	H	-0.012873	0.001171	-0.008847	0.401029	0.009875	0.002052
10	H	-0.007471	0.005999	-0.003561	0.001997	0.382484	0.015309
11	H	-0.001342	0.006828	0.011427	-0.007717	0.392834	-0.007673
12	H	0.002042	-0.008442	-0.042322	0.001986	0.421154	0.010414
13	O	-0.133573	0.062920	0.091958	-0.057257	-0.054856	0.416954
14	O	0.000906	-0.016209	0.049083	-0.030397	0.011035	0.185113
15	C	-0.302885	0.139023	0.085116	-0.002550	-0.086293	-1.199733
16	C	1.365188	-0.247572	0.331750	-0.064629	0.149192	-2.542630
17	C	-1.045827	0.280315	-0.166564	-0.007823	-0.051414	2.424065
18	C	0.300499	0.003711	0.065461	-0.027931	0.068902	-0.969448
19	C	0.036669	-0.030672	0.009082	0.013163	-0.003738	0.072412
20	C	0.029467	0.013177	-0.005020	-0.005412	0.009029	-0.150002
21	H	0.001408	0.003791	-0.009902	0.002188	-0.005402	0.042810
22	H	-0.001722	0.000795	-0.001222	0.000006	0.000130	-0.003467
23	H	-0.000045	-0.000080	-0.000333	-0.000003	0.000417	0.002047
24	H	-0.000437	0.000042	-0.000075	0.000005	-0.000003	0.001945

25	H	0.005326	0.405612	-0.079996	0.012332	0.005290	0.009477
26	H	0.000206	-0.000077	0.000042	-0.000003	0.000011	-0.000285
27	H	0.001399	-0.001017	-0.005461	0.376147	0.001559	0.003575
		7	8	9	10	11	12
1	C	0.381671	-0.001490	-0.012873	-0.007471	-0.001342	0.002042
2	C	0.018600	0.011866	0.001171	0.005999	0.006828	-0.008442
3	C	-0.021450	0.027536	-0.008847	-0.003561	0.011427	-0.042322
4	C	0.005520	0.377473	0.401029	0.001997	-0.007717	0.001986
5	C	-0.001444	-0.012721	0.009875	0.382484	0.392834	0.421154
6	S	-0.011268	-0.015012	0.002052	0.015309	-0.007673	0.010414
7	H	0.507219	0.000018	0.000029	0.000031	0.000019	-0.000024
8	H	0.000018	0.518093	-0.018524	-0.000048	0.001692	-0.000001
9	H	0.000029	-0.018524	0.513707	-0.000281	-0.000094	0.000060
10	H	0.000031	-0.000048	-0.000281	0.541199	-0.019890	-0.024945
11	H	0.000019	0.001692	-0.000094	-0.019890	0.509359	-0.020154
12	H	-0.000024	-0.000001	0.000060	-0.024945	-0.020154	0.514598
13	O	0.001201	-0.006549	-0.007206	-0.006799	-0.009055	0.008783
14	O	0.004398	0.000555	0.000722	0.000037	-0.000026	0.000062
15	C	0.034915	0.000402	0.009840	-0.007446	0.005908	-0.003188
16	C	-0.021981	0.002666	-0.000732	-0.005656	-0.002819	0.001229
17	C	0.000569	-0.000605	0.000438	-0.000377	0.000980	-0.000142
18	C	-0.004269	0.000189	-0.000395	0.007306	-0.003013	0.000033
19	C	0.000560	-0.000054	0.000030	-0.000081	-0.000172	0.000037
20	C	-0.001251	0.000020	0.000046	-0.001040	0.000067	-0.000012
21	H	0.000101	0.000007	0.000009	0.001884	-0.000170	0.000064
22	H	-0.000003	0.000000	-0.000003	0.000000	0.000000	0.000000
23	H	0.000000	0.000000	0.000000	-0.000021	0.000000	0.000000
24	H	0.000000	0.000000	0.000000	0.000000	0.000000	0.000000
25	H	-0.004527	-0.000120	0.000048	-0.000534	-0.000238	0.001741
26	H	0.000000	0.000000	0.000000	0.000000	0.000000	0.000000
27	H	-0.000017	-0.021657	-0.021983	0.000084	0.000262	0.000548
		13	14	15	16	17	18
1	C	-0.133573	0.000906	-0.302885	1.365188	-1.045827	0.300499
2	C	0.062920	-0.016209	0.139023	-0.247572	0.280315	0.003711
3	C	0.091958	0.049083	0.085116	0.331750	-0.166564	0.065461
4	C	-0.057257	-0.030397	-0.002550	-0.064629	-0.007823	-0.027931
5	C	-0.054856	0.011035	-0.086293	0.149192	-0.051414	0.068902
6	S	0.416954	0.185113	-1.199733	-2.542630	2.424065	-0.969448
7	H	0.001201	0.004398	0.034915	-0.021981	0.000569	-0.004269
8	H	-0.006549	0.000555	0.000402	0.002666	-0.000605	0.000189
9	H	-0.007206	0.000722	0.009840	-0.000732	0.000438	-0.000395
10	H	-0.006799	0.000037	-0.007446	-0.005656	-0.000377	0.007306
11	H	-0.009055	-0.000026	0.005908	-0.002819	0.000980	-0.003013
12	H	0.008783	0.000062	-0.003188	0.001229	-0.000142	0.000033
13	O	8.089875	-0.010912	0.023155	-0.188255	0.088532	-0.001516
14	O	-0.010912	8.069933	0.187071	0.358506	-0.563895	0.023375
15	C	0.023155	0.187071	11.452985	-2.210110	-0.821998	-0.605277
16	C	-0.188255	0.358506	-2.210110	14.322526	-5.820195	-0.065845
17	C	0.088532	-0.563895	-0.821998	-5.820195	13.479421	-1.295528
18	C	-0.001516	0.023375	-0.605277	-0.065845	-1.295528	7.495139
19	C	-0.002900	0.030480	-0.281629	-0.495347	-0.566930	0.477853
20	C	0.005677	-0.005644	-0.863360	0.422762	0.175018	0.409930
21	H	0.003106	0.002504	-0.159921	0.550423	-0.081506	0.015700
22	H	0.000591	0.010337	-0.041985	0.017865	0.383719	-0.003650
23	H	-0.000045	0.000013	0.007597	-0.013121	0.004008	0.365328

24	H	-0.000031	0.000122	0.012147	-0.003919	0.015617	0.004607
25	H	-0.001399	0.001216	-0.009827	0.018374	-0.007028	0.001401
26	H	0.000014	-0.000008	0.001073	0.017686	-0.007142	-0.020157
27	H	0.008408	0.000225	-0.002225	0.002250	-0.000325	0.000265

		19	20	21	22	23	24
1	C	0.036669	0.029467	0.001408	-0.001722	-0.000045	-0.000437
2	C	-0.030672	0.013177	0.003791	0.000795	-0.000080	0.000042
3	C	0.009082	-0.005020	-0.009902	-0.001222	-0.000333	-0.000075
4	C	0.013163	-0.005412	0.002188	0.000006	-0.000003	0.000005
5	C	-0.003738	0.009029	-0.005402	0.000130	0.000417	-0.000003
6	S	0.072412	-0.150002	0.042810	-0.003467	0.002047	0.001945
7	H	0.000560	-0.001251	0.000101	-0.000003	0.000000	0.000000
8	H	-0.000054	0.000020	0.000007	0.000000	0.000000	0.000000
9	H	0.000030	0.000046	0.000009	-0.000003	0.000000	0.000000
10	H	-0.000081	-0.001040	0.001884	0.000000	-0.000021	0.000000
11	H	-0.000172	0.000067	-0.000170	0.000000	0.000000	0.000000
12	H	0.000037	-0.000012	0.000064	0.000000	0.000000	0.000000
13	O	-0.002900	0.005677	0.003106	0.000591	-0.000045	-0.000031
14	O	0.030480	-0.005644	0.002504	0.010337	0.000013	0.000122
15	C	-0.281629	-0.863360	-0.159921	-0.041985	0.007597	0.012147
16	C	-0.495347	0.422762	0.550423	0.017865	-0.013121	-0.003919
17	C	-0.566930	0.175018	-0.081506	0.383719	0.004008	0.015617
18	C	0.477853	0.409930	0.015700	-0.003650	0.365328	0.004607
19	C	6.216317	0.358022	-0.000783	0.007085	0.004544	0.354968
20	C	0.358022	5.266409	0.006919	0.011339	0.001738	-0.011863
21	H	-0.000783	0.006919	0.495247	-0.000110	-0.004128	0.000066
22	H	0.007085	0.011339	-0.000110	0.465476	0.000058	-0.004297
23	H	0.004544	0.001738	-0.004128	0.000058	0.507908	-0.000151
24	H	0.354968	-0.011863	0.000066	-0.004297	-0.000151	0.503076
25	H	-0.000097	0.000088	0.000095	0.000001	0.000000	0.000000
26	H	-0.002771	0.386412	-0.000157	-0.000139	-0.004877	-0.005012
27	H	-0.000019	0.000004	0.000001	0.000000	0.000000	0.000000

		25	26	27
1	C	0.005326	0.000206	0.001399
2	C	0.405612	-0.000077	-0.001017
3	C	-0.079996	0.000042	-0.005461
4	C	0.012332	-0.000003	0.376147
5	C	0.005290	0.000011	0.001559
6	S	0.009477	-0.000285	0.003575
7	H	-0.004527	0.000000	-0.000017
8	H	-0.000120	0.000000	-0.021657
9	H	0.000048	0.000000	-0.021983
10	H	-0.000534	0.000000	0.000084
11	H	-0.000238	0.000000	0.000262
12	H	0.001741	0.000000	0.000548
13	O	-0.001399	0.000014	0.008408
14	O	0.001216	-0.000008	0.000225
15	C	-0.009827	0.001073	-0.002225
16	C	0.018374	0.017686	0.002250
17	C	-0.007028	-0.007142	-0.000325
18	C	0.001401	-0.020157	0.000265
19	C	-0.000097	-0.002771	-0.000019
20	C	0.000088	0.386412	0.000004
21	H	0.000095	-0.000157	0.000001
22	H	0.000001	-0.000139	0.000000

23	H	0.000000	-0.004877	0.000000
24	H	0.000000	-0.005012	0.000000
25	H	0.506535	0.000000	0.001261
26	H	0.000000	0.499595	0.000000
27	H	0.001261	0.000000	0.526251

Mulliken charges:

		1
1	C	0.239713
2	C	-0.321415
3	C	0.556114
4	C	-0.287388
5	C	-0.338163
6	S	0.186447
7	H	0.111383
8	H	0.136261
9	H	0.131884
10	H	0.121819
11	H	0.142989
12	H	0.136479
13	O	-0.320821
14	O	-0.308605
15	C	0.639196
16	C	0.122396
17	C	-0.415386
18	C	-0.242672
19	C	-0.196031
20	C	-0.052517
21	H	0.135756
22	H	0.159198
23	H	0.129148
24	H	0.133193
25	H	0.134968
26	H	0.135588
27	H	0.130466

Sum of Mulliken charges = 1.00000

Mulliken charges with hydrogens summed into heavy atoms:

		1
1	C	0.351096
2	C	-0.186448
3	C	0.556114
4	C	0.111223
5	C	0.063124
6	S	0.186447
13	O	-0.320821
14	O	-0.308605
15	C	0.639196
16	C	0.258152
17	C	-0.256188
18	C	-0.113524
19	C	-0.062838
20	C	0.083071

Electronic spatial extent (au): <R**2>= 3121.2734

Charge= 1.0000 electrons

Dipole moment (field-independent basis, Debye):

X= 1.3666 Y= 2.6968 Z= 2.4357 Tot= 3.8824

Quadrupole moment (field-independent basis, Debye-Ang):
XX= -38.2353 YY= -79.6796 ZZ= -82.0947
XY= 2.9900 XZ= 3.6455 YZ= -1.1410
Traceless Quadrupole moment (field-independent basis, Debye-Ang):
XX= 28.4345 YY= -13.0097 ZZ= -15.4249
XY= 2.9900 XZ= 3.6455 YZ= -1.1410
Octapole moment (field-independent basis, Debye-Ang**2):
XXX= -14.8950 YYY= 18.2726 ZZZ= 10.3014 XYY= -9.5259
XXY= 18.6570 XXZ= -7.5279 XZZ= 26.4821 YZZ= -7.6720
YYZ= 0.0481 XYZ= -4.1265
Hexadecapole moment (field-independent basis, Debye-Ang**3):
XXXX= -2276.6222 YYYY= -763.7553 ZZZZ= -293.6454 XXXY= 3.0633
XXXZ= 34.4833 YYYX= 4.1095 YYYZ= 2.9799 ZZZX= 7.9472
ZZZY= -15.3255 XXYY= -539.9989 XXZZ= -550.5848 YYZZ= -188.0384
XXYZ= 18.4043 YYXZ= -5.4689 ZZXY= 9.9592
N-N= 1.046328959030D+03 E-N=-4.365100401454D+03 KE= 9.725860567104D+02
*****************************Gaussian NBO Version 3.1******************************
N A T U R A L A T O M I C O R B I T A L A N D
N A T U R A L B O N D O R B I T A L A N A L Y S I S
*****************************Gaussian NBO Version 3.1******************************

/RESON / : Allow strongly delocalized NBO set
/NAOMO / : Print all MOs in the NAO basis

Analyzing the SCF density

Job title: SO2phcat NBOnaomo

Storage needed: 1268289 in NPA, 1014309 in NBO (131069797 available)

NATURAL POPULATIONS: Natural atomic orbital occupancies

NAO	Atom	No	lang	Type(AO)	Occupancy	Energy
1	C	1	S	Cor(1S)	1.99885	-10.25170
2	C	1	S	Val(2S)	1.06045	-0.45363
3	C	1	S	Ryd(4S)	0.00316	0.98688
4	C	1	S	Ryd(3S)	0.00025	0.51638
5	C	1	S	Ryd(5S)	0.00001	22.31859
6	C	1	px	Val(2p)	1.10319	-0.31311
7	C	1	px	Ryd(4p)	0.00247	0.66082
8	C	1	px	Ryd(3p)	0.00024	0.46897
9	C	1	px	Ryd(5p)	0.00016	3.15478
10	C	1	py	Val(2p)	1.12583	-0.32027
11	C	1	py	Ryd(4p)	0.00275	0.54620
12	C	1	py	Ryd(3p)	0.00025	0.31829
13	C	1	py	Ryd(5p)	0.00008	2.86799
14	C	1	pz	Val(2p)	1.11527	-0.30450
15	C	1	pz	Ryd(4p)	0.00539	0.85822
16	C	1	pz	Ryd(3p)	0.00030	0.37924
17	C	1	pz	Ryd(5p)	0.00007	3.24094
18	C	1	dxy	Ryd(3d)	0.00171	1.31832
19	C	1	dxy	Ryd(4d)	0.00009	3.36333
20	C	1	dxz	Ryd(3d)	0.00242	1.39241

21	C	1	dxz	Ryd(4d)	0.00038	3.48360
22	C	1	dyz	Ryd(3d)	0.00198	1.13668
23	C	1	dyz	Ryd(4d)	0.00020	3.36274
24	C	1	dx2y2	Ryd(3d)	0.00134	1.03280
25	C	1	dx2y2	Ryd(4d)	0.00011	3.26673
26	C	1	dz2	Ryd(3d)	0.00313	1.40225
27	C	1	dz2	Ryd(4d)	0.00022	3.55946
28	C	2	S	Cor(1S)	1.99893	-10.22904
29	C	2	S	Val(2S)	0.97359	-0.38340
30	C	2	S	Ryd(3S)	0.00241	0.90795
31	C	2	S	Ryd(4S)	0.00010	1.88347
32	C	2	S	Ryd(5S)	0.00001	20.64035
33	C	2	px	Val(2p)	1.09362	-0.27126
34	C	2	px	Ryd(4p)	0.00323	0.76630
35	C	2	px	Ryd(3p)	0.00030	0.54792
36	C	2	px	Ryd(5p)	0.00011	3.14443
37	C	2	py	Val(2p)	0.91472	-0.29228
38	C	2	py	Ryd(4p)	0.00335	0.52478
39	C	2	py	Ryd(3p)	0.00029	0.40728
40	C	2	py	Ryd(5p)	0.00008	2.79081
41	C	2	pz	Val(2p)	1.10142	-0.25667
42	C	2	pz	Ryd(4p)	0.00513	0.69112
43	C	2	pz	Ryd(3p)	0.00031	0.53195
44	C	2	pz	Ryd(5p)	0.00007	3.19923
45	C	2	dxy	Ryd(3d)	0.00154	1.54675
46	C	2	dxy	Ryd(4d)	0.00007	3.31278
47	C	2	dxz	Ryd(3d)	0.00067	1.51155
48	C	2	dxz	Ryd(4d)	0.00046	3.57198
49	C	2	dyz	Ryd(3d)	0.00061	1.28910
50	C	2	dyz	Ryd(4d)	0.00031	3.30374
51	C	2	dx2y2	Ryd(3d)	0.00118	1.31454
52	C	2	dx2y2	Ryd(4d)	0.00011	3.24379
53	C	2	dz2	Ryd(3d)	0.00172	1.45877
54	C	2	dz2	Ryd(4d)	0.00024	3.46458
55	C	3	S	Cor(1S)	1.99907	-10.30100
56	C	3	S	Val(2S)	0.93540	-0.41202
57	C	3	S	Ryd(3S)	0.00101	0.94069
58	C	3	S	Ryd(4S)	0.00013	1.96423
59	C	3	S	Ryd(5S)	0.00000	22.29485
60	C	3	px	Val(2p)	0.77463	-0.26941
61	C	3	px	Ryd(3p)	0.00513	0.73368
62	C	3	px	Ryd(4p)	0.00034	1.02221
63	C	3	px	Ryd(5p)	0.00003	2.97636
64	C	3	py	Val(2p)	1.05667	-0.29121
65	C	3	py	Ryd(3p)	0.00325	0.75637
66	C	3	py	Ryd(4p)	0.00028	1.01162
67	C	3	py	Ryd(5p)	0.00005	2.83851
68	C	3	pz	Val(2p)	0.95299	-0.27817
69	C	3	pz	Ryd(3p)	0.00490	0.67255
70	C	3	pz	Ryd(4p)	0.00029	0.95988
71	C	3	pz	Ryd(5p)	0.00003	2.78086
72	C	3	dxy	Ryd(3d)	0.00125	1.96609
73	C	3	dxy	Ryd(4d)	0.00032	2.98364

74	C	3	dxz	Ryd(3d)	0.00067	2.06167
75	C	3	dxz	Ryd(4d)	0.00053	3.01049
76	C	3	dyz	Ryd(3d)	0.00052	1.87231
77	C	3	dyz	Ryd(4d)	0.00033	2.89581
78	C	3	dx2y2	Ryd(3d)	0.00148	2.03683
79	C	3	dx2y2	Ryd(4d)	0.00016	3.06424
80	C	3	dz2	Ryd(3d)	0.00144	1.97982
81	C	3	dz2	Ryd(4d)	0.00018	3.08011
82	C	4	S	Cor(1S)	1.99919	-10.16719
83	C	4	S	Val(2S)	1.08672	-0.40268
84	C	4	S	Ryd(4S)	0.00072	0.94780
85	C	4	S	Ryd(3S)	0.00007	0.55720
86	C	4	S	Ryd(5S)	0.00000	22.79053
87	C	4	px	Val(2p)	1.11106	-0.24565
88	C	4	px	Ryd(4p)	0.00203	0.54813
89	C	4	px	Ryd(3p)	0.00015	0.19853
90	C	4	px	Ryd(5p)	0.00004	3.18377
91	C	4	py	Val(2p)	1.17705	-0.24458
92	C	4	py	Ryd(4p)	0.00330	0.57487
93	C	4	py	Ryd(3p)	0.00015	0.23161
94	C	4	py	Ryd(5p)	0.00002	3.32882
95	C	4	pz	Val(2p)	1.19870	-0.24837
96	C	4	pz	Ryd(4p)	0.00219	0.55822
97	C	4	pz	Ryd(3p)	0.00011	0.19606
98	C	4	pz	Ryd(5p)	0.00001	3.32394
99	C	4	dxy	Ryd(3d)	0.00172	1.32469
100	C	4	dxy	Ryd(4d)	0.00009	3.35291
101	C	4	dxz	Ryd(3d)	0.00168	1.31096
102	C	4	dxz	Ryd(4d)	0.00008	3.38774
103	C	4	dyz	Ryd(3d)	0.00134	1.24620
104	C	4	dyz	Ryd(4d)	0.00004	3.33778
105	C	4	dx2y2	Ryd(3d)	0.00143	1.37420
106	C	4	dx2y2	Ryd(4d)	0.00015	3.52053
107	C	4	dz2	Ryd(3d)	0.00199	1.31074
108	C	4	dz2	Ryd(4d)	0.00006	3.49066
109	C	5	S	Cor(1S)	1.99919	-10.17190
110	C	5	S	Val(2S)	1.08382	-0.40601
111	C	5	S	Ryd(4S)	0.00068	0.94832
112	C	5	S	Ryd(3S)	0.00008	0.52357
113	C	5	S	Ryd(5S)	0.00000	22.77608
114	C	5	px	Val(2p)	1.21921	-0.25040
115	C	5	px	Ryd(4p)	0.00297	0.57661
116	C	5	px	Ryd(3p)	0.00009	0.27269
117	C	5	px	Ryd(5p)	0.00001	3.34507
118	C	5	py	Val(2p)	1.03348	-0.24842
119	C	5	py	Ryd(4p)	0.00215	0.51454
120	C	5	py	Ryd(3p)	0.00025	0.14736
121	C	5	py	Ryd(5p)	0.00004	3.18285
122	C	5	pz	Val(2p)	1.23206	-0.25289
123	C	5	pz	Ryd(4p)	0.00267	0.56681
124	C	5	pz	Ryd(3p)	0.00008	0.20278
125	C	5	pz	Ryd(5p)	0.00001	3.34929
126	C	5	dxy	Ryd(3d)	0.00069	1.34002

127	C	5	dxy	Ryd(4d)	0.00018	3.44221
128	C	5	dxz	Ryd(3d)	0.00208	1.24975
129	C	5	dxz	Ryd(4d)	0.00002	3.40074
130	C	5	dyz	Ryd(3d)	0.00042	1.29711
131	C	5	dyz	Ryd(4d)	0.00014	3.37035
132	C	5	dx2y2	Ryd(3d)	0.00236	1.37635
133	C	5	dx2y2	Ryd(4d)	0.00005	3.35836
134	C	5	dz2	Ryd(3d)	0.00246	1.31402
135	C	5	dz2	Ryd(4d)	0.00002	3.44749

136	S	6	S	Cor(1S)	2.00000	-88.06389
137	S	6	S	Cor(2S)	1.99850	-9.21313
138	S	6	S	Val(3S)	1.12146	-0.75201
139	S	6	S	Ryd(4S)	0.00415	0.86470
140	S	6	S	Ryd(5S)	0.00019	3.95397
141	S	6	S	Ryd(6S)	0.00004	15.25298
142	S	6	S	Ryd(7S)	0.00000	178.77098
143	S	6	px	Cor(2p)	1.99982	-6.24099
144	S	6	px	Val(3p)	0.96388	-0.38020
145	S	6	px	Ryd(6p)	0.00447	1.46846
146	S	6	px	Ryd(5p)	0.00106	0.75773
147	S	6	px	Ryd(4p)	0.00030	0.71789
148	S	6	px	Ryd(7p)	0.00001	15.93398
149	S	6	py	Cor(2p)	1.99980	-6.23907
150	S	6	py	Val(3p)	0.74104	-0.33967
151	S	6	py	Ryd(6p)	0.01171	1.23014
152	S	6	py	Ryd(5p)	0.00115	0.87074
153	S	6	py	Ryd(4p)	0.00034	0.78372
154	S	6	py	Ryd(7p)	0.00003	16.39918
155	S	6	pz	Cor(2p)	1.99984	-6.24292
156	S	6	pz	Val(3p)	0.92771	-0.39512
157	S	6	pz	Ryd(6p)	0.00530	1.10546
158	S	6	pz	Ryd(4p)	0.00068	0.67865
159	S	6	pz	Ryd(5p)	0.00052	0.74462
160	S	6	pz	Ryd(7p)	0.00001	16.30445
161	S	6	dxy	Ryd(3d)	0.04269	0.77230
162	S	6	dxy	Ryd(4d)	0.00009	2.95421
163	S	6	dxz	Ryd(3d)	0.02222	0.66308
164	S	6	dxz	Ryd(4d)	0.00008	2.44546
165	S	6	dyz	Ryd(3d)	0.03317	0.69703
166	S	6	dyz	Ryd(4d)	0.00017	2.48769
167	S	6	dx2y2	Ryd(3d)	0.02756	0.82652
168	S	6	dx2y2	Ryd(4d)	0.00031	2.52518
169	S	6	dz2	Ryd(3d)	0.02260	0.68842
170	S	6	dz2	Ryd(4d)	0.00014	2.56546

171	H	7	S	Val(1S)	0.72618	-0.11310
172	H	7	S	Ryd(3S)	0.00067	1.52051
173	H	7	S	Ryd(2S)	0.00019	1.10844
174	H	7	px	Ryd(2p)	0.00015	1.16894
175	H	7	px	Ryd(3p)	0.00013	3.86636
176	H	7	py	Ryd(2p)	0.00013	1.03550
177	H	7	py	Ryd(3p)	0.00013	3.72953
178	H	7	pz	Ryd(2p)	0.00060	1.64096
179	H	7	pz	Ryd(3p)	0.00030	3.89262

180	H	8	S	Val(1S)	0.76214	-0.09048
181	H	8	S	Ryd(3S)	0.00058	1.55842
182	H	8	S	Ryd(2S)	0.00008	1.17676
183	H	8	px	Ryd(2p)	0.00021	1.91993
184	H	8	px	Ryd(3p)	0.00006	2.92373
185	H	8	py	Ryd(2p)	0.00020	1.91494
186	H	8	py	Ryd(3p)	0.00005	2.91857
187	H	8	pz	Ryd(2p)	0.00029	2.65854
188	H	8	pz	Ryd(3p)	0.00022	3.09501
189	H	9	S	Val(1S)	0.76969	-0.09367
190	H	9	S	Ryd(3S)	0.00087	1.36858
191	H	9	S	Ryd(2S)	0.00010	1.35046
192	H	9	px	Ryd(2p)	0.00034	1.83367
193	H	9	px	Ryd(3p)	0.00006	3.02370
194	H	9	py	Ryd(2p)	0.00028	2.62321
195	H	9	py	Ryd(3p)	0.00022	3.16847
196	H	9	pz	Ryd(2p)	0.00012	1.80033
197	H	9	pz	Ryd(3p)	0.00005	2.98720
198	H	10	S	Val(1S)	0.78231	-0.10734
199	H	10	S	Ryd(3S)	0.00072	1.40340
200	H	10	S	Ryd(2S)	0.00008	1.38954
201	H	10	px	Ryd(2p)	0.00037	2.15019
202	H	10	px	Ryd(3p)	0.00014	3.19883
203	H	10	py	Ryd(2p)	0.00020	1.92286
204	H	10	py	Ryd(3p)	0.00012	3.18948
205	H	10	pz	Ryd(2p)	0.00017	1.80960
206	H	10	pz	Ryd(3p)	0.00008	3.11815
207	H	11	S	Val(1S)	0.76115	-0.09419
208	H	11	S	Ryd(3S)	0.00057	1.54719
209	H	11	S	Ryd(2S)	0.00007	1.20961
210	H	11	px	Ryd(2p)	0.00011	1.98772
211	H	11	px	Ryd(3p)	0.00005	2.81553
212	H	11	py	Ryd(2p)	0.00022	2.04012
213	H	11	py	Ryd(3p)	0.00007	2.85551
214	H	11	pz	Ryd(2p)	0.00033	2.70795
215	H	11	pz	Ryd(3p)	0.00021	3.02606
216	H	12	S	Val(1S)	0.76456	-0.10482
217	H	12	S	Ryd(3S)	0.00031	1.46721
218	H	12	S	Ryd(2S)	0.00006	1.24070
219	H	12	px	Ryd(2p)	0.00029	2.74197
220	H	12	px	Ryd(3p)	0.00014	2.77165
221	H	12	py	Ryd(2p)	0.00015	2.31538
222	H	12	py	Ryd(3p)	0.00012	2.63251
223	H	12	pz	Ryd(2p)	0.00015	2.34284
224	H	12	pz	Ryd(3p)	0.00007	2.63253
225	O	13	S	Cor(1S)	1.99977	-19.15698
226	O	13	S	Val(2S)	1.71255	-1.13391
227	O	13	S	Ryd(3S)	0.00217	0.97095
228	O	13	S	Ryd(4S)	0.00096	2.42198

229	O	13	S	Ryd(5S)	0.00000	49.29222
230	O	13	px	Val(2p)	1.53670	-0.52577
231	O	13	px	Ryd(3p)	0.00185	0.60339
232	O	13	px	Ryd(4p)	0.00090	1.44005
233	O	13	px	Ryd(5p)	0.00004	4.46649
234	O	13	py	Val(2p)	1.71814	-0.52864
235	O	13	py	Ryd(3p)	0.00353	0.47049
236	O	13	py	Ryd(4p)	0.00051	1.20377
237	O	13	py	Ryd(5p)	0.00002	4.73825
238	O	13	pz	Val(2p)	1.74081	-0.53664
239	O	13	pz	Ryd(3p)	0.00237	0.37399
240	O	13	pz	Ryd(4p)	0.00072	1.02729
241	O	13	pz	Ryd(5p)	0.00008	4.73201
242	O	13	dxy	Ryd(3d)	0.00184	1.78086
243	O	13	dxy	Ryd(4d)	0.00002	6.80136
244	O	13	dxz	Ryd(3d)	0.00523	1.85985
245	O	13	dxz	Ryd(4d)	0.00004	6.83673
246	O	13	dyz	Ryd(3d)	0.00570	1.74908
247	O	13	dyz	Ryd(4d)	0.00003	6.77668
248	O	13	dx2y2	Ryd(3d)	0.00145	1.70262
249	O	13	dx2y2	Ryd(4d)	0.00002	6.76837
250	O	13	dz2	Ryd(3d)	0.00407	1.63806
251	O	13	dz2	Ryd(4d)	0.00002	6.75092
252	O	14	S	Cor(1S)	1.99978	-19.06089
253	O	14	S	Val(2S)	1.81220	-1.17063
254	O	14	S	Ryd(3S)	0.00046	1.19507
255	O	14	S	Ryd(4S)	0.00010	2.26694
256	O	14	S	Ryd(5S)	0.00000	49.55547
257	O	14	px	Val(2p)	1.78399	-0.46748
258	O	14	px	Ryd(3p)	0.00120	0.66411
259	O	14	px	Ryd(4p)	0.00015	0.94992
260	O	14	px	Ryd(5p)	0.00001	4.89369
261	O	14	py	Val(2p)	1.49164	-0.51928
262	O	14	py	Ryd(3p)	0.00044	0.42906
263	O	14	py	Ryd(4p)	0.00023	1.25529
264	O	14	py	Ryd(5p)	0.00007	5.01663
265	O	14	pz	Val(2p)	1.71650	-0.46865
266	O	14	pz	Ryd(3p)	0.00099	0.44171
267	O	14	pz	Ryd(4p)	0.00004	0.90970
268	O	14	pz	Ryd(5p)	0.00001	4.82462
269	O	14	dxy	Ryd(3d)	0.00882	1.62224
270	O	14	dxy	Ryd(4d)	0.00003	6.76380
271	O	14	dxz	Ryd(3d)	0.00078	1.35400
272	O	14	dxz	Ryd(4d)	0.00000	6.58773
273	O	14	dyz	Ryd(3d)	0.01000	1.61810
274	O	14	dyz	Ryd(4d)	0.00002	6.75001
275	O	14	dx2y2	Ryd(3d)	0.01169	1.63481
276	O	14	dx2y2	Ryd(4d)	0.00001	6.76901
277	O	14	dz2	Ryd(3d)	0.00454	1.47204
278	O	14	dz2	Ryd(4d)	0.00001	6.67462
279	C	15	S	Cor(1S)	1.99875	-10.24042
280	C	15	S	Val(2S)	0.95979	-0.38466
281	C	15	S	Ryd(4S)	0.00245	1.07854

282	C	15	S	Ryd(3S)	0.00015	0.77493
283	C	15	S	Ryd(5S)	0.00001	22.11446
284	C	15	px	Val(2p)	1.02284	-0.30910
285	C	15	px	Ryd(4p)	0.00710	1.10245
286	C	15	px	Ryd(3p)	0.00018	0.88734
287	C	15	px	Ryd(5p)	0.00012	3.29469
288	C	15	py	Val(2p)	1.11666	-0.27155
289	C	15	py	Ryd(4p)	0.00600	1.02688
290	C	15	py	Ryd(3p)	0.00020	0.77680
291	C	15	py	Ryd(5p)	0.00015	3.34093
292	C	15	pz	Val(2p)	1.19179	-0.31398
293	C	15	pz	Ryd(3p)	0.00182	0.45454
294	C	15	pz	Ryd(4p)	0.00013	0.63150
295	C	15	pz	Ryd(5p)	0.00005	2.48438
296	C	15	dxy	Ryd(3d)	0.00109	2.11313
297	C	15	dxy	Ryd(4d)	0.00055	3.57198
298	C	15	dxz	Ryd(3d)	0.00095	1.03620
299	C	15	dxz	Ryd(4d)	0.00015	3.27178
300	C	15	dyz	Ryd(3d)	0.00068	1.07348
301	C	15	dyz	Ryd(4d)	0.00023	3.24437
302	C	15	dx2y2	Ryd(3d)	0.00156	1.99465
303	C	15	dx2y2	Ryd(4d)	0.00059	3.53506
304	C	15	dz2	Ryd(3d)	0.00208	1.56847
305	C	15	dz2	Ryd(4d)	0.00009	3.40045
306	C	16	S	Cor(1S)	1.99902	-10.19487
307	C	16	S	Val(2S)	0.94307	-0.32537
308	C	16	S	Ryd(4S)	0.00168	1.11058
309	C	16	S	Ryd(3S)	0.00013	0.88228
310	C	16	S	Ryd(5S)	0.00001	21.13207
311	C	16	px	Val(2p)	1.15299	-0.22286
312	C	16	px	Ryd(4p)	0.00637	1.12134
313	C	16	px	Ryd(3p)	0.00027	0.57079
314	C	16	px	Ryd(5p)	0.00011	3.26614
315	C	16	py	Val(2p)	1.09045	-0.22014
316	C	16	py	Ryd(4p)	0.00458	0.95525
317	C	16	py	Ryd(3p)	0.00028	0.55499
318	C	16	py	Ryd(5p)	0.00014	3.24116
319	C	16	pz	Val(2p)	0.96269	-0.26945
320	C	16	pz	Ryd(4p)	0.00144	0.45011
321	C	16	pz	Ryd(3p)	0.00029	0.27509
322	C	16	pz	Ryd(5p)	0.00004	2.67653
323	C	16	dxy	Ryd(3d)	0.00088	2.05198
324	C	16	dxy	Ryd(4d)	0.00072	3.63293
325	C	16	dxz	Ryd(3d)	0.00049	1.29320
326	C	16	dxz	Ryd(4d)	0.00007	2.98777
327	C	16	dyz	Ryd(3d)	0.00034	1.33990
328	C	16	dyz	Ryd(4d)	0.00013	3.00268
329	C	16	dx2y2	Ryd(3d)	0.00103	2.02710
330	C	16	dx2y2	Ryd(4d)	0.00031	3.57736
331	C	16	dz2	Ryd(3d)	0.00128	1.78590
332	C	16	dz2	Ryd(4d)	0.00006	3.25880
333	C	17	S	Cor(1S)	1.99902	-10.19063
334	C	17	S	Val(2S)	0.95042	-0.32348

335	C	17	S	Ryd(4S)	0.00172	1.12966
336	C	17	S	Ryd(3S)	0.00015	0.91567
337	C	17	S	Ryd(5S)	0.00001	20.96790
338	C	17	px	Val(2p)	1.06180	-0.21237
339	C	17	px	Ryd(4p)	0.00471	1.00105
340	C	17	px	Ryd(3p)	0.00044	0.43112
341	C	17	px	Ryd(5p)	0.00017	3.18783
342	C	17	py	Val(2p)	1.18820	-0.22122
343	C	17	py	Ryd(4p)	0.00670	1.04278
344	C	17	py	Ryd(3p)	0.00029	0.45705
345	C	17	py	Ryd(5p)	0.00007	3.38426
346	C	17	pz	Val(2p)	0.93448	-0.26279
347	C	17	pz	Ryd(4p)	0.00131	0.43100
348	C	17	pz	Ryd(3p)	0.00034	0.23095
349	C	17	pz	Ryd(5p)	0.00005	2.68679
350	C	17	dxy	Ryd(3d)	0.00112	2.01706
351	C	17	dxy	Ryd(4d)	0.00032	3.55454
352	C	17	dxz	Ryd(3d)	0.00043	1.36146
353	C	17	dxz	Ryd(4d)	0.00012	2.93125
354	C	17	dyz	Ryd(3d)	0.00045	1.32719
355	C	17	dyz	Ryd(4d)	0.00006	3.01796
356	C	17	dx2y2	Ryd(3d)	0.00084	2.03166
357	C	17	dx2y2	Ryd(4d)	0.00068	3.59137
358	C	17	dz2	Ryd(3d)	0.00127	1.79519
359	C	17	dz2	Ryd(4d)	0.00006	3.24910
360	C	18	S	Cor(1S)	1.99915	-10.18169
361	C	18	S	Val(2S)	0.95631	-0.32038
362	C	18	S	Ryd(4S)	0.00137	1.14435
363	C	18	S	Ryd(3S)	0.00011	0.74334
364	C	18	S	Ryd(5S)	0.00001	20.93488
365	C	18	px	Val(2p)	1.05100	-0.19474
366	C	18	px	Ryd(4p)	0.00411	0.95274
367	C	18	px	Ryd(3p)	0.00035	0.43011
368	C	18	px	Ryd(5p)	0.00019	3.16512
369	C	18	py	Val(2p)	1.18078	-0.20727
370	C	18	py	Ryd(4p)	0.00714	1.04261
371	C	18	py	Ryd(3p)	0.00018	0.36960
372	C	18	py	Ryd(5p)	0.00008	3.33750
373	C	18	pz	Val(2p)	0.97111	-0.25464
374	C	18	pz	Ryd(4p)	0.00078	0.45157
375	C	18	pz	Ryd(3p)	0.00019	0.21241
376	C	18	pz	Ryd(5p)	0.00005	2.63674
377	C	18	dxy	Ryd(3d)	0.00101	2.01033
378	C	18	dxy	Ryd(4d)	0.00035	3.49666
379	C	18	dxz	Ryd(3d)	0.00025	1.43386
380	C	18	dxz	Ryd(4d)	0.00010	2.87104
381	C	18	dyz	Ryd(3d)	0.00057	1.38710
382	C	18	dyz	Ryd(4d)	0.00003	2.94655
383	C	18	dx2y2	Ryd(3d)	0.00092	2.10723
384	C	18	dx2y2	Ryd(4d)	0.00061	3.56758
385	C	18	dz2	Ryd(3d)	0.00122	1.88769
386	C	18	dz2	Ryd(4d)	0.00005	3.19793
387	C	19	S	Cor(1S)	1.99915	-10.17915

388	C	19	S	Val(2S)	0.95735	-0.31796
389	C	19	S	Ryd(4S)	0.00139	1.08891
390	C	19	S	Ryd(3S)	0.00011	0.82721
391	C	19	S	Ryd(5S)	0.00000	20.92034
392	C	19	px	Val(2p)	1.12292	-0.19754
393	C	19	px	Ryd(4p)	0.00596	1.03521
394	C	19	px	Ryd(3p)	0.00028	0.40055
395	C	19	px	Ryd(5p)	0.00012	3.26231
396	C	19	py	Val(2p)	1.10849	-0.19905
397	C	19	py	Ryd(4p)	0.00528	0.96653
398	C	19	py	Ryd(3p)	0.00030	0.40925
399	C	19	py	Ryd(5p)	0.00014	3.21513
400	C	19	pz	Val(2p)	0.96939	-0.25186
401	C	19	pz	Ryd(4p)	0.00083	0.45584
402	C	19	pz	Ryd(3p)	0.00019	0.22628
403	C	19	pz	Ryd(5p)	0.00006	2.62419
404	C	19	dxy	Ryd(3d)	0.00091	2.10911
405	C	19	dxy	Ryd(4d)	0.00062	3.57995
406	C	19	dxz	Ryd(3d)	0.00042	1.38426
407	C	19	dxz	Ryd(4d)	0.00009	2.92381
408	C	19	dyz	Ryd(3d)	0.00041	1.41574
409	C	19	dyz	Ryd(4d)	0.00005	2.91808
410	C	19	dx2y2	Ryd(3d)	0.00103	2.01038
411	C	19	dx2y2	Ryd(4d)	0.00034	3.49695
412	C	19	dz2	Ryd(3d)	0.00123	1.87929
413	C	19	dz2	Ryd(4d)	0.00005	3.19784
414	C	20	S	Cor(1S)	1.99917	-10.18326
415	C	20	S	Val(2S)	0.96405	-0.32249
416	C	20	S	Ryd(3S)	0.00123	1.02489
417	C	20	S	Ryd(4S)	0.00014	1.28766
418	C	20	S	Ryd(5S)	0.00000	20.78804
419	C	20	px	Val(2p)	1.16525	-0.19899
420	C	20	px	Ryd(4p)	0.00664	1.01396
421	C	20	px	Ryd(3p)	0.00023	0.44679
422	C	20	px	Ryd(5p)	0.00007	3.31320
423	C	20	py	Val(2p)	1.06854	-0.19184
424	C	20	py	Ryd(4p)	0.00452	0.92912
425	C	20	py	Ryd(3p)	0.00043	0.42472
426	C	20	py	Ryd(5p)	0.00014	3.17915
427	C	20	pz	Val(2p)	0.91076	-0.25014
428	C	20	pz	Ryd(4p)	0.00060	0.45053
429	C	20	pz	Ryd(3p)	0.00017	0.24085
430	C	20	pz	Ryd(5p)	0.00006	2.59182
431	C	20	dxy	Ryd(3d)	0.00096	2.01455
432	C	20	dxy	Ryd(4d)	0.00047	3.51644
433	C	20	dxz	Ryd(3d)	0.00053	1.39647
434	C	20	dxz	Ryd(4d)	0.00003	2.91237
435	C	20	dyz	Ryd(3d)	0.00036	1.47208
436	C	20	dyz	Ryd(4d)	0.00009	2.87821
437	C	20	dx2y2	Ryd(3d)	0.00096	2.03351
438	C	20	dx2y2	Ryd(4d)	0.00049	3.52641
439	C	20	dz2	Ryd(3d)	0.00115	1.83927
440	C	20	dz2	Ryd(4d)	0.00006	3.16158

441	H	21	S	Val(1S)	0.77199	-0.10244
442	H	21	S	Ryd(3S)	0.00091	1.61442
443	H	21	S	Ryd(2S)	0.00013	1.37107
444	H	21	px	Ryd(2p)	0.00054	1.59273
445	H	21	px	Ryd(3p)	0.00018	3.96822
446	H	21	py	Ryd(2p)	0.00028	1.48364
447	H	21	py	Ryd(3p)	0.00014	3.92011
448	H	21	pz	Ryd(2p)	0.00022	0.87206
449	H	21	pz	Ryd(3p)	0.00005	3.67847
450	H	22	S	Val(1S)	0.74702	-0.07723
451	H	22	S	Ryd(3S)	0.00063	1.53921
452	H	22	S	Ryd(2S)	0.00014	1.36030
453	H	22	px	Ryd(2p)	0.00013	1.26194
454	H	22	px	Ryd(3p)	0.00008	3.94760
455	H	22	py	Ryd(2p)	0.00048	1.85042
456	H	22	py	Ryd(3p)	0.00026	3.95021
457	H	22	pz	Ryd(2p)	0.00015	0.99934
458	H	22	pz	Ryd(3p)	0.00003	3.56244
459	H	23	S	Val(1S)	0.76592	-0.07789
460	H	23	S	Ryd(2S)	0.00033	1.38417
461	H	23	S	Ryd(3S)	0.00014	1.53736
462	H	23	px	Ryd(2p)	0.00014	1.31326
463	H	23	px	Ryd(3p)	0.00005	3.90942
464	H	23	py	Ryd(2p)	0.00050	1.94269
465	H	23	py	Ryd(3p)	0.00029	3.80970
466	H	23	pz	Ryd(2p)	0.00010	0.85903
467	H	23	pz	Ryd(3p)	0.00007	3.68955
468	H	24	S	Val(1S)	0.76331	-0.07258
469	H	24	S	Ryd(2S)	0.00032	1.40167
470	H	24	S	Ryd(3S)	0.00014	1.50536
471	H	24	px	Ryd(2p)	0.00036	1.73247
472	H	24	px	Ryd(3p)	0.00020	3.85356
473	H	24	py	Ryd(2p)	0.00026	1.52771
474	H	24	py	Ryd(3p)	0.00014	3.86648
475	H	24	pz	Ryd(2p)	0.00009	0.87236
476	H	24	pz	Ryd(3p)	0.00007	3.69046
477	H	25	S	Val(1S)	0.74009	-0.11266
478	H	25	S	Ryd(3S)	0.00049	1.51227
479	H	25	S	Ryd(2S)	0.00009	1.28304
480	H	25	px	Ryd(2p)	0.00028	1.50497
481	H	25	px	Ryd(3p)	0.00018	3.81715
482	H	25	py	Ryd(2p)	0.00015	1.12096
483	H	25	py	Ryd(3p)	0.00006	3.56444
484	H	25	pz	Ryd(2p)	0.00029	1.55709
485	H	25	pz	Ryd(3p)	0.00018	3.84677
486	H	26	S	Val(1S)	0.76745	-0.07291
487	H	26	S	Ryd(3S)	0.00023	1.68761
488	H	26	S	Ryd(2S)	0.00012	1.23623
489	H	26	px	Ryd(2p)	0.00042	1.86301
490	H	26	px	Ryd(3p)	0.00027	3.82170

491 H 26 py Ryd(2p) 0.00018 1.35157
492 H 26 py Ryd(3p) 0.00008 3.88859
493 H 26 pz Ryd(2p) 0.00006 0.84756
494 H 26 pz Ryd(3p) 0.00006 3.69266

495 H 27 S Val(1S) 0.76740 -0.10210
496 H 27 S Ryd(3S) 0.00033 1.44723
497 H 27 S Ryd(2S) 0.00006 1.25430
498 H 27 px Ryd(2p) 0.00022 2.73604
499 H 27 px Ryd(3p) 0.00018 2.88099
500 H 27 py Ryd(2p) 0.00017 2.14296
501 H 27 py Ryd(3p) 0.00007 2.72439
502 H 27 pz Ryd(2p) 0.00019 2.22305
503 H 27 pz Ryd(3p) 0.00008 2.75580

WARNING: 1 low occupancy (<1.9990e) core orbital found on C 1
1 low occupancy (<1.9990e) core orbital found on C 2
1 low occupancy (<1.9990e) core orbital found on S 6
1 low occupancy (<1.9990e) core orbital found on C 15

WARNING: Population inversion found on atom C 1
Population inversion found on atom C 2
Population inversion found on atom C 4
Population inversion found on atom C 5
Population inversion found on atom S 6
Population inversion found on atom H 7
Population inversion found on atom H 8
Population inversion found on atom H 9
Population inversion found on atom H 10
Population inversion found on atom H 11
Population inversion found on atom H 12
Population inversion found on atom C 15
Population inversion found on atom C 16
Population inversion found on atom C 17
Population inversion found on atom C 18
Population inversion found on atom C 19
Population inversion found on atom C 20
Population inversion found on atom H 21
Population inversion found on atom H 22
Population inversion found on atom H 25
Population inversion found on atom H 26
Population inversion found on atom H 27

Summary of Natural Population Analysis:

Atom	No	Natural Charge	Natural Population Core	Valence	Rydberg	Total
C	1	-0.43032	1.99885	4.40475	0.02672	6.43032
C	2	-0.10456	1.99893	4.08336	0.02227	6.10456
C	3	0.25892	1.99907	3.71970	0.02231	5.74108
C	4	-0.59010	1.99919	4.57352	0.01738	6.59010
C	5	-0.58520	1.99919	4.56856	0.01744	6.58520

S	6	2.06898	9.99795	3.75409	0.17898	13.93102
H	7	0.27152	0.00000	0.72618	0.00229	0.72848
H	8	0.23617	0.00000	0.76214	0.00169	0.76383
H	9	0.22826	0.00000	0.76969	0.00205	0.77174
H	10	0.21582	0.00000	0.78231	0.00187	0.78418
H	11	0.23723	0.00000	0.76115	0.00162	0.76277
H	12	0.23416	0.00000	0.76456	0.00128	0.76584
O	13	-0.73952	1.99977	6.70819	0.03156	8.73952
O	14	-0.84373	1.99978	6.80434	0.03961	8.84373
C	15	-0.31619	1.99875	4.29107	0.02637	6.31619
C	16	-0.16886	1.99902	4.14919	0.02066	6.16886
C	17	-0.15525	1.99902	4.13491	0.02132	6.15525
C	18	-0.17800	1.99915	4.15919	0.01966	6.17800
C	19	-0.17711	1.99915	4.15816	0.01980	6.17711
C	20	-0.12709	1.99917	4.10860	0.01933	6.12709
H	21	0.22558	0.00000	0.77199	0.00243	0.77442
H	22	0.25107	0.00000	0.74702	0.00190	0.74893
H	23	0.23247	0.00000	0.76592	0.00161	0.76753
H	24	0.23512	0.00000	0.76331	0.00158	0.76488
H	25	0.25818	0.00000	0.74009	0.00173	0.74182
H	26	0.23114	0.00000	0.76745	0.00140	0.76886
H	27	0.23129	0.00000	0.76740	0.00130	0.76871

===

* Total *	1.00000	35.98700	73.50685	0.50615	110.00000

Natural Population

Core	35.98700 (99.9639% of 36)
Valence	73.50685 (99.3336% of 74)
Natural Minimal Basis	109.49385 (99.5399% of 110)
Natural Rydberg Basis	0.50615 (0.4601% of 110)

Atom	No	Natural Electron Configuration
C	1	[core]2S(1.06)2p(3.34)3d(0.01)4p(0.01)
C	2	[core]2S(0.97)2p(3.11)3d(0.01)4p(0.01)
C	3	[core]2S(0.94)2p(2.78)3p(0.01)3d(0.01)
C	4	[core]2S(1.09)2p(3.49)3d(0.01)4p(0.01)
C	5	[core]2S(1.08)2p(3.48)3d(0.01)4p(0.01)
S	6	[core]3S(1.12)3p(2.63)3d(0.15)6p(0.02)
H	7	1S(0.73)
H	8	1S(0.76)
H	9	1S(0.77)
H	10	1S(0.78)
H	11	1S(0.76)
H	12	1S(0.76)
O	13	[core]2S(1.71)2p(5.00)3p(0.01)3d(0.02)
O	14	[core]2S(1.81)2p(4.99)3d(0.04)
C	15	[core]2S(0.96)2p(3.33)3d(0.01)4p(0.01)
C	16	[core]2S(0.94)2p(3.21)4p(0.01)
C	17	[core]2S(0.95)2p(3.18)4p(0.01)
C	18	[core]2S(0.96)2p(3.20)4p(0.01)
C	19	[core]2S(0.96)2p(3.20)4p(0.01)
C	20	[core]2S(0.96)2p(3.14)4p(0.01)

H	21	1S(0.77)
H	22	1S(0.75)
H	23	1S(0.77)
H	24	1S(0.76)
H	25	1S(0.74)
H	26	1S(0.77)
H	27	1S(0.77)

V. References

1. Dolomanov, O. V.; Bourhis, L. J.; Gildea, R. J.; Howard, J. A. K.; Puschmann, H. *J. Appl. Cryst.* **2009**, *42*, 339-341. doi: 10.1107/S0021889808042726;

2. Sheldrick, G. M. *Acta Cryst.* **2008**, *A64*, 112-122. doi: 10.1107/S0108767307043930

3. Frisch, M. J.; Trucks, G. W.; Schlegel, H. B.; Scuseria, G. E.; Robb, M. A.; Cheeseman, J. R.; Scalmani, G.; Barone, V.; Mennucci, B.; Petersson, G. A.; Nakatsuji, H.; Caricato, M.; Li, X.; Hratchian, H. P.; Izmaylov, A. F.; Bloino, J.; Zheng, G.; Sonnenberg, J. L.; Hada, M.; Ehara, M.; Toyota, K.; Fukuda, R.; Hasegawa, J.; Ishida, M.; Nakajima, T.; Honda, Y.; Kitao, O.; Nakai, H.; Vreven, T.; Montgomery, Jr., J. A.; Peralta, J. E.; Ogliaro, F.; Bearpark, M.; Heyd, J. J.; Brothers, E.; Kudin, K. N.; Staroverov, V. N.; Keith, T.; Kobayashi, R.; Normand, J.; Raghavachari, K.; Rendell, A.; Burant, J. C.; Iyengar, S. S.; Tomasi, J.; Cossi, M.; Rega, N.; Millam, J. M.; Klene, M.; Knox, J. E.; Cross, J. B.; Bakken, V.; Adamo, C.; Jaramillo, J.; Gomperts, R.; Stratmann, R. E.; Yazyev, O.; Austin, A. J.; Cammi, R.; Pomelli, C.; Ochterski, J. W.; Martin, R. L.; Morokuma, K.; Zakrzewski, V. G.; Voth, G. A.; Salvador, P.; Dannenberg, J. J.; Dapprich, S.; Daniels, A. D.; Farkas, O.; Foresman, J. B.; Ortiz, J. V.; Cioslowski, J.; Fox, D. J.; *Gaussian 09, Revision C.01*, Gaussian, Inc., Wallingford CT, **2010**.

4. 1. Ma, S.; Ren, H.; Wei, Q. *J. Am. Chem. Soc*. 2003, 125, 4817−4830. doi: 10.1021/ja034039q

5. Buynak J.D.; Rao M. N. *J. Org. Chem.* **1986**, *51*, 1571-1574. doi: 10.1021/jo00359a036

6. Swamy K. C; Suresh R.R.; Gangadhararao G.; *Synthesis* **2017**, *49*, 2275-2285. doi: 10.1055/s-0036-1558951

7. Denmark, S. E.; Harmata, M. A.; White, K. S. *J. Org. Chem.* **1987**, *52*, 4031-4042. doi: 10.1021/jo00227a017

8. Hu C.; Chen Y. *Org. Chem. Front.* **2015**, 2, 1352-1355. doi: 10.1039/C5QO00187K

9. Pal A.; Koduri N.D.; Wang Z.; Quiroz E.L.; Chong A.; Vuong M.; Rajagopal N.; Nguyen M.; Roberts K.P.; Hussaini S.R. *Tetrahedron Lett.* **2017**, *58*, 586-589. doi: 10.1016/j.tetlet.2017.01.004

10. Fang Z.; Zhou C.; Fu C.; Ma S. *Org. Biomol. Chem.* **2010**, *8*, 4554-4561. doi: 10.1039/C0OB00007H
